# Biotransformation of a potent anabolic steroid, mibolerone, with *Cunninghamella blakesleeana*, *C*. *echinulata*, and *Macrophomina phaseolina*, and biological activity evaluation of its metabolites

**DOI:** 10.1371/journal.pone.0171476

**Published:** 2017-02-24

**Authors:** Mahwish Siddiqui, Malik Shoaib Ahmad, Atia-tul- Wahab, Sammer Yousuf, Narjis Fatima, Nimra Naveed Shaikh, Atta-ur- Rahman, M. Iqbal Choudhary

**Affiliations:** 1 H. E. J. Research Institute of Chemistry, International Center for Chemical and Biological Sciences, University of Karachi, Karachi, Pakistan; 2 Dr. Panjwani Center for Molecular Medicine and Drug Research, International Center for Chemical and Biological Sciences, University of Karachi, Karachi, Pakistan; 3 Department of Biochemistry, Faculty of Science, King Abdulaziz University, Jeddah, Saudi Arabia; Aligarh Muslim University, INDIA

## Abstract

Seven metabolites were obtained from the microbial transformation of anabolic-androgenic steroid mibolerone (**1**) with *Cunninghamella blakesleeana*, *C*. *echinulata*, and *Macrophomina phaseolina*. Their structures were determined as 10*β*,17*β*-dihydroxy-7*α*,17*α*-dimethylestr-4-en-3-one (**2**), 6*β*,17*β*-dihydroxy-7*α*,17*α*-dimethylestr-4-en-3-one (**3**), 6*β*,10*β*,17*β*-trihydroxy-7*α*,17*α*-dimethylestr-4-en-3-one (**4**), 11*β*,17*β*-dihydroxy-(20-hydroxymethyl)-7*α*,17*α*-dimethylestr-4-en-3-one (**5**), 1*α*,17*β*-dihydroxy-7*α*,17*α*-dimethylestr-4-en-3-one (**6**), 1*α*,11*β*,17*β*-trihydroxy-7*α*,17*α*-dimethylestr-4-en-3-one (**7**), and 11*β*,17*β*-dihydroxy-7*α*,17*α*-dimethylestr-4-en-3-one (**8**), on the basis of spectroscopic studies. All metabolites, except **8**, were identified as new compounds. This study indicates that *C*. *blakesleeana*, and *C*. *echinulata* are able to catalyze hydroxylation at allylic positions, while *M*. *phaseolina* can catalyze hydroxylation of CH_2_ and CH_3_ groups of substrate **1**. Mibolerone (**1**) was found to be a moderate inhibitor of *β*-glucuronidase enzyme (IC_50_ = 42.98 ± 1.24 μM) during random biological screening, while its metabolites **2**–**4**, and **8** were found to be inactive. Mibolerone (**1**) was also found to be significantly active against *Leishmania major* promastigotes (IC_50_ = 29.64 ± 0.88 μM). Its transformed products **3** (IC_50_ = 79.09 ± 0.06 μM), and **8** (IC_50_ = 70.09 ± 0.05 μM) showed a weak leishmanicidal activity, while **2** and **4** were found to be inactive. In addition, substrate **1** (IC_50_ = 35.7 ± 4.46 μM), and its metabolite **8** (IC_50_ = 34.16 ± 5.3 μM) exhibited potent cytotoxicity against HeLa cancer cell line (human cervical carcinoma). Metabolite **2** (IC_50_ = 46.5 ± 5.4 μM) also showed a significant cytotoxicity, while **3** (IC_50_ = 107.8 ± 4.0 μM) and **4** (IC_50_ = 152.5 ± 2.15 μM) showed weak cytotoxicity against HeLa cancer cell line. Compound **1** (IC_50_ = 46.3 ± 11.7 μM), and its transformed products **2** (IC_50_ = 43.3 ± 7.7 μM), **3** (IC_50_ = 65.6 ± 2.5 μM), and **4** (IC_50_ = 89.4 ± 2.7 μM) were also found to be moderately toxic to 3T3 cell line (mouse fibroblast). Interestingly, metabolite **8** showed no cytotoxicity against 3T3 cell line. Compounds **1**–**4**, and **8** were also evaluated for inhibition of tyrosinase, carbonic anhydrase, and *α*-glucosidase enzymes, and all were found to be inactive.

## Introduction

Mibolerone (7*α*,17*α*-dimethyl-19-nortestosterone) (**1**) is a potent synthetic anabolic and androgenic steroid, marketed by the Upjohn Company under the brand name of Check Drops, for the treatment of estrous (heat) in female dogs. It is stable to metabolic conversion in the rat ventral prostate. Because of its stability and high affinity binding, it has been used as a ligand for the characterization and quantitation of androgen in prostate, liver, and cultured cells. In addition, mibolerone (**1**) is more receptor-selective for androgenic receptor than methyltrienolone. The binding interaction of compound **1** with testosterone-estradiol binding globulin of human serum is weaker than 5*α*-dihydrotestosterone. Mibolerone (**1**) also acts through the progesterone receptor (PR) as it eliminates progesterone receptor expression at lower doses (1 nM), in contrast to 5*α*-dehydrotestosterone (10–100 nM), which reduces PR to basal levels. In breast cancer cells, mibolerone (**1**) has shown a dual action, *i*.*e*., androgenic and progestagenic [1–4].

The regio-, chemo-, and stereo-selective synthesis of organic compounds has been an area of active research since several decades. Many of these conversions are difficult to achieve through conventional synthetic methodologies. However, biocatalysts can carry out these reactions effectively. Biocatalysis has several advantages over chemical synthesis, such as selectivity, mild reaction conditions, and their eco-friendly nature. Various biocatalysts, such as pure enzymes and whole-cell systems, are being used for the transformation of organic compounds. However, whole-cell biocatalysis, especially with fungi, is an efficient choice for regio- and stereo-selective transformations [5–9], as they have P450 cytochrome enzyme systems, which catalyze hydroxylation at various positions of steroids [10–12].

**Fig 1:**
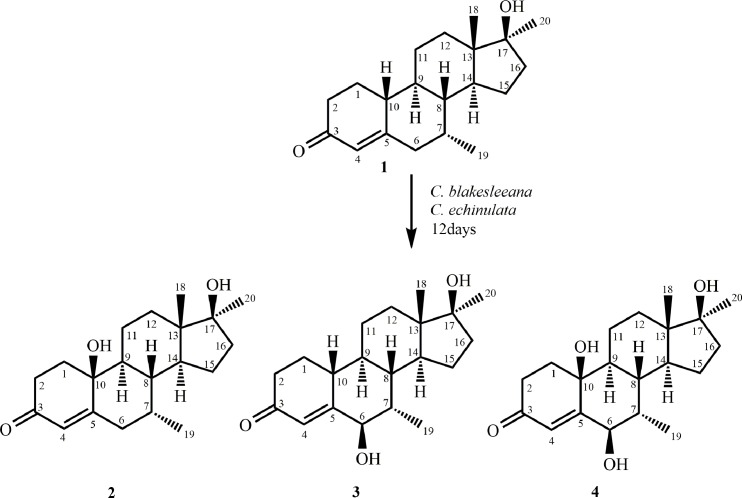
Compounds 2–4 obtained by the biotransformation of mibolerone (1) with *Cunninghamella blakesleeana* and *C*. *echinulata*.

**Fig 2:**
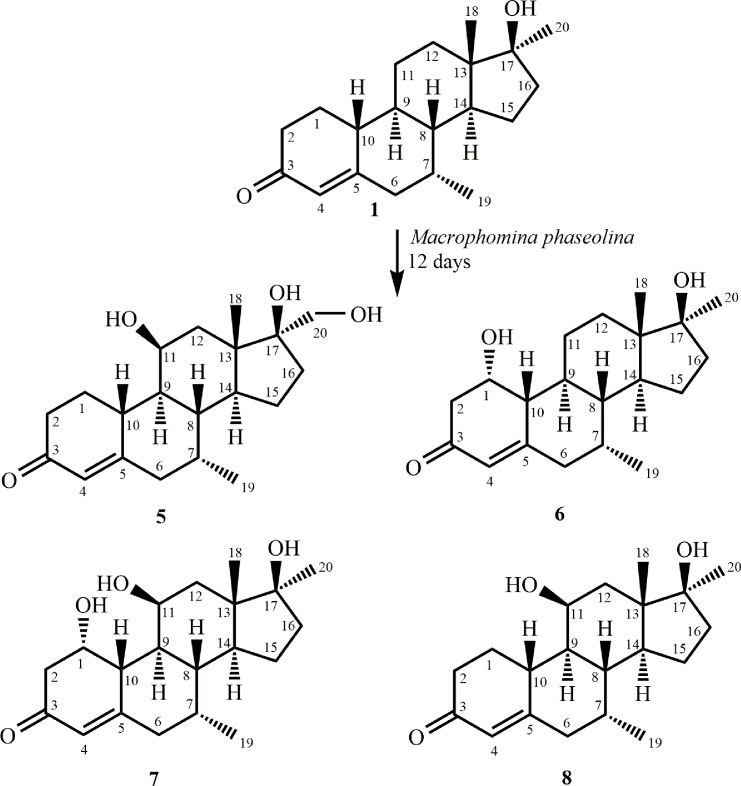
Compounds 5–8 obtained by the biotransformation of mibolerone (1) with *Macrophomina phaseolina*.

In continuation of our research on the fungal transformation of bioactive steroids [13–17], we incubated mibolerone (**1**) (C_20_H_30_O_2_), with *Cunninghamella blakesleeana*, *C*. *echinulata*, and *Macrophomina phaseolina*. Compounds **2**–**4** (Fig 1) were obtained on incubation of substrate **1** with *C*. *blakesleeana*, and *C*. *echinulata*, whereas metabolites **5**–**8** (Fig 2) were obtained on the transformation of substrate **1** with *M*. *phaseolina*. Metabolites **2**–**7** were found to be new, whereas metabolite **8** was identified as a known compound. Interestingly, substrate **1** was found to be active against *β*-glucuronidase enzyme, *Leishmania major* promastigotes, and HeLa (cervical cancer) and 3T3 (normal) cell lines in preliminary assays.

*β*-Glucuronidase is an inducible exoglycosidase enzyme. Its increased level in blood can create problems in the detoxification process of various toxic substances. Toxic carcinogenic substances, along with endogenously produced toxic metabolites such as steroids, are metabolized in the liver. Before their excretion into the small intestine *via* the bile, these substances undergo conjugation with glucuronic acid. *β*-Glucuronidase, produced by intestinal bacteria, catalyzes the hydrolysis of these conjugates in the colon. Increased activity of *β*-glucuronidase is one of the key observations in colon cancer. Hence, *β*-glucuronidase plays a key role in the etiology of colon cancer [18–22].

Leishmaniasis, a neglected tropical disease (NTD), is a major vector borne disease of protozoal origin. Nearly 350 million people in 88 countries are at risk of the disease. Orally available drugs for the treatment of leishmaniasis are very few and often less effective. Therefore, development of safe and effective new therapeutic agents for leishmaniasis is urgently needed to reduce the burden of the disease [23–25].

After breast cancer, cervical cancer is the second most prevalent cancer in women across the world. In this cancer, malignant cells are formed in tissues of the cervix. Currently, radiotherapy, surgery, and most commonly, cisplatin based chemotherapic agents are used for the treatment of cancers. However, the response rate to chemotherapy is often very low due to frequent development of resistance of cancer cells against chemotherapeutic agents. HeLa cell line, obtained from human cervical cancer, provide a useful model to evaluate the cytotoxic potential of chemical compounds against cervical cancer *in vitro* [26–29].

## Experimental

### Chemicals and reagents

Mibolerone (**1**) was purchased from the Bettersyn Co., Ltd (China). Media ingredients were purchased from Daejung Chemicals and Metals Co., Ltd. (Korea), Oxoid Ltd. (England), and Sigma-Aldrich (Germany).

### Chromatographic protocols

The purity of compound **1** and the degree of its transformations were analyzed by TLC (Thin layer chromatography) (silica gel, 20×20, 0.25 mm thick, PF_254,_ Merck, Germany), while silica gel (70–230 mesh, Merck, Germany) was used for column chromatography. Compounds were finally purified on a recycling HPLC (JAI LC-908W, Japan), equipped with YMC L-80 (4–5 μm, 20−50 mm i.d.). Ceric sulphate reagent was used for visualizing the compounds on TLCs. All solvents used for chromatography were of analytical grade.

### Instrumental analysis

^1^H- (400, 500, and 600 MHz), and ^13^C-NMR (100, 125, and 150 MHz) and 2D-NMR spectra were recorded on Bruker Avance-NMR spectrometers (France) in CD_3_OD, CD_3_COCD_3_ or DMSO-*d*_*6*_. Melting points were recorded on Buchi M-560 apparatus (Japan). EI- and HREI-MS were recorded on JEOL JMS-600H (Japan). Optical rotations of all isolated compounds were measured on JASCO P-2000 polarimeter (Japan) in chloroform or methanol. IR analyses were performed on Bruker Vector 22 FT-IR spectrometer (France). Evolution 300 UV-visible spectrophotometer was used to record the UV spectra (Thermo Scientific, England). Single-crystal X-ray diffraction data was collected on Bruker APEXII D8 Venture diffractometer, fitted with PHOTON 100 detector (CMOS technology), and fine-focus sealed tube having X-ray source [Cu K*α* radiation *α* = 1.54178 Å]. Reflection intensities were integrated using SAINT software. Absorption correction was done on M-multi-scan. Structures were solved on SHELXTL [30–31].

Crystallographic data for compounds **1**, **2**, **4**, and **8** were deposited with the Cambridge Crystallographic Data Center and can be obtained free of charge from the Cambridge Crystallographic Data Center *via* www.ccdc.cam.ac.uk/data_request/cif.

### Fungal cultures

Fungal strains of *Cunninghamella blakesleeana* (ATCC 8688A), and *Cunninghamella echinulata* (ATCC 9244) were obtained from the American Type Culture Collection (ATCC), whereas *Macrophomina phaseolina* (KUCC 730) was obtained from the Karachi University Culture Collection (KUCC). All cultures were stored on Sabouraud dextrose agar (SDA) at 4°C.

### General fermentation protocol

The ingredients used for 1 L culture medium were comprised 10 g glucose, 5 g peptone, 5 g KH_2_PO_4_, 5 g yeast extract, 5 g NaCl, and 10 mL glycerol in 1 L of distilled water.

The aforementioned ingredients were used to prepare the culture medium for the growth of *C*. *blakesleeana*, *C*. *echinulata*, and *M*. *phaseolina*. The experiments were carried out on two scales, *i*.*e*., the experimental and the preparative scales. In the experimental scale, 600 mL media was prepared for each fungus, transferred to 6 flasks of 250 mL, and autoclaved. Two flasks served as positive (fungal media + substrate) and negative (fully grown fungus in media) controls, whereas the remaining four flasks were used as test flasks. The fungi were grown in test flasks and negative control by transferring its spores. After mature growth of the fungi, 20 mg of the substrate was dissolved in 0.5 mL of methanol and incubated in each culture-containing test flask. One test flask was harvested every 4^th^ day, followed by filtration of the mycelia, and extraction with dichloromethane (DCM). Based on the small scale screening, substrate **1** was subjected to preparative scale transformation.

Four liters of liquid media was prepared for each fungus, and distributed equally in 40 flasks of 250 mL each. The media was then autoclaved and inoculated with spores of the appropriate fungus at 22°C. After 4 days of inoculation, fungus cultures were found to be fully matured. Substrate **1** (600 mg) was dissolved in 20 mL methanol, and dispensed equally (0.5 mL in each) in all flasks. These flasks were then placed on a rotatory shaker for 12 days at 22°C.

### Extraction of metabolites

On the12^th^ day, the content of all flasks of each fungus were combined and filtered to remove the mycelia. The aqueous layer was extracted thrice with CH_2_Cl_2_ (24 L). The crude extract was made moisture free by adding sodium sulphate, filtered, and concentrated on a rotary evaporator, which yielded a thick brown gum like material.

### Isolation of metabolites of mibolerone (1) from the incubation of mibolerone (1) with *Cunninghamella blakesleeana*, and *C*. *echinulata*

Incubation of substrate **1** with *C*. *blakesleeana*, and *C*. *echinulata* yielded about 2 g of crude extracts. The extracts were subjected to silica gel column chromatography. The mobile phase comprised hexanes-acetone mixtures. The polarity of the mobile phase was increased by increasing the concentration of acetone (5–100% gradient of acetone). 500 mL of solvent system at each concentration was passed through the column. The fractions obtained were analyzed by TLC analysis, and the fractions of similar composition were pooled together. Three main fractions (1–3) were obtained, which were then purified on a reverse phase recycling HPLC. Metabolite **2** (*R*_*T*_ = 26 min, MeOH: H_2_O; 70: 30) was obtained from fraction 1. Fraction 2 yielded compound **3** (*R*_*T*_ = 23 min, MeOH: H_2_O; 70: 30), and compound **4** (*R*_*T*_ = 22 min, MeOH: H_2_O; 70: 30) was obtained from fraction 3 on purification with reverse phase recycling HPLC.

#### 17*β*-Hydroxy-7*α*,17*α*-dimethylestr-4-en-3-one (1)

Single-crystal X-ray data: empirical formula C_20_H_30_O_2_; formula weight 302.44; crystal system orthorhombic, space group P2_1_2_1_2_1_, unit cell, **a** = 8.2316(5) Å, **b** = 9.8785(6) Å, **c** = 20.6304(12) Å, *α* = *β* = *γ* = 90°, volume 1677.58(17) A^3^, Z = 4, calculated density 1.197 mg/m^3^, F(000) 664, crystal size 0.32 x 0.24 x 0.12 mm, *Ɵ* range for data collection 4.29 to 79.81°, reflections collected 20,227, unique reflections 3,597 (R_int_ = 0.0976), goodness-of-fit on F^2^ 1.097, final R indices [I>2δ(I)] R_1_ = 0.0661, wR2 = 0.2004, R indices R1 = 0.0695, wR2 = 0.2074 for all data, largest diff. peak and hole 0.599 and -0.620 e.A^-3^.

#### 10*β*,17*β*-Dihydroxy-7*α*,17*α*-dimethylestr-4-en-3-one (2)

White crystalline; yield (percentage yield): 40 mg (4%); melting point 200 ‒ 201°C; UV λ_max_ (log ε): 235 (4.38); ${\left[\alpha \right]}_{D}^{25}$ = ‒ 22.5 (*c* 0.004, CH_3_OH); IR (CHCl_3_) υ_max_; 3360 (O-H), 1654 (*α*,*β*-unsaturated ketone); EI-MS *m/z* (rel. int., %): 318.2 [M^+^] (59), 300.0 (63), 290.2 (23), 230.2 (47), 229.1 (64), 43.9 (100); HREI-MS *m/z*: 318.2192 [M^+^] (mol. formula C_20_H_30_O_3_, calcd. 318.2195); ^1^H-NMR (CD_3_OD, 400 MHz): Table 1; ^13^C-NMR (CD_3_OD, 125 MHz): Table 1. Single-crystal X-ray data: empirical formula C_20_H_30_O_3_; formula weight 318.44; crystal system orthorhombic space group P2_1_2_1_2_1_, unit cell, **a** = 8.338(2) Å, **b** = 9.733(2) Å, **c** = 20.949(5) Å, *α* = *β* = γ = 90°, volume 1700.1(7) A^3^, Z = 4, calculated density 1.244 mg/m^3^, F(000) 696, crystal size 0.31 x 0.11 x 0.07 mm, *Ɵ* range for data collection 4.22 to 43.50°, reflections collected 3,259, unique reflections 770 (R_int_ = 0.0465), goodness-of-fit on F^2^ 1.119, final R indices [I>2δ(I)] R1 = 0.0418, wR2 = 0.1008, R indices R1 = 0.0458, wR2 = 0.1035 for all data, largest diff. peak and hole 0.294 and -0.200 e.A^-3^.

**Table 1:** ^13^C- and ^1^H-NMR chemical shift data (*J* in Hz) of compounds 1–4 (*δ* ppm).

Position	1		2		3		4		
	*δ*_C_	*δ*_H_ (*J* in Hz)	*δ*_C_	*δ*_H_ (*J* in Hz)	*δ*_C_	*δ*_H_ (*J* in Hz)	*δ*_C_	*δ*_H_ (*J* in Hz)	
1	27.8	1.94, m; 2.35, overlap	34.5	1.92, m; 2.22, m	27.3	1.32, m; 1.61, m	34.1		1.84, overlap 2.16, m
2	32.5	1.31, overlap; 1.51, overlap	34.5	2.27, m; 2.56, ddd (*J*_1/2_ = 4.8, *J*_1/3_ = 16.8)	37.2	2.32, m; 2.40, m	34.7		2.26, m; 2. 63, m
3	202.5	─	202.0	─	202.9	─	202.3		─
4	126.6	5.79, s	126.7	5.72, s	127.4	5.86, d (*J*_4/6_ = 2)	127.9		5.83, s
5	169.4	─	166.7	─	167.3	─	160.6		─
6	44.5	2.33, overlap; 2.56, dd (*J*_6/6_ = 14, *J*_6/7_ = 4.8)	41.0	2.10, m; 2.90, dd (*J*_6/6_ = 13.2, *J*_6/7_ = 3.6)	78.7	3.97, d (*J*_6/7_ = 3)	80.1		4.08, s
7	32.1	1.99, m	39.5	2.02, m	38.4	1.95, m	38.6		2.01, m
8	44.6	1.66, overlap	32.1	1.99, m	37.4	2.14, ddd (*J*_8a/9a_ = *J*_8a/14a_ = 11.5, *J*_8a/7e_ = 4)	33.6		2.30, m
9	43.6	1.83, overlap	47.4	1.41, m	43.3	1.14, m	46.6		1.35, m
10	44.2	2.15, m	70.8	─	39.8	2.50, m	72.4		─
11	23.3	1.57, overlap; 1.86, overlap	21.0	1.70, m; 1.73, m	23.2	1.44, m; 1.54, m	20.8		1.67, overlap; 1.78, m
12	39.1	1.69, overlap; 1.86, overlap	32.3	1.29, overlap; 1.56, overlap	32.5	1.30, m; 1.52, m	32.1		1.32, overlap; 1.54, m
13	46.9	─	46.7	─	47.1	─	46.9		─
14	47.4	1.42, overlap	46.6	1.32, overlap	46.7	1.42, m	46.6		1.45, m
15	27.7	1.34, overlap; 1.53, overlap	23.6	1.30, overlap; 1.57, overlap	27.6	1.89, m; 2.30, m	23.5		1.35, m; 1.57, m
16	37.4	2.32, overlap; 2.36, overlap	39.1	1.67, m; 1.84, m	39.1	1.67, m; 1.87, m	39.0		1.69, m; 1.88, m
17	82.1	─	82.1	─	82.1	─	82.1		─
18	14.5	0.92, s	14.3	0.92, s	14.5	0.94, m	14.3		0.93, s
19	13.1	0.79, d (*J*_19/7*β*_ = 7.2)	12.6	0.79, d (*J*_19/7*β*_ = 6.8)	11.1	0.74, d (*J*_19/7*β*_ = 7.5)	10.9		0.75, d (*J*_19/7*β*_ = 7.2)
20	26.1	1.29, s	26.1	1.18, s	26.1	1.18, s	26.1		1.19, s

a = ^1^H-NMR (400 MHz), and ^13^C-NMR (125 MHz)

b = ^1^H-NMR (500 MHz), and ^13^C-NMR (125 MHz)

#### 6*β*,17*β*-Dihydroxy-7*α*,17*α*-dimethylestr-4-en-3-one (3)

White solid; yield (percentage yield): 20 mg (2%); melting point 219‒221°C; UV λ_max_ (log ε): 230 (4.27); ${\left[\alpha \right]}_{D}^{25}$ = ‒ 134.2 (*c* 0.0014, CH_3_OH); IR (CHCl_3_) υ_max_; 3431 (O-H), 1659 (*α*,*β*-unsaturated ketone); EI-MS *m/z* (rel. int., %): 318.3 [M^+^] (16), 300.3 (100), 242.2 (39), 229.2 (98), 174.1 (41), 147.0 (27), 136.0 (26), 95.0 (21), 42.9 (54); HRESI-MS *m/z*: 318.2196 [M^+^] (mol. formula C_20_H_30_O_3_, calcd. 318.2195); ^1^H-NMR (CD_3_OD, 500 MHz): Table 1; ^13^C-NMR (CD_3_OD, 125 MHz): Table 1.

#### 6*β*,10*β*,17*β*-Trihydroxy-7*α*,17*α*-dimethylestr-4-en-3-one (4)

White crystalline; yield (percentage yield): 300 mg (30%); melting point 228‒229°C; UV λ_max_ (log ε): 230 (5.20); ${\left[\alpha \right]}_{D}^{25}$ = ‒ 22.9 (*c* 0.0041, CH_3_OH); IR (CHCl_3_) υ_max_; 3412 (O-H), 1664 (α, β-unsaturated ketone); EI-MS *m/z* (rel. int., %): 334.2 [M^+^] (100), 298.2 (24), 240.1 (11), 227.1 (64), 172.1 (19), 43.0 (16); HREI-MS *m/z*: 334.2159 [M^+^] (mol. formula C_20_H_30_O_4_, calc. for 334.2144); ^1^H-NMR (CD_3_OD, 400 MHz): Table 1; ^13^C-NMR (CD_3_OD, 125 MHz): Table 1. Single-crystal X-ray data: empirical formula C_20_H_32_O_5_; formula weight 352.46; crystal system orthorhombic, space group P2_1_2_1_2_1_, unit cell, **a** = 7.6451(10) Å, **b** = 10.734(2) Å, **c** = 23.051(3) Å, *α* = *β* = γ = 90°, Volume 1891.6(5) A^3^, Z = 4, calculated density 1.238 mg/m^3^, F(000) 768, crystal size 0.30 x 0.25 x 0.20 mm, *Ɵ* range for data collection 7.11 to 70.13°, reflections collected 15,121, unique reflections 2040 (R_int_ = 0.0240), goodness-of-fit on F^2^ 1.079, final R indices [I>2δ(I)] R_1_ = 0.0300, wR2 = 0.0799, R indices R1 = 0.0302, wR2 = 0.0802 for all data, largest diff. peak and hole 0.243 and -0.175 e.A^-3^.

### Isolation of metabolites of mibolerone (1) from the incubation of mibolerone (1) with *M*. *phaseolina*

Incubation of substrate **1** with *M*. *phaseolina* afforded 2.3 g crude extract. Silica gel column chromatography of the crude extract yielded four main fractions (4–7), which were further purified on reverse phase recycling HPLC. Metabolite **5** (*R*_*T*_ = 21 min, 6 mg) was obtained from fraction 4 through purification on reverse phase HPLC (MeOH: H_2_O; 70: 30). Metabolite **6** (*R*_*T*_ = 22 min, 7 mg) from fraction 5, compound **7** (*R*_*T*_ = 24 min, 6 mg) from fraction 6, and compound **8** (*R*_*T*_ = 23 min, 6 mg) from fraction 7 were obtained, on purification with reverse phase recycling HPLC (MeOH: H_2_O; 70: 30).

#### 11*β*,17*β*-Dihydroxy-(20-hydroxymethyl)-7*α*,17*α*-dimethylestr-4-en-3-one (5)

White solid; yield (percentage yield): 2.9 mg (0.4%); melting point 228 ‒ 230°C; UV λ_max_ (log ε): 248 (3.82); ${\left[\alpha \right]}_{D}^{25}$ = + 70.0 (*c* 0.0019, CH_3_OH); IR (CHCl_3_) υ_max_; 3412 (O-H), 1643 (*α*,*β*-unsaturated ketone); EI-MS *m/z* (rel. int., %): 334.2 [M^+^] (25), 317.2 (31), 286.1 (32), 285.1 (80), 267.1 (51), 242.1 (50), 229.1 (100) 133.1 (18), 55.0 (11); HREI-MS *m/z*: 334.2146 [M^+^] (mol. formula C_20_H_30_O_4_, calc. for 334.2144); ^1^H-NMR (CD_3_OD, 600 MHz): Table 2; ^13^C-NMR (CD_3_OD, 150 MHz): Table 2.

**Table 2:** ^13^C- and ^1^H-NMR chemical shift data (*J* in Hz) of compounds 5–8 (*δ* ppm).

Position	5		6		7		8	
	*δ*_C_	*δ*_H_ (*J* in Hz)	*δ*_C_	*δ*_H_ (*J* in Hz)	*δ*_C_	*δ*_H_ (*J* in Hz)	*δ*_C_	*δ*_H_ (*J* in Hz)
1	27.2	2.29, overlap; 2.34, overlap	66.3	4.40, d (*J* = 2.8)	66.3	4.38, t (*J* = 3)	27.3	1.57, m; 2.33, m
2	37.5	2.30, overlap; 2.35, overlap	47.1	2.41, overlap; 2.45, overlap	48.9	2.55, overlap; 2.59, overlap	37.5	2.34, m 2(H)
3	202.6	-	200.8	-	200.9	-	202.6	-
4	126.8	5.83, s	126.4	5.83, s	126.5	5.86, s	126.8	5.79, s
5	170.8	-	164.9	-	166.4	-	170.6	-
6	44.3	2.31, overlap; 2.54, dd (*J*_6/6_ = 13.5, *J*_6/7_ = 4.5)	44.3	2.37, d (*J* = 14.8); 2.53, overlap	44.2	2.52, dd (*J*_6/6_ = 14.4, *J*_6/7_ = 4.2)	44.3	2. 31, m; 2.53, dd (*J*_6/6_ = 13.6, *J*_6/7_ = 4.4)
7	32.1	2.18, m	31.4	1.95, m	31.4	2.02, m	32.1	2.07, m
8	38.9	1.96, m	36.4	1.68, overlap	38.9	1.96, m	39.3	1.94, m
9	49.2	1.23, m	44.2	1.69, overlap	41.2	1.81, overlap	48.3	1.24, m
10	39.1	2.65, overlap-	48.5	2.27, m	43.3	2.85, overlap;	39.0	2.64, m
11	67.4	4.19, d (*J*_11/9,12_ = 7.2)	27.0	1.32, m; 1.99, m	67.2	4.25, d (*J*_11/9,12_ = 2.4)	67.5	4.21, d (*J*_11/9,12_ = 3.2)
12	39.9	1.87, dd (*J*_12/12_ = 13.8, *J*_12/11_ = 2.4); 1.49, overlap	32.4	1.37, m; 1.54, m	39.8	1.49, m; 1.82, overlap	39.9	1.46, dd; (*J*_12/12_ = 14, *J*_12/11_ = 3.2); 1.82, dd (*J*_12/12_ = 14, *J*_12/11_ = 2.8)
13	46.4	-	46.9	-	47.1	-	46.5	-
14	48.2	1.48, m	47.6	1.31, overlap	46.8	1.58, m	48.4	1.38, overlap
15	23.6	1.35, m; 1.61, m	23.2	1.21, m; 1.45, m	23.3	1.41, m; 1.61, m	26.4	1.35, m; 1.61, m
16	33.3	1.67, m; 1.88, m	39.0	1.69, overlap; 1.89, overlap	48.7	1.45, m; 1.59, m	39.0	1.67, m; 1.88, m
17	84.5	-	82.2	-	82.5	-	82.4	-
18	17.4	1.15, s	14.4	0.93, s	17.0	1.13, s	17.1	1.12, s
19	13.3	0.79, d (*J*_19/7*β*_ = 7.2)	13.0	0.81, d (*J*_19/7*β*_ = 7.2)	12.9	0.82, d (*J*_19/7*β*_ = 7.2)	13.0	0.79, d (*J*_19/7*β*_ = 6.8)
20	67.7	3.42, d (*J*_20/20_ = 11.4); 3.57, d (*J*_20/20_ = 11.4)	26.1	1.21, s	26.4	1.18, s	23.3	1.18, s

c = ^1^H-NMR (600 MHz), and ^13^C-NMR (150 MHz)

d = ^1^H-NMR (400 MHz), and ^13^C-NMR (150 MHz)

e = ^1^H-NMR (400 MHz), and ^13^C-NMR (100 MHz)

#### 1*α*,17*β*-Dihydroxy-7*α*,17*α*-dimethylestr-4-en-3-one (6)

White solid; yield (percentage yield): 2.4 mg (0.24%); melting point 170‒174°; UV λ_max_ (log ε): 247 (2.99); ${\left[\alpha \right]}_{D}^{25}$ = ‒ 41.4 (*c* 0.0049, CH_3_OH); (CHCl_3_); υ_max_ 3433 (O-H), 1651 (*α*,*β*-unsaturated ketone); EI-MS *m/z* (rel. int., %): 318.9 [M^+^] (28), 301.9 (35), 300.9 (100), 299.9 (96), 261.9 (61), 228.8 (67), 226.9 (49) 173.9 (53), 135.0 (33); HREI-MS *m/z*: 318.2193 [M^+^] (mol. formula C_20_H_30_O_3_, calc. for 318.2195); ^1^H-NMR (CD_3_OD, 400 MHz): Table 2; ^13^C-NMR (CD_3_OD, 150 MHz): Table 2.

#### 1*α*,11*β*,17*β*-Trihydroxy-7*α*,17*α*-dimethylestr-4-en-3-one (7)

White solid; yield (percentage yield): 2.4 mg (0.34%); melting point 280 ‒ 282°C; UV λ_max_ (log ε): 247 (4.12); ${\left[\alpha \right]}_{D}^{25}$ = ‒ 94.2 (*c* 0.0019, CH_3_OH); IR (CHCl_3_) υ_max_; 3418 (O-H), 1661 (*α*,*β*-unsaturated ketone); EI-MS *m/z* (rel. int., %): 334.2 [M^+^] (2.6), 318.1 (41), 317.1 (90), 259.1 (68), 241.1 (49), 228.1 (100), 214.1 (81) 201.1 (47), 43.0 (36); HREI-MS *m/z* 334.2140 [M^+^] (mol. formula C_20_H_30_O_4_, calc. for 334.2144); ^1^H-NMR (CD_3_OD, 600 MHz): Table 2; ^13^C-NMR (CD_3_OD, 150 MHz): Table 2.

#### 11*β*,17*β*-Dihydroxy-7*α*,17*α*-dimethylestr-4-en-3-one (8)

White crystalline; yield (percentage yield): 21.1 mg (3%); melting point 249‒252°C; UV λ_max_ (log ε): 229 (3.88); ${\left[\alpha \right]}_{D}^{25}$ = + 104.3 (*c* 0.0021, IR CH_3_OH); (CHCl_3_) υ_max_; 3410 (O-H), 1651 (*α*,*β*-unsaturated ketone); EI-MS *m/z* (rel. int., %): 334.2 [M^+^] (2.6), 318.1 (41), 317.1 (90), 259.1 (68), 241.1 (49), 228.1 (100), 214.1 (81) 201.1 (47), 43.0 (36); HREI-MS *m/z*: 318.2211 [M^+^] (mol. formula C_20_H_30_O_3_, calcd. for 318.2195); ^1^H-NMR (CD_3_OD, 400 MHz): Table 2; ^13^C-NMR (CD_3_OD, 100 MHz): Table 2. Single-crystal X-ray data: empirical formula C_20_H_30_O_3_; formula weight 318.44; crystal system monoclinic, space group P2_1_, unit cell, **a** = 5.9750(11) Å, **b** = 11.550(3) Å, **c** = 12.642(2) Å, *β* = 99.728(14°, volume 859.9(3) A^3^, Z = 2, calculated density 1.230 mg/m^3^, F(000) 348, crystal size 0.32 x 0.28 x 0.18 mm, *Ɵ* range for data collection 7.52 to 72.00°, reflections collected 13,571, unique reflections 3,334 (R_int_ = 0.0236), goodness-of-fit on F^2^ 1.037, final R indices [I>2δ(I)] R1 = 0.0270, wR2 = 0.0734, R indices R1 = 0.0272, wR2 = 0.0736 for all data, largest diff. peak and hole 0.219 and -0.160 e. A^-3^.

### Assay protocol for *β*-glucuronidase inhibition

The inhibition of *β*-glucuronidase enzyme (E.C. 3.2.1.31, bovine liver) by test compounds was determined on a spectrophotometer by measuring the absorbance of *p*-nitrophenol at 405 nm, produced from the substrate. The reaction mixture comprised 185 μL of 0.1 M acetate buffer, and 5 μL of test compound solution, and 10 μL of (1U) enzyme solution in a 96-well plate. The mixture was incubated at 37°C for 30 min. The test compounds were solubilized in DMSO (100%), and 5 μL volume was added in each well (2% of total volume). Similar conditions were used for the standard (D-saccharic acid 1,4-lactone). The plates were read on a multiplate reader (SpectraMax plus 384) at 405 nm and 37°C, after addition of 50 μL of 0.4 mM *p*-nitrophenyl-*β*-D-glucuronide. All assays were performed in triplicate. IC_50_ values were calculated through EZ-Fit software (Perrella Scientific Inc., Amherst, MA, USA).

### Assay protocol for leishmanicidal activity

*Leishmania major* (DESTO, Pakistan) was cultured in a mixture of *NNN*-biphasic medium, and normal physiological saline solution. *L*. *major* promastigotes were grown on RPMI 1640 medium, supplemented with fetal bovine serum (FBS) (10% heat inactivated). Parasites were centrifuged (at log phase) for 10 min at 2,000 rpm and washed thrice with saline. The final density of fresh culture (106 cells/mL) was acquired through dilution of parasites. Sample solution of 1 mg of test compounds was prepared in a mixture 50 μL of DMSO and 950 μL of RPMI media. Fully dissolved compounds were transferred to 96-well plate. The first row of 96-well plate received 180 μL of the medium, while the remaining wells received 100 μL medium. Test compounds (20 μL each) were added into the medium containing wells, followed by serial dilution. The parasite culture (100 μL) was then transferred to each well. Negative controls comprised of only the growth medium, while standard leishmanicidal drugs (amphotericin or pentamidine) were used as positive controls. The plate was then incubated for 72 h at 21–22°C. The inhibitory effect of test compounds on the culture was analyzed microscopically on an improved Neubauer counting chamber. IC_50_ values were calculated through Ezfit 5.03 Perella Scientific software (USA).

### Assay protocol for cytotoxicity

MTT (3-(4,5-dimethyl thiazol-2yl)-2,5-diphenyl tetrazolium bromide) assay was employed to study the cytotoxic activity against HeLa (human cervical carcinoma, provided by Prof. Dr. Anwar Ali Siddiqui from Aga Khan University, Karachi, Pakistan), and mouse fibroblast 3T3 (ATCC^®^ CRL-1658, purchased from American Type Culture Collection, ATCC, Virginia, USA) cell lines. The cells (1 × 10^5^ /well) were plated in 0.2 mL of DMEM high glucose medium/well in 96-well plates. The cells were treated for 24 hours with test compounds in the range of 25, 50, 75, 100, and 200 μM concentrations, respectively. For the MTT assay, the medium from the wells was removed carefully after 24 hours treatment. Each well was washed with 1X PBS for 2–3 times, and 200 μL of MTT (5 mg/mL) was added in to media containing wells (1:10). The plates were incubated for 4 hours at 37°C, in a 5% CO_2_ incubator. After incubation, MTT was removed and 0.1 mL of DMSO was added to each well and mixed by keeping on a stirrer. The presence of viable cells was visualized by the development of purple color due to formation of the formazan crystals. The plates were observed under spectrophotometer (Spectramax) and absorbance was taken at 545 and 570 nm for cancer and normal cell lines, respectively. Measurements were performed and the concentration required for a 50% inhibition of viability (IC_50_) was determined graphically.

## Results and discussion

### Structure elucidation of compounds 1–8

**Fig 3:**
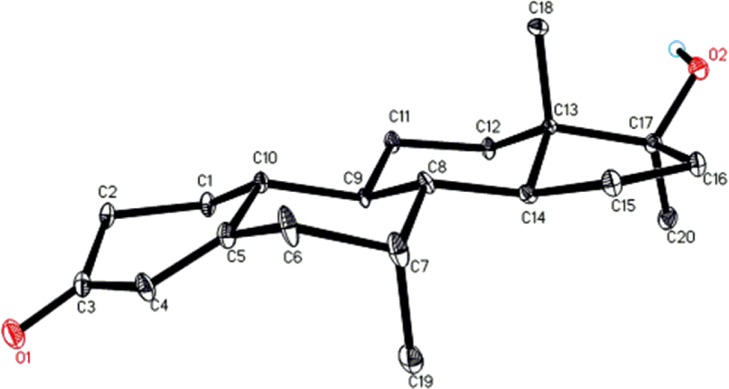
ORTEP drawing of X-ray structure of compound 1.

Compound **1** is a white solid with the [M^+^] at *m/z* 302.2. The identity of substrate **1** was confirmed by ^1^H-, ^13^C-NMR and 2D-NMR techniques (Table 1) [1–4]. Single-crystal X-ray diffraction analysis of compound **1** showed four fused rings, *i*.*e*., A (C1-C5/C10), B (C5-C10), C (C8-C9/C11-C14), and D (C13-C17). Ring A exists in a half chair conformation and contains *α*-*β* unsaturated carbonyl moiety, whereas trans-fused rings B, and C were in chair conformations. Five membered ring D attains an envelope conformation. A *β*-oriented hydroxyl group at C-17 is in a *pseudo* equatorial orientation (Fig 3). Single-crystal X-ray data of **1** was submitted to the Cambridge Crystallographic Data Centre (CCDC 1483437).

**Fig 4:**
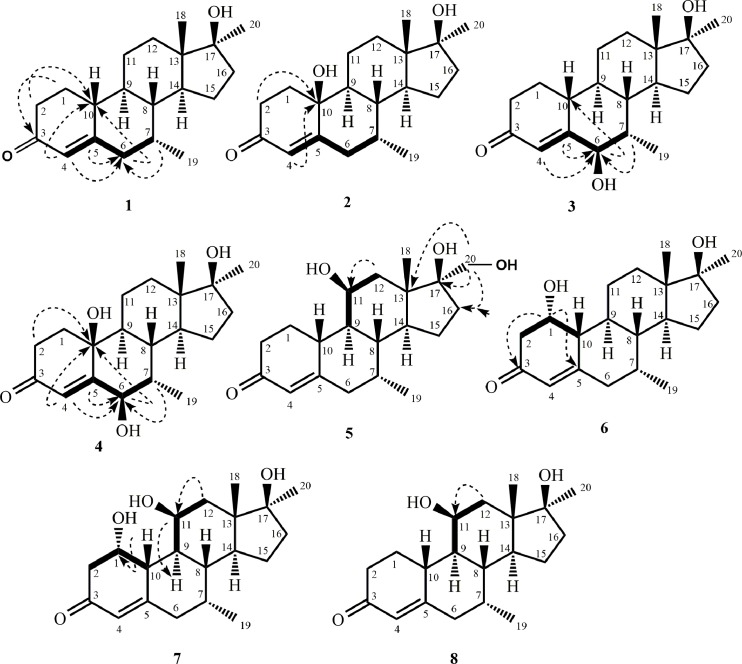
Key HMBC and COSY correlations of compounds 1–8.

**Fig 5:**
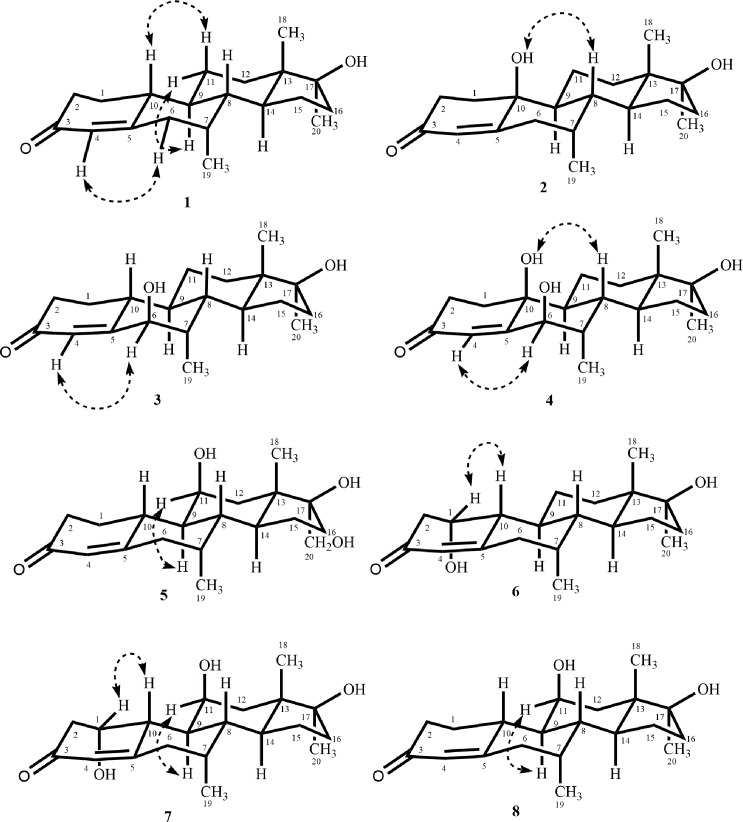
Key NOESY correlations of compounds 1–8.

**Fig 6:**
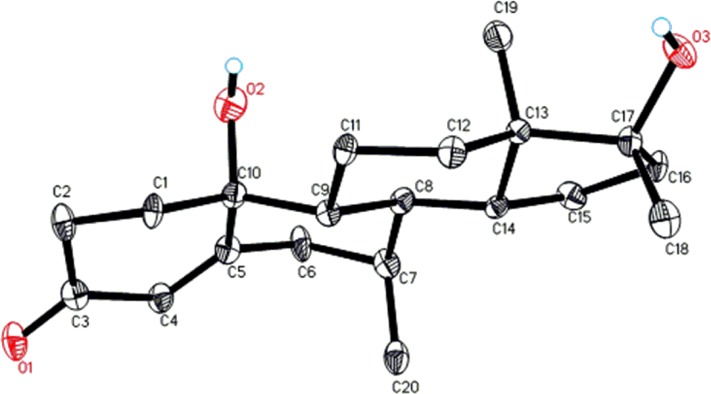
ORTEP drawing of X-ray structure of compound 2.

Metabolite **2** was isolated as white crystals. It displayed the [M^+^] in HREI-MS at *m/z* 318.2192 (C_20_H_30_O_3_, calcd. 318.2195), which suggested the addition of an oxygen in substrate **1** (*m/z* 302.45). The IR absorbances at 1654 and 3360 cm^-1^ were due to the presence of *α*,*β*-unsaturated ketone, and hydroxyl functionalities, respectively. The ^1^H-NMR spectrum was distinctly similar to substrate **1** (Table 1). An additional oxygenated quaternary carbon signal (*δ* 70.8) was observed in the ^13^C-NMR spectrum of compound **2**, which suggested the hydroxylation of substrate **1** (Table 1). This OH group was placed at C-10, based on the HMBC correlations of H-4 (*δ* 5.72, s) and H_2_-1 (*δ* 1.92, m; 2.22, m) with C-10 (*δ* 70.8) (Fig 4). The OH (*δ* 4.93) (DMSO-*d*_6_) at C-10 was deduced to be *β*-oriented based on its NOE correlation with the *β*-oriented H-8 (*δ* 1.99, m) (Fig 5). Single-crystal X-ray diffraction analysis was carried out to further deduce the structure of metabolite **2**. Structure was comprised of four fused rings *i*.*e*., A (C1-C5/C10), B (C5-C10), C (C8-C9/C11-C14), and D (C13-C17). Six-membered ring A containing *α*,*β-*unsaturated carbonyl moiety, exists in a half-chair conformation, whereas *trans*-fused rings B, and C were found to be in a chair conformation. Five membered ring D attains an envelope conformation. The two *β*-oriented hydroxyl groups at C-10 and C-17 are in *axial* and *pseudo* equatorial orientations, respectively (Fig 6). Single-crystal X-ray data of metabolite **2** was submitted to Cambridge Crystallographic Data Centre (CCDC 1483441). The structure of the new compound was thus deduced as 10*β*,17*β*-dihydroxy-7*α*,17*α*-dimethylestr4-en-3-one **2**.

Metabolite **3** was obtained as a white solid. It displayed the [M^+^] in HREI-MS at *m/z* 318.2196 (C_20_H_30_O_3_, calcd. 318.2195), which suggested the addition of an oxygen in substrate **1** (*m/z* 302.45). The presence of hydroxyl (3431 cm^-1^) and *α*,*β*-unsaturated ketone (1659 cm^-1^) were inferred from the IR spectrum. A downfield methine proton signal (*δ* 3.97, d, *J*_6/7_ = 3.0 Hz) in the ^1^H-NMR spectrum suggested hydroxylation in substrate **1** (Table 1). Similarly, the ^13^C-NMR spectrum of compound **3** showed a signal for a new downfield methine carbon signal (*δ* 78.7), suggesting an additional OH group (Table 1). The position of the OH group at C-6 was based on the HMBC correlations of H-4 (*δ* 5.86, d, *J*_4/6_ = 2.0 Hz) with the newly appearing methine carbon at *δ* 78.7 (C-6). The HMBC correlations of H-6 (*δ* 3.97, d) with C-5 (*δ* 167.3), C-10 (*δ* 39.8), and C-7 (*δ* 38.4) further supported an OH at C-6 (Fig 4). The key COSY correlations of allylic H-6 (*δ* 3.97, d) with H-4 (*δ* 5.86, d), and vicinal H-7 (*δ* 1.95, m) further confirmed the position of OH at C-6 (Fig 4). The OH at C-6 was deduced to be *β*-oriented on the basis of NOE correlations of the geminal H-6 (*δ* 3.97, d) with H-4 (*δ* 5.86, d) and H-19 (*δ* 0.74, d, *J*_19/7*β*_ = 7.5 Hz) (Fig 5). The structure of the compound was thus elucidated as 6*β*,17*β*-dihydroxy-7*α*,17*α*-dimethylestr-4-en-3-one **3**.

**Fig 7:**
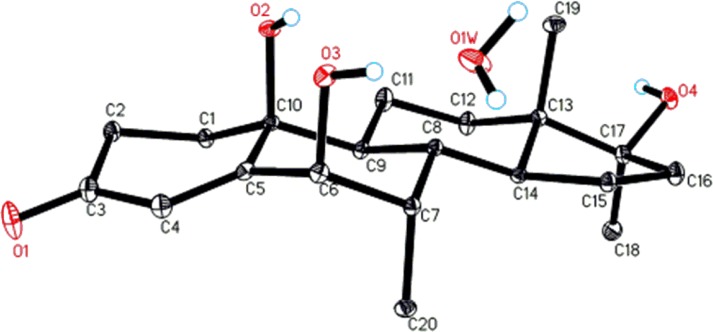
ORTEP drawing of X-ray structure of compound 4. Water appeared as solvent of crystallization.

Compound **4** was obtained as white crystals. The HREI-MS of metabolite **4** showed the [M^+^] in HREI-MS at *m/z* 334.2159 (C_20_H_30_O_4_, calcd. 334.2144), suggesting the addition of two oxygen atoms in substrate **1** (*m/z* 302.45). The absorbances for the hydroxyl (3412 cm^-1^) and *α*,*β*-unsaturated ketone groups (1664 cm^-1^) were spotted in the IR spectrum. A downfield methine proton signal (*δ* 4.08, s) in the ^1^H-NMR spectrum further indicated hydroxylation in substrate **1** (Table 1). The ^13^C-NMR spectrum of compound **4** showed signals for a new downfield methine carbon at *δ* 80.1, as well as another downfield quaternary carbon at *δ* 72.4, suggesting dihydroxylation of substrate **1** (Table 1). One of the two OH groups was placed at C-6, based on the HMBC correlations of H-4 (*δ* 5.83, s) with the newly appearing methine carbon at *δ* 80.1 (C-6), whereas the second OH group was placed at C-10 on the basis of HMBC correlations of H-4 (*δ* 5.83, s) and H-2 (*δ* 2.26, m; 2.63, m) with the downfield quaternary carbon at *δ* 72.4 (C-10). The HMBC correlations of H-6 (*δ* 4.08, s) with C-5 (*δ* 160.6), C-10 (*δ* 72.4), and C-7 (*δ* 38.6) further supported the positions of OH group at C-6, and C-10 (Fig 4). This was also indicated in the COSY spectrum, which showed correlations of H-6 (*δ* 4.08, s) with H-7 (*δδ* 2.01, m) (Fig 4). The OH at C-6 was deduced to be *β*-oriented on the basis of NOESY correlations of H-6 (*δ* 4.08, s) with H-4 (*δ* 5.83, s) and H-19 (*δ* 0.75, d, *J*_19/7*β*_ = 7.2 Hz). The OH (*δ* 4.29) (acetone-*d*_6_) at C-10 was deduced to be *β*-oriented based on its NOE correlations with *β*-oriented OH-6 (*δ* 5.26), and *β*-oriented H-8 (*δ* 2.30, m) (Fig 5). Single-crystal X-ray diffraction analysis was carried out to further establish the structure for metabolite **4**. The analysis revealed that metabolite **4** was crystalized out as a water solvate, and comprised three six-membered rings A (C1-C5/C10), and B (C5-C10), C (C8-C9/ C11-C14), existing in half chair and chair conformations, respectively, while a five-membered ring D (C13-C17) exists in an envelope conformation (Fig 7). Single-crystal X-ray data of metabolite **4** was submitted to the Cambridge Crystallographic Data Centre (CCDC 1483442). The structure of the compound was thus deduced as 6*β*,10*β*,17*β*-trihydroxy-7*α*,17*α*-dimethylestr-4-en-3-one **4**.

Compound **5** was obtained as a white solid. HREI-MS of metabolite **5** showed the [M^+^] at *m/z* 334.2146 (C_20_H_30_O_4_, calcd. 334.2144). Increase in mass of 32 amu suggested dihydroxylation in substrate **1** (*m/z* 302.45). The IR absorbances at 3412 and 1643 cm^-1^ indicated the presence of OH and *α*,*β*-unsaturated ketone functionalities, respectively. The ^1^H-NMR spectrum showed signals for a new downfield methine (*δ* 4.19, d, *J*_11/9,12_ = 7.2 Hz) and methylene protons (*δ* 3.42, d, *J*_20/20_ = 11.4 Hz; 3.57, d, *J*_20/20_ = 11.4 Hz), which suggested dihydroxylation in substrate **1** (Table 2). The ^13^C-NMR spectrum displayed additional signals of a new methine (*δ* 67.4), and methylene carbons (*δ* 67.7), confirming dihydroxylation in substrate **1** (Table 2). One of the two OH groups was deduced to be at C-11 on the basis of HMBC correlations of H_2_-12 (*δ* 1.49, overlap; 1.87, dd, *J*_12/12_ = 13.8 Hz, *J*_12/11_ = 2.4 Hz) with the newly formed methine carbon at *δ* 67.4 (C-11), whereas second OH group was placed at C-20 based on the HMBC correlations of H_2_-20 (*δ* 3.42, d; 3.57, d) with C-13 (*δ* 46.4), C-16 (*δ* 33.3), and C-17 (*δ* 84.5) (Fig 4). The positions of the OH at C-11 and at C-20 were further established by the COSY correlations of H-11 (*δ* 4.19, d, *J*_11/9,12_ = 7.2 Hz) with H-9 (*δ* 1.23, m), and H_2_-12 (*δ* 1.49; overlap 1.87, dd), and H_2_-20 (*δ* 3.42, d; 3.57, d) with OH-17 (*δ* 4.32) (DMSO-*d*_6_) (Fig 4). H-11 (*δ* 4.19, d) showed NOE correlations with *α*-oriented H-9 (*δ* 1.23, m). Therefore, OH at C-11 was deduced to be *β*-oriented (Fig 5). The structure of the new compound was thus deduced as 11*β*,17*β*-dihydroxy-(20-hydroxymethyl)-7*α*,17*α*-dimethylestr-4-en-3-one **5**.

Metabolite **6** was obtained as a white solid. Its HREI-MS displayed the [M^+^] at *m/z* 318.2193 (C_20_H_30_O_4_, calcd. 318.2195) which suggested monohydroxylation of substrate **1** 302.45). The IR absorbances at 3433 and 1651 cm^-1^ were due to OH and *α*, *β*-unsaturated ketone moieties, respectively. The ^1^H-NMR spectrum showed signals for a new downfield methine proton (*δ* 4.40, d, *J*_1/2,10_ = 2.8 Hz), indicating addition of an OH group in substrate **1** (Table 2). A new methine carbon (*δ* 66.3) in the ^13^C-NMR spectrum supported the above inference (Table 2). In the HMBC spectrum, correlations of the methine proton (*δ* 4.40, d) observed with C-3 (*δ* 200.8), and C-5 (*δ* 164.9) (Fig 4). The COSY correlations of H-1 (*δ* 4.40, d) with H-10 (*δ* 2.27, m), and H_2_-2 (*δ* 2.41, overlap; 2.45, overlap) further confirmed the structure (Fig 4). Geminal H-1 (*δ* 4.40, d) showed NOE correlation with *β*-oriented H-10 (*δ* 2.27, m). Therefore, OH at C-1 was deduced to be *α*-oriented (Fig 5). Thus, the structure of the compound was deduced as 1*α*,17*β*-dihydroxy-7*α*,17*α*-dimethylestr-4-en-3-one **6**.

Compound **7** was obtained as a white solid. The HREI-MS of compound **7** showed the [M^+^] at *m/z* 334.2140 (C_20_H_30_O_4_, calcd. 334.2144). This indicated that dihydroxylation had occurred on substrate **1** (*m/z* 302.45). The IR absorbances at 3418 and 1661 cm^-1^ indicated the presence of OH and *α*,*β*-unsaturated ketone groups, respectively. The ^1^H-NMR spectrum showed signals for two new downfield methine protons at *δ* 4.25, d, and 4.38, t, suggesting dihydroxylation in substrate **1** (Table 2). Two new oxy-methine carbon signals (*δ* 66.3, 67.2) in ^13^C-NMR spectrum further supported dihydroxylation (Table 2). The OH was placed at C-1 on the basis of HMBC correlations between H-10 (*δ* 2.85, overlap) and C-1 (*δ* 66.3), and the COSY correlations of H-1 (*δ* 4.38, t, *J*_1/2,10_ = 3.0 Hz) with H-10 (*δ* 2.85, overlap), and H_2_-2 (*δ* 2.55, overlap; 2.59, overlap). The second OH was placed at C-11 on the basis of HMBC correlations of H-9 (*δ* 1.81, overlap) and H_2_-12 (*δ* 1.49, m; 1.82, overlap) with C-11 (*δ* 67.2), and the COSY correlations of H-11 (*δ* 4.25, d, *J*_*11/9*,*12*_ = 2.4 Hz) with H-9 (*δ* 1.81, overlap), and H_2_-12 (*δ* 1.49, m; 1.82, overlap) (Fig 4). H-1 (*δ* 4.38) showed NOE correlations with the *β*-oriented H-10 (*δ* 2.85, overlap), while H-11 (*δ* 4.25, d) showed NOE correlations with *α*-oriented H-9 (*δ* 1.81, overlap). Therefore, OH at C-1 as *α*-oriented, while OH at C-11 was deduced to be *β*-oriented (Fig 5). The new compound was thus characterized as 1*α*,11*β*,17*β*-trihydroxy-7*α*,17*α*-dimethylestr-4-en-3-one **7**.

**Fig 8:**
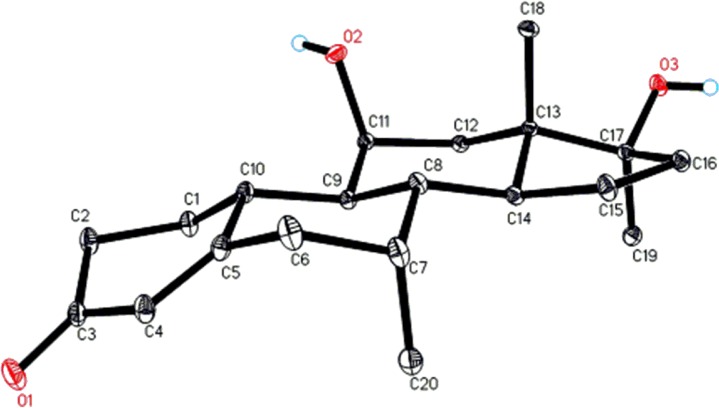
ORTEP drawing of X-ray structure of compound 8.

Metabolite **8** was obtained as a white crystalline material. The HREI-MS of compound **8** showed the [M^+^] at *m/z* 318.2211 (C_20_H_30_O_3_, calcd. 318.2195), indicating monohydroxylation in substrate **1** (*m/z* 302.45). Single-crystal diffraction showed that compound **8** has the same structural features those of metabolites **2** and **4**, and consists of three six-membered rings A, B, and C, possessing half chair, chair, and chair conformations, respectively. The five-membered ring D possesses an envelope conformation. Two OH substituents were attached at C-11, and C-17 in *axial*, and *pseudo* equatorial orientations, respectively. All bond angles and lengths were within the normal range, and found to similar to metabolites **2**, and **4** (Fig 8). The single-crystal X-ray data of metabolite **8** was submitted to the Cambridge Crystallographic Data Centre (CCDC 1483443). The compound was thus identified as 11*β*,17*β*-dihydroxy-7*α*,17*α*-dimethylestr-4-en-3-one **8**, which has been previously synthesized through biotransformation [32].

#### β-Glucuronidase inhibitory activity

**Fig 9:**
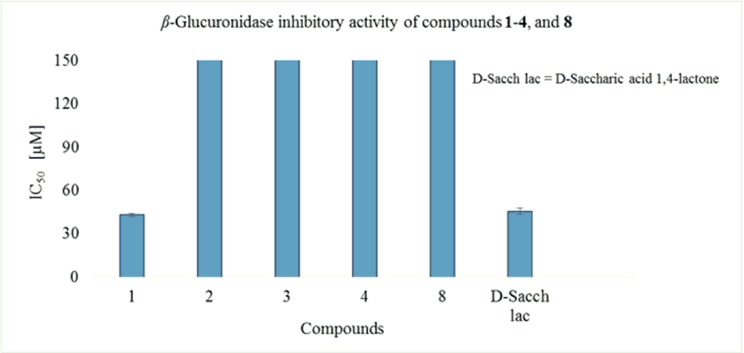
*β*-Glucuronidase activity of compounds were shown in graphical representation.

Mibolerone (**1**) and its transformed products **2**–**4**, and **8** were evaluated for their *β*-glucuronidase inhibitory activity. Compound **1** was identified as a potent inhibitor of *β*-glucuronidase with IC_50_ = 42.98 ± 1.24 μM, as compared to the standard drug, D-saccharic acid 1,4-lactone (IC_50_ = 45.75 ± 2.16 μM) (Fig 9). Interestingly, its metabolites were found to be inactive (Table 3).

**Table 3:** *In vitro β*-glucuronidase inhibitory activity of mibolerone (1), and its metabolites.

Compounds	IC_50_ ± SD [μM]
**1**	42.98 ± 1.24
**2**	Inactive
**3**	Inactive
**4**	Inactive
**8**	Inactive
Standard drug D-Saccharic acid 1,4-lactone	45.75 ± 2.16

#### Leishmanicidal activity

**Fig 10:**
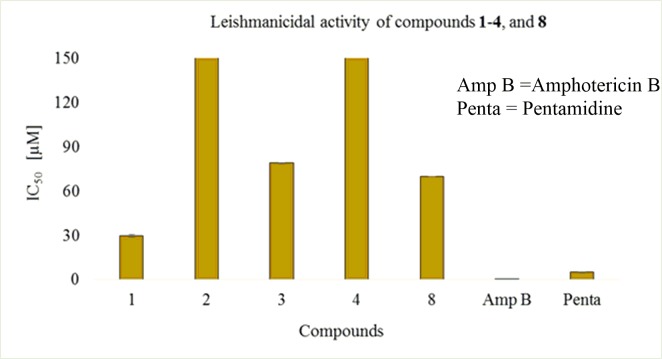
Leishmanicidal activity of compounds were shown in graphical representation.

Mibolerone (**1**) and its metabolites **2**–**4**, and **8** were assessed for leishmanicidal activity. Substrate **1** showed a significant leishmanicidal activity against the promastigotes of *Leishmania major* with IC_50_ value of 29.64 ± 0.88 μM as compared to the standard drugs, pentamidine (IC_50_ = 5.09 ± 0.09 μM), and amphotericin B (IC_50_ = 0.29 ± 0.05 μM) (Fig 10). However, its derivatives **3** (IC_50_ = 79.09 ± 0.06 μM) and **8** (IC_50_ = 70.09 ± 0.05 μM) showed only weak activity against the promastigotes of *Leishmania major*, while compounds **4** and **5** were found to be inactive (Table 4).

**Table 4:** Leishmanicidal activity of mibolerone (1), and its metabolites.

Compounds	IC_50_ ± SD [μM]
**1**	29.64 ± 0.88
**2**	Inactive
**3**	79.09 ± 0.06
**4**	Inactive
**8**	70.09 ± 0.05
Amphotericin B (Standard drug)	0.29 ± 0.05
Pentamidine (Standard drug)	5.09 ± 0.09

#### Cytotoxic activity

**Fig 11:**
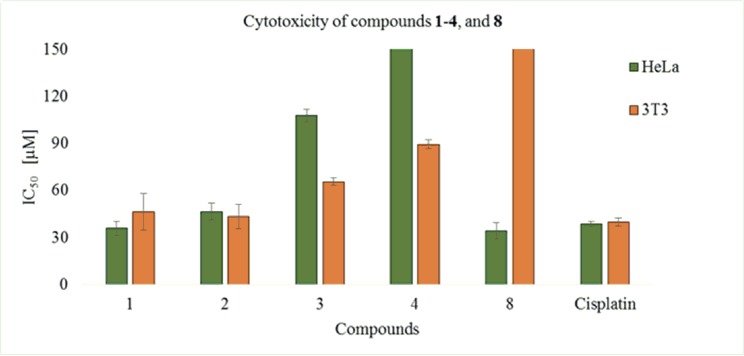
Cytotoxicity of compounds were shown in graphical representation.

Mibolerone (**1**) and its metabolites **2**–**4**, and **8** were evaluated for their cytotoxic activity against HeLa cancer and 3T3 cell lines. Compounds **1**, and **8** were found to be strongly active against HeLa cancer cell line (human epithelial carcinoma) with IC_50_ values of 35.7 ± 4.46, and 34.1 ± 5.3 μM, respectively, as compared to the standard drug, cisplatin (IC_50_ = 38.5 ± 1.8 μM) (Fig 11). Compound **2** also showed a significant activity with IC_50_ = 46.5 ± 5.4 μM, while **3**, and **4** showed a weak activity with IC_50_ = 107.8 ± 4.0, and IC_50_ = 152.5 ± 2.1 μM, respectively. Compounds **1**–**4** also showed cytotoxicity against 3T3 (mouse fibroblast) cell line with IC_50_ values of 46.3 ± 11.7, 43.3 ± 7.7, 65.6 ± 2.5, and 89.4 ± 2.7 μM, while compound **8** showed no cytotoxicity against 3T3 cell line (Table 5).

**Table 5:** Cytotoxicity of mibolerone (1), and its metabolites.

Compounds	HeLa Cell line IC_50_ ± SD [μM]	3T3 Cell line IC_50_ ± SD [μM]
**1**	35.7 ± 4.46	46.3 ± 11.7
**2**	46.5 ± 5.4	43.3 ± 7.7
**3**	107.8 ± 4.0	65.6 ± 2.5
**4**	152.5 ± 2.1	89.4 ± 2.7
**8**	34.1 ± 5.3	Inactive
Standard drug Cisplatin	38.5 ± 1.8	39.7 ± 2.7

## Conclusion

In conclusion, microbial transformations of mibolerone (**1**) with *C*. *blakesleeana*, *C*. *echinulata*, and *M*. *phaseolina* yielded six new metabolites **2**–**7**, along with a known metabolite **8**. During these transformations, hydroxylations at C-1, C-6, C-10, C-11, and C-20 were observed. C-6, C-10, and C-11 were the sites for *β*-hydroxylation, whereas *α*-hydroxylation occurred at C-1. Substrate **1** was found to be significantly active against *β*-glucuronidase enzyme, leishmaniasis, and HeLa cancer, and 3T3 normal cell lines *in vitro*. Metabolites **2**, and **8** were found to be potently active against HeLa cancer cell line, while metabolites **3**, and **4** were weakly active. Metabolites **2**–**4** were toxic to 3T3 cell line, whereas metabolite **8** showed no cytotoxicity against 3T3 cell line. In addition, metabolites **3** and **8** also showed weak leishmanicidal activity *in vitro* against the promastigotes of *Leishmania major*. The presented study indicated that compound **8** deserves to be further studied for its therapeutic potential.

## Supporting information

S1 Data Spectral data of compounds 2. (PDF)

S2 Data Spectral data of compounds 3. (PDF)

S3 Data Spectral data of compounds 4. (PDF)

S4 Data Spectral data of compounds 5. (PDF)

S5 Data Spectral data of compounds 6. (PDF)

S6 Data Spectral data of compounds 7. (PDF)

S7 Data Spectral data of compounds 8. (PDF)
