# Supplementary material for: Biotransformation of a potent anabolic steroid, mibolerone, with Cunninghamella blakesleeana, C. echinulata, and Macrophomina phaseolina, and biological activity evaluation of its metabolites
Source: PLoS One. 2017 Feb 24;12(2):e0171476. doi: 10.1371/journal.pone.0171476 (PMC5325191; doi:10.1371/journal.pone.0171476)
Supplement: S1 Data — (PDF) [file pone.0171476.s001.pdf]

File: EC-M-2
Sample: MAHWISH /DR. IQBAL
Instrument: JEOL MS 600H-1

Date Run: 07-01-2015 (Time Run: 09:19:15)

Ionization mode: EI+

compound 2

Scan: 12
Base: m/z 44; 32%FS TIC: 7057680

R.T.: .98

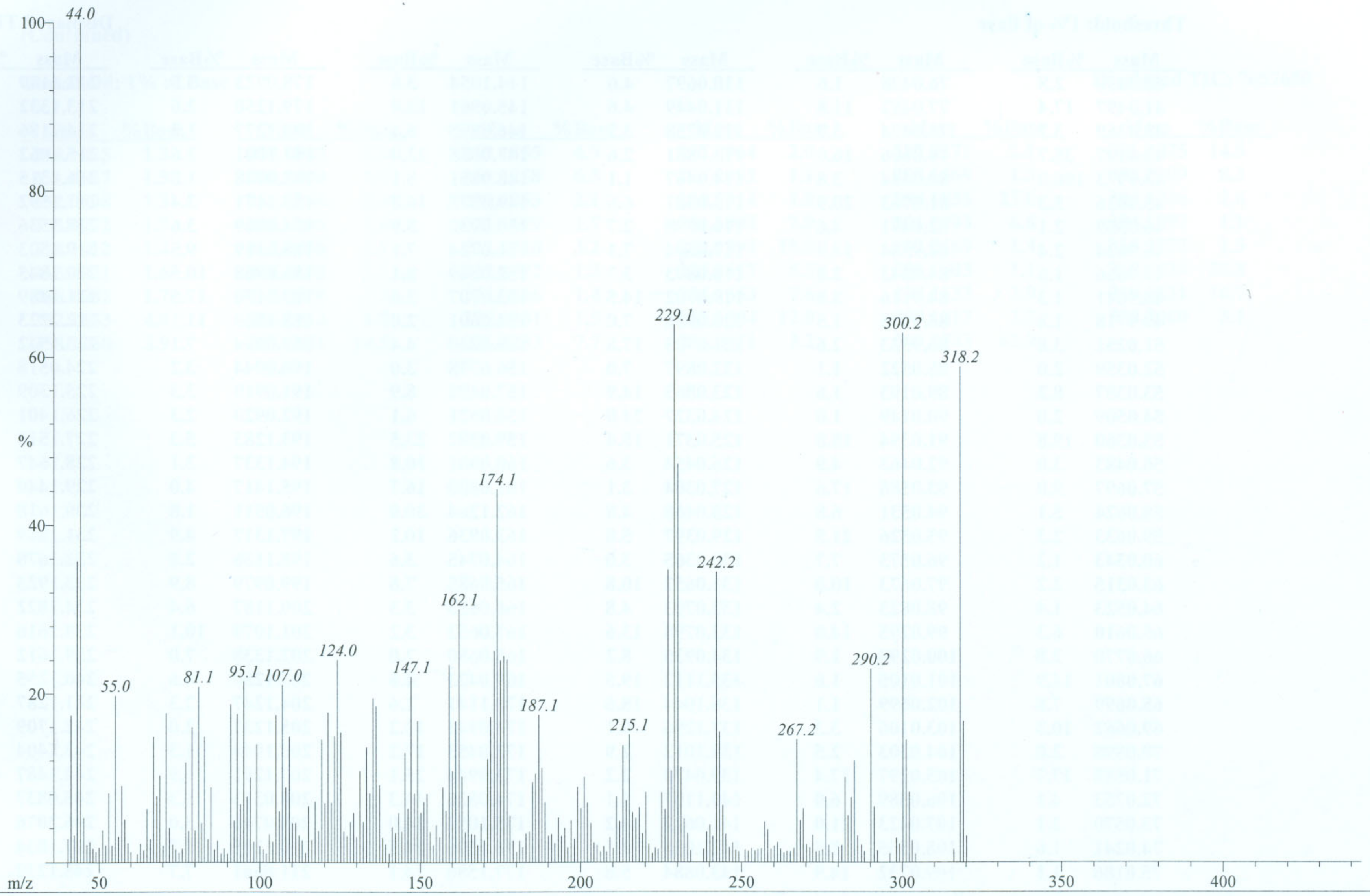

Compound 2

Mass	Relative Intensity	Theoretical Mass	Delta [ppm]	Delta [mmu]	RDB	Composition
254.1675	1.1	254.1671	1.6	0.4	8.0	$C_{18} H_{22} O_1$
255.1735	1.0	255.1749	-5.6	-1.4	7.5	$C_{18} H_{23} O_1$
256.1852	1.0	256.1827	9.8	2.5	7.0	$C_{18} H_{24} O_1$
257.1930	6.6	257.1905	9.6	2.5	6.5	$C_{18} H_{25} O_1$
258.1653	3.0	258.1620	12.9	3.3	7.0	$C_{17} H_{22} O_2$
258.2003	3.5	258.1984	7.4	1.9	6.0	$C_{18} H_{26} O_1$
260.1786	1.4	260.1776	3.8	1.0	6.0	$C_{17} H_{24} O_2$
261.1838	2.3	261.1855	-6.5	-1.7	5.5	$C_{17} H_{25} O_2$
263.1448	1.1	263.1436	4.4	1.2	10.5	$C_{19} H_{19} O_1$
265.1569	1.5	265.1592	-8.7	-2.3	9.5	$C_{19} H_{21} O_1$
267.1759	22.4	267.1749	3.9	1.0	8.5	$C_{19} H_{23} O_1$
268.1790	4.7	268.1827	-13.8	-3.7	8.0	$C_{19} H_{24} O_1$
269.1890	2.0	269.1905	-5.9	-1.6	7.5	$C_{19} H_{25} O_1$
272.2148	2.8	272.2140	2.9	0.8	6.0	$C_{19} H_{28} O_1$
276.2093	8.8	276.2089	1.3	0.4	5.0	$C_{18} H_{28} O_2$
277.2150	2.1	277.2168	-6.3	-1.8	4.5	$C_{18} H_{29} O_2$
280.1822	2.6	280.1827	-2.0	-0.6	9.0	$C_{20} H_{24} O_1$
281.1913	1.1	281.1905	2.8	0.8	8.5	$C_{20} H_{25} O_1$
282.1982	16.3	282.1984	-0.6	-0.2	8.0	$C_{20} H_{26} O_1$
283.2020	4.9	283.2062	-14.9	-4.2	7.5	$C_{20} H_{27} O_1$
284.2126	2.8	284.2140	-4.8	-1.4	7.0	$C_{20} H_{28} O_1$
285.1860	16.3	285.1855	1.8	0.5	7.5	$C_{19} H_{25} O_2$
286.1876	3.5	286.1933	-19.7	-5.7	7.0	$C_{19} H_{26} O_2$
290.2224	36.2	290.2246	-7.4	-2.1	5.0	$C_{19} H_{30} O_2$
291.2285	8.7	291.2324	-13.4	-3.9	4.5	$C_{19} H_{31} O_2$
292.2271	1.1					
298.1933	3.6	298.1933	0.0	0.0	8.0	$C_{20} H_{26} O_2$
299.2007	1.5	299.2011	-1.2	-0.4	7.5	$C_{20} H_{27} O_2$
300.2081	89.5	300.2089	-2.8	-0.8	7.0	$C_{20} H_{28} O_2$
301.2141	21.5	301.2168	-8.7	-2.6	6.5	$C_{20} H_{29} O_2$
302.2220	5.0	302.2246	-8.6	-2.6	6.0	$C_{20} H_{30} O_2$
303.2256	1.3					
317.2427	1.2	317.2481	-16.9	-5.4	5.5	$C_{21} H_{33} O_2$
318.2192	77.5	318.2195	-0.9	-0.3	6.0	$C_{20} H_{30} O_3$
319.2239	18.2	319.2273	-10.6	-3.4	5.5	$C_{20} H_{31} O_3$
320.2260	2.6					

Comp 2

MAHWISH/DR.IQBAL/EC.M.2
1H/

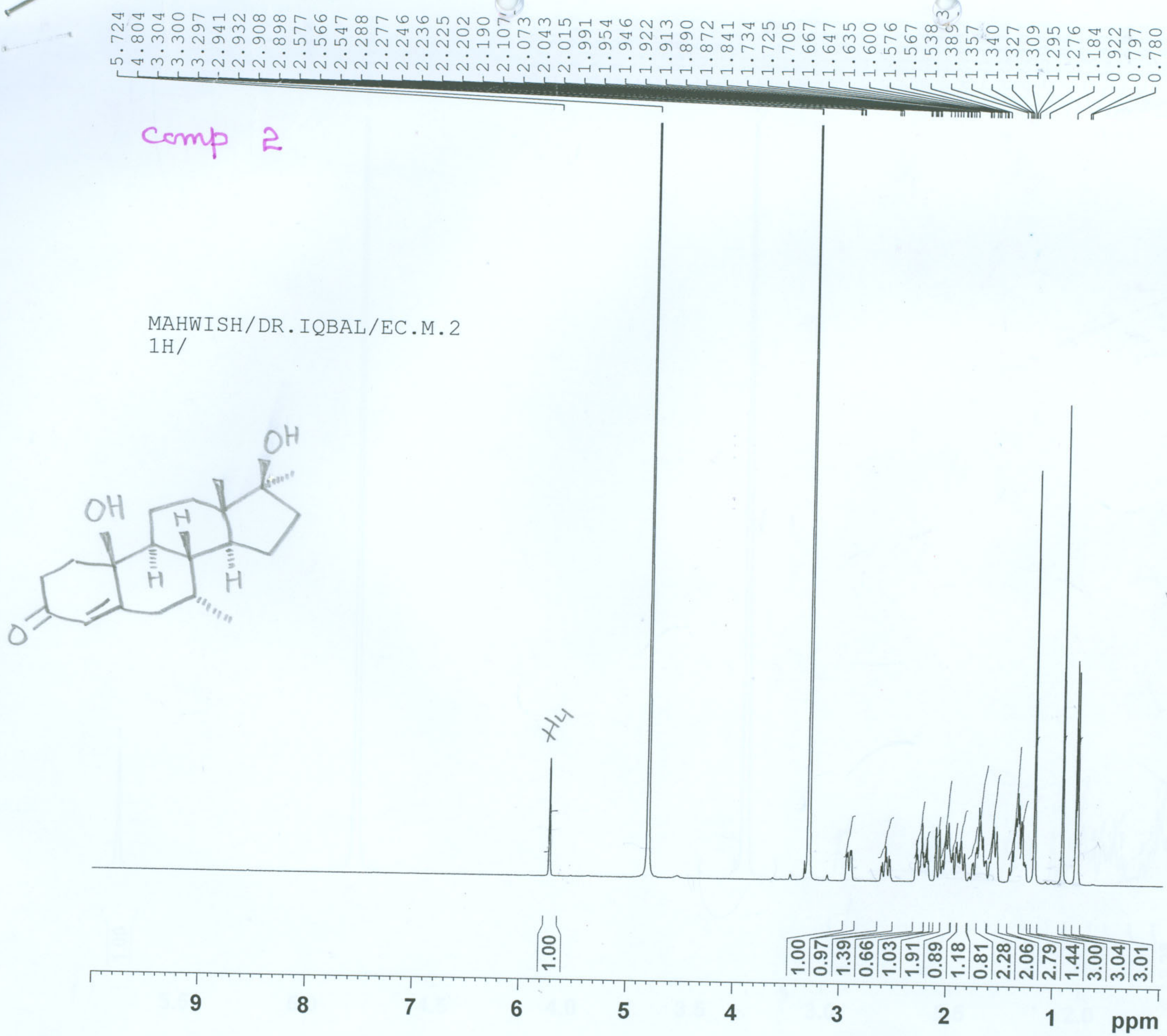

AVANCE AV-400 MHz
Lab # 115

NAME	jun25-15
EXPNO	1
PROCNO	1
Date_	20150625
Time	10.43
INSTRUM	spect
PROBHD	5 mm SEI 1H-13
PULPROG	zg30
TD	32768
SOLVENT	MeOD
NS	128
DS	0
SWH	8012.820 Hz
FIDRES	0.244532 Hz
AQ	2.0447731 sec
RG	512
DW	62.400 usec
DE	6.50 usec
TE	300.0 K
D1	2.00000000 sec
TD0	1

======== CHANNEL f1 ========

NUC1	1H
P1	10.80 usec
PL1	3.00 dB
SFO1	400.0332002 MHz
SI	16384
SF	400.0300087 MHz
WDW	EM
SSB	0
LB	0.30 Hz
GB	0
PC	1.00

MAHWISH/DR.IQBAL/EC-M-2/CD3OD
ICCBS/U.O.K
B.B

compound 2

AVANCE AV-500
LAB NO: 109B

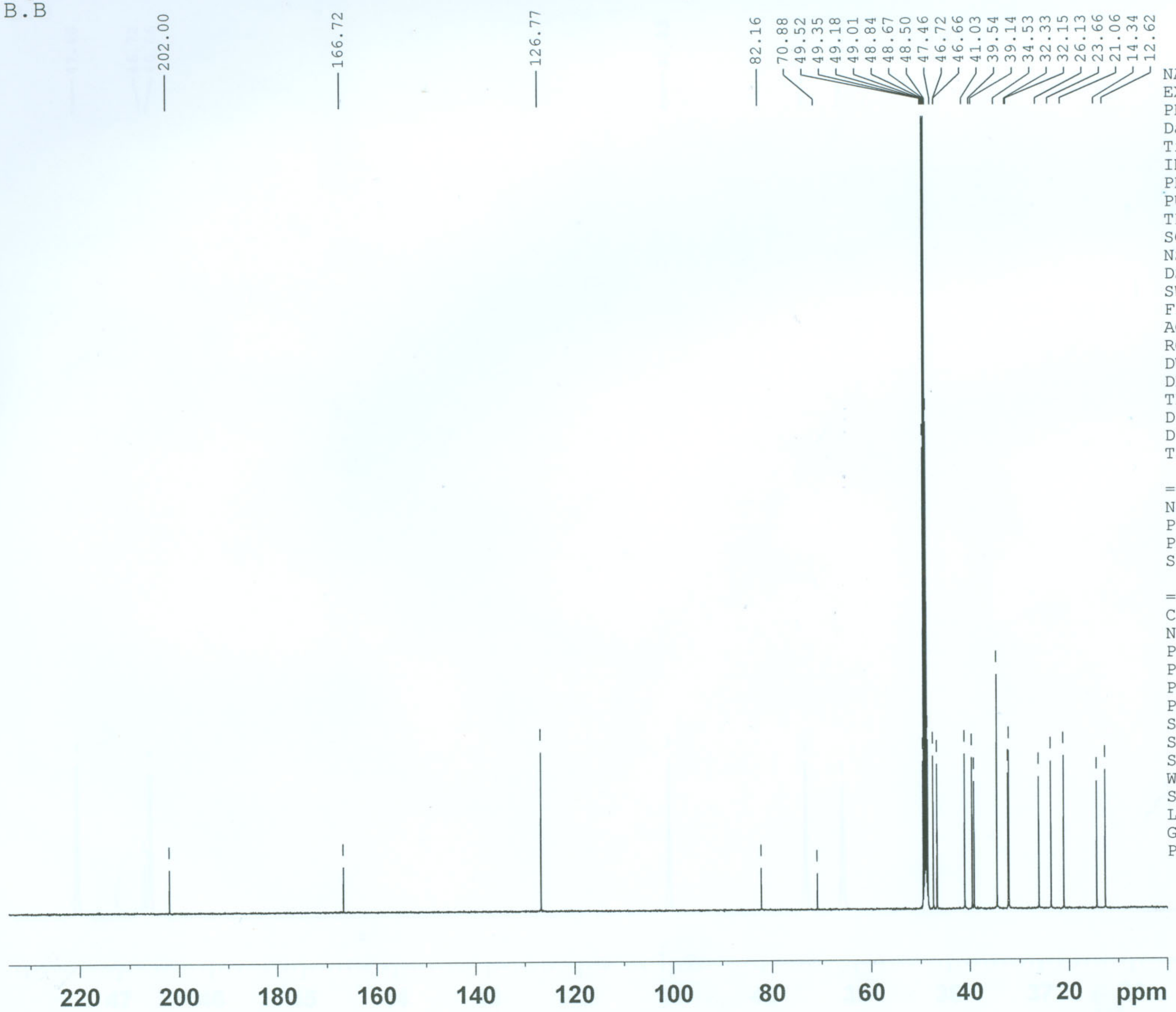

NAME	jun16-15
EXPNO	1
PROCNO	1
Date_	20150616
Time	14.21
INSTRUM	spect
PROBHD	5 mm BBI 1H/D-
PULPROG	zgpg
TD	32768
SOLVENT	MeOD
NS	18432
DS	4
SWH	29498.525 Hz
FIDRES	0.900224 Hz
AQ	0.5554845 sec
RG	32768
DW	16.950 usec
DE	6.50 usec
TE	300.9 K
D1	2.00000000 sec
D11	0.03000000 sec
TD0	18
======== CHANNEL f1 ========	
NUC1	13C
P1	12.00 usec
PL1	-3.00 dB
SFO1	125.7723529 MHz
======== CHANNEL f2 ========	
CPDPRG2	waltz16
NUC2	1H
PCPD2	80.00 usec
PL2	-1.00 dB
PL12	19.20 dB
PL13	22.00 dB
SFO2	500.1325007 MHz
SI	32768
SF	125.7576112 MHz
WDW	EM
SSB	0
LB	1.00 Hz
GB	0
PC	1.00

MAHWISH/DR.IQBAL/EC-M-2/CD3OD
ICCBS/U.O.K
DEPT135

comp 2

AVANCE AV-500
LAB NO: 109B

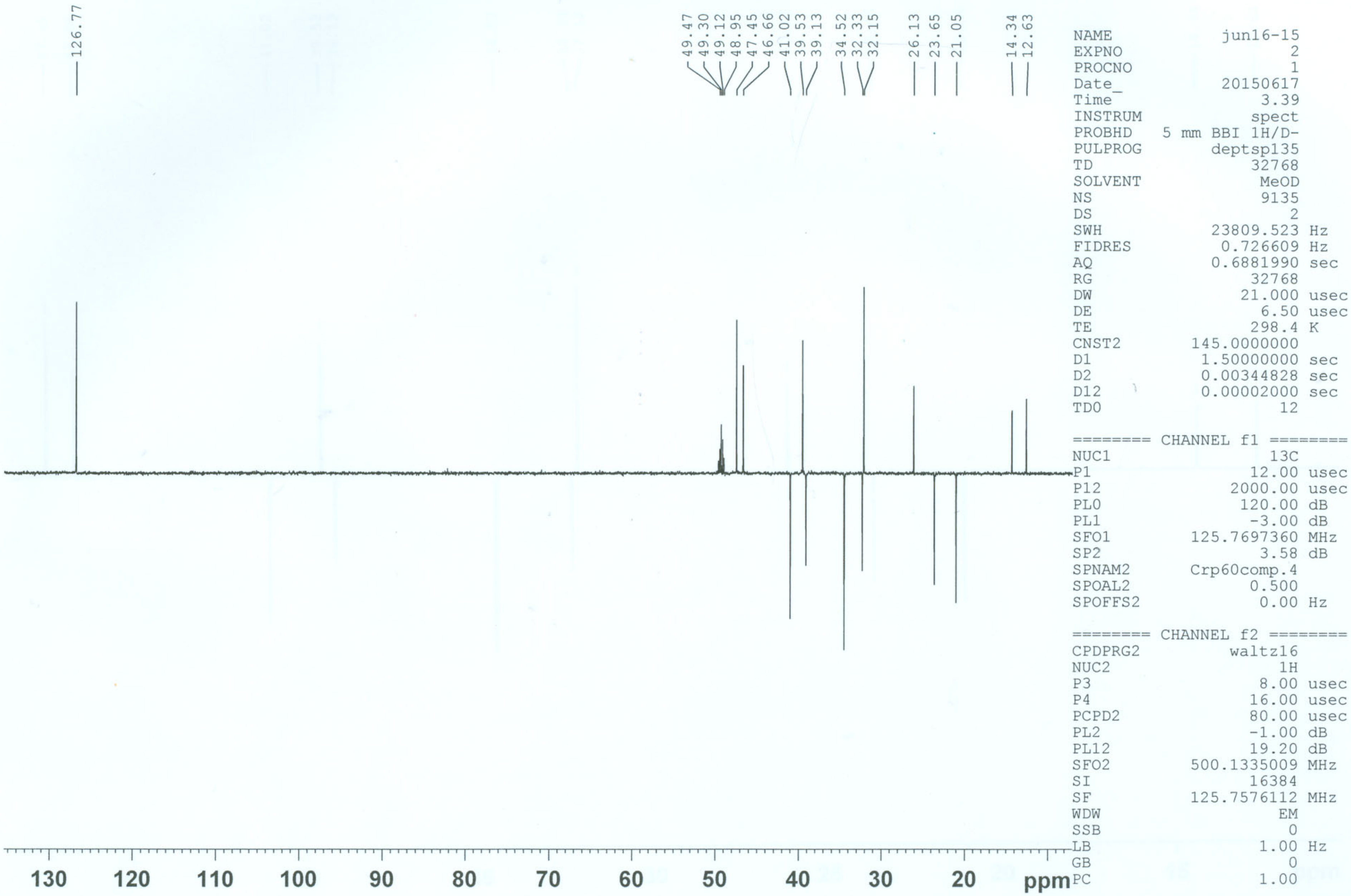

NAME	jun16-15	
EXPNO	2	
PROCNO	1	
Date_	20150617	
Time	3.39	
INSTRUM	spect	
PROBHD	5 mm BBI 1H/D-	
PULPROG	deptsp135	
TD	32768	
SOLVENT	MeOD	
NS	9135	
DS	2	
SWH	23809.523	Hz
FIDRES	0.726609	Hz
AQ	0.6881990	sec
RG	32768	
DW	21.000	usec
DE	6.50	usec
TE	298.4	K
CNST2	145.0000000	
D1	1.50000000	sec
D2	0.00344828	sec
D12	0.00002000	sec
TD0	12	
======== CHANNEL f1 ========		
NUC1	13C	
P1	12.00	usec
P12	2000.00	usec
PL0	120.00	dB
PL1	-3.00	dB
SFO1	125.7697360	MHz
SP2	3.58	dB
SPNAM2	Crp60comp.4	
SPOAL2	0.500	
SPOFFS2	0.00	Hz
======== CHANNEL f2 ========		
CPDPRG2	waltz16	
NUC2	1H	
P3	8.00	usec
P4	16.00	usec
PCPD2	80.00	usec
PL2	-1.00	dB
PL12	19.20	dB
SFO2	500.1335009	MHz
SI	16384	
SF	125.7576112	MHz
WDW	EM	
SSB	0	
LB	1.00	Hz
GB	0	
PC	1.00	

MAHWISH/DR.IQBAL/EC-M-2/CD3OD
ICCBS/U.O.K
DEPT90

AVANCE AV-500
LAB NO: 109B

Compound 2

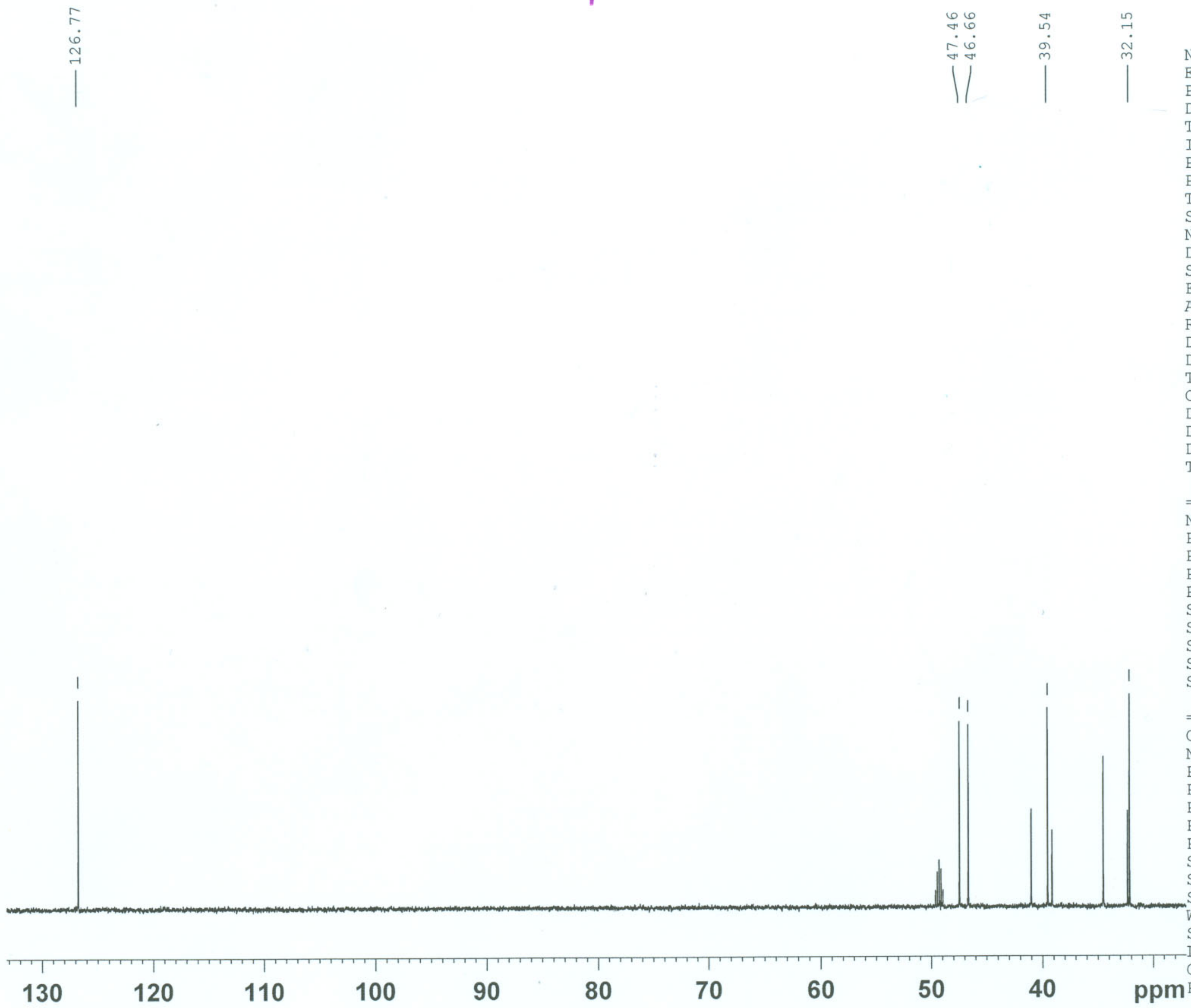

NAME	jun16-15
EXPNO	3
PROCNO	1
Date_	20150617
Time	9.20
INSTRUM	spect
PROBHD	5 mm BBI 1H/D-
PULPROG	deptsp90
TD	32768
SOLVENT	MeOD
NS	4739
DS	2
SWH	23809.523 Hz
FIDRES	0.726609 Hz
AQ	0.6881990 sec
RG	32768
DW	21.000 usec
DE	6.50 usec
TE	299.8 K
CNST2	145.0000000
D1	1.50000000 sec
D2	0.00344828 sec
D12	0.00002000 sec
TD0	6
======== CHANNEL f1 ========	
NUC1	13C
P1	12.00 usec
P12	2000.00 usec
PL0	120.00 dB
PL1	-3.00 dB
SFO1	125.7697360 MHz
SP2	3.58 dB
SPNAM2	Crp60comp.4
SPOAL2	0.500
SPOFFS2	0.00 Hz
======== CHANNEL f2 ========	
CPDPRG2	waltz16
NUC2	1H
P3	8.00 usec
P4	16.00 usec
PCPD2	80.00 usec
PL2	-1.00 dB
PL12	19.20 dB
SFO2	500.1335009 MHz
SI	32768
SF	125.7576112 MHz
WDW	EM
SSB	0
LB	1.00 Hz
GB	0
PC	1.40

MAHWISH/DR.IQBAL/EC-M-2/CD3OD
ICCBS/U.O.K
HSQC

Compound 2

AVANCE AV-500
LAB NO: 109B

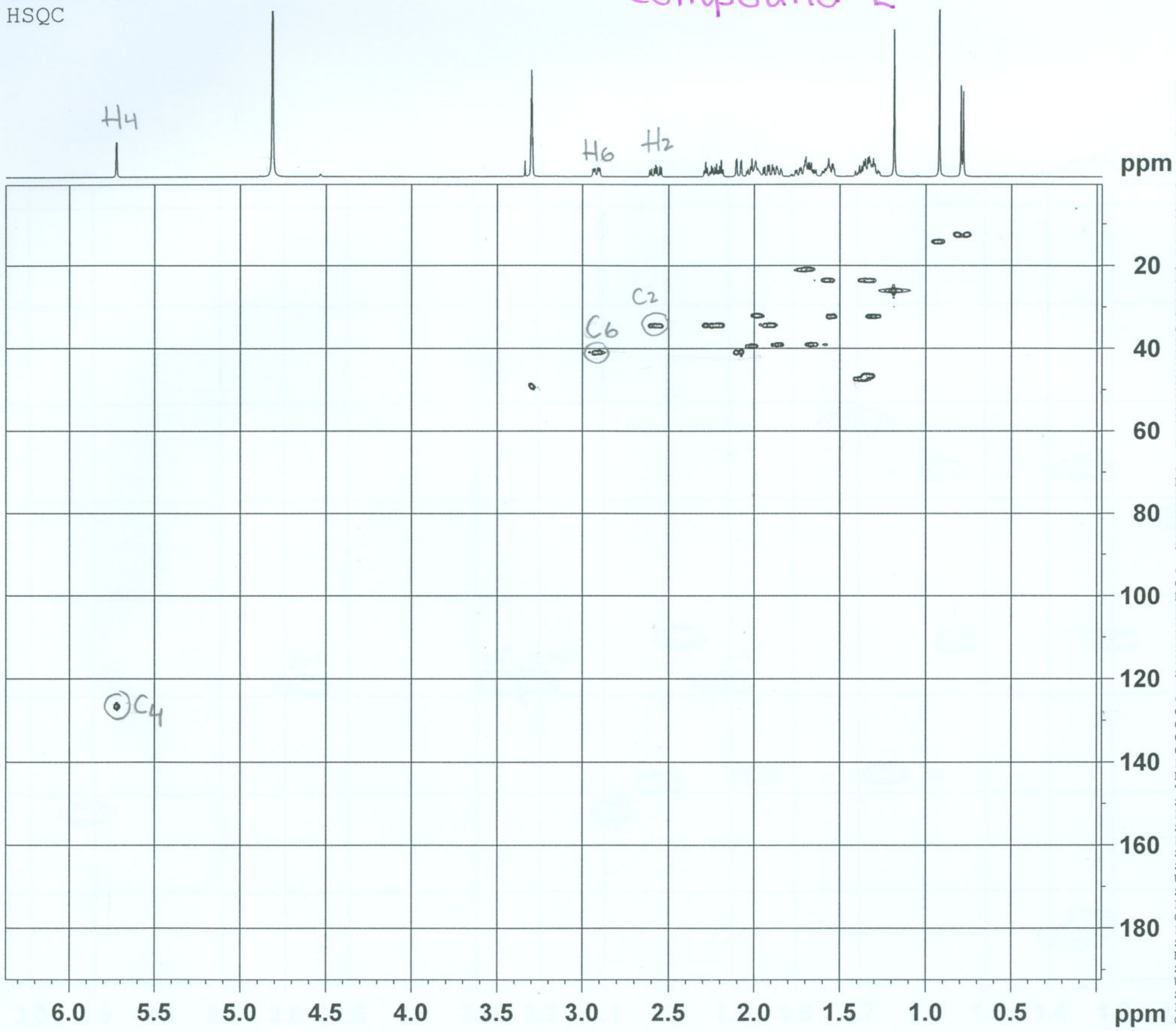

NAME	jun15-15	
EXPNO	16	
PROCNO	1	
Date_	20150615	
Time	20.45	
INSTRUM	spect	
PROBHD	5 mm BBI 1H/D-	
PULPROG	hsqcetgpsi	
TD	1024	
SOLVENT	MeOD	
NS	32	
DS	8	
SWH	3205.128	Hz
FIDRES	3.130008	Hz
AQ	0.1599500	sec
RG	23170.5	
DW	156.000	usec
DE	6.50	usec
TE	298.9	K
CNST2	145.0000000	
D0	0.00000300	sec
D1	1.50000000	sec
D4	0.00172414	sec
D11	0.03000000	sec
D13	0.00000400	sec
D16	0.00020000	sec
D24	0.00110000	sec
IN0	0.00002070	sec
ZGOPTNS		
======== CHANNEL f1 ========		
NUC1	1H	
P1	8.00	usec
P2	16.00	usec
P28	1000.00	usec
PL1	-1.00	dB
SFO1	500.1316004	MHz
======== CHANNEL f2 ========		
CPDPRG2	garp	
NUC2	13C	
P3	12.00	usec
P4	24.00	usec
PCPD2	65.00	usec
PL2	-3.00	dB
PL12	11.67	dB
SFO2	125.7697360	MHz
====== GRADIENT CHANNEL =====		
GPNAM1	SINE.100	
GPNAM2	SINE.100	
GPZ1	80.00	%
GPZ2	20.10	%
P16	1000.00	usec
ND0	2	
TD	256	
SFO1	125.7697	MHz
FIDRES	94.327301	Hz
SW	192.000	ppm
FnMODE	Echo-Antiecho	
SI	1024	
SF	500.1300158	MHz
WDW	QSINE	
SSB	2	
LB	0.00	Hz
GB	0	
PC	4.00	
SI	1024	
MC2	echo-antiecho	
SF	125.7576112	MHz
WDW	QSINE	
SSB	2	
LB	0.00	Hz
GB	0	

MAHWISH/DR.IQBAL/EC-M-2/CD3OD
ICCBS/U.O.K
HMBC

AVANCE AV-500
LAB NO: 109B

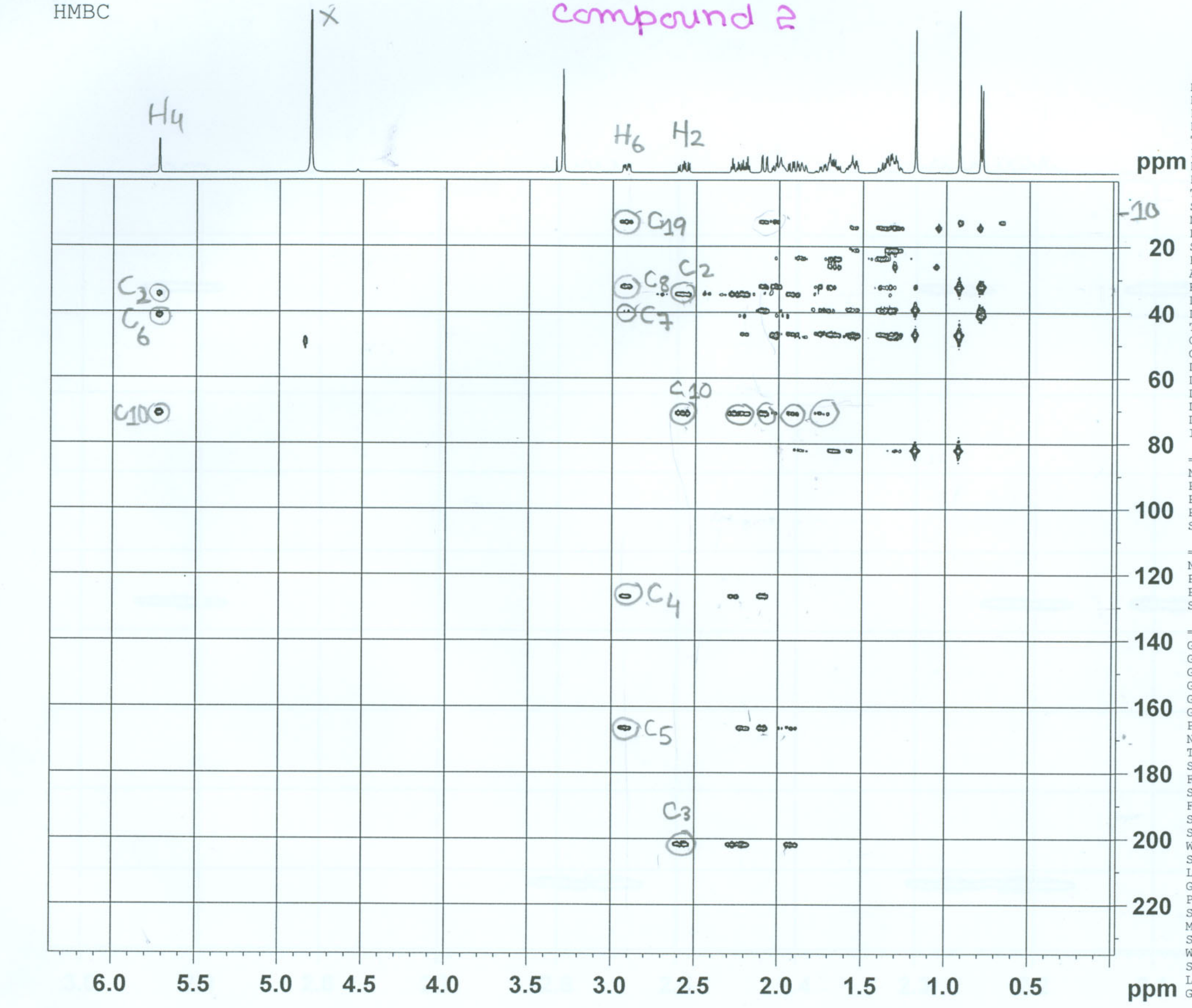

NAME	jun15-15	
EXPNO	17	
PROCNO	1	
Date_	20150616	
Time	0.37	
INSTRUM	spect	
PROBHD	5 mm BBI 1H/D-	
PULPROG	hmbcgplpndqf	
TD	2048	
SOLVENT	MeOD	
NS	64	
DS	8	
SWH	3205.128	Hz
FIDRES	1.565004	Hz
AQ	0.3196940	sec
RG	20642.5	
DW	156.000	usec
DE	6.50	usec
TE	298.9	K
CNST2	145.0000000	
CNST13	10.0000000	
D0	0.00000300	sec
D1	2.00000000	sec
D2	0.00344828	sec
D6	0.05000000	sec
D16	0.00020000	sec
IN0	0.00001690	sec
======== CHANNEL f1 ========		
NUC1	1H	
P1	8.00	usec
P2	16.00	usec
PL1	-1.00	dB
SFO1	500.1316004	MHz
======== CHANNEL f2 ========		
NUC2	13C	
P3	12.00	usec
PL2	-3.00	dB
SFO2	125.7723769	MHz
====== GRADIENT CHANNEL =====		
GPNAM1	SINE.100	
GPNAM2	SINE.100	
GPNAM3	SINE.100	
GPZ1	50.00	%
GPZ2	30.00	%
GPZ3	40.10	%
P16	1000.00	usec
ND0	2	
TD	256	
SFO1	125.7724	MHz
FIDRES	115.455109	Hz
SW	235.000	ppm
FnMODE	QF	
SI	1024	
SF	500.1300158	MHz
WDW	SINE	
SSB	0	
LB	0.00	Hz
GB	0	
PC	4.00	
SI	1024	
MC2	QF	
SF	125.7576112	MHz
WDW	SINE	
SSB	0	
LB	0.00	Hz
GB	0	

MAHWISH/DR.IQBAL/EC-M-2/CD3OD
ICCBS/U.O.K
COSY

AVANCE AV-500
LAB NO: 109B

Comp 2

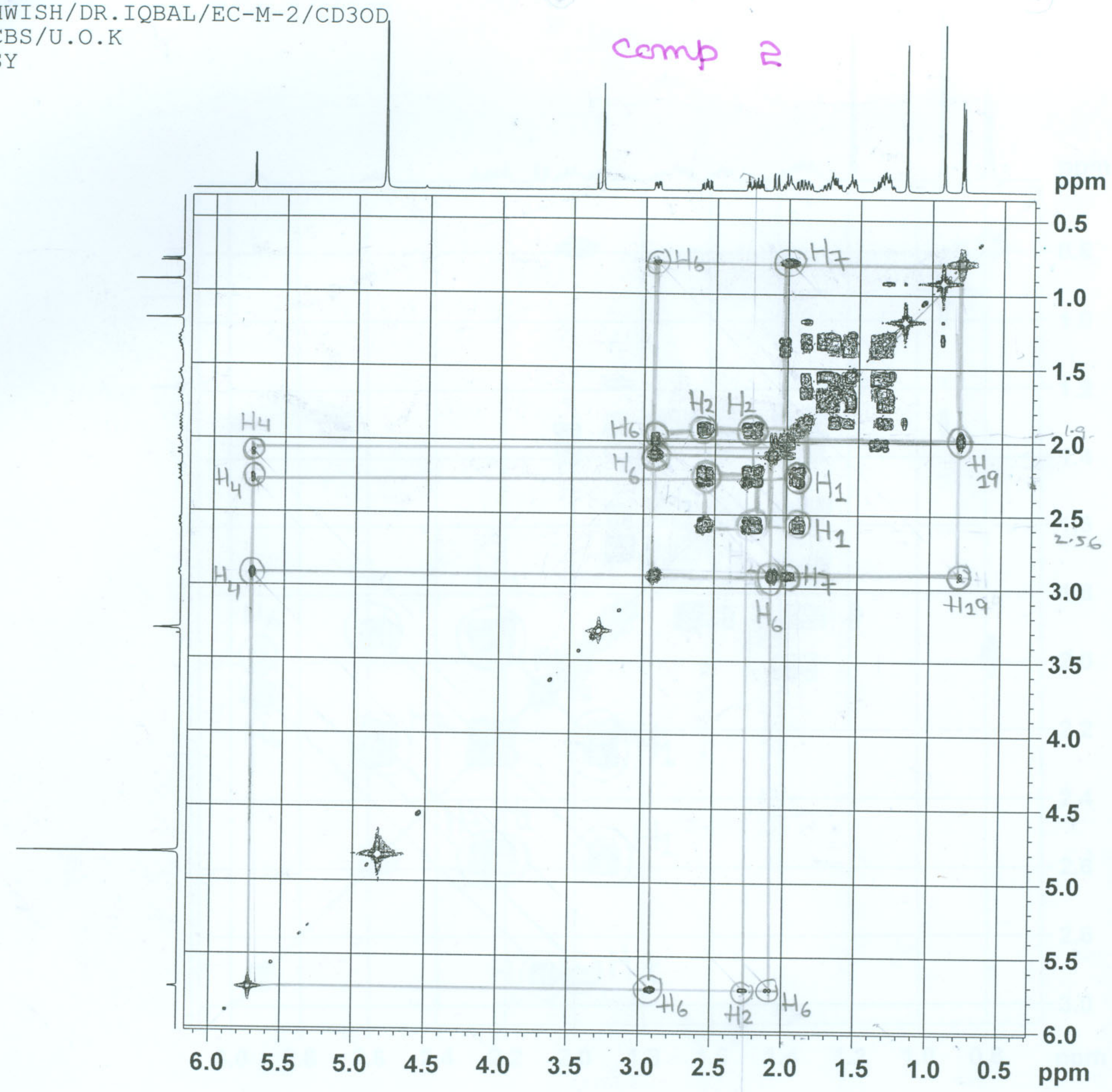

NAME	jun15-15
EXPNO	14
PROCNO	1
Date_	20150615
Time	16.06
INSTRUM	spect
PROBHD	5 mm BBI 1H/D-
PULPROG	cosygpqf
TD	2048
SOLVENT	MeOD
NS	8
DS	8
SWH	3205.128 Hz
FIDRES	1.565004 Hz
AQ	0.3196940 sec
RG	161.3
DW	156.000 usec
DE	6.50 usec
TE	301.5 K
D0	0.00000300 sec
D1	1.50000000 sec
D13	0.00000400 sec
D16	0.00020000 sec
IN0	0.00031200 sec

======== CHANNEL f1 ========

NUC1	1H
P0	8.00 usec
P1	8.00 usec
PL1	-1.00 dB
SFO1	500.1316004 MHz

====== GRADIENT CHANNEL =====

GPNAM1	SINE.100
GPZ1	10.00 %
P16	1000.00 usec
ND0	1
TD	256
SFO1	500.1316 MHz
FIDRES	12.520031 Hz
SW	6.409 ppm
FnMODE	QF
SI	1024
SF	500.1300158 MHz
WDW	SINE
SSB	0
LB	0.00 Hz
GB	0
PC	4.00
SI	1024
MC2	QF
SF	500.1300158 MHz
WDW	SINE
SSB	0
LB	0.00 Hz
GB	0

MAHWISH/DR.IQBAL/EC-M-2/CD3OD
ICCBS/U.O.K
NOESY

Comp 2

AVANCE AV-500
LAB NO: 109B

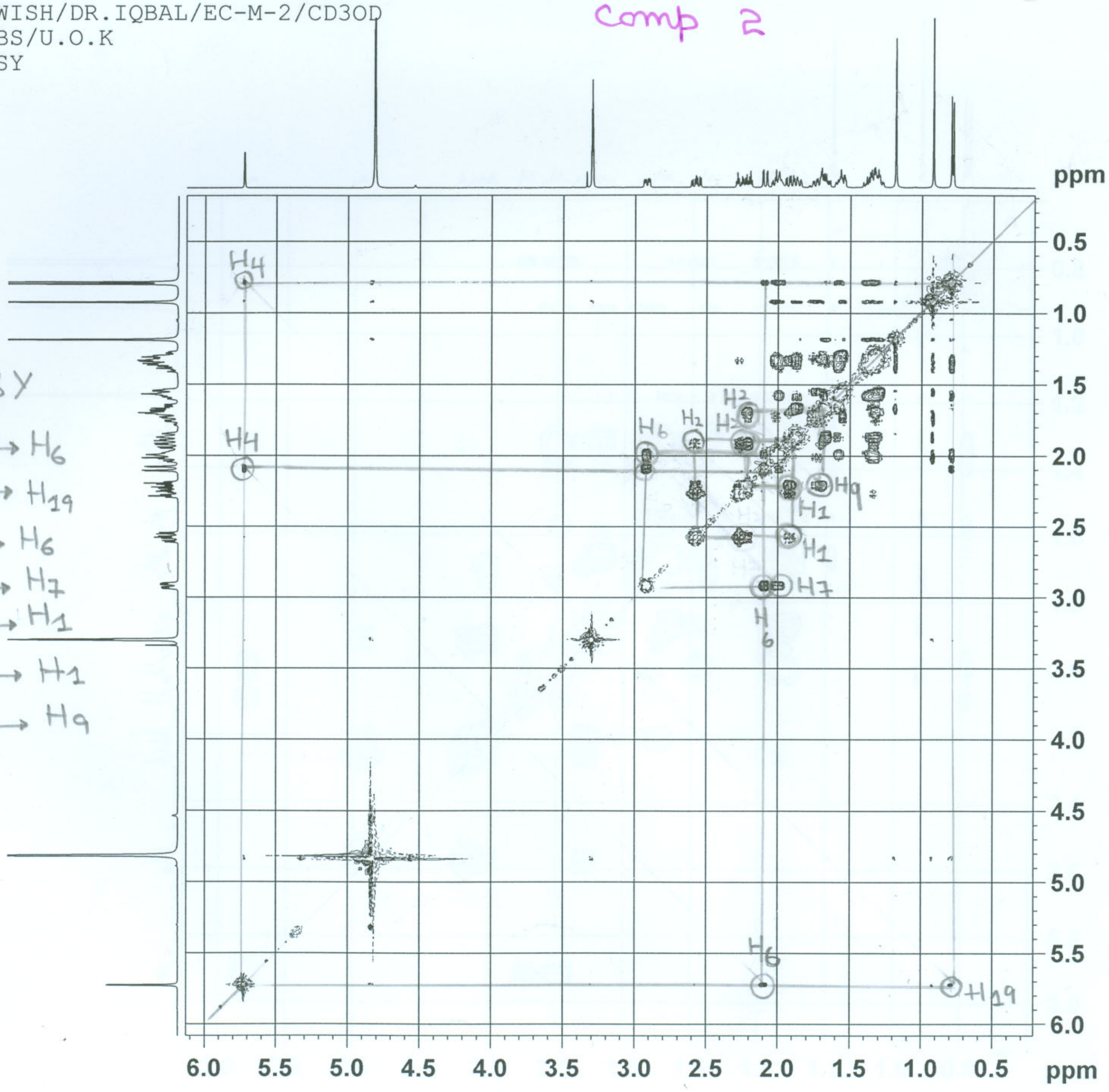

NOESY

H4 ⟷ H6
H4 ⟷ H19
H6 ⟷ H6
H6 ⟷ H7
H2 ⟷ H1
H2 ⟷ H1
H2 ⟷ H9

NAME	jun15-15	
EXPNO	15	
PROCNO	1	
Date_	20150615	
Time	17.10	
INSTRUM	spect	
PROBHD	5 mm BBI 1H/D-	
PULPROG	noesygpph	
TD	2048	
SOLVENT	MeOD	
NS	16	
DS	2	
SWH	3205.128	Hz
FIDRES	1.565004	Hz
AQ	0.3196940	sec
RG	228.1	
DW	156.000	usec
DE	6.50	usec
TE	300.1	K
D0	0.00014581	sec
D1	2.00000000	sec
D8	0.80000001	sec
D16	0.00020000	sec
IN0	0.00031200	sec

======== CHANNEL f1 ========

NUC1	1H	
P1	8.00	usec
P2	16.00	usec
PL1	-1.00	dB
SFO1	500.1316004	MHz

====== GRADIENT CHANNEL =====

GPNAM1	SINE.100	
GPZ1	40.00	%
P16	1000.00	usec
ND0	1	
TD	256	
SFO1	500.1316	MHz
FIDRES	12.520031	Hz
SW	6.409	ppm
FnMODE	States-TPPI	
SI	1024	
SF	500.1300158	MHz
WDW	QSINE	
SSB	2	
LB	0.00	Hz
GB	0	
PC	4.00	
SI	1024	
MC2	States-TPPI	
SF	500.1300158	MHz
WDW	QSINE	
SSB	2	
LB	0.00	Hz
GB	0	

MAHWISH/DR.IQBAL/EC-M-2/DMSO

AVANCE AV-500
LAB NO:118

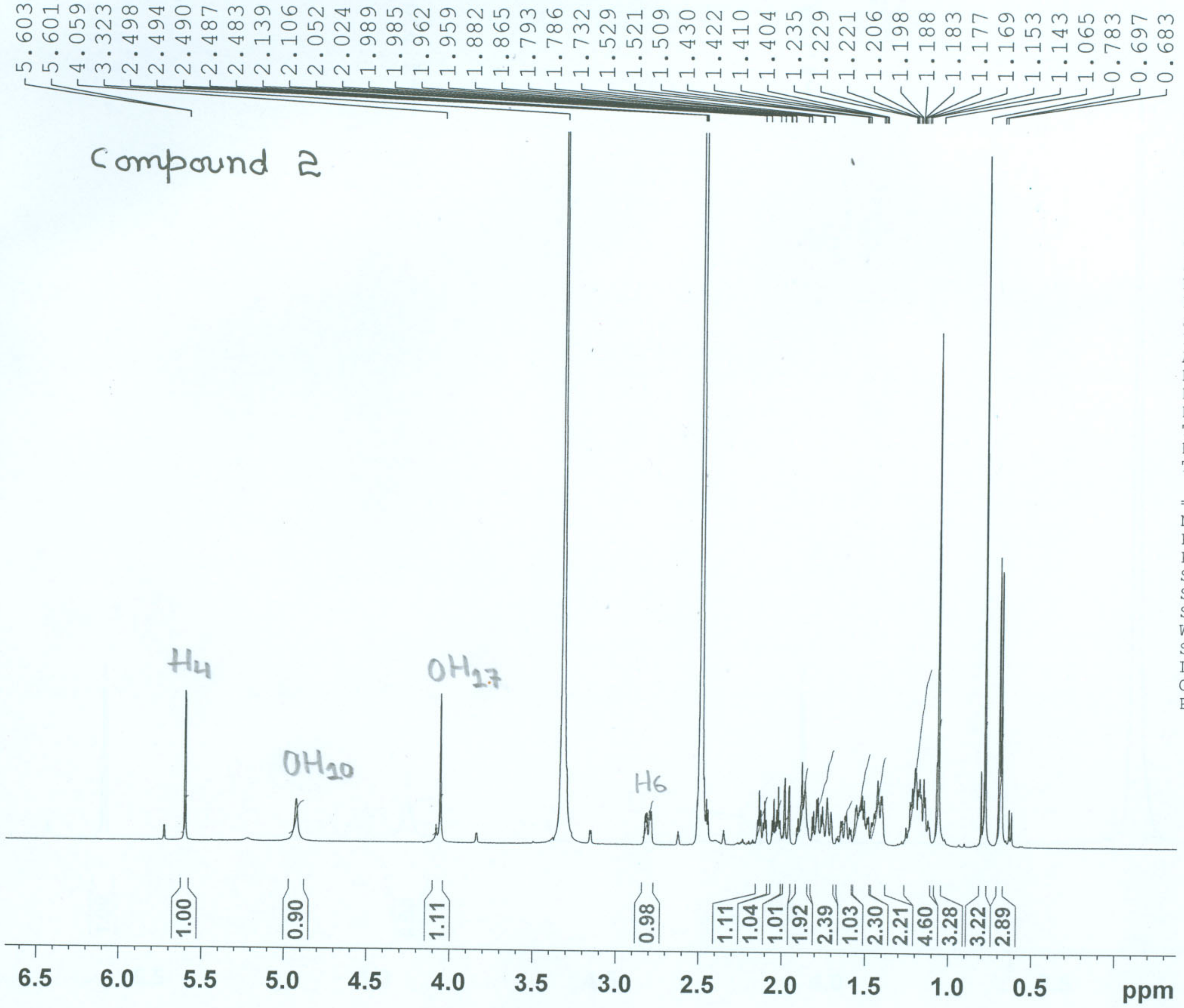

NAME	jan11-16
EXPNO	1
PROCNO	1
Date_	20160111
Time	10.48
INSTRUM	spect
PROBHD	5 mm PABBI 1H/
PULPROG	zg30
TD	65536
SOLVENT	DMSO
NS	128
DS	0
SWH	12019.230 Hz
FIDRES	0.183399 Hz
AQ	2.7263892 sec
RG	256
DW	41.600 usec
DE	6.50 usec
TE	296.7 K
D1	2.00000000 sec
TD0	1

======== CHANNEL f1 ========

NUC1	1H
P1	8.03 usec
PL1	3.00 dB
SFO1	500.2350023 MHz
SI	32768
SF	500.2300060 MHz
WDW	EM
SSB	0
LB	0.30 Hz
GB	0
PC	1.00

MAHWISH/DR.IQBAL/EC-M-2/CD3OD
ICCBS/U.O.K
NOESY

Comp 2

AVANCE AV-500
LAB NO: 109B

NOESY

H4 ⟷ H6
H4 ⟷ H19
H6 ⟷ H6
H6 ⟷ H7
H2 ⟷ H1
H2 ⟷ H1
H2 ⟷ H9

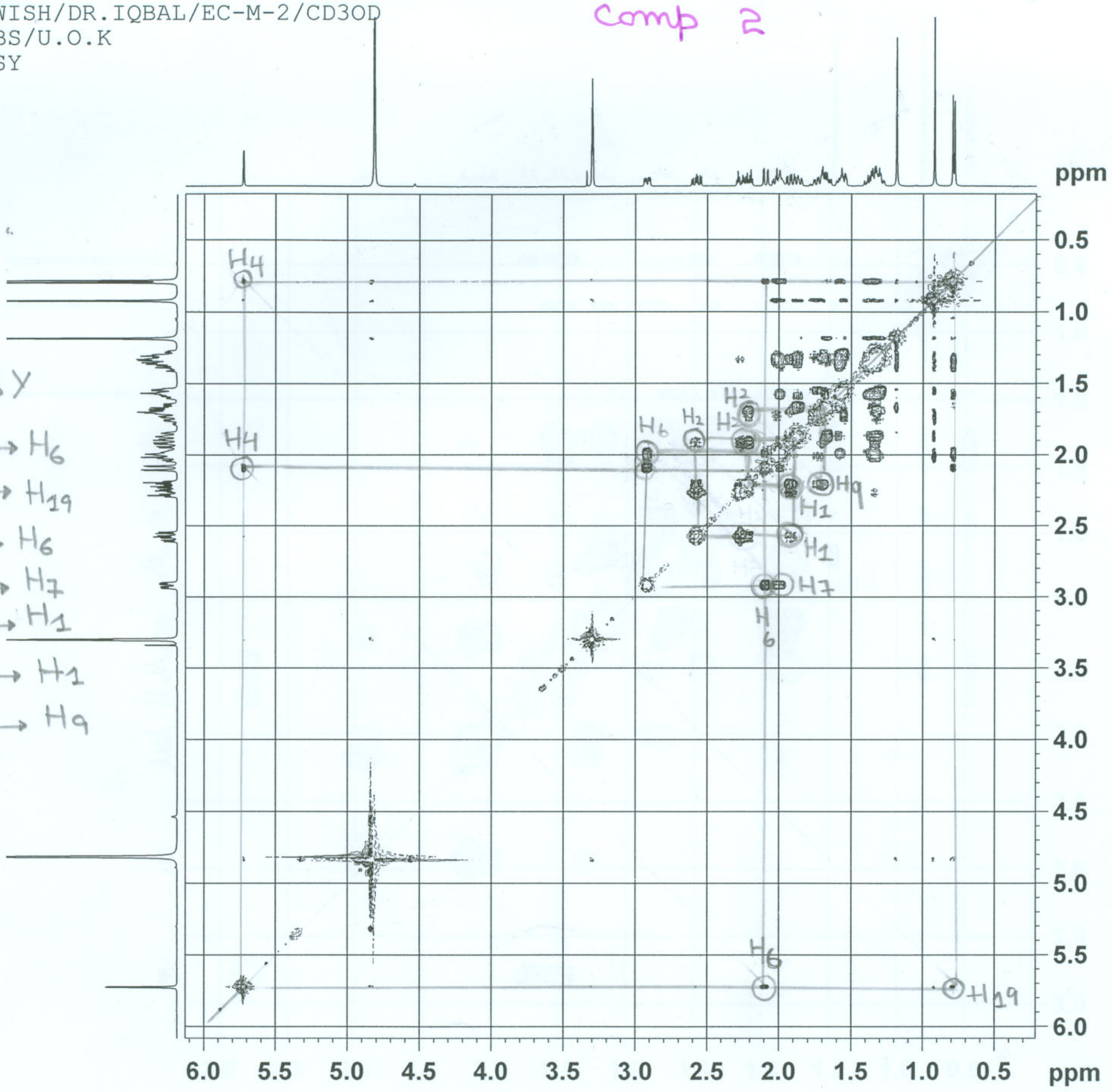

NAME	jun15-15	
EXPNO	15	
PROCNO	1	
Date_	20150615	
Time	17.10	
INSTRUM	spect	
PROBHD	5 mm BBI 1H/D-	
PULPROG	noesygpph	
TD	2048	
SOLVENT	MeOD	
NS	16	
DS	2	
SWH	3205.128	Hz
FIDRES	1.565004	Hz
AQ	0.3196940	sec
RG	228.1	
DW	156.000	usec
DE	6.50	usec
TE	300.1	K
D0	0.00014581	sec
D1	2.00000000	sec
D8	0.80000001	sec
D16	0.00020000	sec
IN0	0.00031200	sec

======== CHANNEL f1 ========

NUC1	1H	
P1	8.00	usec
P2	16.00	usec
PL1	-1.00	dB
SFO1	500.1316004	MHz

====== GRADIENT CHANNEL =====

GPNAM1	SINE.100	
GPZ1	40.00	%
P16	1000.00	usec
ND0	1	
TD	256	
SFO1	500.1316	MHz
FIDRES	12.520031	Hz
SW	6.409	ppm
FnMODE	States-TPPI	
SI	1024	
SF	500.1300158	MHz
WDW	QSINE	
SSB	2	
LB	0.00	Hz
GB	0	
PC	4.00	
SI	1024	
MC2	States-TPPI	
SF	500.1300158	MHz
WDW	QSINE	
SSB	2	
LB	0.00	Hz
GB	0	

MAHWISH/DR.IQBAL/EC-M-2/DMSO

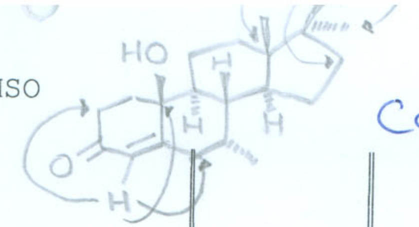

HMBC

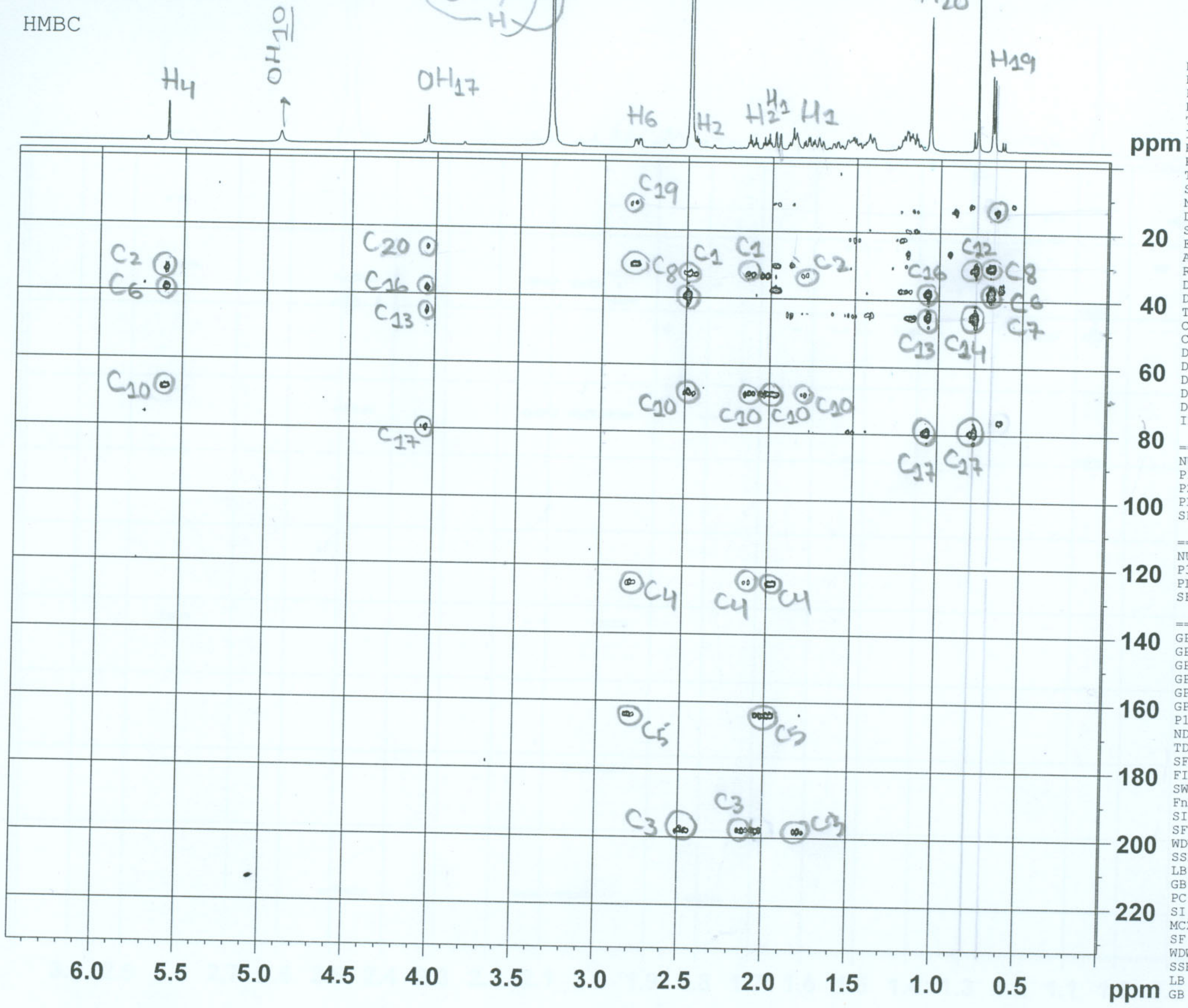

AVANCE AV-500
LAB NO:118

NAME	jan11-16
EXPNO	5
PROCNO	1
Date_	20160111
Time	19.20
INSTRUM	spect
PROBHD	5 mm PABBI 1H/
PULPROG	hmbcgplpndqf
TD	2048
SOLVENT	DMSO
NS	64
DS	16
SWH	3255.208 Hz
FIDRES	1.589457 Hz
AQ	0.3147764 sec
RG	20642.5
DW	153.600 usec
DE	6.50 usec
TE	298.9 K
CNST2	145.0000000
CNST13	10.0000000
D0	0.00000300 sec
D1	2.00000000 sec
D2	0.00344828 sec
D6	0.05000000 sec
D16	0.00020000 sec
IN0	0.00001690 sec
======== CHANNEL f1 ========	
NUC1	1H
P1	8.03 usec
P2	16.06 usec
PL1	3.00 dB
SFO1	500.2316257 MHz
======== CHANNEL f2 ========	
NUC2	13C
P3	13.35 usec
PL2	-3.00 dB
SFO2	125.7974871 MHz
====== GRADIENT CHANNEL =====	
GPNAM1	SINE.100
GPNAM2	SINE.100
GPNAM3	SINE.100
GPZ1	50.00 %
GPZ2	30.00 %
GPZ3	40.10 %
P16	1000.00 usec
ND0	2
TD	256
SFO1	125.7975 MHz
FIDRES	115.478165 Hz
SW	235.000 ppm
FnMODE	QF
SI	1024
SF	500.2300060 MHz
WDW	SINE
SSB	0
LB	0.00 Hz
GB	0
PC	1.00
SI	512
MC2	QF
SF	125.7829924 MHz
WDW	SINE
SSB	0
LB	0.00 Hz
GB	0

MAHWISH/DR.IQBAL/EC-M-2/DMSO

HSQC

Comp 2

AVANCE AV-500
LAB NO:118

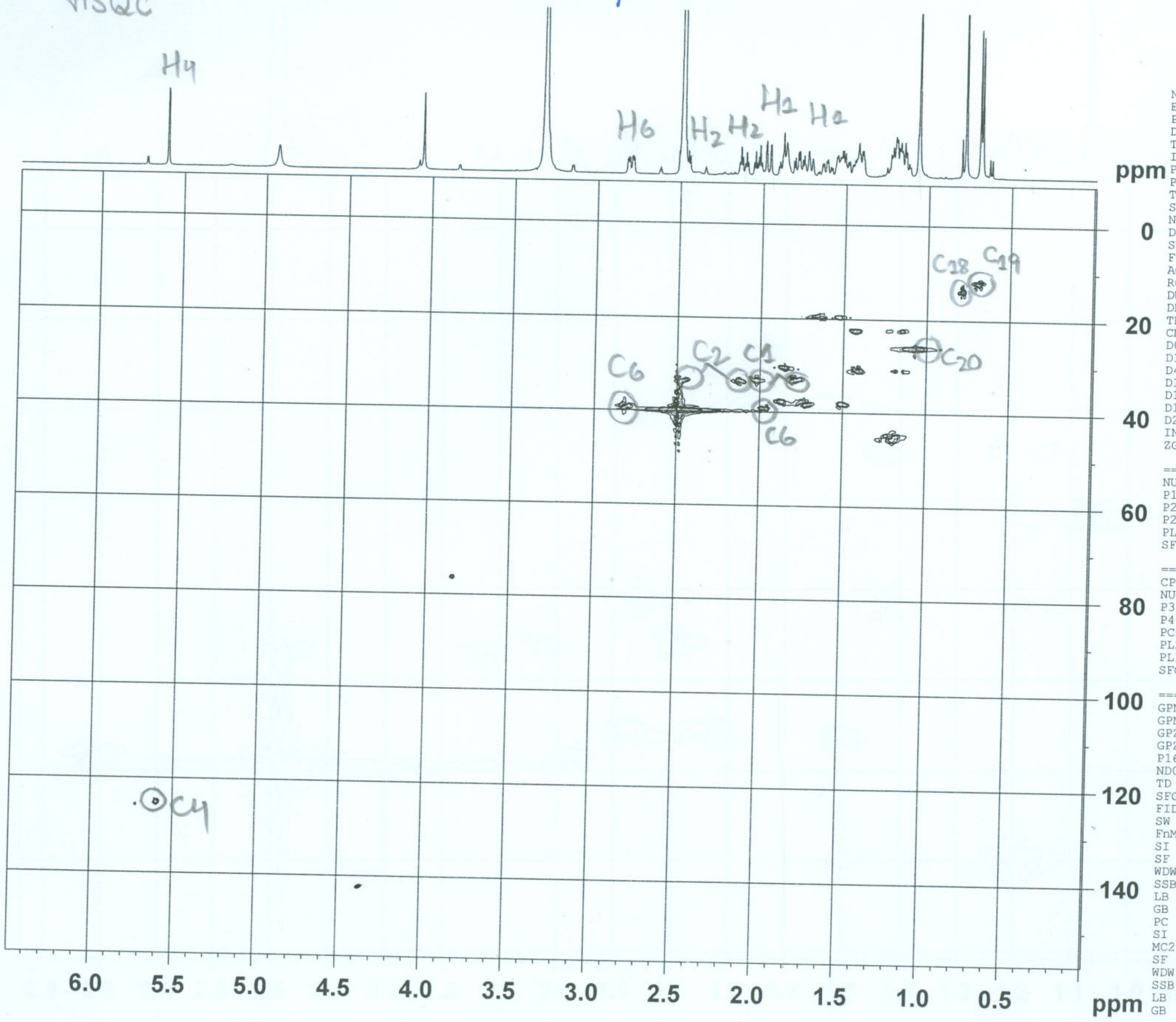

NAME	jan11-16	
EXPNO	4	
PROCNO	1	
Date_	20160111	
Time	15.28	
INSTRUM	spect	
PROBHD	5 mm PABBI 1H/	
PULPROG	hsqcedetgp	
TD	1024	
SOLVENT	DMSO	
NS	32	
DS	16	
SWH	3255.208	Hz
FIDRES	3.178914	Hz
AQ	0.1574900	sec
RG	18390.4	
DW	153.600	usec
DE	6.50	usec
TE	296.6	K
CNST2	145.0000000	
D0	0.00000300	sec
D1	1.50000000	sec
D4	0.00172414	sec
D11	0.03000000	sec
D13	0.00000400	sec
D16	0.00020000	sec
D21	0.00345000	sec
IN0	0.00002400	sec
ZGOPTNS		
======== CHANNEL f1 ========		
NUC1	1H	
P1	8.03	usec
P2	16.06	usec
P28	1000.00	usec
PL1	3.00	dB
SFO1	500.2316257	MHz
======== CHANNEL f2 ========		
CPDPRG2	garp	
NUC2	13C	
P3	13.35	usec
P4	26.70	usec
PCPD2	70.00	usec
PL2	-3.00	dB
PL12	10.00	dB
SFO2	125.7923151	MHz
====== GRADIENT CHANNEL =====		
GPNAM1	SINE.100	
GPNAM2	SINE.100	
GPZ1	80.00	%
GPZ2	20.10	%
P16	1000.00	usec
ND0	2	
TD	256	
SFO1	125.7923	MHz
FIDRES	81.391190	Hz
SW	165.639	ppm
FnMODE	Echo-Antiecho	
SI	1024	
SF	500.2300060	MHz
WDW	QSINE	
SSB	2	
LB	0.00	Hz
GB	0	
PC	0.80	
SI	1024	
MC2	echo-antiecho	
SF	125.7829924	MHz
WDW	QSINE	
SSB	2	
LB	0.00	Hz
GB	0	

MAHWISH/DR.IQBAL/EC-M-2/DMSO
ICCBS/U.O.K
COSY

AVANCE AV-500
LAB NO:118

Comp. 2

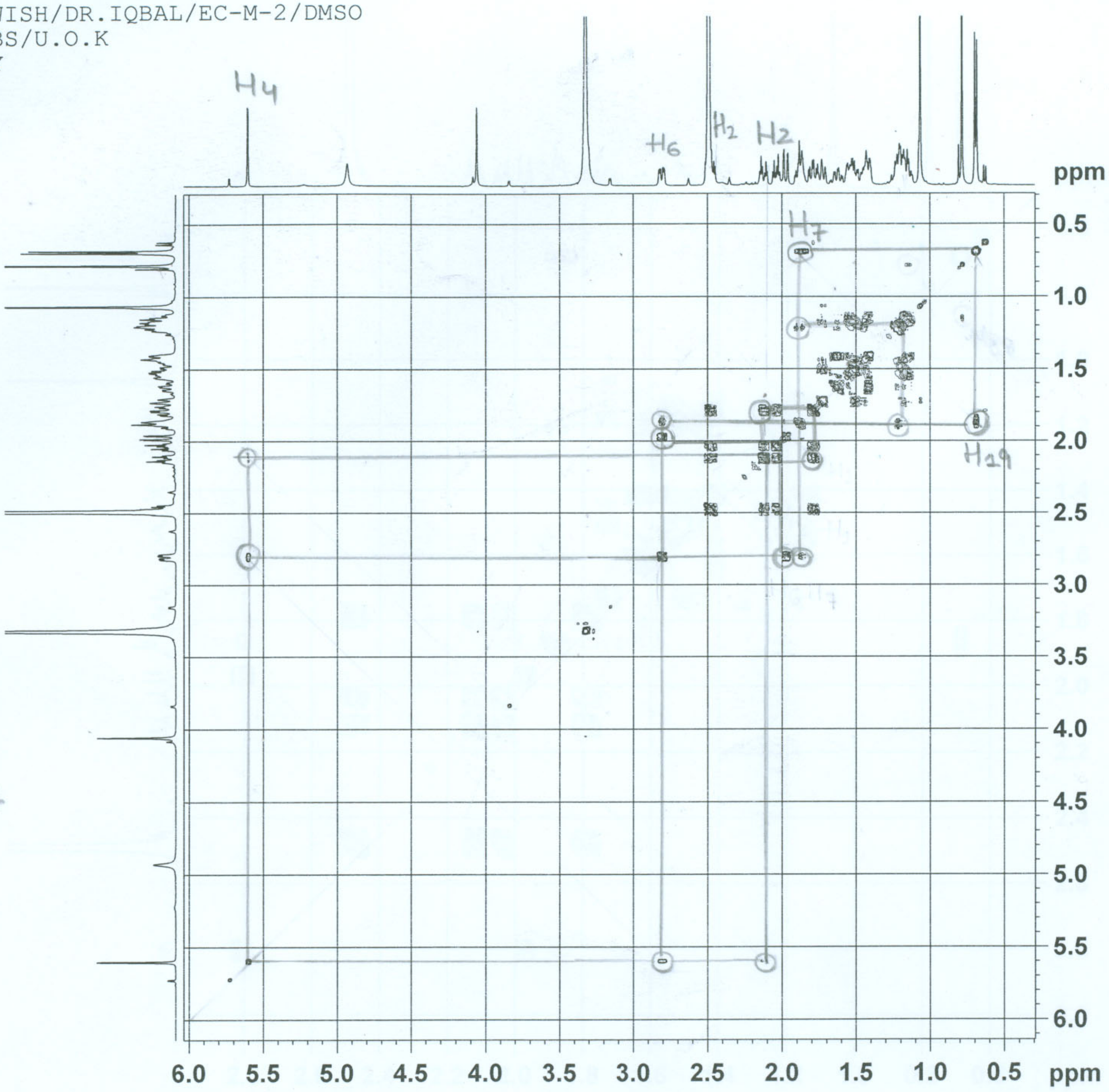

COSY

$H_4 \leftrightarrow H_6$
$H_4 \leftrightarrow H_2$
$H_6 \leftrightarrow H_6$
$H_6 \leftrightarrow H_7$

NAME	jan11-16	
EXPNO	2	
PROCNO	1	
Date_	20160111	
Time	10.48	
INSTRUM	spect	
PROBHD	5 mm PABBI 1H/	
PULPROG	cosydfqf	
TD	2048	
SOLVENT	DMSO	
NS	8	
DS	8	
SWH	3255.208	Hz
FIDRES	1.589457	Hz
AQ	0.3147764	sec
RG	362	
DW	153.600	usec
DE	6.50	usec
TE	296.7	K
D0	0.00000300	sec
D1	1.50000000	sec
D13	0.00000400	sec
D20	0.00000000	sec
IN0	0.00030720	sec

======== CHANNEL f1 ========

NUC1	1H	
P1	8.03	usec
PL1	3.00	dB
SFO1	500.2316257	MHz
ND0	1	
TD	256	
SFO1	500.2316	MHz
FIDRES	12.715656	Hz
SW	6.507	ppm
FnMODE	QF	
SI	1024	
SF	500.2300060	MHz
WDW	QSINE	
SSB	0	
LB	0.00	Hz
GB	0	
PC	1.00	
SI	1024	
MC2	QF	
SF	500.2300060	MHz
WDW	QSINE	
SSB	0	
LB	0.00	Hz
GB	0	

MAHWISH/DR.IQBAL/EC-M-2/DMSO
ICCBS/U.O.K
NOESY

Comp. 2

NOESY
H4 ⟷ H6
H6 ⟷ H6
H6 ⟷ H7
H8 ⟷ H18
H7 ⟷ H19
H9 ⟷ H19

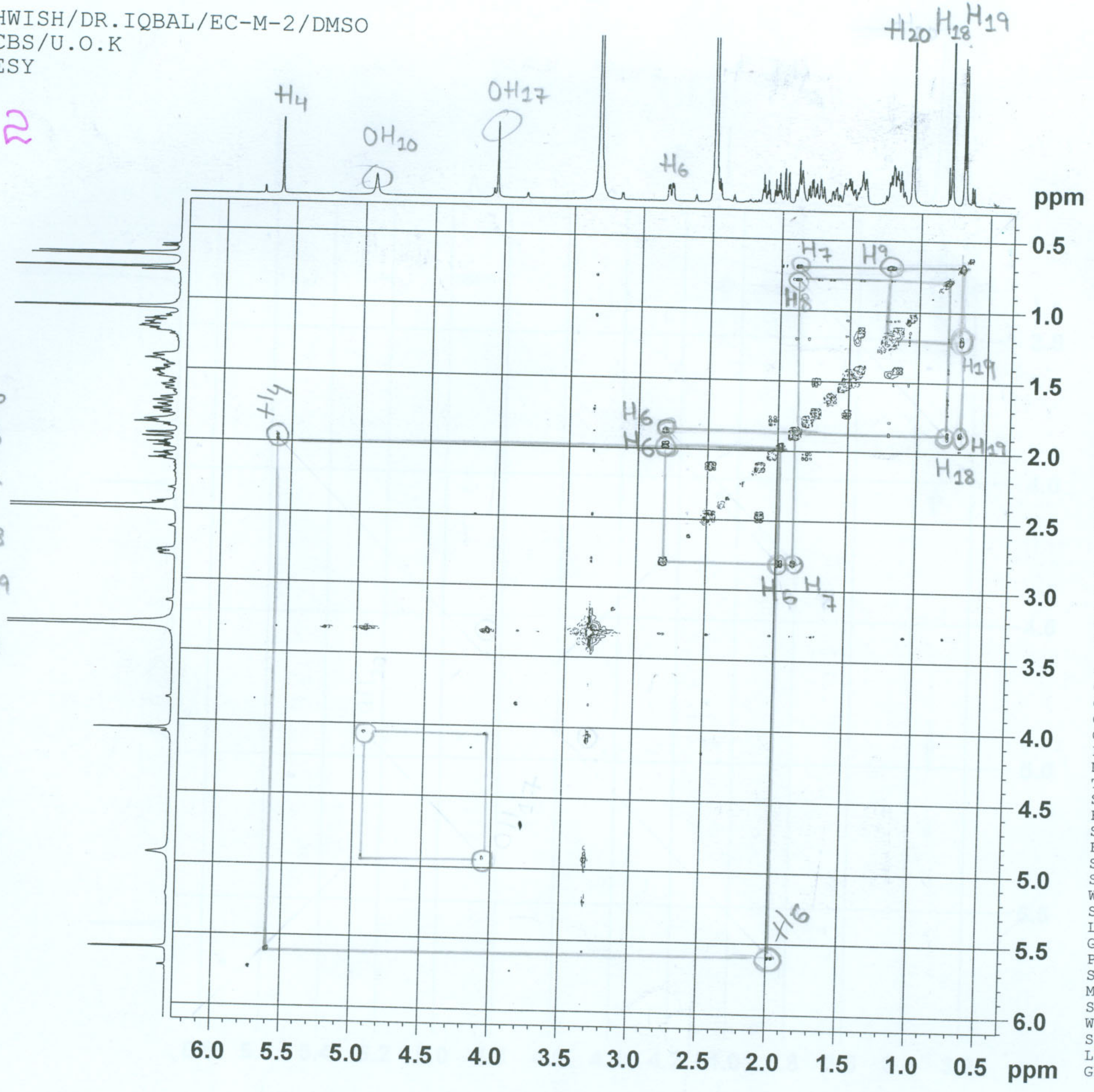

AVANCE AV-500
LAB NO:118

NAME	jan11-16	
EXPNO	3	
PROCNO	1	
Date_	20160111	
Time	11.53	
INSTRUM	spect	
PROBHD	5 mm PABBI 1H/	
PULPROG	noesygpph	
TD	2048	
SOLVENT	DMSO	
NS	16	
DS	8	
SWH	3255.208	Hz
FIDRES	1.589457	Hz
AQ	0.3147764	sec
RG	71.8	
DW	153.600	usec
DE	6.50	usec
TE	296.5	K
D0	0.00014338	sec
D1	2.00000000	sec
D8	0.80000001	sec
D16	0.00020000	sec
IN0	0.00030720	sec

======== CHANNEL f1 ========

NUC1	1H	
P1	8.03	usec
P2	16.06	usec
PL1	3.00	dB
SFO1	500.2316257	MHz

====== GRADIENT CHANNEL =====

GPNAM1	SINE.100	
GPNAM2	SINE.100	
GPZ1	40.00	%
GPZ2	-40.00	%
P16	1000.00	usec
ND0	1	
TD	256	
SFO1	500.2316	MHz
FIDRES	12.715656	Hz
SW	6.507	ppm
FnMODE	States-TPPI	
SI	1024	
SF	500.2300060	MHz
WDW	QSINE	
SSB	2	
LB	0.00	Hz
GB	0	
PC	1.00	
SI	1024	
MC2	States-TPPI	
SF	500.2300060	MHz
WDW	QSINE	
SSB	2	
LB	0.00	Hz
GB	0	

THERMO ELECTRON ~ VISIONpro SOFTWARE V4.10

Operator Name	Arshad Alam	Date of Report	11/6/2015
Department	Analytical laboratory#004 TWC	Time of Report	8:48:27AM
Organization	ICCBS.Karachi University.		
Information	Porf Dr. M.Iqbal /Mahwish.		

Scan Graph Compound 2

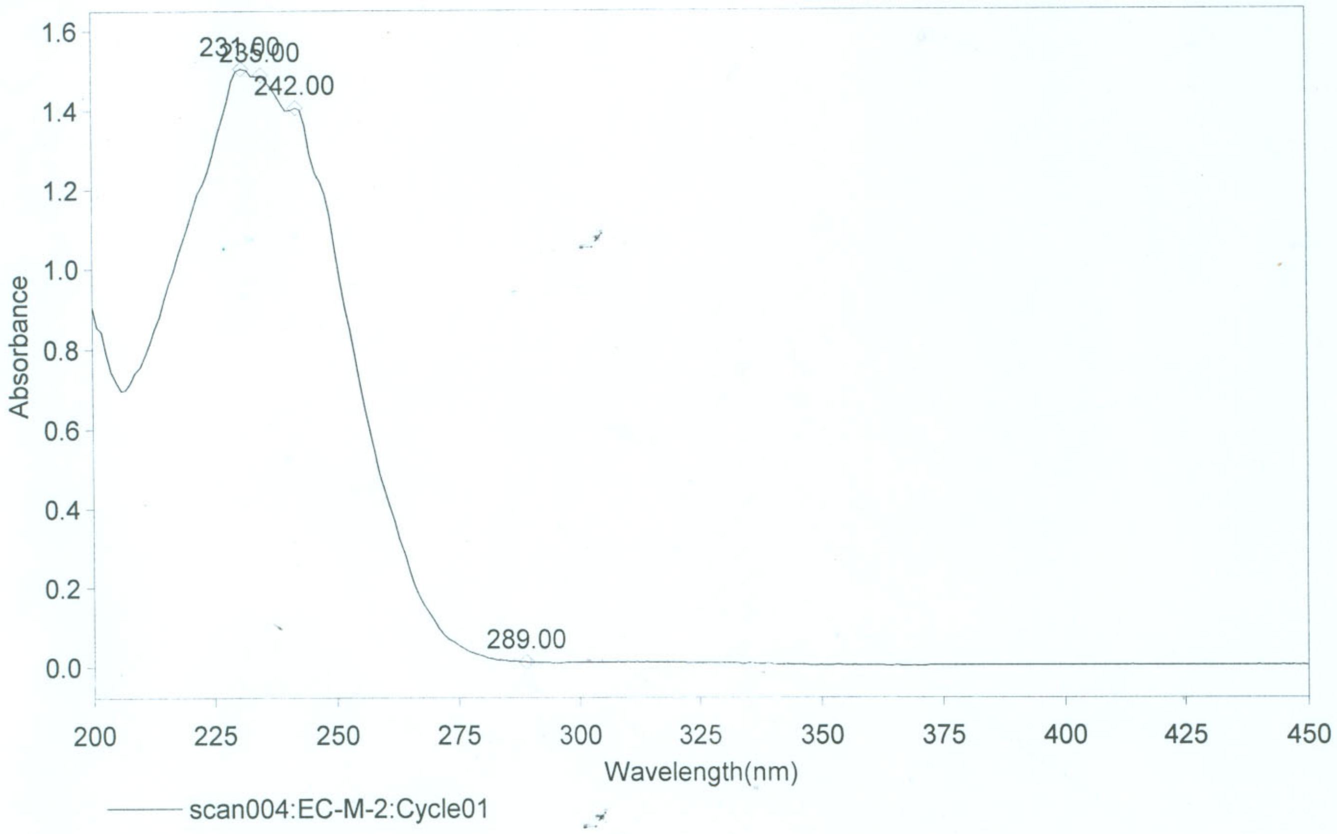

Results Table - EC-M-2.sre,EC-M-2,Cycle01

nm	A	**Peak Pick Method**
231.00	1.504	Find 8 Peaks Above -3.0000 A
235.00	1.488	Start Wavelength 200.00 nm
242.00	1.406	Stop Wavelength 290.00 nm
289.00	0.012	Sort By Wavelength
Sensitivity	Manual	
Rising Points	1	
Falling Points	1	
Min. Change	0.0000	

2 ml → 0.02 ml + 2 ml

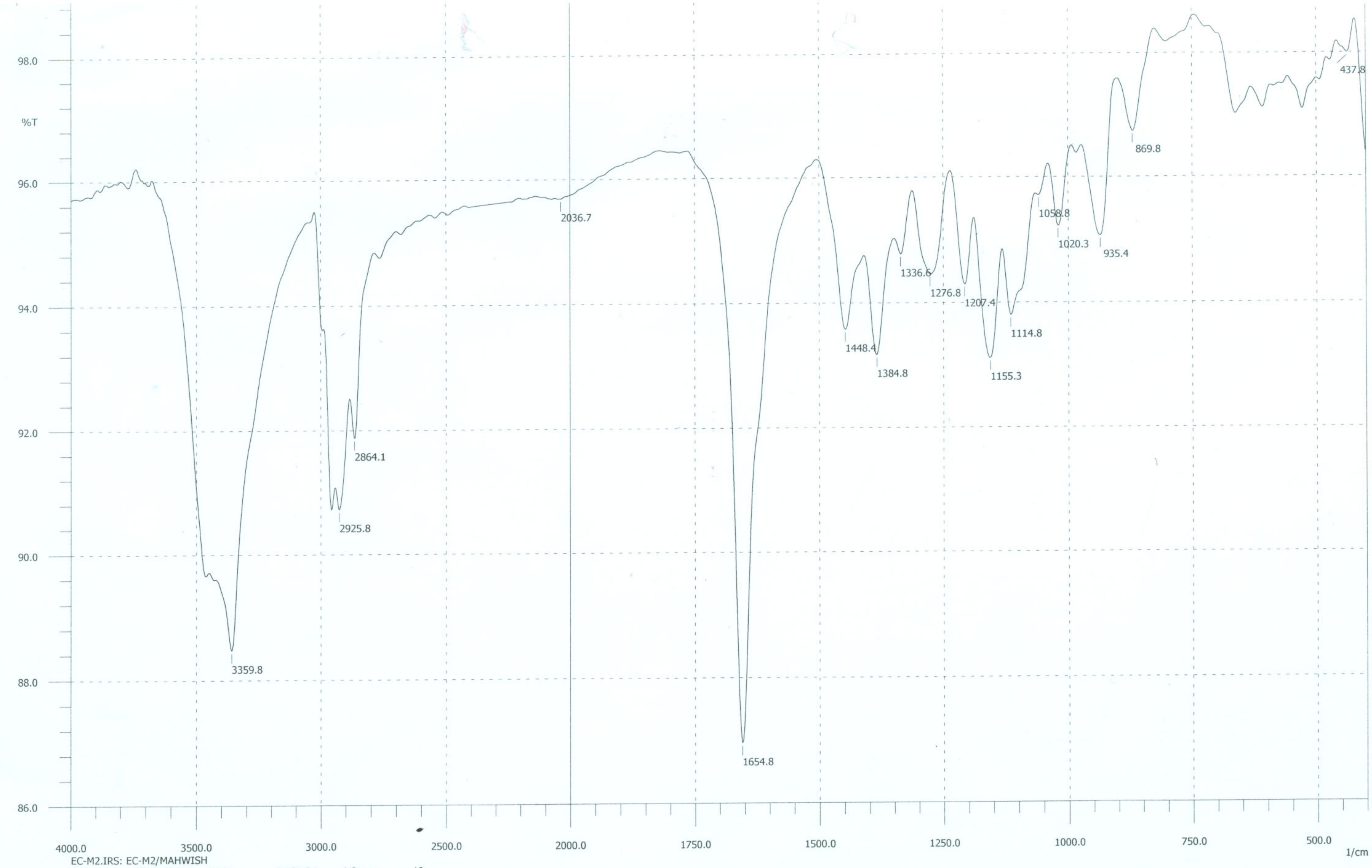

EC-M2.IRS: EC-M2/MAHWISH

Date:	06/30/2015	Time:	11:21:34	NScans:	10		
Type:	HYPER IR	User:	ZUBAIR AHMED			Detector:	standard
Abscissa:	1/cm	Ordinate:	%T	Apodization:	Happ		
Min:	401.17	Max:	3998.16	Range:	1/cm		
Ndp:	1866	Data Interval:	1.92868	Resolution:	4.0		
Gain:	auto	Aperture:	auto	Mirror Speed:	2.8(low)		

Compound 2
