# Supplementary material for: Biotransformation of a potent anabolic steroid, mibolerone, with Cunninghamella blakesleeana, C. echinulata, and Macrophomina phaseolina, and biological activity evaluation of its metabolites
Source: PLoS One. 2017 Feb 24;12(2):e0171476. doi: 10.1371/journal.pone.0171476 (PMC5325191; doi:10.1371/journal.pone.0171476)
Supplement: S2 Data — (PDF) [file pone.0171476.s002.pdf]

File: BM-10
Sample: MAHWISH /DR. IQBAL
Instrument: JEOL MS 600H-1

Date Run: 06-26-2015 (Time Run: 11:19:24)

Ionization mode: EI+

Compound 3

Scan: 11
Base: m/z 300; 78.2%FS TIC: 16094696

R.T.: .9

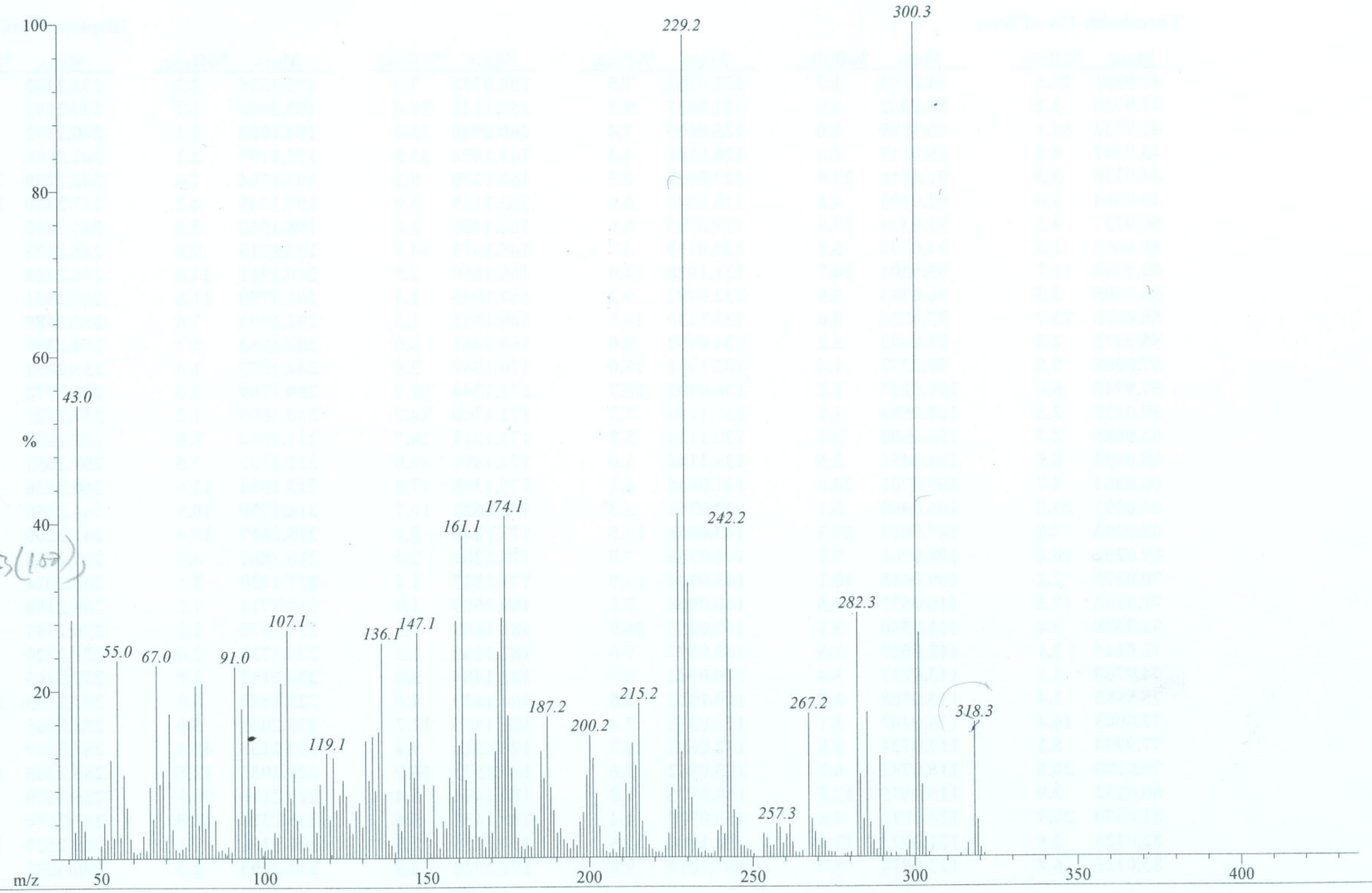

318.3 (16), 300.3 (107),

3

Compound 3

Mass	Relative Intensity	Theoretical Mass	Delta [ppm]	Delta [mmu]	RDB	Composition
258.1963	1.1	258.1984	-7.8	-2.0	6.0	$C_{18} H_{26} O_1$
260.1799	1.2	260.1776	8.8	2.3	6.0	$C_{17} H_{24} O_2$
261.1822	3.0	261.1855	-12.4	-3.2	5.5	$C_{17} H_{25} O_2$
262.1890	1.3	262.1933	-16.2	-4.2	5.0	$C_{17} H_{26} O_2$
267.1744	10.0	267.1749	-1.9	-0.5	8.5	$C_{19} H_{23} O_1$
268.1780	3.0	268.1827	-17.6	-4.7	8.0	$C_{19} H_{24} O_1$
269.1878	1.3	269.1905	-10.2	-2.8	7.5	$C_{19} H_{25} O_1$
282.1995	11.1	282.1984	4.0	1.1	8.0	$C_{20} H_{26} O_1$
283.2012	3.5	283.2062	-17.6	-5.0	7.5	$C_{20} H_{27} O_1$
284.2130	1.4	284.2140	-3.5	-1.0	7.0	$C_{20} H_{28} O_1$
285.1845	7.9	285.1855	-3.4	-1.0	7.5	$C_{19} H_{25} O_2$
286.1939	3.0	286.1933	2.2	0.6	7.0	$C_{19} H_{26} O_2$
287.1972	1.2	287.2011	-13.6	-3.9	6.5	$C_{19} H_{27} O_2$
289.2180	4.2	289.2168	4.5	1.3	5.5	$C_{19} H_{29} O_2$
290.2231	1.9	290.2246	-4.9	-1.4	5.0	$C_{19} H_{30} O_2$
298.1973	1.1	298.1933	13.6	4.1	8.0	$C_{20} H_{26} O_2$
300.2116	32.2	300.2089	8.9	2.7	7.0	$C_{20} H_{28} O_2$
301.2155	11.1	301.2168	-4.3	-1.3	6.5	$C_{20} H_{29} O_2$
302.2212	2.9	302.2246	-11.3	-3.4	6.0	$C_{20} H_{30} O_2$
318.2196	7.3	318.2195	0.3	0.1	6.0	$C_{20} H_{30} O_3$
319.2292	5.2	319.2273	6.0	1.9	5.5	$C_{20} H_{31} O_3$
320.2321	1.0	320.2351	-9.5	-3.0	5.0	$C_{20} H_{32} O_3$

m/z   cald.

[M]+

MAHWISH/DR.IQBAL/BM-10/CD3OD
ICCBS/U.O.K

AVANCE AV-500
LAB NO:118

Compound 3

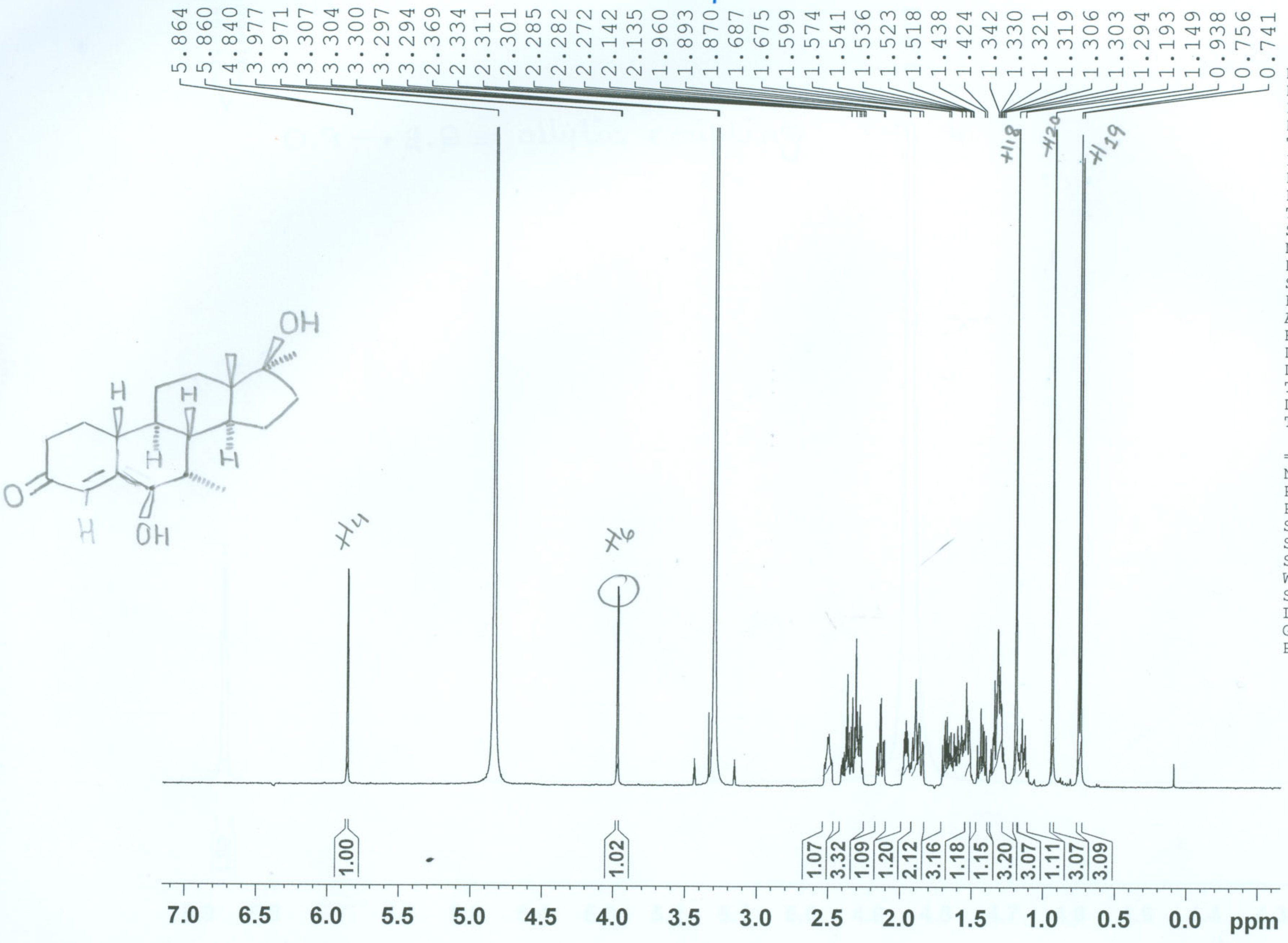

NAME	jun23-15
EXPNO	1
PROCNO	1
Date_	20150623
Time	9.27
INSTRUM	spect
PROBHD	5 mm PABBI 1H/
PULPROG	zg30
TD	32768
SOLVENT	MeOD
NS	128
DS	0
SWH	10000.000 Hz
FIDRES	0.305176 Hz
AQ	1.6385000 sec
RG	128
DW	50.000 usec
DE	6.50 usec
TE	299.0 K
D1	2.00000000 sec
TD0	1

======== CHANNEL f1 ========

NUC1	1H
P1	8.03 usec
PL1	3.00 dB
SFO1	500.2342520 MHz
SI	32768
SF	500.2300118 MHz
WDW	EM
SSB	0
LB	0.30 Hz
GB	0
PC	1.00

MAHWISH/DR.IQBAL/BM-10/CD3OD
ICCBS/U.O.K
B.B

Compound 3

AVANCE AV-500
LAB NO:118

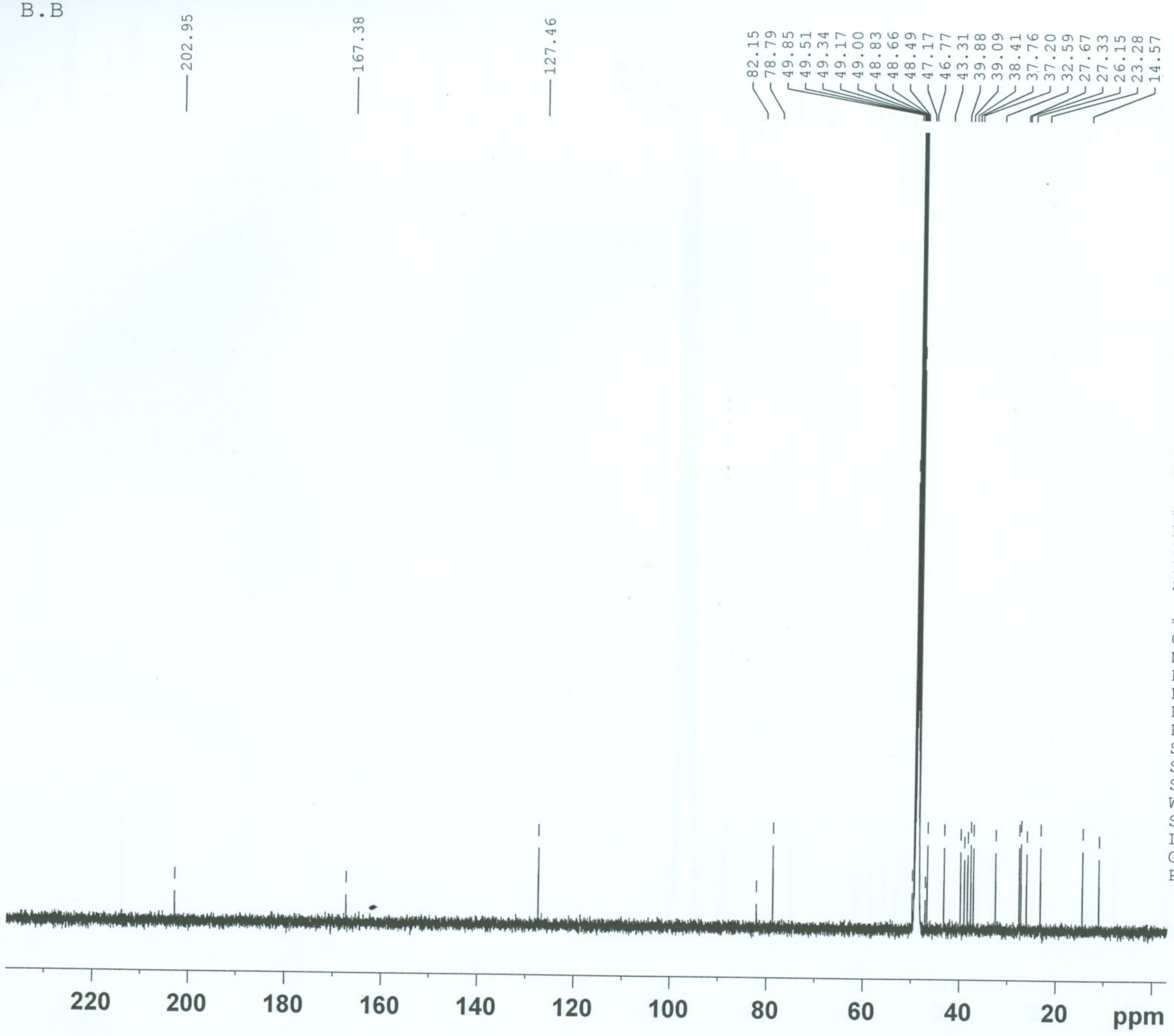

NAME	jun23-15
EXPNO	6
PROCNO	1
Date_	20150624
Time	5.47
INSTRUM	spect
PROBHD	5 mm PABBI 1H/
PULPROG	zgpg
TD	32768
SOLVENT	MeOD
NS	5386
DS	4
SWH	30303.031 Hz
FIDRES	0.924775 Hz
AQ	0.5407385 sec
RG	32768
DW	16.500 usec
DE	6.50 usec
TE	296.2 K
D1	2.00000000 sec
D11	0.03000000 sec
TD0	20

======== CHANNEL f1 ========

NUC1	13C
P1	13.35 usec
PL1	-3.00 dB
SFO1	125.7975248 MHz

======== CHANNEL f2 ========

CPDPRG2	waltz16
NUC2	1H
PCPD2	80.00 usec
PL2	3.00 dB
PL12	22.74 dB
PL13	26.00 dB
SFO2	500.2330014 MHz
SI	32768
SF	125.7827558 MHz
WDW	EM
SSB	0
LB	1.00 Hz
GB	0
PC	1.40

MAHWISH/DR.IQBAL/BM-10/CD3OD
DEPT135

Compound 3

AVANCE AV-500
LAB NO:118

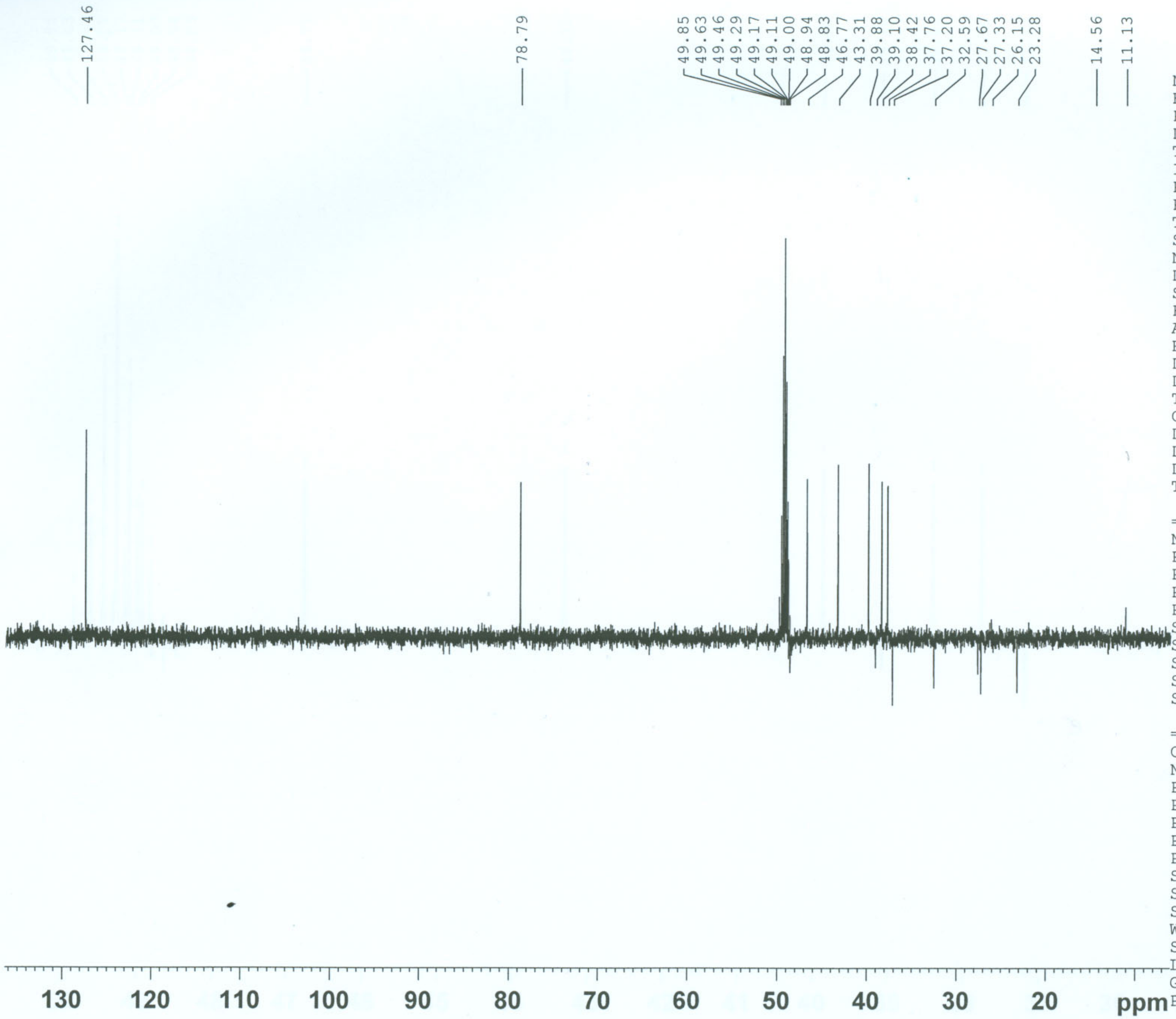

NAME	jun23-15
EXPNO	7
PROCNO	1
Date_	20150624
Time	12.37
INSTRUM	spect
PROBHD	5 mm PABBI 1H/
PULPROG	deptsp135
TD	32768
SOLVENT	MeOD
NS	16384
DS	4
SWH	24752.475 Hz
FIDRES	0.755386 Hz
AQ	0.6619838 sec
RG	32768
DW	20.200 usec
DE	6.50 usec
TE	300.1 K
CNST2	145.0000000
D1	1.50000000 sec
D2	0.00344828 sec
D12	0.00002000 sec
TD0	16

======== CHANNEL f1 ========

NUC1	13C
P1	13.35 usec
P12	2000.00 usec
PL0	120.00 dB
PL1	-3.00 dB
SFO1	125.7950092 MHz
SP2	2.65 dB
SPNAM2	Crp60comp.4
SPOAL2	0.500
SPOFFS2	0.00 Hz

======== CHANNEL f2 ========

CPDPRG2	waltz16
NUC2	1H
P3	8.03 usec
P4	16.06 usec
PCPD2	80.00 usec
PL2	3.00 dB
PL12	22.74 dB
SFO2	500.2330014 MHz
SI	32768
SF	125.7827558 MHz
WDW	EM
SSB	0
LB	1.00 Hz
GB	0
PC	0.80

MAHWISH/DR.IQBAL/BM-10/CD3OD
DEPT90

Compound 3

AVANCE AV-500
LAB NO:118

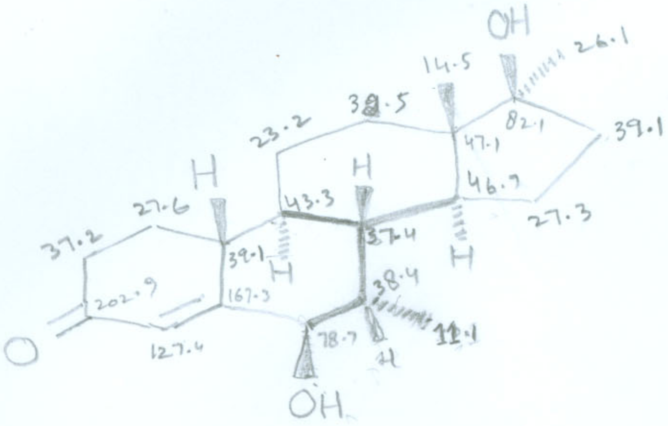

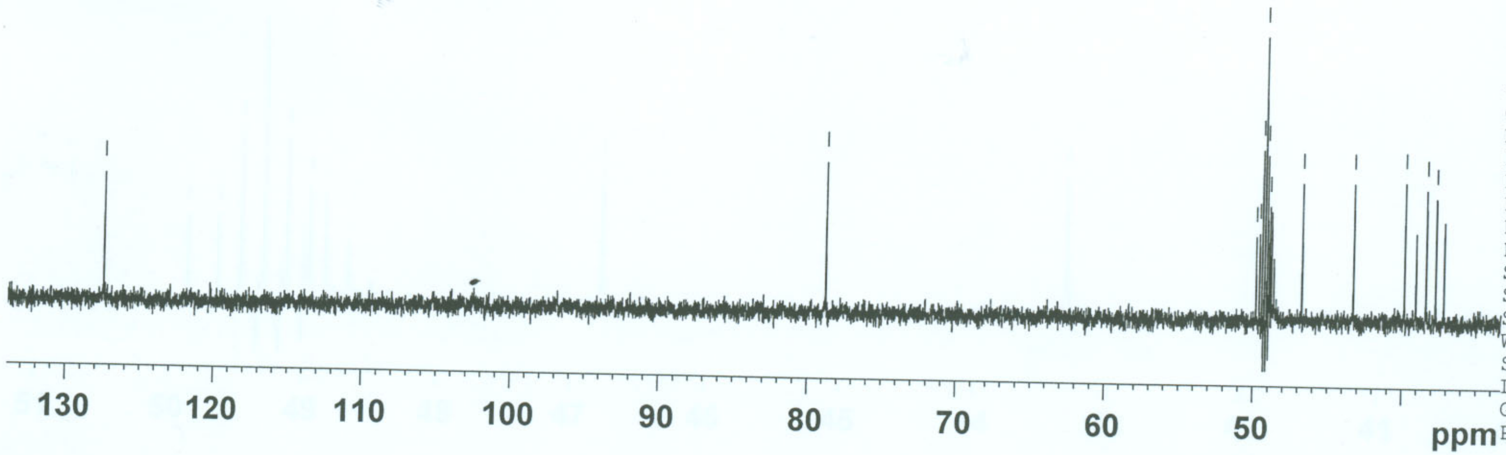

NAME	jun23-15	
EXPNO	8	
PROCNO	1	
Date_	20150624	
Time	22.40	
INSTRUM	spect	
PROBHD	5 mm PABBI 1H/	
PULPROG	deptsp90	
TD	32768	
SOLVENT	MeOD	
NS	7168	
DS	4	
SWH	24752.475	Hz
FIDRES	0.755386	Hz
AQ	0.6619838	sec
RG	32768	
DW	20.200	usec
DE	6.50	usec
TE	296.6	K
CNST2	145.0000000	
D1	1.50000000	sec
D2	0.00344828	sec
D12	0.00002000	sec
TD0	7	

======== CHANNEL f1 ========

NUC1	13C	
P1	13.35	usec
P12	2000.00	usec
PL0	120.00	dB
PL1	-3.00	dB
SFO1	125.7950092	MHz
SP2	2.65	dB
SPNAM2	Crp60comp.4	
SPOAL2	0.500	
SPOFFS2	0.00	Hz

======== CHANNEL f2 ========

CPDPRG2	waltz16	
NUC2	1H	
P3	8.03	usec
P4	16.06	usec
PCPD2	80.00	usec
PL2	3.00	dB
PL12	22.74	dB
SFO2	500.2330014	MHz
SI	32768	
SF	125.7827558	MHz
WDW	EM	
SSB	0	
LB	1.00	Hz
GB	0	
PC	0.80	

MAHWISH/DR.IQBAL/BM-10/CD3OD
ICCBS/U.O.K
HSQC

AVANCE AV-500
LAB NO:118

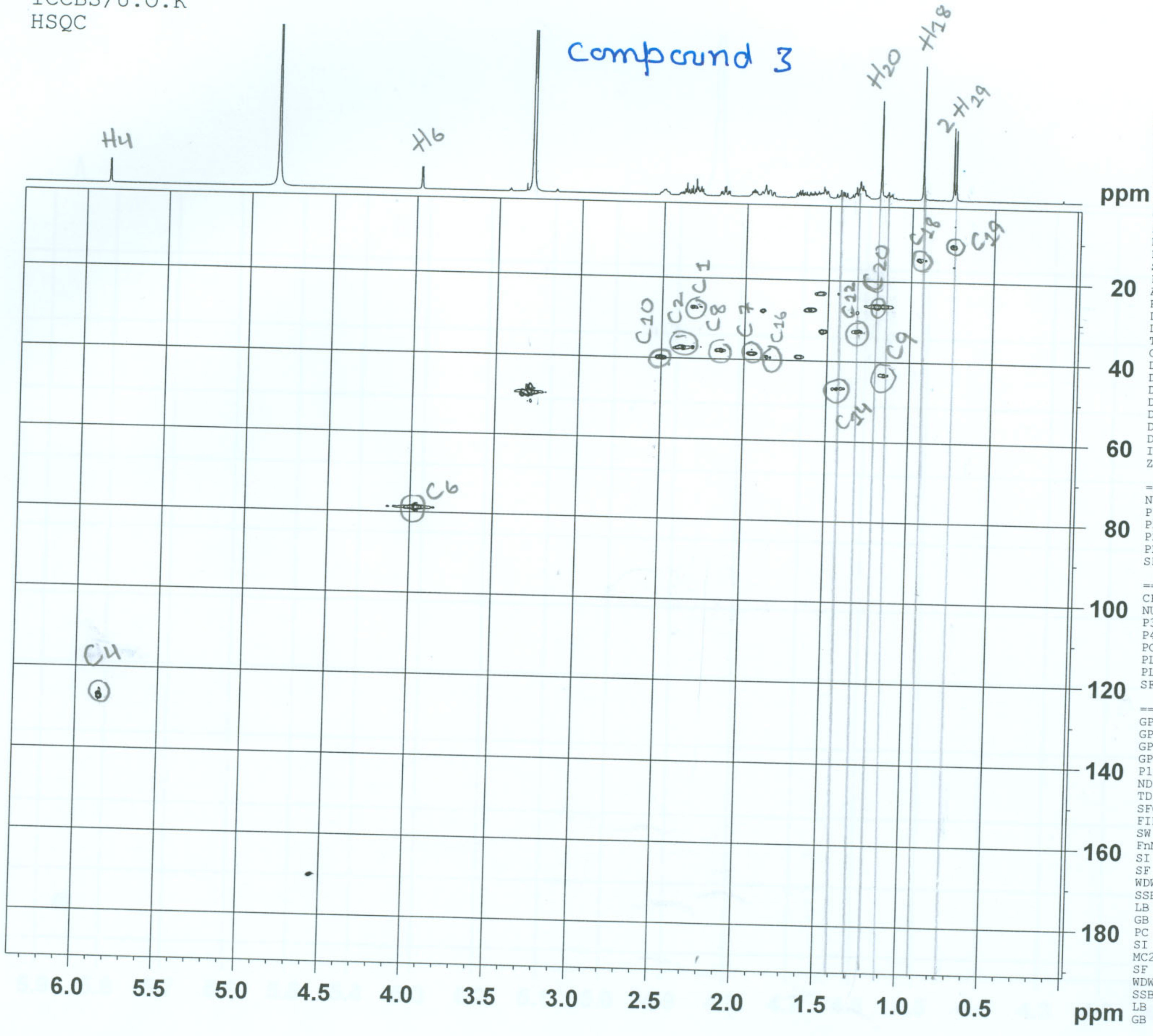

NAME	jun23-15
EXPNO	4
PROCNO	1
Date_	20150623
Time	14.22
INSTRUM	spect
PROBHD	5 mm PABBI 1H/
PULPROG	hsqcetgpsi
TD	1024
SOLVENT	MeOD
NS	32
DS	16
SWH	3205.128 Hz
FIDRES	3.130008 Hz
AQ	0.1599500 sec
RG	18390.4
DW	156.000 usec
DE	6.50 usec
TE	300.6 K
CNST2	145.0000000
D0	0.00000300 sec
D1	1.50000000 sec
D4	0.00172414 sec
D11	0.03000000 sec
D13	0.00000400 sec
D16	0.00020000 sec
D24	0.00110000 sec
IN0	0.00002090 sec
ZGOPTNS	

======== CHANNEL f1 ========

NUC1	1H
P1	8.03 usec
P2	16.07 usec
P28	1000.00 usec
PL1	3.00 dB
SFO1	500.2316007 MHz

======== CHANNEL f2 ========

CPDPRG2	garp
NUC2	13C
P3	13.35 usec
P4	26.70 usec
PCPD2	70.00 usec
PL2	-3.00 dB
PL12	10.00 dB
SFO2	125.7948834 MHz

====== GRADIENT CHANNEL =====

GPNAM1	SINE.100
GPNAM2	SINE.100
GPZ1	80.00 %
GPZ2	20.10 %
P16	1000.00 usec
ND0	2
TD	256
SFO1	125.7949 MHz
FIDRES	93.363388 Hz
SW	190.000 ppm
FnMODE	Echo-Antiecho
SI	1024
SF	500.2300118 MHz
WDW	QSINE
SSB	2
LB	0.00 Hz
GB	0
PC	1.00
SI	1024
MC2	echo-antiecho
SF	125.7827558 MHz
WDW	QSINE
SSB	2
LB	0.00 Hz
GB	0

MAHWISH/DR.IQBAL/BM-10/CD3OD
ICCBS/U.O.K
HMBC

AVANCE AV-500
LAB NO:118

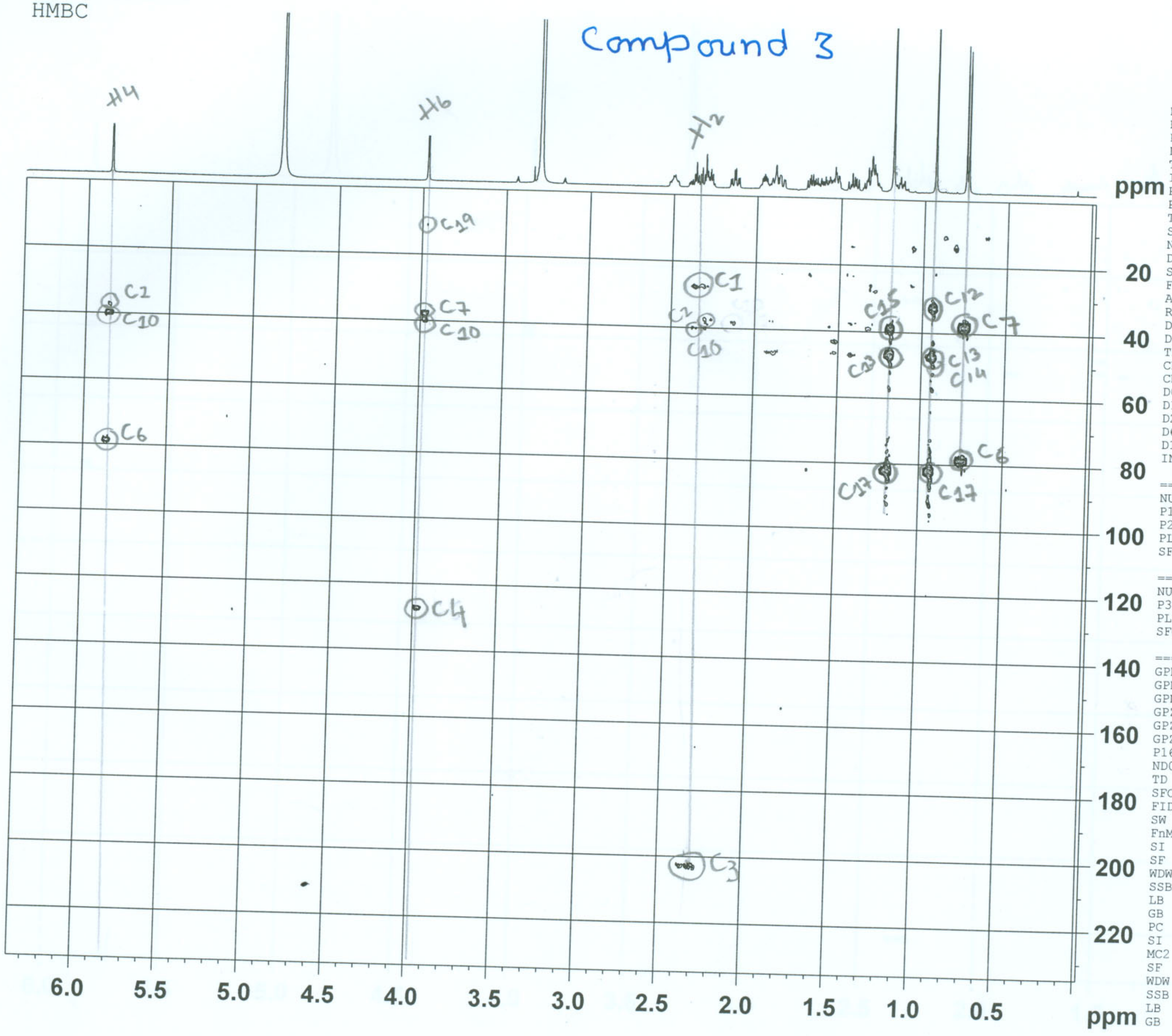

NAME	jun23-15	
EXPNO	5	
PROCNO	1	
Date_	20150623	
Time	18.14	
INSTRUM	spect	
PROBHD	5 mm PABBI 1H/	
PULPROG	hmbcgplpndqf	
TD	2048	
SOLVENT	MeOD	
NS	64	
DS	16	
SWH	3205.128	Hz
FIDRES	1.565004	Hz
AQ	0.3196940	sec
RG	20642.5	
DW	156.000	usec
DE	6.50	usec
TE	298.5	K
CNST2	145.0000000	
CNST13	10.0000000	
D0	0.00000300	sec
D1	2.00000000	sec
D2	0.00344828	sec
D6	0.05000000	sec
D16	0.00020000	sec
IN0	0.00001690	sec

======== CHANNEL f1 ========

NUC1	1H	
P1	8.03	usec
P2	16.06	usec
PL1	3.00	dB
SFO1	500.2316007	MHz

======== CHANNEL f2 ========

NUC2	13C	
P3	13.35	usec
PL2	-3.00	dB
SFO2	125.7974871	MHz

====== GRADIENT CHANNEL =====

GPNAM1	SINE.100	
GPNAM2	SINE.100	
GPNAM3	SINE.100	
GPZ1	50.00	%
GPZ2	30.00	%
GPZ3	40.10	%
P16	1000.00	usec
ND0	2	
TD	256	
SFO1	125.7975	MHz
FIDRES	115.478165	Hz
SW	235.000	ppm
FnMODE	QF	
SI	1024	
SF	500.2300118	MHz
WDW	SINE	
SSB	0	
LB	0.00	Hz
GB	0	
PC	1.00	
SI	512	
MC2	QF	
SF	125.7827558	MHz
WDW	SINE	
SSB	0	
LB	0.00	Hz
GB	0	

MAHWISH/DR.IQBAL/BM-10/CD3OD
ICCBS/U.O.K
COSY

Compound 3

AVANCE AV-500
LAB NO:118

COSY

H4 ↔ H6
H4 ↔ H10
H6 ↔ H7

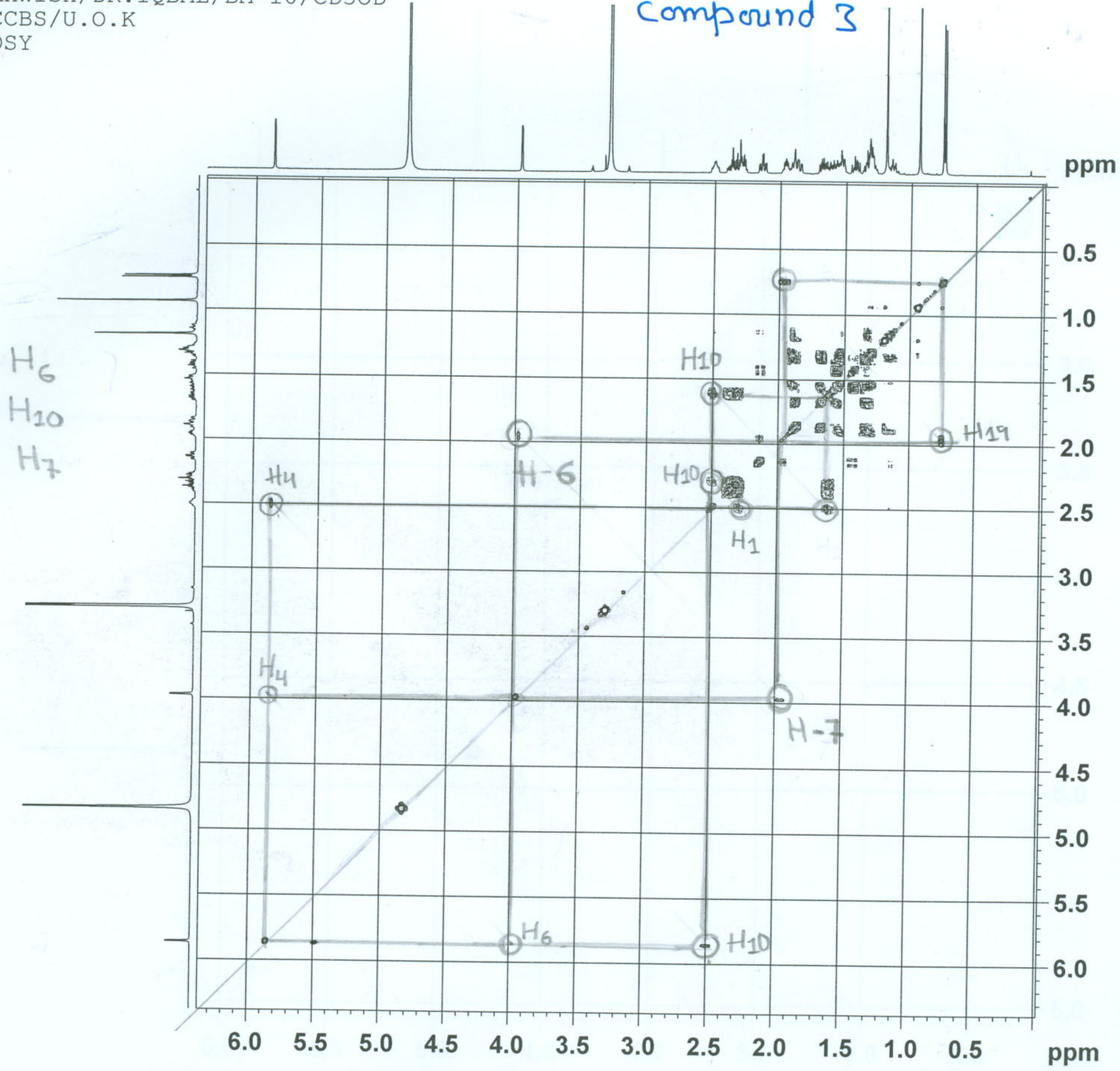

NAME	jun23-15	
EXPNO	2	
PROCNO	1	
Date_	20150623	
Time	9.29	
INSTRUM	spect	
PROBHD	5 mm PABBI 1H/	
PULPROG	cosygpqf	
TD	2048	
SOLVENT	MeOD	
NS	8	
DS	8	
SWH	3205.128	Hz
FIDRES	1.565004	Hz
AQ	0.3196940	sec
RG	256	
DW	156.000	usec
DE	6.50	usec
TE	299.0	K
D0	0.00000300	sec
D1	1.50000000	sec
D13	0.00000400	sec
D16	0.00020000	sec
IN0	0.00031200	sec

======== CHANNEL f1 ========

NUC1	1H	
P0	8.03	usec
P1	8.03	usec
PL1	3.00	dB
SFO1	500.2316007	MHz

====== GRADIENT CHANNEL =====

GPNAM1	SINE.100	
GPZ1	10.00	%
P16	1000.00	usec
ND0	1	
TD	256	
SFO1	500.2316	MHz
FIDRES	12.520031	Hz
SW	6.407	ppm
FnMODE	QF	
SI	1024	
SF	500.2300118	MHz
WDW	QSINE	
SSB	0	
LB	0.00	Hz
GB	0	
PC	1.00	
SI	1024	
MC2	QF	
SF	500.2300118	MHz
WDW	QSINE	
SSB	0	
LB	0.00	Hz
GB	0	

MAHWISH/DR.IQBAL/BM-10/CD3OD
ICCBS/U.O.K
NOESY

Compound 3

AVANCE AV-500
LAB NO:118

NOESY

H6 ⟷ H7
H6 ⟷ H19
H4 ⟷ H6
H4 ⟷ H19
H10 ⟷ H11
H10 ⟷ H8
H10 ⟷ H2

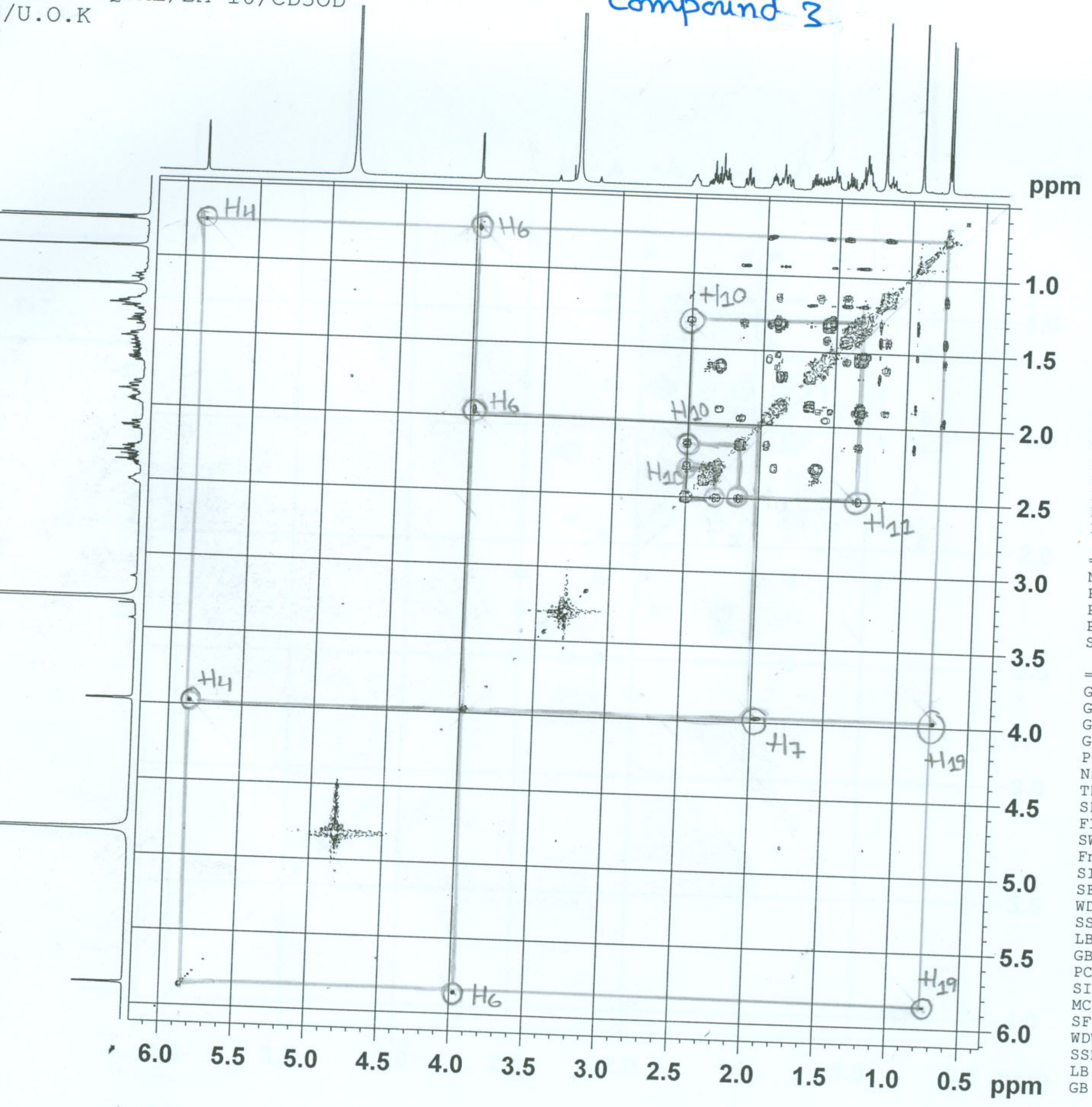

NAME	jun23-15
EXPNO	3
PROCNO	1
Date_	20150623
Time	10.47
INSTRUM	spect
PROBHD	5 mm PABBI 1H/
PULPROG	noesygpph
TD	2048
SOLVENT	MeOD
NS	16
DS	8
SWH	3205.128 Hz
FIDRES	1.565004 Hz
AQ	0.3196940 sec
RG	362
DW	156.000 usec
DE	6.50 usec
TE	299.7 K
D0	0.00014578 sec
D1	2.00000000 sec
D8	0.80000001 sec
D16	0.00020000 sec
IN0	0.00031200 sec

======== CHANNEL f1 ========

NUC1	1H
P1	8.03 usec
P2	16.06 usec
PL1	3.00 dB
SFO1	500.2316007 MHz

====== GRADIENT CHANNEL =====

GPNAM1	SINE.100
GPNAM2	SINE.100
GPZ1	40.00 %
GPZ2	-40.00 %
P16	1000.00 usec
ND0	1
TD	256
SFO1	500.2316 MHz
FIDRES	12.520031 Hz
SW	6.407 ppm
FnMODE	States-TPPI
SI	1024
SF	500.2300118 MHz
WDW	QSINE
SSB	2
LB	0.00 Hz
GB	0
PC	1.00
SI	1024
MC2	States-TPPI
SF	500.2300118 MHz
WDW	QSINE
SSB	2
LB	0.00 Hz
GB	0

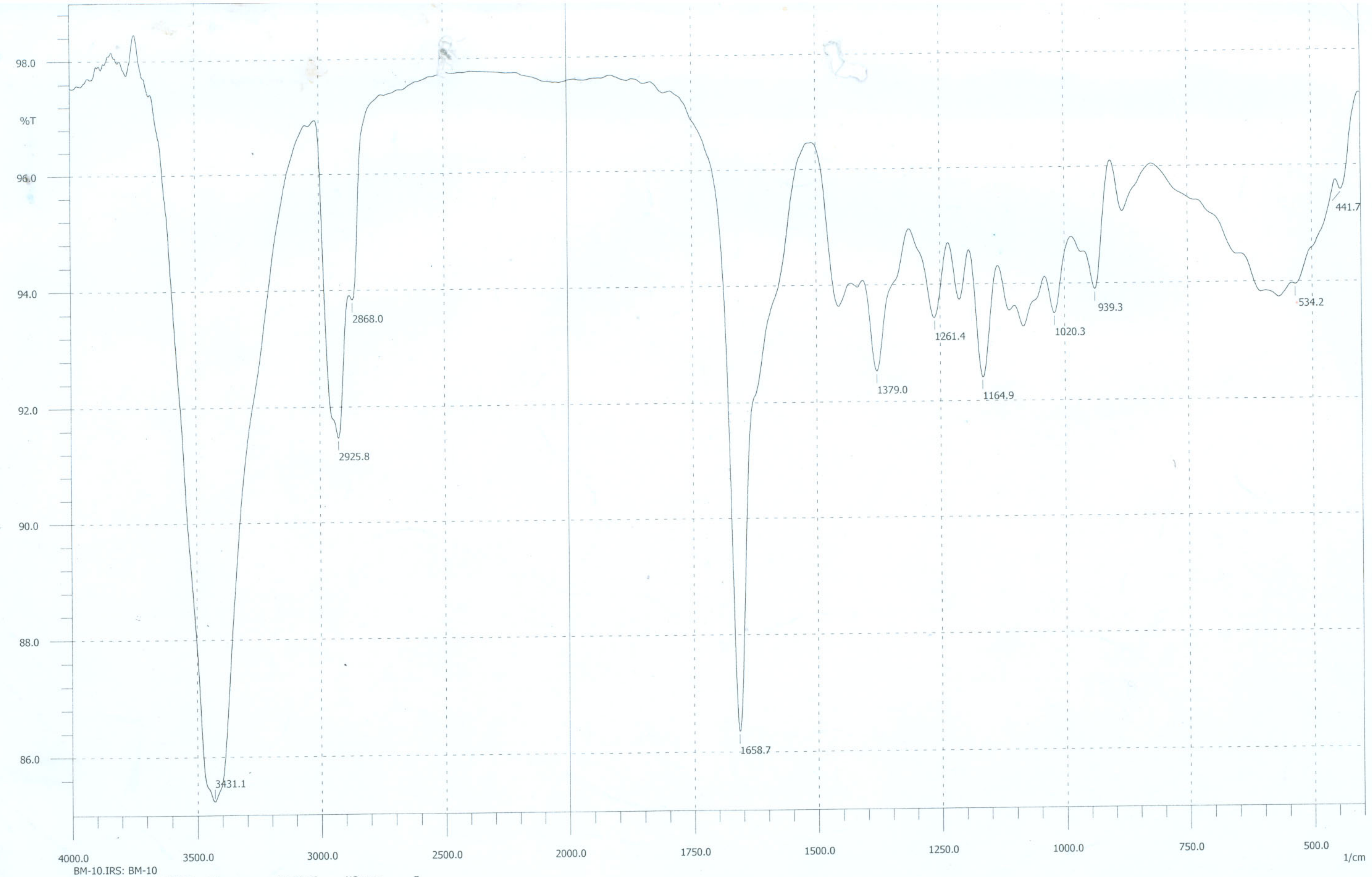

BM-10.IRS: BM-10

Date:	07/27/2015	Time:	11:50:48	NScans:	5		
Type:	HYPER IR	User:	ZUBAIR AHMED	Detector:	standard		
Abscissa:	1/cm	Ordinate:	%T	Apodization:	Happ		
Min:	401.17	Max:	3998.16	Range:	1/cm		
Ndp:	1866	Data Interval:	1.92868	Resolution:	4.0		
Gain:	auto	Aperture:	auto	Mirror Speed:	2.8(low)		

comp - 3

comp- 3

THERMO ELECTRON ~ VISIONpro SOFTWARE V4.10

Operator Name	Zubair Ahmad	Date of Report	7/23/2015
Department	Analytical laboratory#004 TWC	Time of Report	10:04:13AM
Organization	ICCBS.Karachi University.		
Information	Mahwish/Prof.Dr.M.Iqbal		

Scan Graph

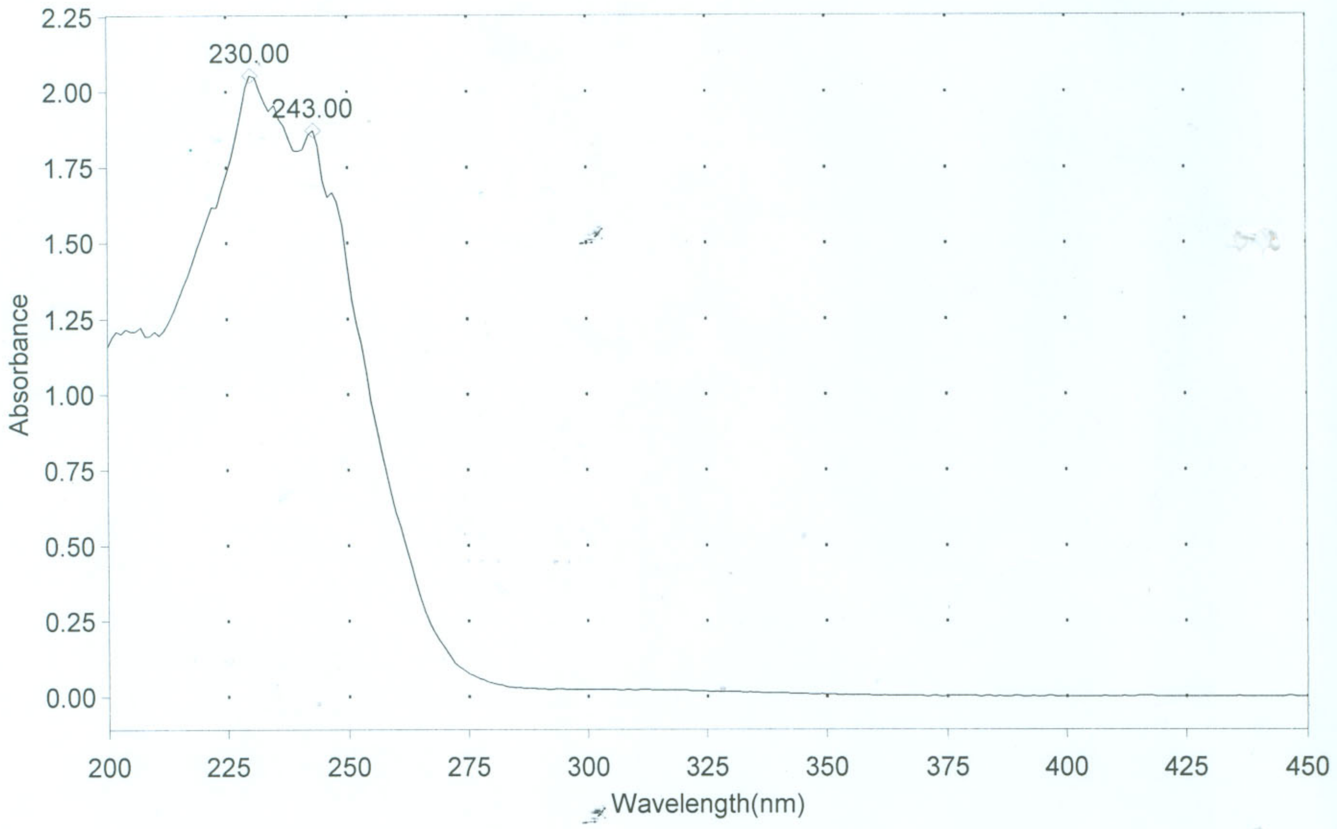

BM-10

Results Table ~~MCB~~- 10.sre,MCB- 10,Cycle01

nm	A
230.00	2.055
243.00	1.870

Peak Pick Method
Find 8 Peaks Above -3.0000 A
Start Wavelength 200.00 nm
Stop Wavelength 450.00 nm
Sort By Wavelength

Sensitivity Auto

2ml ⟶ 0.1ml+2ml
