# Supplementary material for: Biotransformation of a potent anabolic steroid, mibolerone, with Cunninghamella blakesleeana, C. echinulata, and Macrophomina phaseolina, and biological activity evaluation of its metabolites
Source: PLoS One. 2017 Feb 24;12(2):e0171476. doi: 10.1371/journal.pone.0171476 (PMC5325191; doi:10.1371/journal.pone.0171476)
Supplement: S3 Data — (PDF) [file pone.0171476.s003.pdf]

File: C-M-11 Date Run: 05-26-2015 (Time Run: 11:36:45)
Sample: MAHWISH SIDDIQUI /DR. IQBAL
Instrument: JEOL MSRoute
Inlet: My Inlet Ionization mode: EI+

Scan: 17 R.T.: 1.43
Base: m/z 334; 99.7%FS TIC: 11702190 #Ions: 285

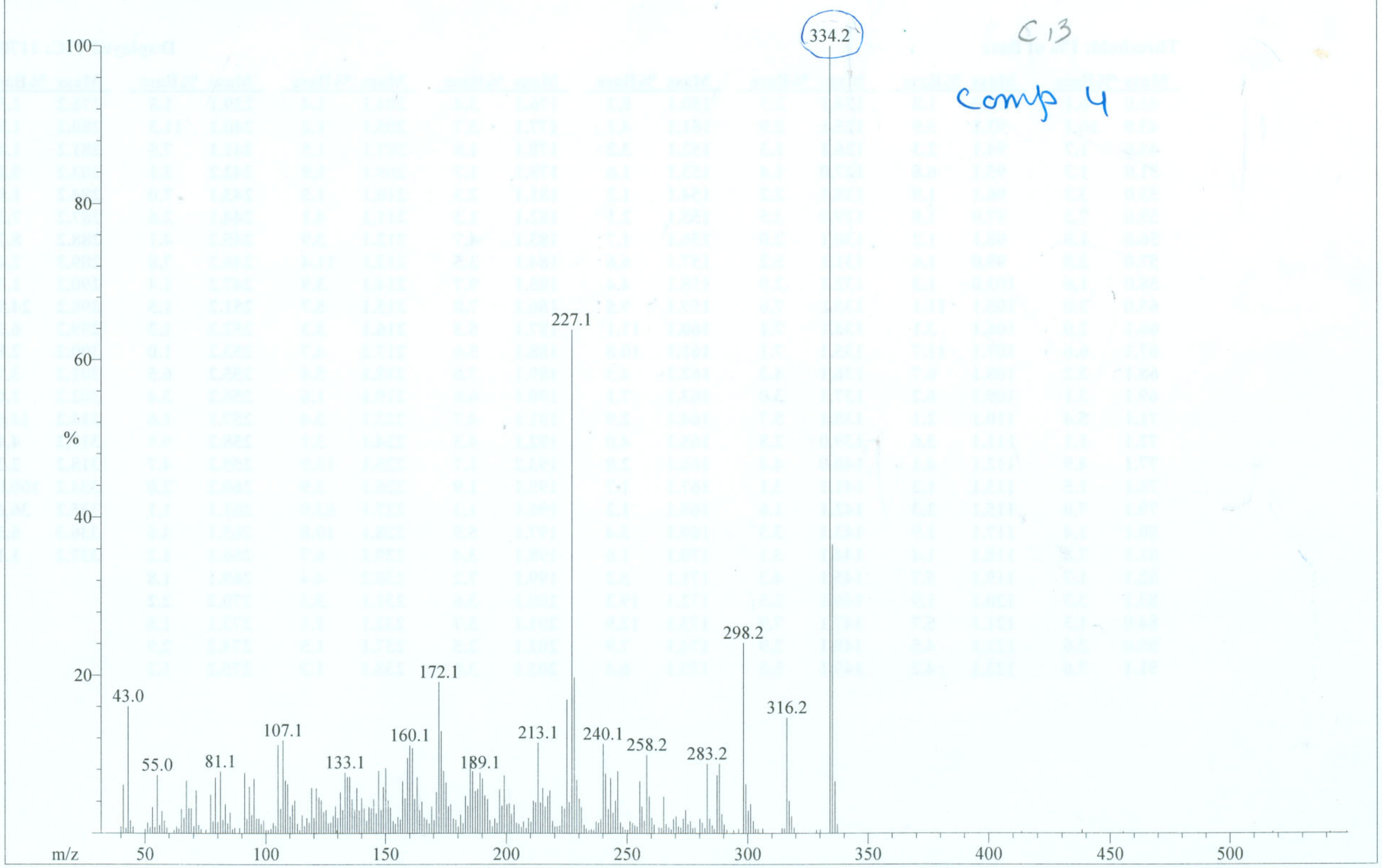

Compound 4

Mass	Relative Intensity	Theoretical Mass	Delta [ppm]	Delta [mmu]	RDB	Composition
215.1436	7.8	215.1436	-0.0	-0.0	6.5	$C_{15} H_{19} O_{1}$
216.1480	4.0	216.1514	-15.7	-3.4	6.0	$C_{15} H_{20} O_{1}$
217.1207	2.4	217.1229	-10.1	-2.2	6.5	$C_{14} H_{17} O_{2}$
217.1579	4.6	217.1592	-6.1	-1.3	5.5	$C_{15} H_{21} O_{1}$
218.1297	3.2	218.1307	-4.5	-1.0	6.0	$C_{14} H_{18} O_{2}$
218.1647	3.5	218.1671	-10.9	-2.4	5.0	$C_{15} H_{22} O_{1}$
223.1139	5.5	223.1123	7.3	1.6	9.5	$C_{16} H_{15} O_{1}$
		223.1182	-19.1	-4.3	0.5	$C_{9} H_{19} O_{6}$
223.1486	2.4	223.1487	-0.4	-0.1	8.5	$C_{17} H_{19}$
224.1196	5.0	224.1201	-2.3	-0.5	9.0	$C_{16} H_{16} O_{1}$
225.1281	31.7	225.1279	0.6	0.1	8.5	$C_{16} H_{17} O_{1}$
226.1330	10.7	226.1358	-12.1	-2.7	8.0	$C_{16} H_{18} O_{1}$
227.1440	100.0	227.1436	1.6	0.4	7.5	$C_{16} H_{19} O_{1}$
228.1495	31.9	228.1514	-8.3	-1.9	7.0	$C_{16} H_{20} O_{1}$
229.1229	2.9	229.1229	0.2	0.0	7.5	$C_{15} H_{17} O_{2}$
229.1566	11.3	229.1592	-11.5	-2.6	6.5	$C_{16} H_{21} O_{1}$
230.1645	7.7	230.1671	-11.2	-2.6	6.0	$C_{16} H_{22} O_{1}$
231.1386	3.5	231.1385	0.5	0.1	6.5	$C_{15} H_{19} O_{2}$
231.1737	3.0	231.1749	-5.2	-1.2	5.5	$C_{16} H_{23} O_{1}$
237.1647	2.6	237.1643	1.8	0.4	8.5	$C_{18} H_{21}$
238.1349	2.5	238.1358	-3.6	-0.9	9.0	$C_{17} H_{18} O_{1}$
239.1421	2.9	239.1436	-6.1	-1.5	8.5	$C_{17} H_{19} O_{1}$
240.1499	28.6	240.1514	-6.3	-1.5	8.0	$C_{17} H_{20} O_{1}$
241.1227	2.5	241.1229	-0.4	-0.1	8.5	$C_{16} H_{17} O_{2}$
241.1557	14.7	241.1592	-14.8	-3.6	7.5	$C_{17} H_{21} O_{1}$
242.1290	2.6	242.1307	-7.1	-1.7	8.0	$C_{16} H_{18} O_{2}$
242.1650	5.7	242.1671	-8.7	-2.1	7.0	$C_{17} H_{22} O_{1}$
243.1379	13.2	243.1385	-2.6	-0.6	7.5	$C_{16} H_{19} O_{2}$
244.1440	5.2	244.1463	-9.6	-2.3	7.0	$C_{16} H_{20} O_{2}$
245.1546	6.6	245.1542	1.9	0.5	6.5	$C_{16} H_{21} O_{2}$
246.1615	9.2	246.1620	-2.0	-0.5	6.0	$C_{16} H_{22} O_{2}$
247.1652	3.2	247.1698	-18.4	-4.6	5.5	$C_{16} H_{23} O_{2}$
251.1423	2.2	251.1436	-5.2	-1.3	9.5	$C_{18} H_{19} O_{1}$
255.1744	7.7	255.1749	-2.0	-0.5	7.5	$C_{18} H_{23} O_{1}$
256.1794	2.9	256.1827	-13.0	-3.3	7.0	$C_{18} H_{24} O_{1}$
258.1629	11.0	258.1620	3.4	0.9	7.0	$C_{17} H_{22} O_{2}$
259.1663	7.0	259.1698	-13.5	-3.5	6.5	$C_{17} H_{23} O_{2}$
260.1725	2.4	260.1776	-19.6	-5.1	6.0	$C_{17} H_{24} O_{2}$
265.1585	9.9	265.1592	-3.0	-0.8	9.5	$C_{19} H_{21} O_{1}$
266.1604	3.0					
267.1719	2.3	267.1749	-11.1	-3.0	8.5	$C_{19} H_{23} O_{1}$
270.1967	3.1	270.1984	-6.2	-1.7	7.0	$C_{19} H_{26} O_{1}$
273.1829	2.3	273.1855	-9.5	-2.6	6.5	$C_{18} H_{25} O_{2}$
274.1918	3.2	274.1933	-5.3	-1.5	6.0	$C_{18} H_{26} O_{2}$
280.1849	3.6	280.1827	7.7	2.2	9.0	$C_{20} H_{24} O_{1}$
		280.1886	-13.2	-3.7	0.0	$C_{13} H_{28} O_{6}$
283.1691	13.0	283.1698	-2.5	-0.7	8.5	$C_{19} H_{23} O_{2}$
284.1778	4.2	284.1776	0.7	0.2	8.0	$C_{19} H_{24} O_{2}$
287.2019	9.0	287.2011	2.7	0.8	6.5	$C_{19} H_{27} O_{2}$
288.2096	12.2	288.2089	2.4	0.7	6.0	$C_{19} H_{28} O_{2}$
289.2143	4.2	289.2168	-8.5	-2.4	5.5	$C_{19} H_{29} O_{2}$
298.1955	21.2	298.1933	7.6	2.3	8.0	$C_{20} H_{26} O_{2}$
299.1983	7.8	299.2011	-9.3	-2.8	7.5	$C_{20} H_{27} O_{2}$
300.2061	5.0	300.2089	-9.6	-2.9	7.0	$C_{20} H_{28} O_{2}$
301.1829	4.1	301.1804	8.4	2.5	7.5	$C_{19} H_{25} O_{3}$
301.2141	2.2	301.2168	-8.7	-2.6	6.5	$C_{20} H_{29} O_{2}$
316.2038	16.4	316.2038	-0.3	-0.1	7.0	$C_{20} H_{28} O_{3}$
317.2072	7.7	317.2117	-14.2	-4.5	6.5	$C_{20} H_{29} O_{3}$
318.2140	4.2	318.2195	-17.2	-5.5	6.0	$C_{20} H_{30} O_{3}$
334.2159	50.8	334.2144	4.3	1.4	6.0	$C_{20} H_{30} O_{4}$
335.2243	30.7	335.2222	6.2	2.1	5.5	$C_{20} H_{31} O_{4}$
336.2299	8.4	336.2301	-0.5	-0.2	5.0	$C_{20} H_{32} O_{4}$

HREI-MS showed m/z at 334.2159 [$M^{+\cdot}$; calcd. 334.2144]

Compound 4

MAHWISH/DR.IQBAL/CM 11
1H/

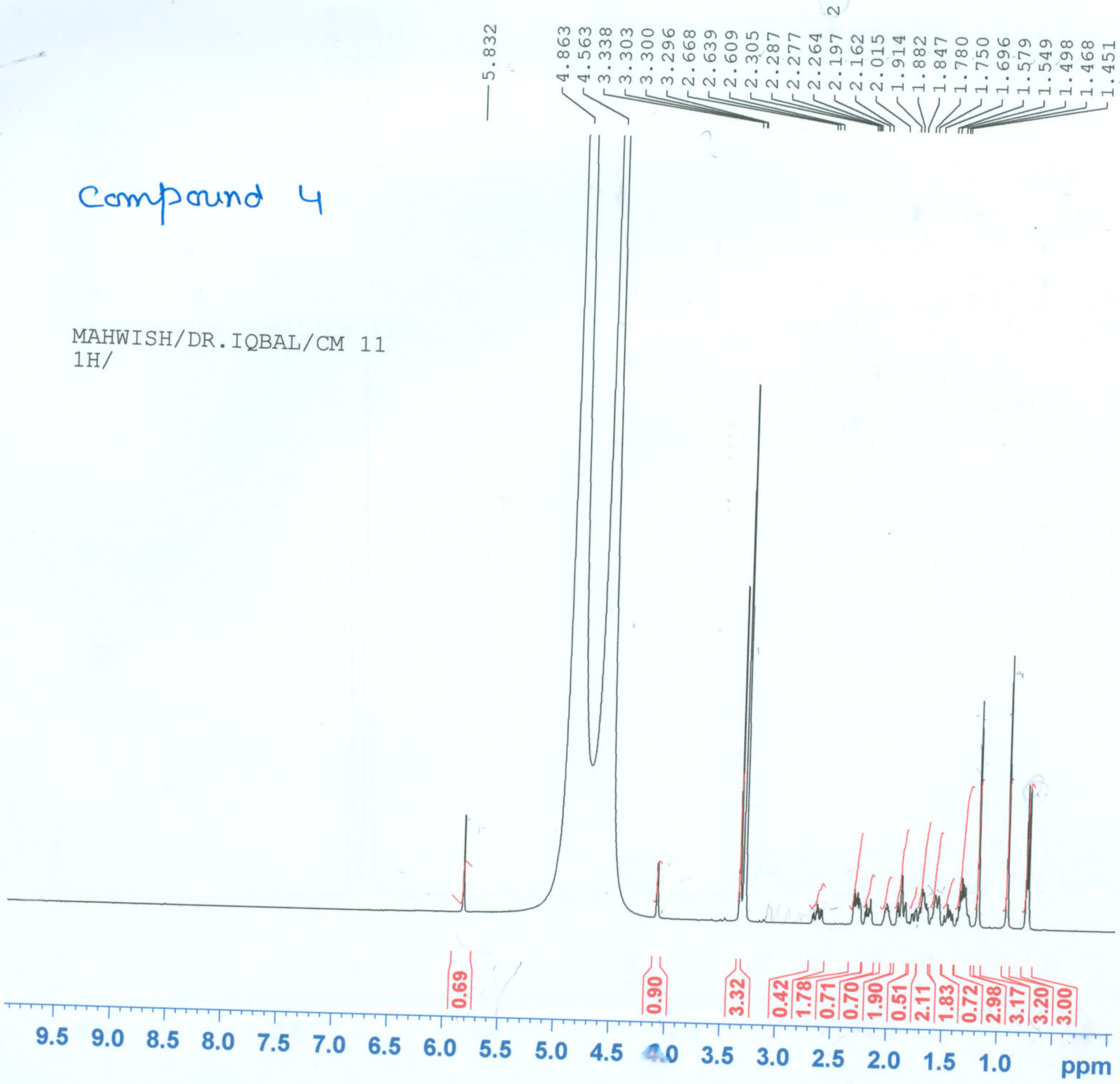

AVANCE AV-400 MHz
Lab # 115

NAME	may25-15
EXPNO	10
PROCNO	1
Date_	20150525
Time	12.50
INSTRUM	spect
PROBHD	5 mm SEI 1H-13
PULPROG	zg30
TD	32768
SOLVENT	MeOD
NS	128
DS	0
SWH	8012.820 Hz
FIDRES	0.244532 Hz
AQ	2.0447731 sec
RG	28.5
DW	62.400 usec
DE	6.50 usec
TE	300.0 K
D1	2.00000000 sec
TD0	1

======== CHANNEL f1 ========

NUC1	1H
P1	10.80 usec
PL1	3.00 dB
SFO1	400.0332002 MHz
SI	16384
SF	400.0300087 MHz
WDW	EM
SSB	0
LB	0.30 Hz
GB	0
PC	1.00

MAHWISH/DR.IQBAL/C-M-11/CD3OD
ICCBS/U.O.K
B.B

Compound 4

AVANCE AV-500
LAB NO: 109B

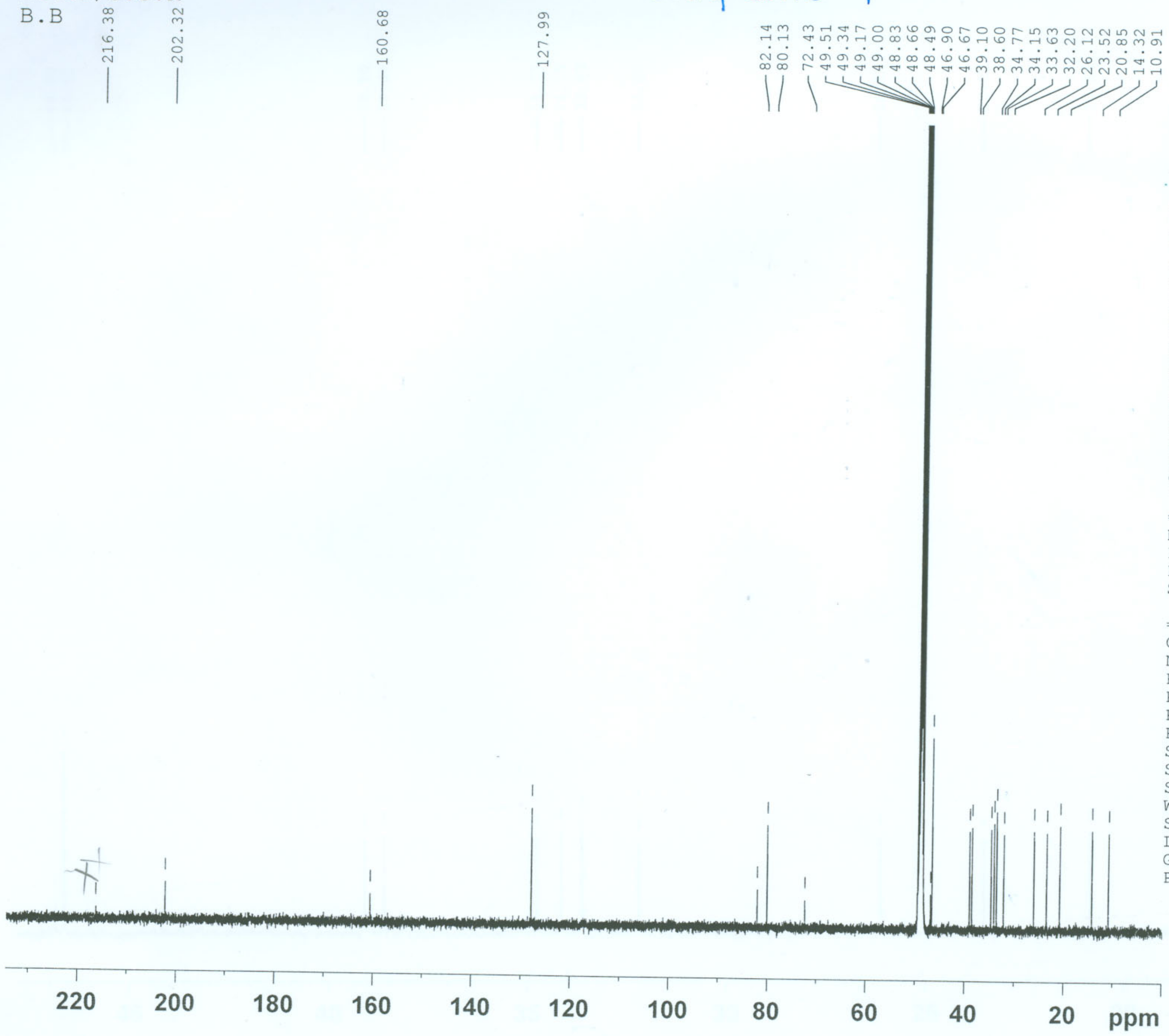

NAME	may29-15	
EXPNO	16	
PROCNO	1	
Date_	20150530	
Time	11.13	
INSTRUM	spect	
PROBHD	5 mm BBI 1H/D-	
PULPROG	zgpg	
TD	32768	
SOLVENT	MeOD	
NS	20480	
DS	4	
SWH	29498.525	Hz
FIDRES	0.900224	Hz
AQ	0.5554845	sec
RG	32768	
DW	16.950	usec
DE	6.50	usec
TE	297.9	K
D1	2.00000000	sec
D11	0.03000000	sec
TD0	20	

======== CHANNEL f1 ========

NUC1	13C	
P1	12.00	usec
PL1	-3.00	dB
SFO1	125.7723529	MHz

======== CHANNEL f2 ========

CPDPRG2	waltz16	
NUC2	1H	
PCPD2	80.00	usec
PL2	-1.00	dB
PL12	19.20	dB
PL13	22.00	dB
SFO2	500.1325007	MHz
SI	32768	
SF	125.7576112	MHz
WDW	EM	
SSB	0	
LB	1.00	Hz
GB	0	
PC	1.00	

MAHWISH/DR.IQBAL/C-M-11/CD3OD
ICCBS/U.O.K
DEPT90

Compound 4

AVANCE AV-500
LAB NO: 109B

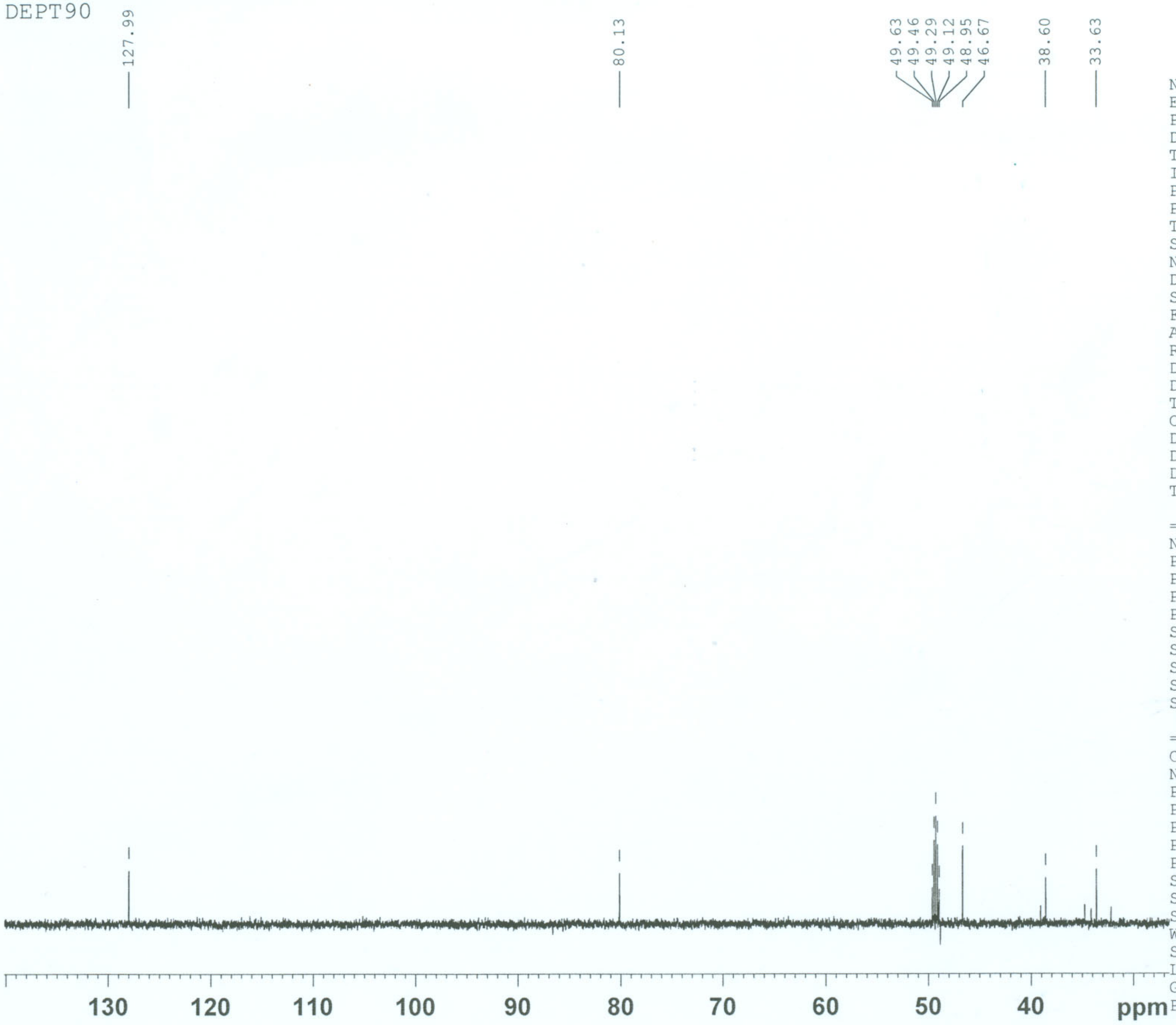

NAME may29-15
EXPNO 18
PROCNO 1
Date_ 20150531
Time 9.39
INSTRUM spect
PROBHD 5 mm BBI 1H/D-
PULPROG deptsp90
TD 32768
SOLVENT MeOD
NS 4864
DS 2
SWH 23809.523 Hz
FIDRES 0.726609 Hz
AQ 0.6881990 sec
RG 32768
DW 21.000 usec
DE 6.50 usec
TE 297.8 K
CNST2 145.0000000
D1 1.50000000 sec
D2 0.00344828 sec
D12 0.00002000 sec
TD0 6

======== CHANNEL f1 ========
NUC1 13C
P1 12.00 usec
P12 2000.00 usec
PL0 120.00 dB
PL1 -3.00 dB
SFO1 125.7697360 MHz
SP2 3.58 dB
SPNAM2 Crp60comp.4
SPOAL2 0.500
SPOFFS2 0.00 Hz

======== CHANNEL f2 ========
CPDPRG2 waltz16
NUC2 1H
P3 8.00 usec
P4 16.00 usec
PCPD2 80.00 usec
PL2 -1.00 dB
PL12 19.20 dB
SFO2 500.1335009 MHz
SI 32768
SF 125.7576112 MHz
WDW EM
SSB 0
LB 1.00 Hz
GB 0
PC 1.40

MAHWISH/DR.IQBAL/C-M-11/CD3OD
ICCBS/U.O.K
DEPT135

Compound - 4

AVANCE AV-500
LAB NO: 109B

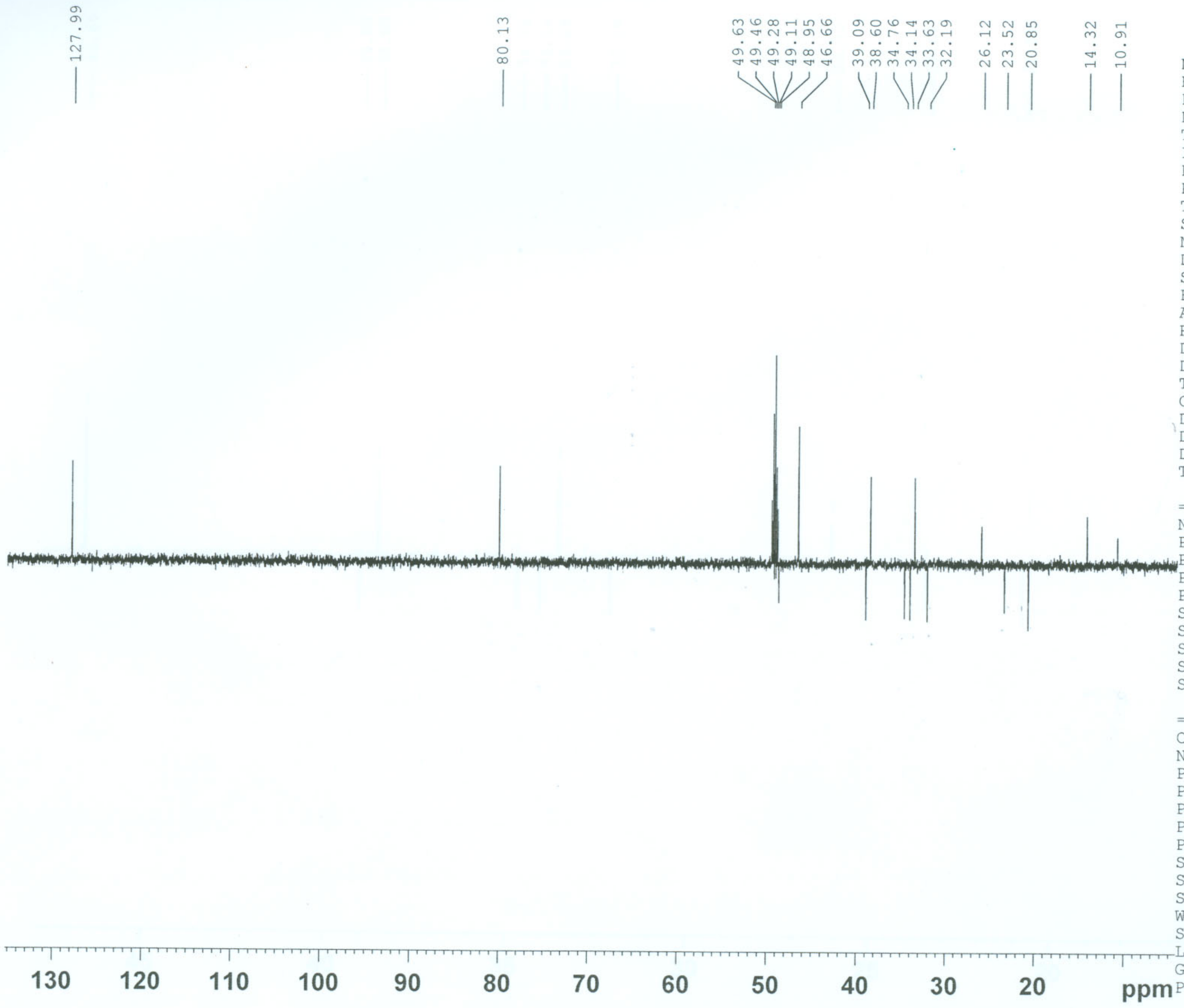

NAME	may29-15
EXPNO	17
PROCNO	1
Date_	20150531
Time	2.01
INSTRUM	spect
PROBHD	5 mm BBI 1H/D-
PULPROG	deptsp135
TD	32768
SOLVENT	MeOD
NS	12288
DS	2
SWH	23809.523 Hz
FIDRES	0.726609 Hz
AQ	0.6881990 sec
RG	32768
DW	21.000 usec
DE	6.50 usec
TE	297.8 K
CNST2	145.0000000
D1	1.50000000 sec
D2	0.00344828 sec
D12	0.00002000 sec
TD0	12
======== CHANNEL f1 ========	
NUC1	13C
P1	12.00 usec
P12	2000.00 usec
PL0	120.00 dB
PL1	-3.00 dB
SFO1	125.7697360 MHz
SP2	3.58 dB
SPNAM2	Crp60comp.4
SPOAL2	0.500
SPOFFS2	0.00 Hz
======== CHANNEL f2 ========	
CPDPRG2	waltz16
NUC2	1H
P3	8.00 usec
P4	16.00 usec
PCPD2	80.00 usec
PL2	-1.00 dB
PL12	19.20 dB
SFO2	500.1335009 MHz
SI	16384
SF	125.7576112 MHz
WDW	EM
SSB	0
LB	1.00 Hz
GB	0
PC	1.00

Comp 4

MAHWISH/DR.IQBAL/C-M-11/CD3OD
ICCBS/U.O.K

HSQC

AVANCE AV-500
LAB NO: 109B

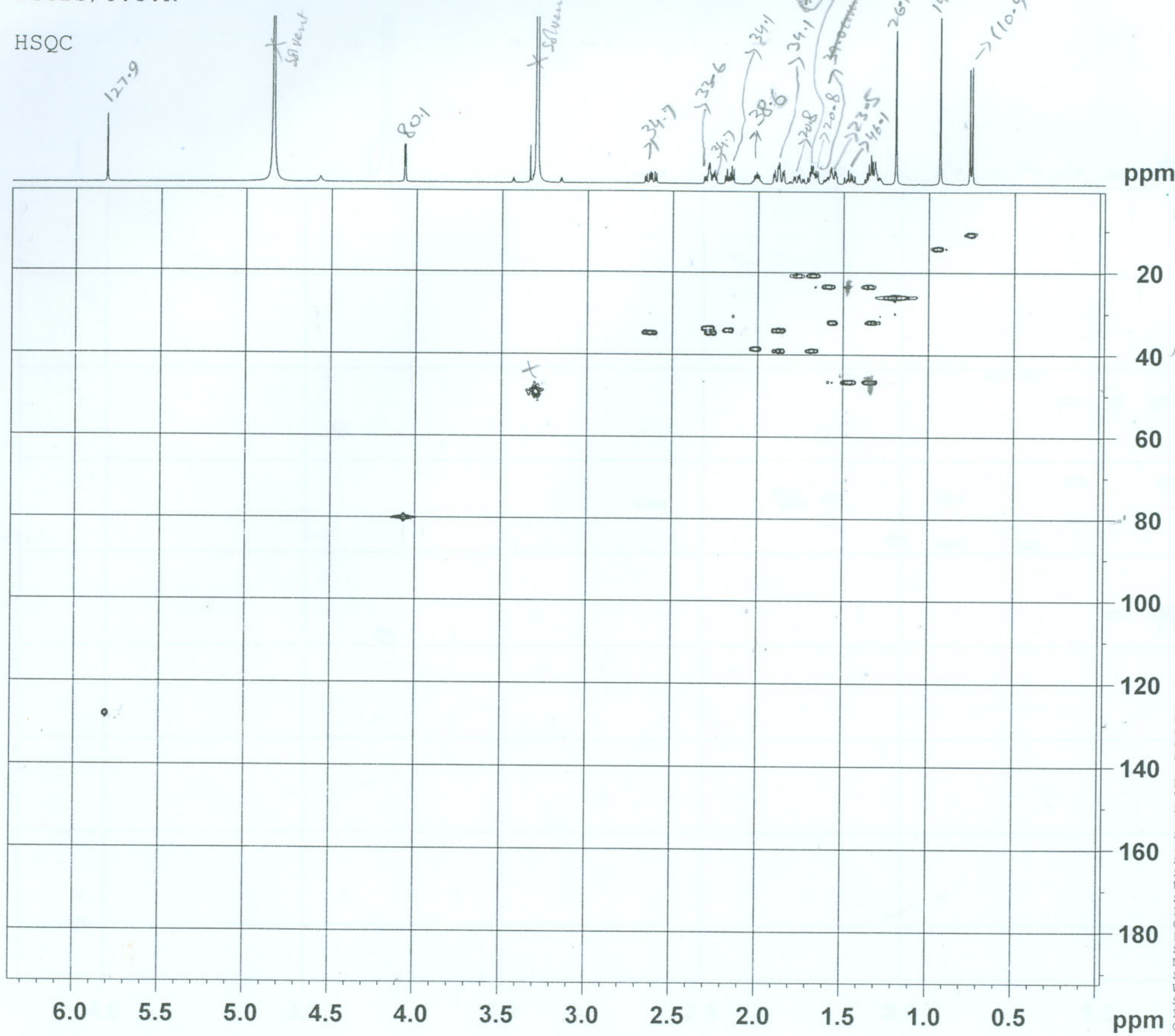


```
NAME          may29-15
EXPNO               14
PROCNO               1
Date_         20150529
Time             19.48
INSTRUM          spect
PROBHD   5 mm BBI 1H/D-
PULPROG     hsqcetgpsi
TD                1024
SOLVENT           MeOD
NS                  32
DS                   8
SWH           3205.128 Hz
FIDRES        3.130008 Hz
AQ           0.1599500 sec
RG             23170.5
DW             156.000 usec
DE                6.50 usec
TE               298.2 K
CNST2      145.0000000
D0          0.00000300 sec
D1          1.50000000 sec
D4          0.00172414 sec
D11         0.03000000 sec
D13         0.00000400 sec
D16         0.00020000 sec
D24         0.00110000 sec
IN0         0.00002070 sec
ZGOPTNS

======== CHANNEL f1 ========
NUC1                1H
P1                8.00 usec
P2               16.00 usec
P28            1000.00 usec
PL1              -1.00 dB
SFO1       500.1316004 MHz

======== CHANNEL f2 ========
CPDPRG2           garp
NUC2               13C
P3               12.00 usec
P4               24.00 usec
PCPD2            65.00 usec
PL2              -3.00 dB
PL12             11.67 dB
SFO2       125.7697360 MHz

====== GRADIENT CHANNEL =====
GPNAM1        SINE.100
GPNAM2        SINE.100
GPZ1             80.00 %
GPZ2             20.10 %
P16            1000.00 usec
ND0                  2
TD                 256
SFO1          125.7697 MHz
FIDRES       94.327301 Hz
SW             192.000 ppm
FnMODE    Echo-Antiecho
SI                1024
SF         500.1300158 MHz
WDW              QSINE
SSB                  2
LB                0.00 Hz
GB                   0
PC                4.00
SI                1024
MC2      echo-antiecho
SF         125.7576112 MHz
WDW              QSINE
SSB                  2
LB                0.00 Hz
GB                   0
```

MAHWISH/DR.IQBAL/C-M-11/CD3OD
ICCBS/U.O.K

HMBC

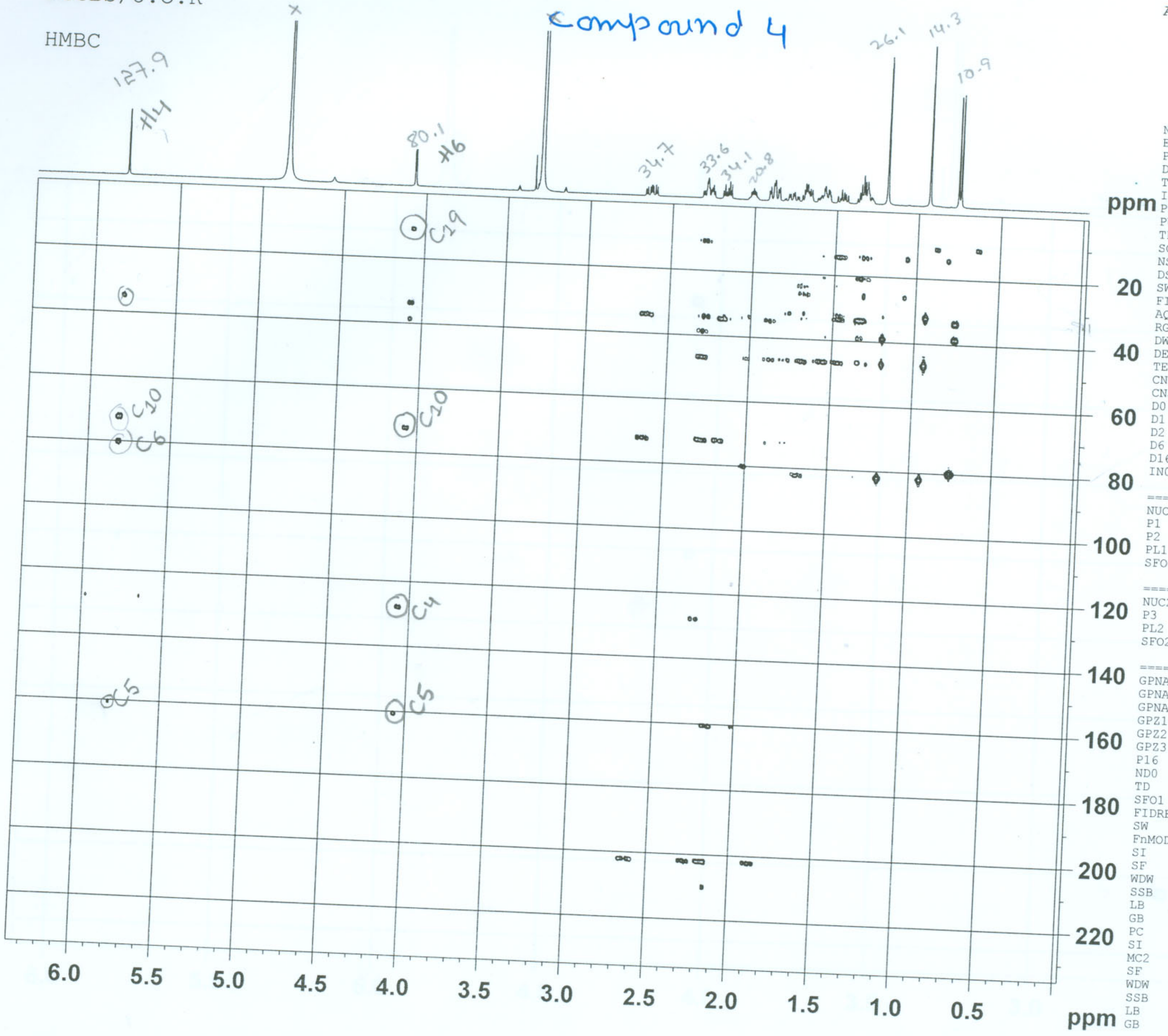

AVANCE AV-500
LAB NO: 109B

NAME	may29-15
EXPNO	15
PROCNO	1
Date_	20150529
Time	23.40
INSTRUM	spect
PROBHD	5 mm BBI 1H/D-
PULPROG	hmbcgplpndqf
TD	2048
SOLVENT	MeOD
NS	64
DS	8
SWH	3205.128 Hz
FIDRES	1.565004 Hz
AQ	0.3196940 sec
RG	20642.5
DW	156.000 usec
DE	6.50 usec
TE	298.3 K
CNST2	145.0000000
CNST13	10.0000000
D0	0.00000300 sec
D1	2.00000000 sec
D2	0.00344828 sec
D6	0.05000000 sec
D16	0.00020000 sec
IN0	0.00001690 sec

======== CHANNEL f1 ========

NUC1	1H
P1	8.00 usec
P2	16.00 usec
PL1	-1.00 dB
SFO1	500.1316004 MHz

======== CHANNEL f2 ========

NUC2	13C
P3	12.00 usec
PL2	-3.00 dB
SFO2	125.7723769 MHz

====== GRADIENT CHANNEL =====

GPNAM1	SINE.100
GPNAM2	SINE.100
GPNAM3	SINE.100
GPZ1	50.00 %
GPZ2	30.00 %
GPZ3	40.10 %
P16	1000.00 usec
ND0	2
TD	256
SFO1	125.7724 MHz
FIDRES	115.455109 Hz
SW	235.000 ppm
FnMODE	QF
SI	1024
SF	500.1300158 MHz
WDW	SINE
SSB	0
LB	0.00 Hz
GB	0
PC	4.00
SI	1024
MC2	QF
SF	125.7576112 MHz
WDW	SINE
SSB	0
LB	0.00 Hz
GB	0

MAHWISH/DR.IQBAL/C-M-11/CD3OD
ICCBS/U.O.K

COSY

AVANCE AV-500
LAB NO: 109B

comp - 4

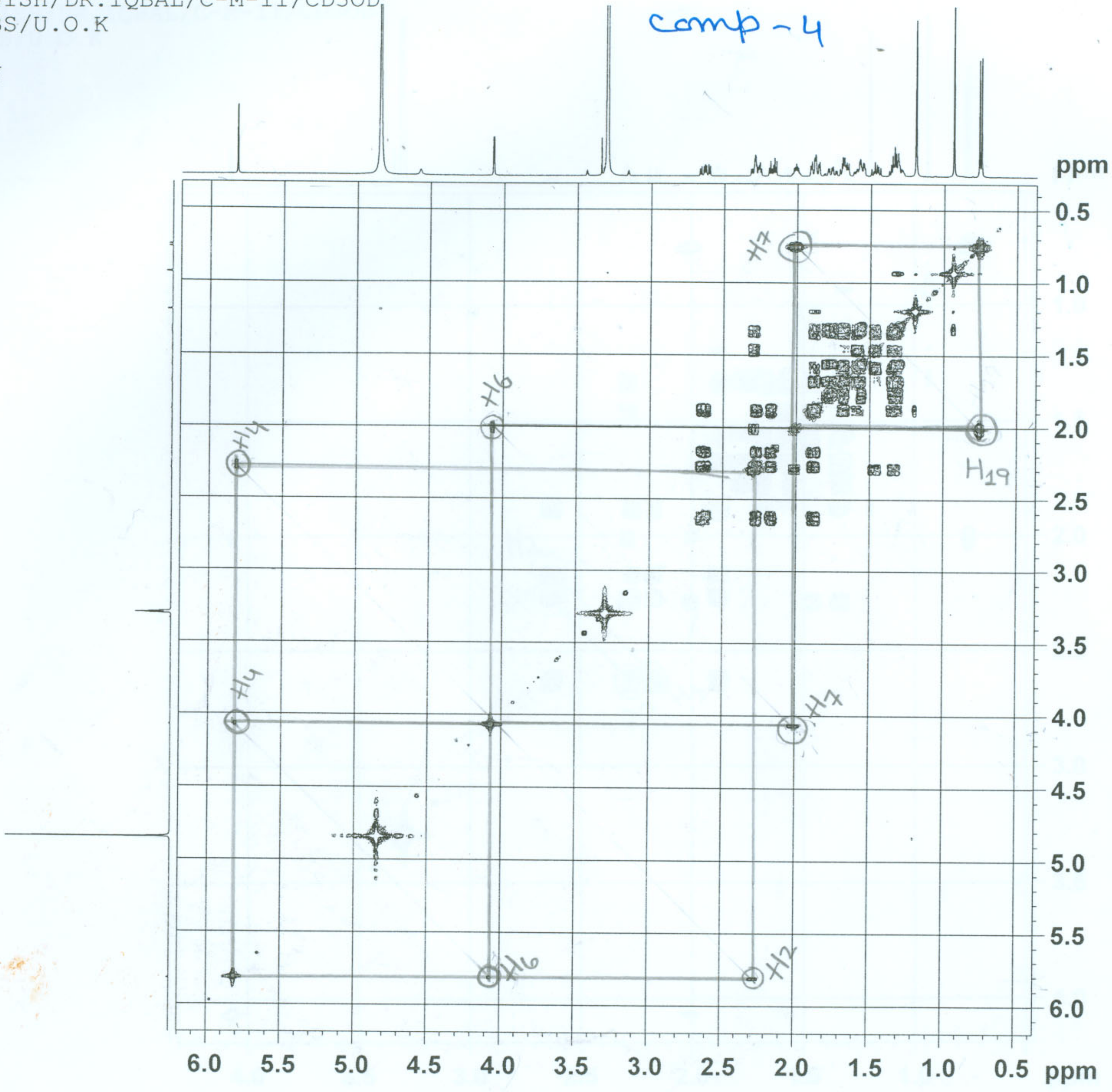

NAME	may29-15
EXPNO	12
PROCNO	1
Date_	20150529
Time	15.08
INSTRUM	spect
PROBHD	5 mm BBI 1H/D-
PULPROG	cosygpqf
TD	2048
SOLVENT	MeOD
NS	8
DS	8
SWH	3205.128 Hz
FIDRES	1.565004 Hz
AQ	0.3196940 sec
RG	574.7
DW	156.000 usec
DE	6.50 usec
TE	298.4 K
D0	0.00000300 sec
D1	1.50000000 sec
D13	0.00000400 sec
D16	0.00020000 sec
IN0	0.00031200 sec

======== CHANNEL f1 ========

NUC1	1H
P0	8.00 usec
P1	8.00 usec
PL1	-1.00 dB
SFO1	500.1316004 MHz

====== GRADIENT CHANNEL =====

GPNAM1	SINE.100
GPZ1	10.00 %
P16	1000.00 usec
ND0	1
TD	256
SFO1	500.1316 MHz
FIDRES	12.520031 Hz
SW	6.409 ppm
FnMODE	QF
SI	1024
SF	500.1300158 MHz
WDW	SINE
SSB	0
LB	0.00 Hz
GB	0
PC	4.00
SI	1024
MC2	QF
SF	500.1300158 MHz
WDW	SINE
SSB	0
LB	0.00 Hz
GB	0

MAHWISH/DR.IQBAL/C-M-11/CD3OD
ICCBS/U.O.K
NOESY

AVANCE AV-500
LAB NO: 109B

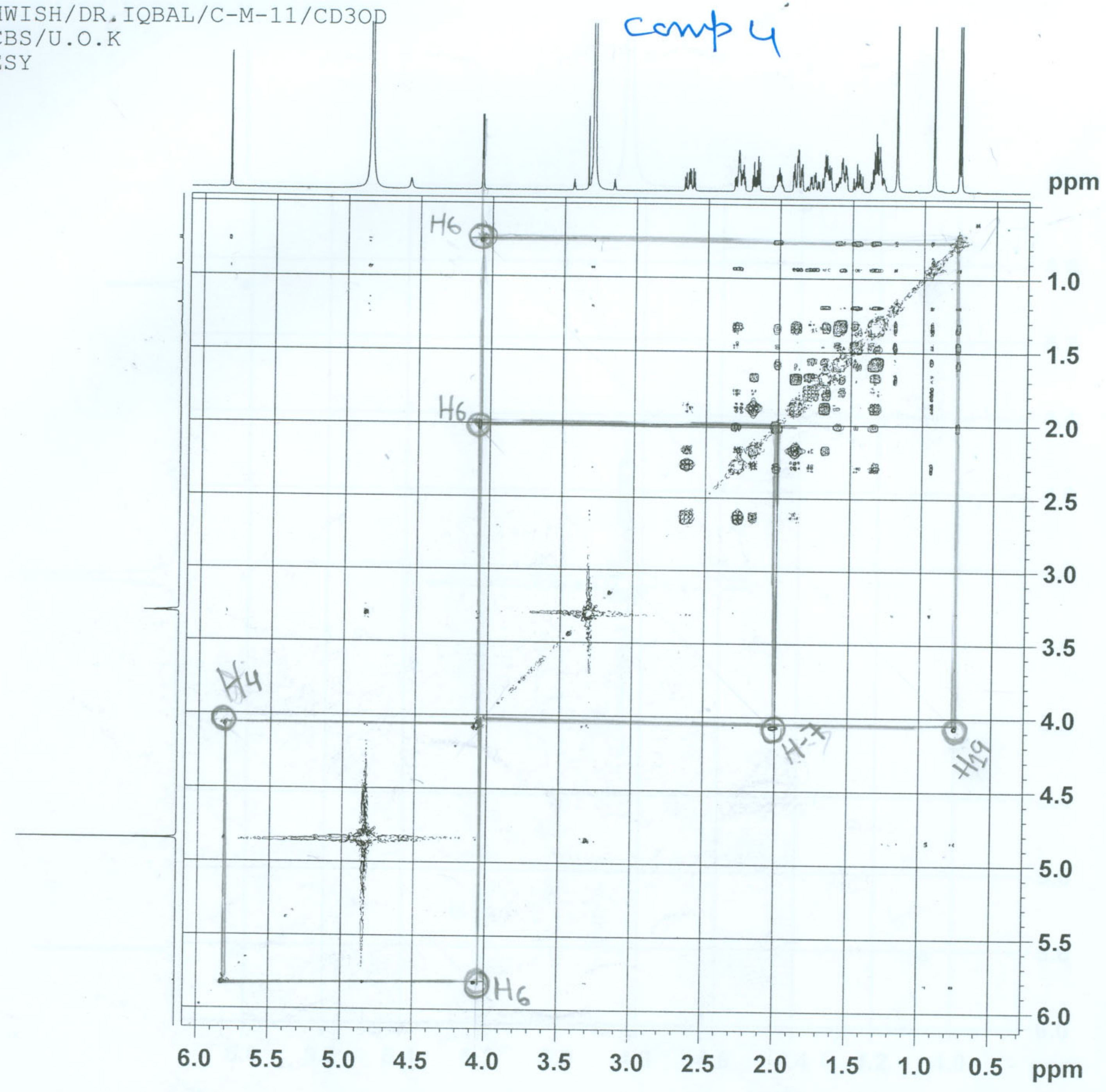

NAME	may29-15
EXPNO	13
PROCNO	1
Date_	20150529
Time	16.13
INSTRUM	spect
PROBHD	5 mm BBI 1H/D-
PULPROG	noesygpph
TD	2048
SOLVENT	MeOD
NS	16
DS	2
SWH	3205.128 Hz
FIDRES	1.565004 Hz
AQ	0.3196940 sec
RG	1290.2
DW	156.000 usec
DE	6.50 usec
TE	298.4 K
D0	0.00014581 sec
D1	2.00000000 sec
D8	0.80000001 sec
D16	0.00020000 sec
IN0	0.00031200 sec

======== CHANNEL f1 ========

NUC1	1H
P1	8.00 usec
P2	16.00 usec
PL1	-1.00 dB
SFO1	500.1316004 MHz

====== GRADIENT CHANNEL =====

GPNAM1	SINE.100
GPZ1	40.00 %
P16	1000.00 usec
ND0	1
TD	256
SFO1	500.1316 MHz
FIDRES	12.520031 Hz
SW	6.409 ppm
FnMODE	States-TPPI
SI	1024
SF	500.1300158 MHz
WDW	QSINE
SSB	2
LB	0.00 Hz
GB	0
PC	4.00
SI	1024
MC2	States-TPPI
SF	500.1300158 MHz
WDW	QSINE
SSB	2
LB	0.00 Hz
GB	0

MAHWISH/DR.IQBAL/C-M-11/C3D6O

AVANCE AV-500
LAB NO: 109B

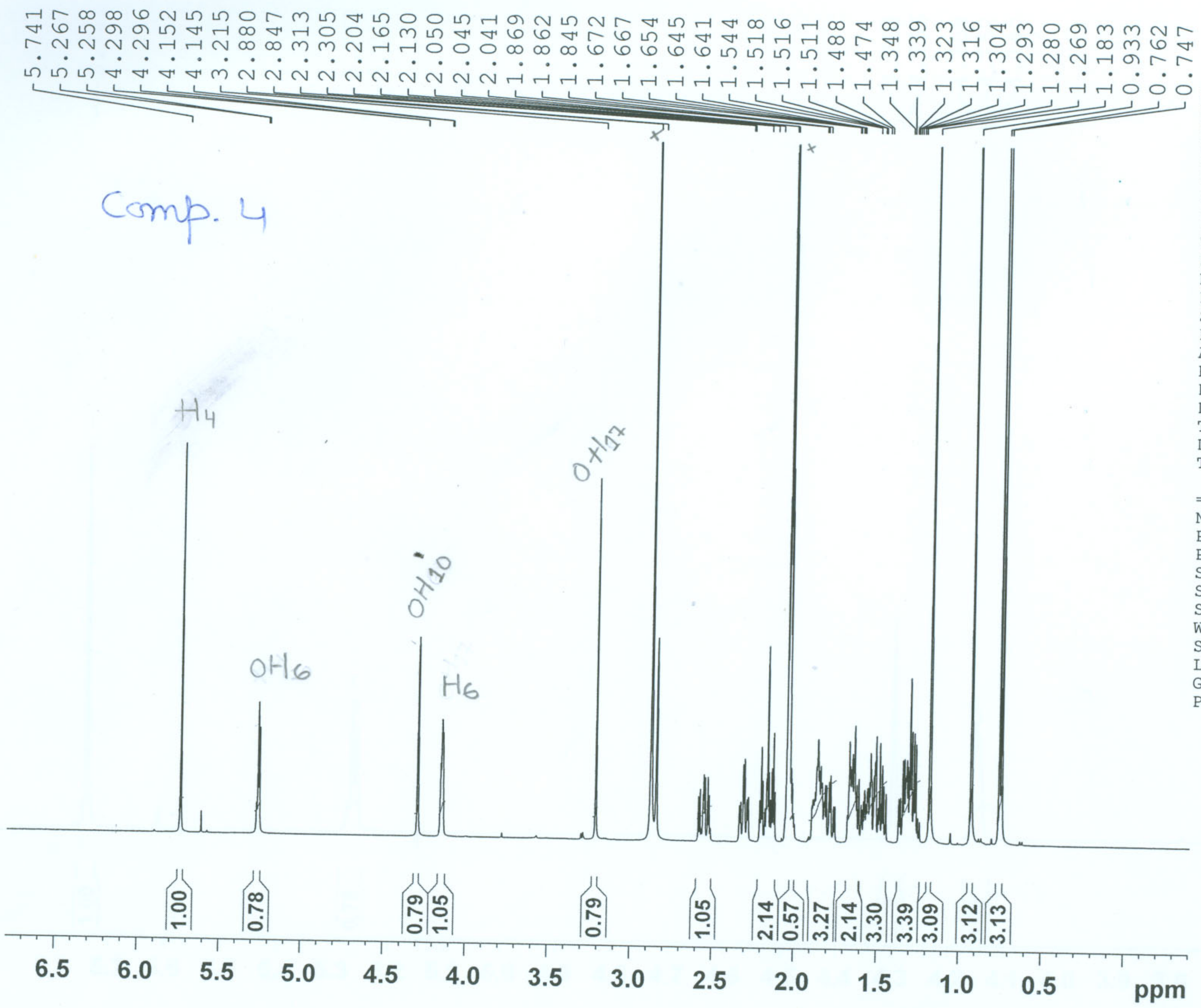

NAME	jan25-16
EXPNO	1
PROCNO	1
Date_	20160125
Time	16.08
INSTRUM	spect
PROBHD	5 mm BBI 1H/D-
PULPROG	zg30
TD	65536
SOLVENT	Acetone
NS	128
DS	0
SWH	10330.578 Hz
FIDRES	0.157632 Hz
AQ	3.1720407 sec
RG	71.8
DW	48.400 usec
DE	6.50 usec
TE	295.2 K
D1	1.50000000 sec
TD0	1

======== CHANNEL f1 ========

NUC1	1H
P1	8.00 usec
PL1	-1.00 dB
SFO1	500.1342511 MHz
SI	32768
SF	500.1300147 MHz
WDW	EM
SSB	0
LB	0.30 Hz
GB	0
PC	1.00

MAHWISH/DR.IQBAL/C-M-11/C3D6O
HSQC

AVANCE AV-500
LAB NO: 109B

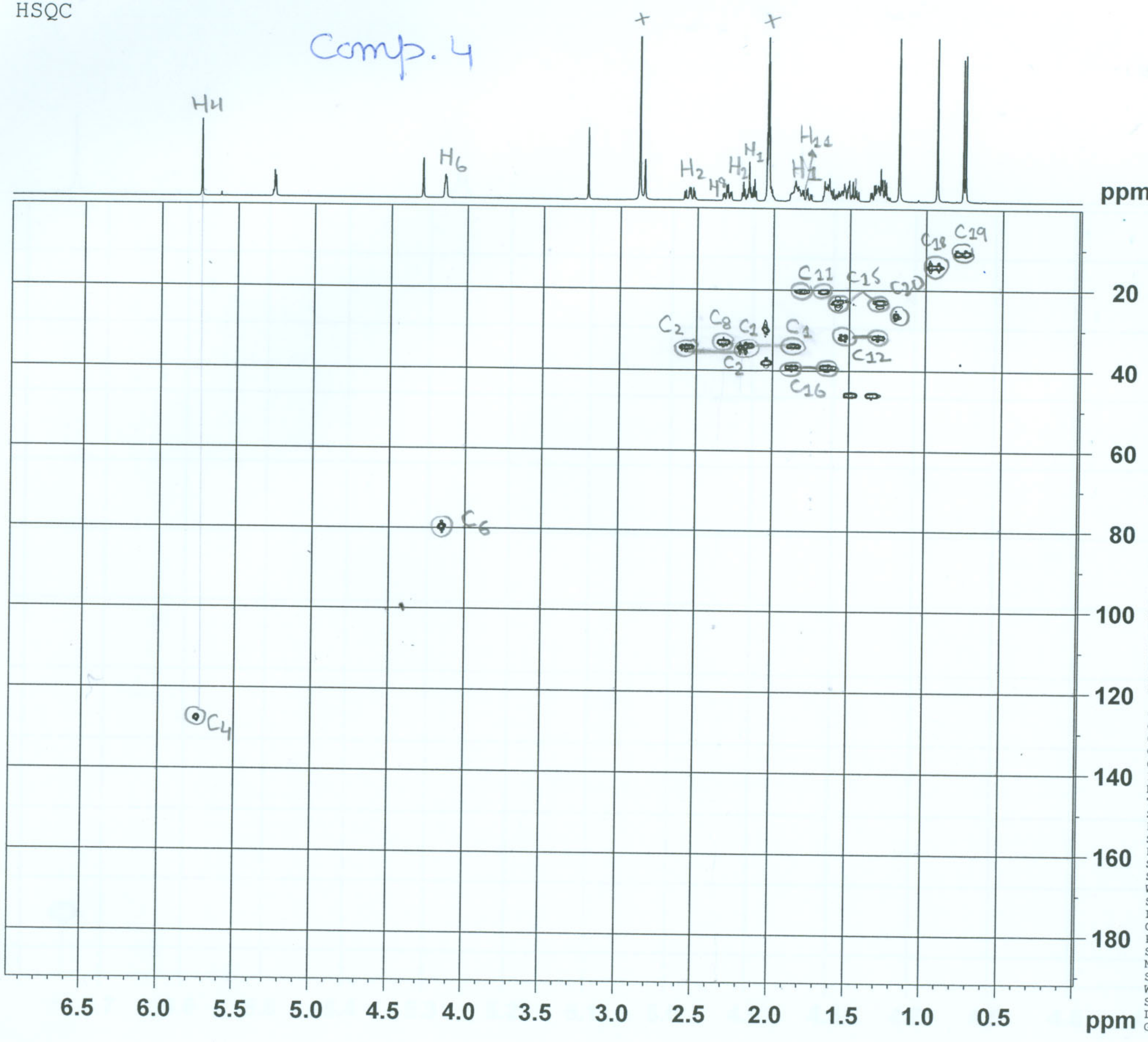

NAME	jan25-16
EXPNO	4
PROCNO	1
Date_	20160125
Time	20.46
INSTRUM	spect
PROBHD	5 mm BBI 1H/D-
PULPROG	hsqcedetgp
TD	1024
SOLVENT	Acetone
NS	32
DS	8
SWH	3501.401 Hz
FIDRES	3.419337 Hz
AQ	0.1464200 sec
RG	26008
DW	142.800 usec
DE	6.50 usec
TE	298.3 K
CNST2	145.0000000
D0	0.00000300 sec
D1	2.00000000 sec
D4	0.00172414 sec
D11	0.03000000 sec
D13	0.00000400 sec
D16	0.00020000 sec
D21	0.00350000 sec
IN0	0.00002070 sec
ZGOPTNS	
======== CHANNEL f1 ========	
NUC1	1H
P1	8.00 usec
P2	16.00 usec
P28	1000.00 usec
PL1	-1.00 dB
SFO1	500.1317505 MHz
======== CHANNEL f2 ========	
CPDPRG2	garp
NUC2	13C
P3	12.70 usec
P4	25.40 usec
PCPD2	65.00 usec
PL2	-3.00 dB
PL12	11.67 dB
SFO2	125.7697360 MHz
====== GRADIENT CHANNEL =====	
GPNAM1	SINE.100
GPNAM2	SINE.100
GPZ1	80.00 %
GPZ2	20.10 %
P16	1000.00 usec
ND0	2
TD	256
SFO1	125.7697 MHz
FIDRES	94.327301 Hz
SW	192.000 ppm
FnMODE	Echo-Antiecho
SI	1024
SF	500.1300102 MHz
WDW	QSINE
SSB	2
LB	0.00 Hz
GB	0
PC	4.00
SI	1024
MC2	echo-antiecho
SF	125.7576836 MHz
WDW	QSINE
SSB	2
LB	0.00 Hz
GB	0

MAHWISH/DR.IQBAL/C-M-11/C3D6O
ICCBS/U.O.K
HMBC

Comp. 4

AVANCE AV-500
LAB NO: 109B

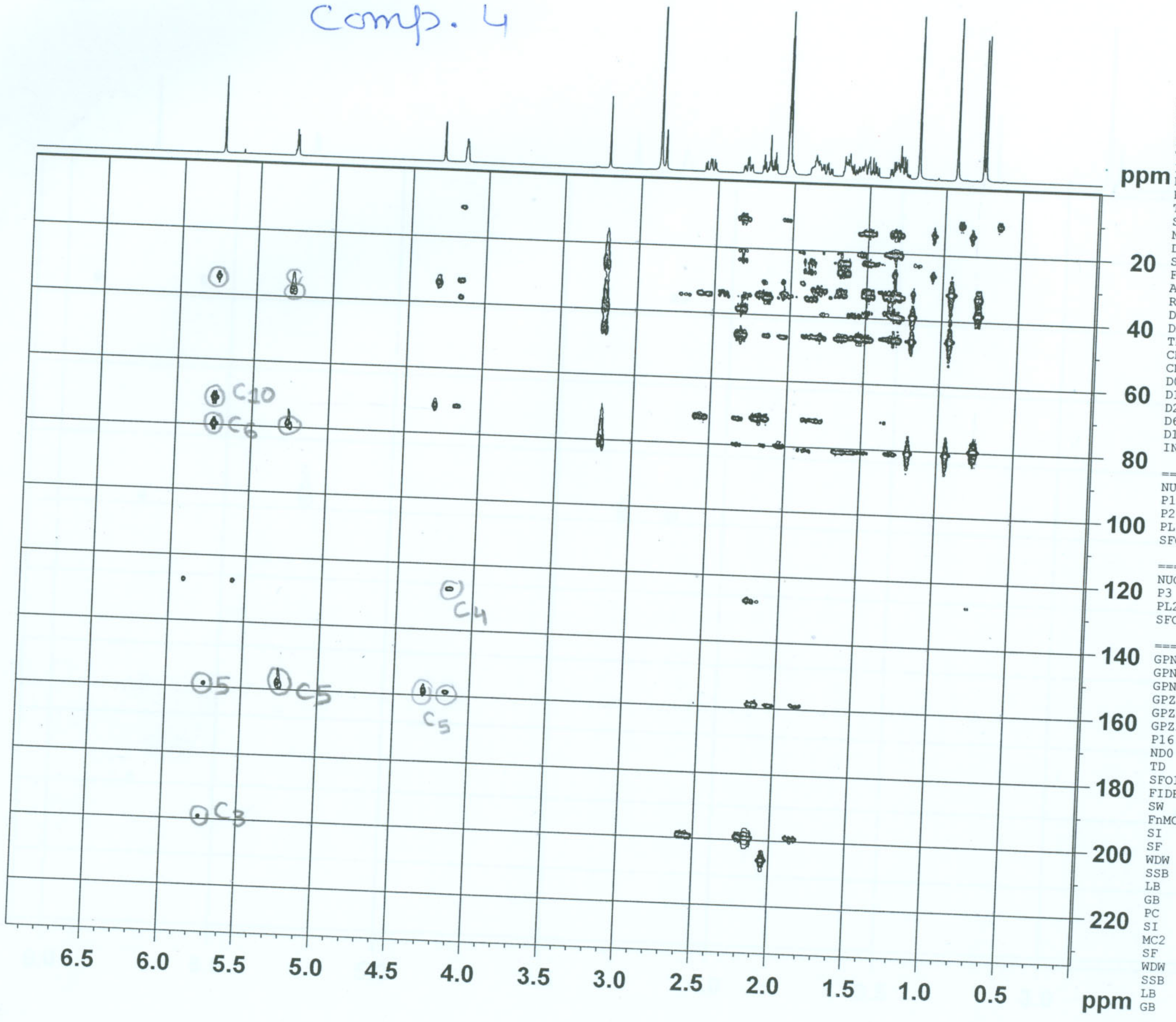

NAME jan25-16
EXPNO 5
PROCNO 1
Date_ 20160126
Time 1.43
INSTRUM spect
PROBHD 5 mm BBI 1H/D-
PULPROG hmbcgplpndqf
TD 2048
SOLVENT Acetone
NS 64
DS 8
SWH 3501.401 Hz
FIDRES 1.709668 Hz
AQ 0.2926472 sec
RG 20642.5
DW 142.800 usec
DE 6.50 usec
TE 299.0 K
CNST2 145.0000000
CNST13 10.0000000
D0 0.00000300 sec
D1 2.00000000 sec
D2 0.00344828 sec
D6 0.05000000 sec
D16 0.00020000 sec
IN0 0.00001690 sec

======== CHANNEL f1 ========
NUC1 1H
P1 8.00 usec
P2 16.00 usec
PL1 -1.00 dB
SFO1 500.1317505 MHz

======== CHANNEL f2 ========
NUC2 13C
P3 12.70 usec
PL2 -3.00 dB
SFO2 125.7723769 MHz

====== GRADIENT CHANNEL =====
GPNAM1 SINE.100
GPNAM2 SINE.100
GPNAM3 SINE.100
GPZ1 50.00 %
GPZ2 30.00 %
GPZ3 40.10 %
P16 1000.00 usec
ND0 2
TD 256
SFO1 125.7724 MHz
FIDRES 115.455109 Hz
SW 235.000 ppm
FnMODE QF
SI 1024
SF 500.1300102 MHz
WDW SINE
SSB 0
LB 0.00 Hz
GB 0
PC 4.00
SI 1024
MC2 QF
SF 125.7576836 MHz
WDW SINE
SSB 0
LB 0.00 Hz
GB 0

MAHWISH/DR.IQBAL/C-M-11/C3D6O
ICCBS/U.O.K
COSY

Comp. 4

AVANCE AV-500
LAB NO: 109B

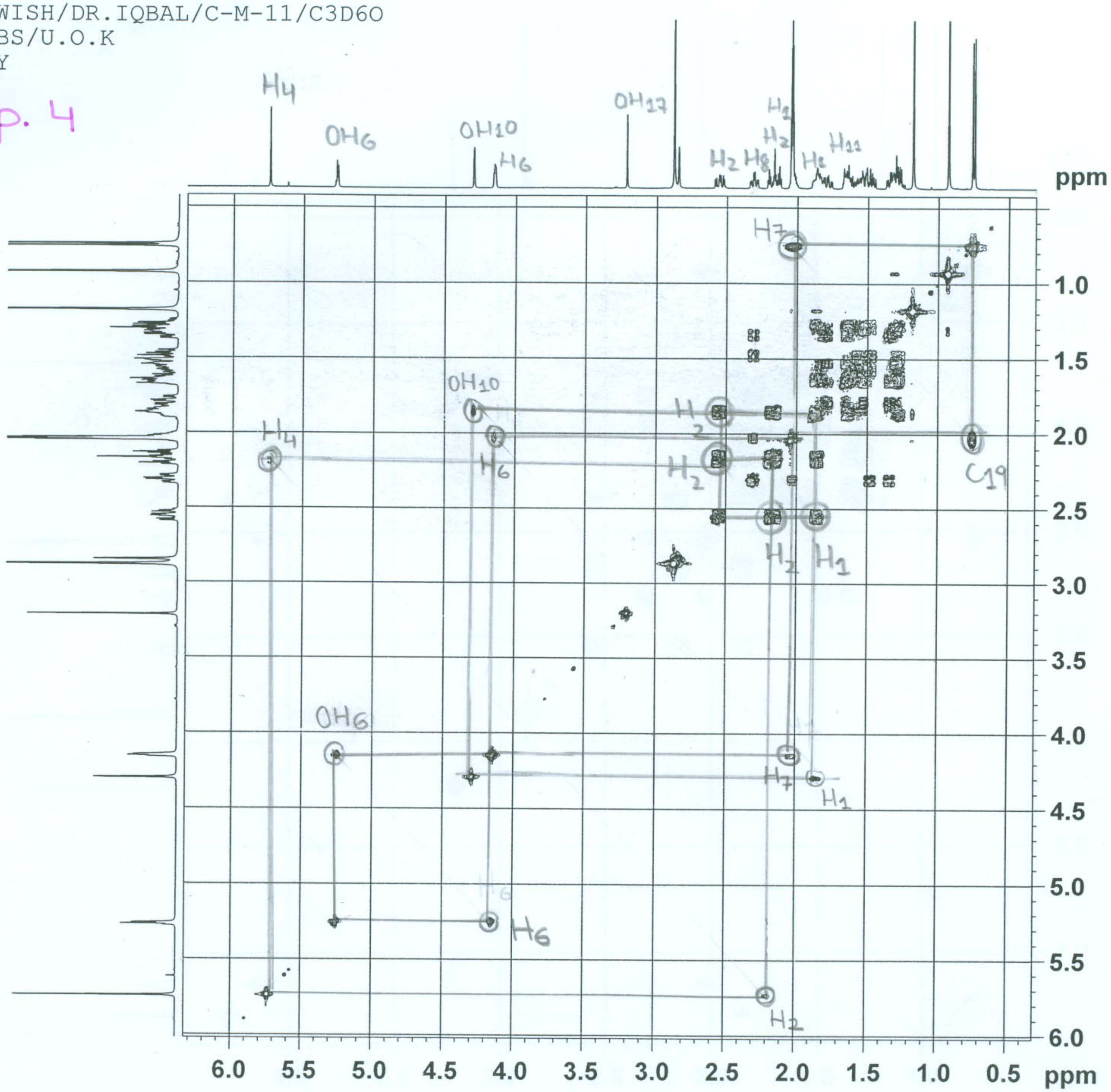

NAME	jan25-16	
EXPNO	2	
PROCNO	1	
Date_	20160125	
Time	16.09	
INSTRUM	spect	
PROBHD	5 mm BBI 1H/D-	
PULPROG	cosygpqf	
TD	2048	
SOLVENT	Acetone	
NS	8	
DS	8	
SWH	3501.401	Hz
FIDRES	1.709668	Hz
AQ	0.2926472	sec
RG	256	
DW	142.800	usec
DE	6.50	usec
TE	295.2	K
D0	0.00000300	sec
D1	1.50000000	sec
D13	0.00000400	sec
D16	0.00020000	sec
IN0	0.00028560	sec

======== CHANNEL f1 ========

NUC1	1H	
P0	8.00	usec
P1	8.00	usec
PL1	-1.00	dB
SFO1	500.1317505	MHz

====== GRADIENT CHANNEL =====

GPNAM1	SINE.100	
GPZ1	10.00	%
P16	1000.00	usec
ND0	1	
TD	256	
SFO1	500.1318	MHz
FIDRES	13.677351	Hz
SW	7.001	ppm
FnMODE	QF	
SI	1024	
SF	500.1300147	MHz
WDW	SINE	
SSB	0	
LB	0.00	Hz
GB	0	
PC	4.00	
SI	1024	
MC2	QF	
SF	500.1300147	MHz
WDW	SINE	
SSB	0	
LB	0.00	Hz
GB	0	

MAHWISH/DR.IQBAL/C-M-11/C3D6O
ICCBS/U.O.K
NOESY

Comp. 4

AVANCE AV-500
LAB NO: 109B

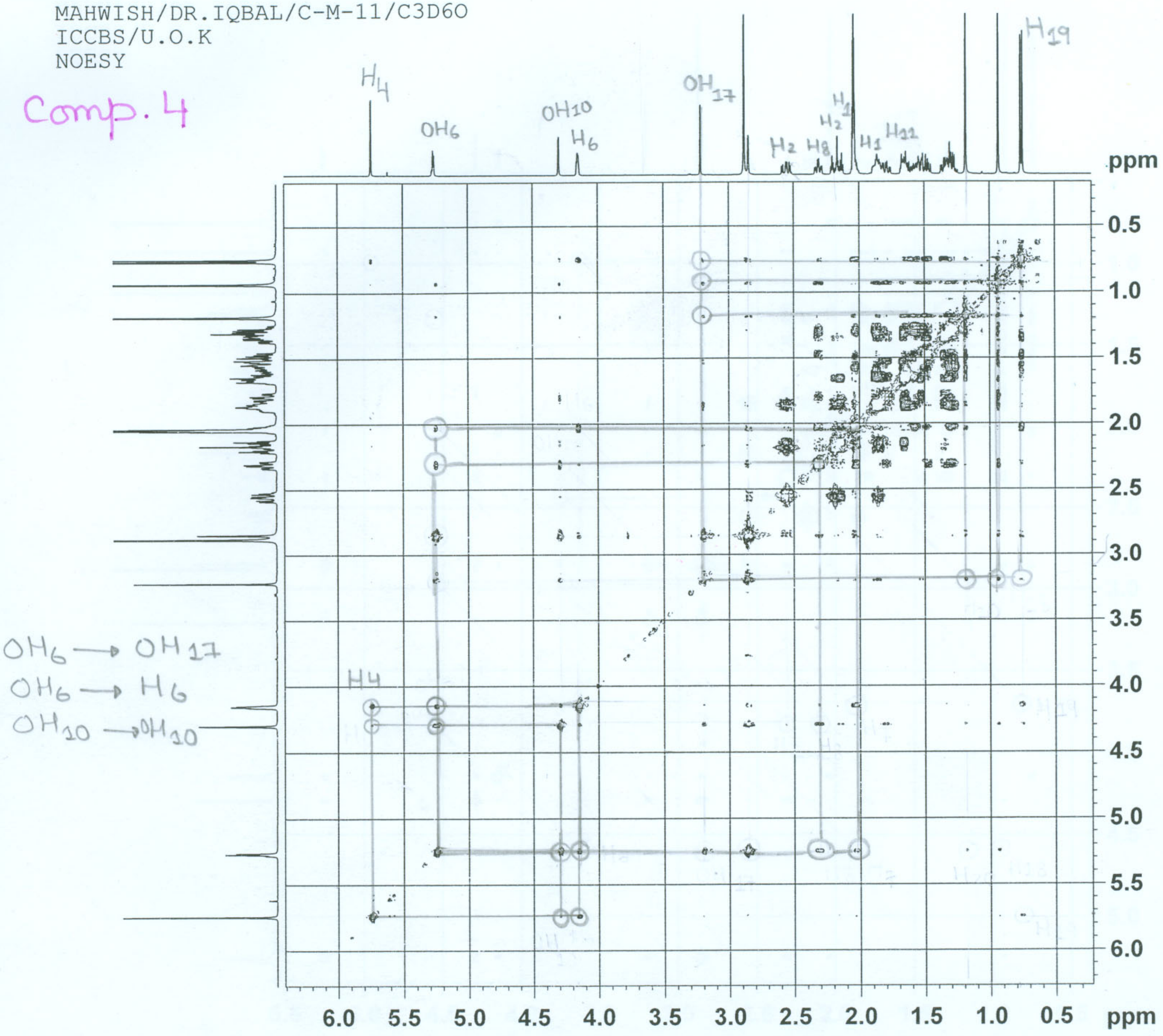

OH6 → OH17
OH6 → H6
OH10 → OH10

NAME	jan25-16
EXPNO	3
PROCNO	1
Date_	20160125
Time	17.12
INSTRUM	spect
PROBHD	5 mm BBI 1H/D-
PULPROG	noesygpph
TD	2048
SOLVENT	Acetone
NS	16
DS	2
SWH	3501.401 Hz
FIDRES	1.709668 Hz
AQ	0.2926472 sec
RG	362
DW	142.800 usec
DE	6.50 usec
TE	296.7 K
D0	0.00013261 sec
D1	2.00000000 sec
D8	0.80000001 sec
D16	0.00020000 sec
IN0	0.00028560 sec

======== CHANNEL f1 ========

NUC1	1H
P1	8.00 usec
P2	16.00 usec
PL1	-1.00 dB
SFO1	500.1317505 MHz

====== GRADIENT CHANNEL =====

GPNAM1	SINE.100
GPZ1	40.00 %
P16	1000.00 usec
ND0	1
TD	256
SFO1	500.1318 MHz
FIDRES	13.677351 Hz
SW	7.001 ppm
FnMODE	States-TPPI
SI	1024
SF	500.1300147 MHz
WDW	QSINE
SSB	2
LB	0.00 Hz
GB	0
PC	4.00
SI	1024
MC2	States-TPPI
SF	500.1300147 MHz
WDW	QSINE
SSB	2
LB	0.00 Hz
GB	0

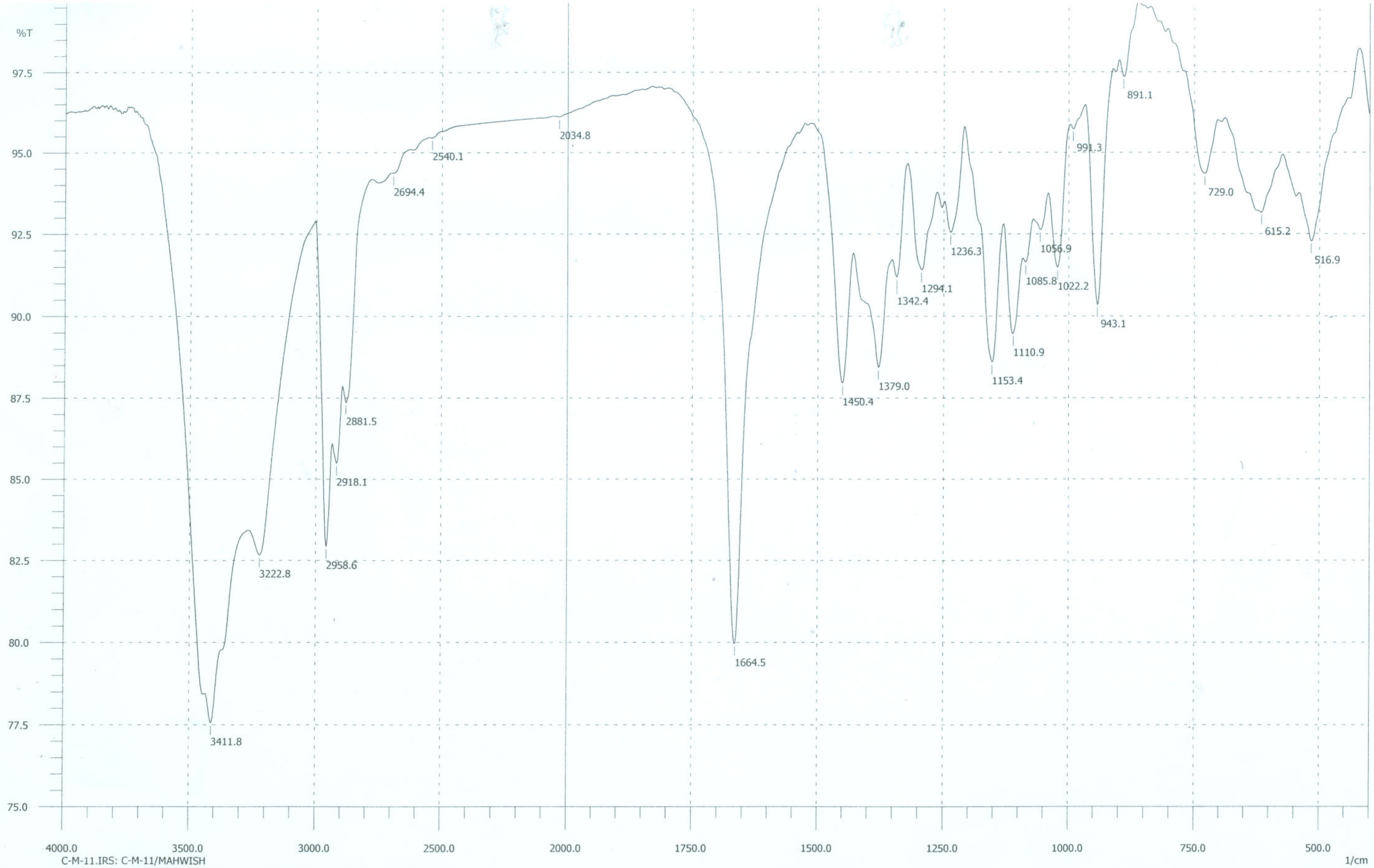

C-M-11.IRS: C-M-11/MAHWISH

Date:	06/30/2015	Time:	11:23:13	NScans:	10
Type:	HYPER IR	User:	ZUBAIR AHMED	Detector:	standard
Abscissa:	1/cm	Ordinate:	%T	Apodization:	Happ
Min:	401.17	Max:	3998.16	Range:	1/cm
Ndp:	1866	Data Interval:	1.92868	Resolution:	4.0
Gain:	auto	Aperture:	auto	Mirror Speed:	2.8(low)

Comp - 4

Compound 4

THERMO ELECTRON ~ VISIONpro SOFTWARE V4.10

Operator Name	Arshad Alam	Date of Report	11/6/2015
Department	Analytical laboratory#004 TWC	Time of Report	8:40:46AM
Organization	ICCBS.Karachi University.		
Information	Porf Dr. M.Iqbal /Mahwish.		

Scan Graph

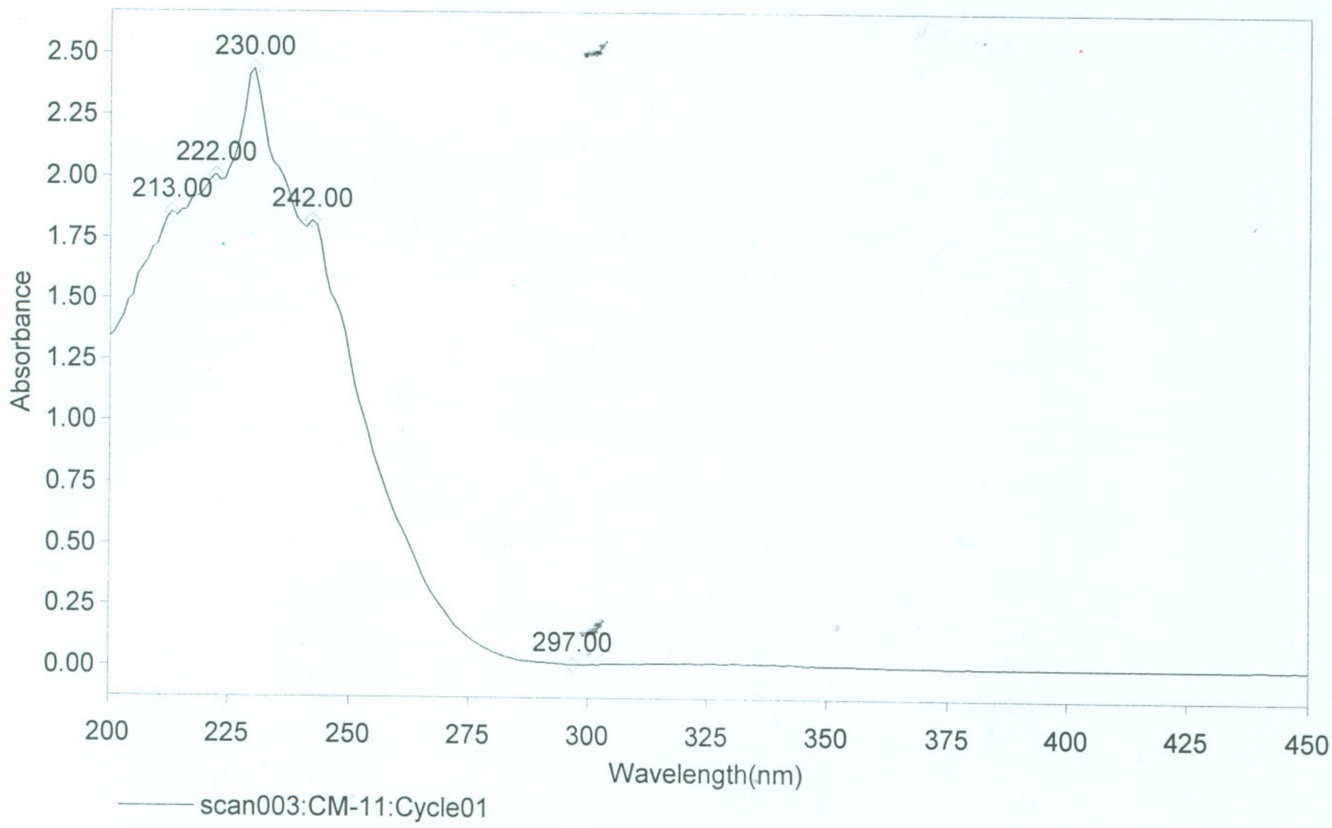

Results Table - CM-11.sre,CM-11,Cycle01

nm	A
213.00	1.854
222.00	2.005
230.00	2.435
242.00	1.819
297.00	0.012

Peak Pick Method

Find 8 Peaks Above -3.0000 A
Start Wavelength 200.00 nm
Stop Wavelength 300.00 nm
Sort By Wavelength
Sensitivity Very High

2ml —> 0.05+2ml
