# Supplementary material for: Biotransformation of a potent anabolic steroid, mibolerone, with Cunninghamella blakesleeana, C. echinulata, and Macrophomina phaseolina, and biological activity evaluation of its metabolites
Source: PLoS One. 2017 Feb 24;12(2):e0171476. doi: 10.1371/journal.pone.0171476 (PMC5325191; doi:10.1371/journal.pone.0171476)
Supplement: S4 Data — (PDF) [file pone.0171476.s004.pdf]

File: JM-25-2
Sample: MAHWISH / DR. M. IQBAL
Instrument: JEOL MS 600H-1

Date Run: 10-30-2015 (Time Run: 10:38:17)

Ionization mode: EI+

Scan: 13
Base: m/z 229; 29.5%FS TIC: 5723794

R.T.: 1.08

Compound 5

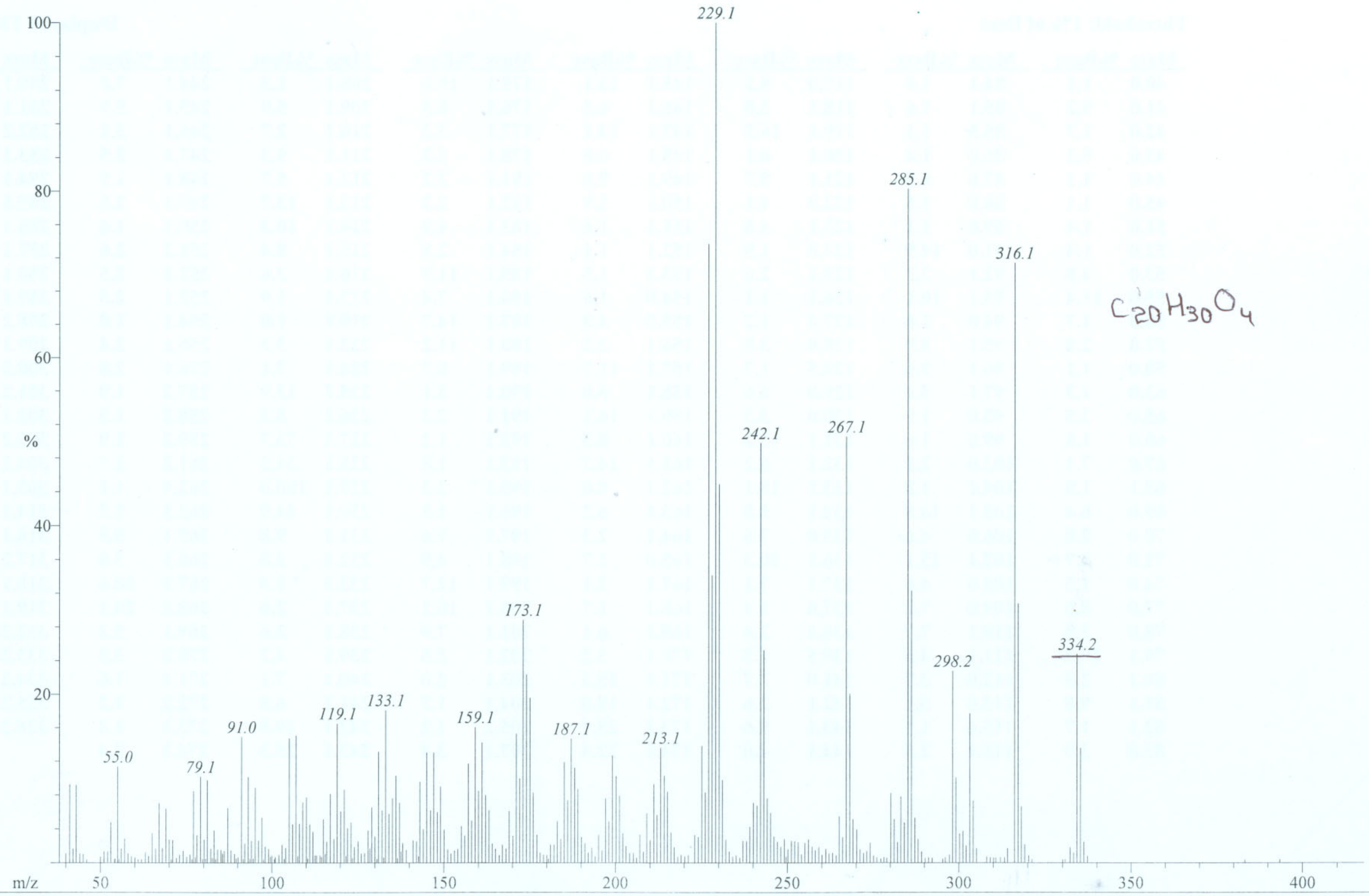

$C_{20}H_{30}O_4$

comp. 5

Mass	Relative Intensity	Theoretical Mass	Delta [ppm]	Delta [mmu]	RDB	Composition
		265.1651	-25.2	-6.7	0.5	$C_{12} H_{25} O_{6}$
266.1628	6.2	266.1671	-16.0	-4.3	9.0	$C_{19} H_{22} O_{1}$
267.1735	39.9	267.1749	-5.2	-1.4	8.5	$C_{19} H_{23} O_{1}$
268.1803	22.1	268.1827	-8.9	-2.4	8.0	$C_{19} H_{24} O_{1}$
269.1882	13.6	269.1905	-8.9	-2.4	7.5	$C_{19} H_{25} O_{1}$
270.1969	15.1	270.1984	-5.3	-1.4	7.0	$C_{19} H_{26} O_{1}$
271.2025	5.7	271.2062	-13.8	-3.7	6.5	$C_{19} H_{27} O_{1}$
272.1805	1.5	272.1776	10.6	2.9	7.0	$C_{18} H_{24} O_{2}$
272.2077	1.6	272.2140	-23.4	-6.4	6.0	$C_{19} H_{28} O_{1}$
279.1729	1.2	279.1749	-7.3	-2.0	9.5	$C_{20} H_{23} O_{1}$
		279.1808	-28.3	-7.9	0.5	$C_{13} H_{27} O_{6}$
280.1833	13.5	280.1827	2.1	0.6	9.0	$C_{20} H_{24} O_{1}$
		280.1886	-18.9	-5.3	0.0	$C_{13} H_{28} O_{6}$
281.1891	5.4	281.1905	-5.1	-1.4	8.5	$C_{20} H_{25} O_{1}$
282.1989	6.6	282.1984	1.9	0.5	8.0	$C_{20} H_{26} O_{1}$
283.1707	12.3	283.1698	3.1	0.9	8.5	$C_{19} H_{23} O_{2}$
284.1778	8.2	284.1776	0.5	0.2	8.0	$C_{19} H_{24} O_{2}$
285.1867	32.7	285.1855	4.5	1.3	7.5	$C_{19} H_{25} O_{2}$
286.1934	17.7	286.1933	0.6	0.2	7.0	$C_{19} H_{26} O_{2}$
287.1957	4.6	287.2011	-18.8	-5.4	6.5	$C_{19} H_{27} O_{2}$
288.2089	1.8	288.2089	-0.2	-0.1	6.0	$C_{19} H_{28} O_{2}$
296.1759	2.2	296.1776	-5.9	-1.8	9.0	$C_{20} H_{24} O_{2}$
		296.1835	-25.8	-7.6	0.0	$C_{13} H_{28} O_{7}$
297.1836	1.2	297.1855	-6.4	-1.9	8.5	$C_{20} H_{25} O_{2}$
298.1919	64.2	298.1933	-4.6	-1.4	8.0	$C_{20} H_{26} O_{2}$
299.1970	25.2	299.2011	-13.6	-4.1	7.5	$C_{20} H_{27} O_{2}$
300.2021	6.2	300.2089	-22.7	-6.8	7.0	$C_{20} H_{28} O_{2}$
		300.1937	28.1	8.4	3.0	$C_{16} H_{28} O_{5}$
301.1853	3.4	301.1804	16.4	4.9	7.5	$C_{19} H_{25} O_{3}$
302.1880	1.2	302.1882	-0.6	-0.2	7.0	$C_{19} H_{26} O_{3}$
303.1958	7.1	303.1960	-0.7	-0.2	6.5	$C_{19} H_{27} O_{3}$
304.1998	2.5	304.2038	-13.1	-4.0	6.0	$C_{19} H_{28} O_{3}$
314.1881	3.1	314.1882	-0.2	-0.1	8.0	$C_{20} H_{26} O_{3}$
315.1924	1.4	315.1960	-11.6	-3.7	7.5	$C_{20} H_{27} O_{3}$
316.2037	23.0	316.2038	-0.6	-0.2	7.0	$C_{20} H_{28} O_{3}$
317.2100	9.6	317.2117	-5.1	-1.6	6.5	$C_{20} H_{29} O_{3}$
318.2144	2.0	318.2195	-16.0	-5.1	6.0	$C_{20} H_{30} O_{3}$
334.2146	7.0	334.2144	0.5	0.2	6.0	$C_{20} H_{30} O_{4}$
335.2197	2.8	335.2222	-7.5	-2.5	5.5	$C_{20} H_{31} O_{4}$

Mahwish / Dr. Iqbal / JM-25-2 / MeOD
1H

Comp - 5

AVANCE AV-600-LC
[illegible]
LAB NO: 108

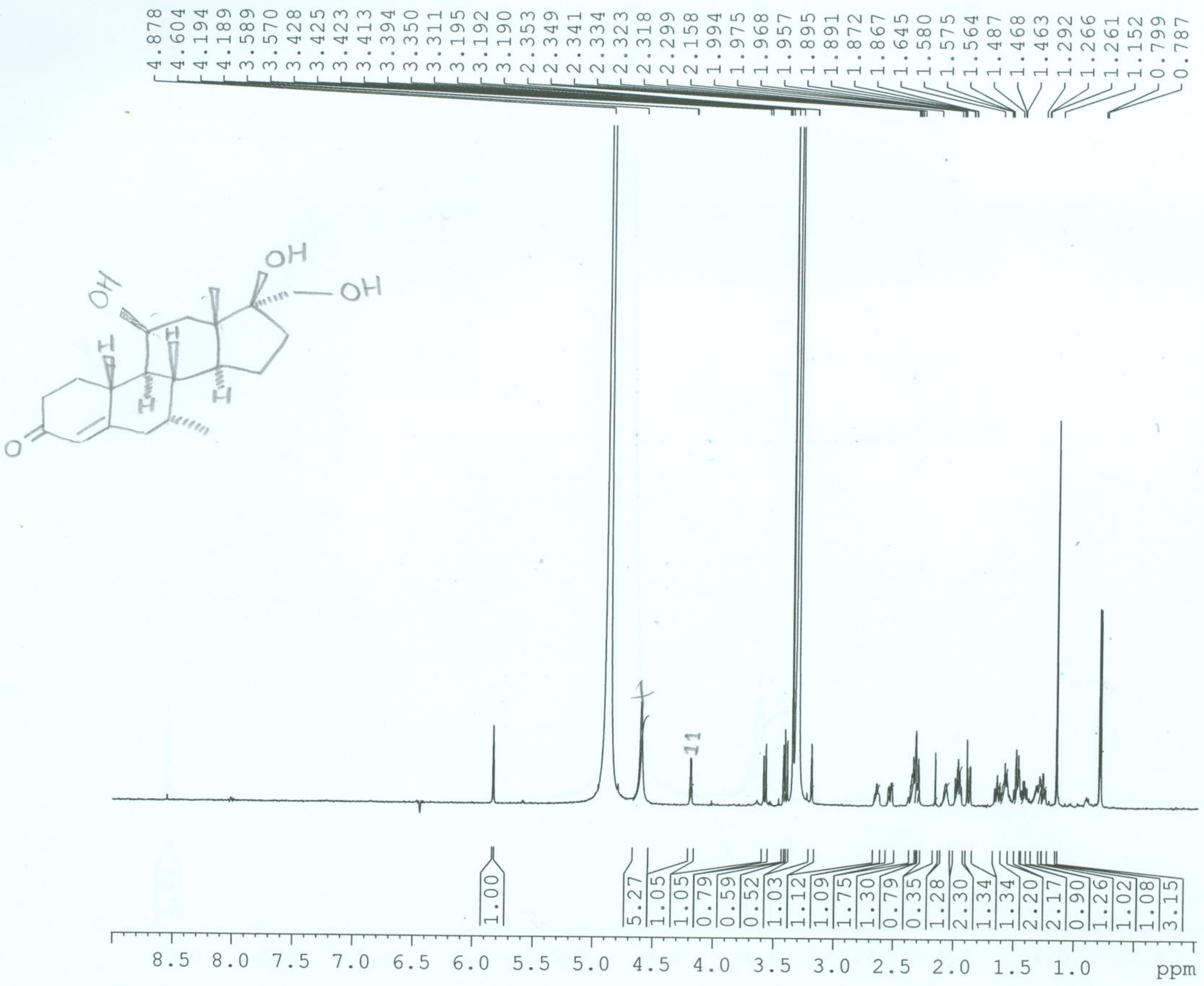

NAME	oct08-15
EXPNO	3
PROCNO	1
Date_	20151008
Time	13.13
INSTRUM	spect
PROBHD	5 mm CPTCI 1H-
PULPROG	zg30
TD	32768
SOLVENT	MeOD
NS	64
DS	0
SWH	12019.230 Hz
FIDRES	0.366798 Hz
AQ	1.3632404 sec
RG	9
DW	41.600 usec
DE	6.50 usec
TE	298.0 K
D1	2.00000000 sec
TD0	1

======== CHANNEL f1 ========

NUC1	1H
P1	8.00 usec
PL1	3.31 dB
PL1W	6.79873323 W
SFO1	600.0348002 MHz
SI	32768
SF	600.0300107 MHz
WDW	EM
SSB	0
LB	0.50 Hz
GB	0
PC	1.40

MAHWISH / Dr. IQBAL / JM-25-2 / CD3OD
BB

comp 5

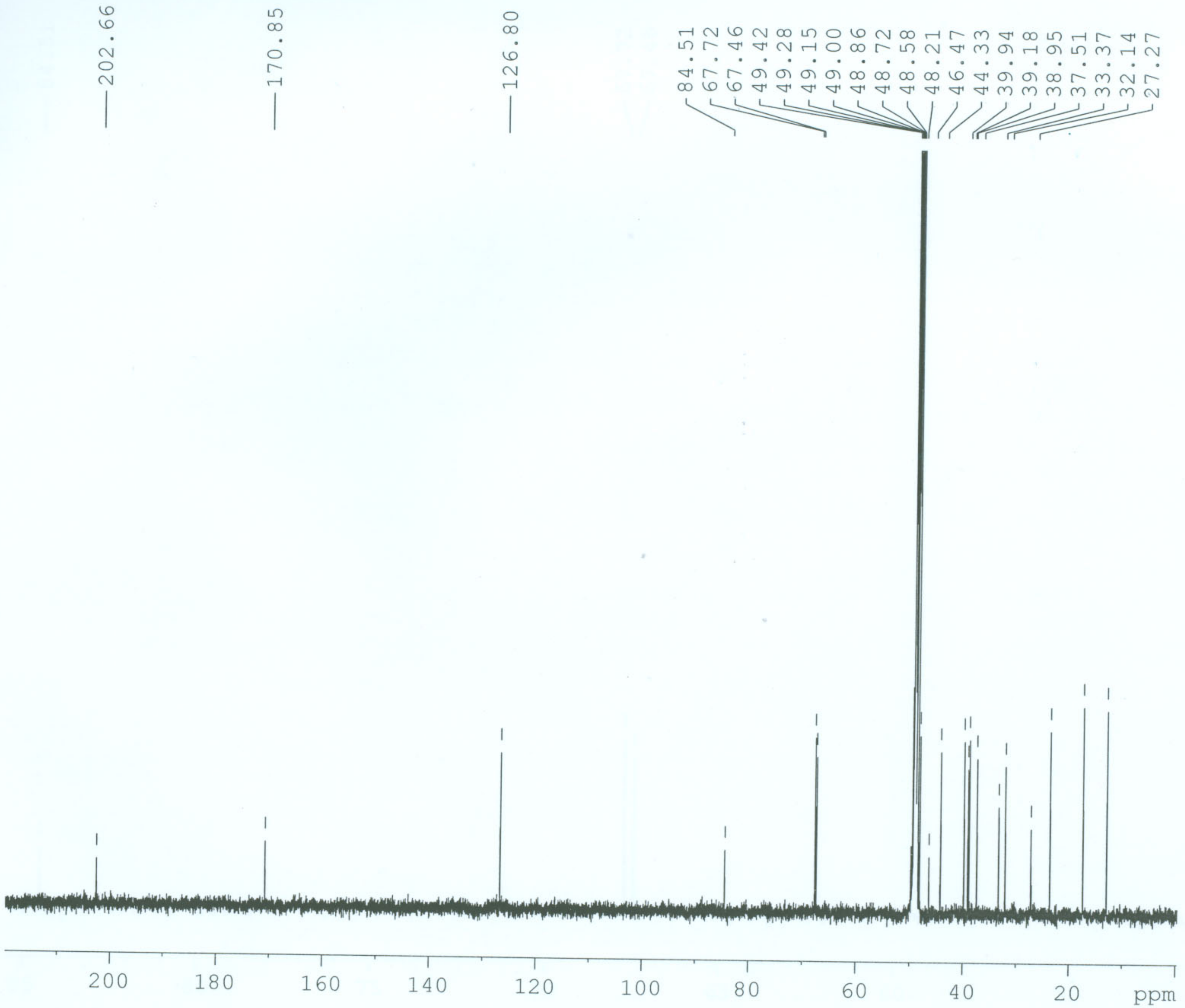

AVANCE AV-600
CRYO PROBE
LAB NO: 108

NAME	oct09-15
EXPNO	6
PROCNO	1
Date_	20151010
Time	14.26
INSTRUM	spect
PROBHD	5 mm CPTCI 1H-
PULPROG	zgpg
TD	32768
SOLVENT	MeOD
NS	16384
DS	2
SWH	35971.223 Hz
FIDRES	1.097755 Hz
AQ	0.4555391 sec
RG	32768
DW	13.900 usec
DE	6.50 usec
TE	298.0 K
D1	1.50000000 sec
D11	0.03000000 sec
TD0	16

======== CHANNEL f1 ========

NUC1	13C
P1	15.40 usec
PL1	1.00 dB
PL1W	83.60149384 W
SFO1	150.9453107 MHz

======== CHANNEL f2 ========

CPDPRG2	waltz16
NUC2	1H
PCPD2	65.00 usec
PL2	3.30 dB
PL12	22.06 dB
PL13	27.00 dB
PL2W	9.16420078 W
PL12W	0.12192553 W
PL13W	0.03909260 W
SFO2	600.2336014 MHz
SI	16384
SF	150.9277401 MHz
WDW	EM
SSB	0
LB	1.00 Hz
GB	0
PC	0.50

MAHWISH / Dr. IQBAL / JM-25-2 / CD3OD
DEPT135

Comp-5

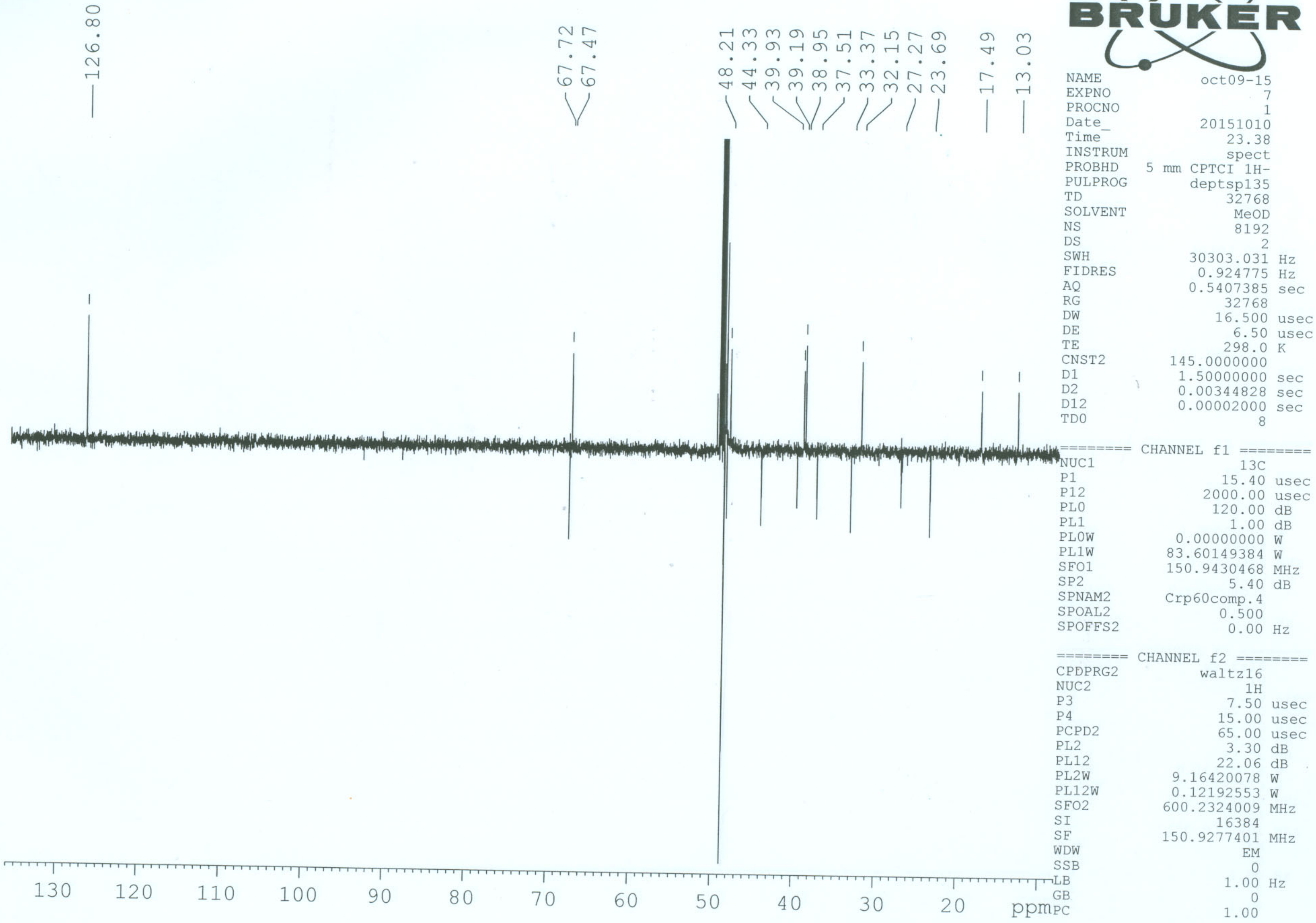

BRUKER

NAME	oct09-15	
EXPNO	7	
PROCNO	1	
Date_	20151010	
Time	23.38	
INSTRUM	spect	
PROBHD	5 mm CPTCI 1H-	
PULPROG	deptsp135	
TD	32768	
SOLVENT	MeOD	
NS	8192	
DS	2	
SWH	30303.031	Hz
FIDRES	0.924775	Hz
AQ	0.5407385	sec
RG	32768	
DW	16.500	usec
DE	6.50	usec
TE	298.0	K
CNST2	145.0000000	
D1	1.50000000	sec
D2	0.00344828	sec
D12	0.00002000	sec
TD0	8	

======== CHANNEL f1 ========

NUC1	13C	
P1	15.40	usec
P12	2000.00	usec
PL0	120.00	dB
PL1	1.00	dB
PL0W	0.00000000	W
PL1W	83.60149384	W
SFO1	150.9430468	MHz
SP2	5.40	dB
SPNAM2	Crp60comp.4	
SPOAL2	0.500	
SPOFFS2	0.00	Hz

======== CHANNEL f2 ========

CPDPRG2	waltz16	
NUC2	1H	
P3	7.50	usec
P4	15.00	usec
PCPD2	65.00	usec
PL2	3.30	dB
PL12	22.06	dB
PL2W	9.16420078	W
PL12W	0.12192553	W
SFO2	600.2324009	MHz
SI	16384	
SF	150.9277401	MHz
WDW	EM	
SSB	0	
LB	1.00	Hz
GB	0	
PC	1.00	

MAHWISH / Dr. IQBAL / JM-25-2 / CD3OD
DEPT90

comp - 5

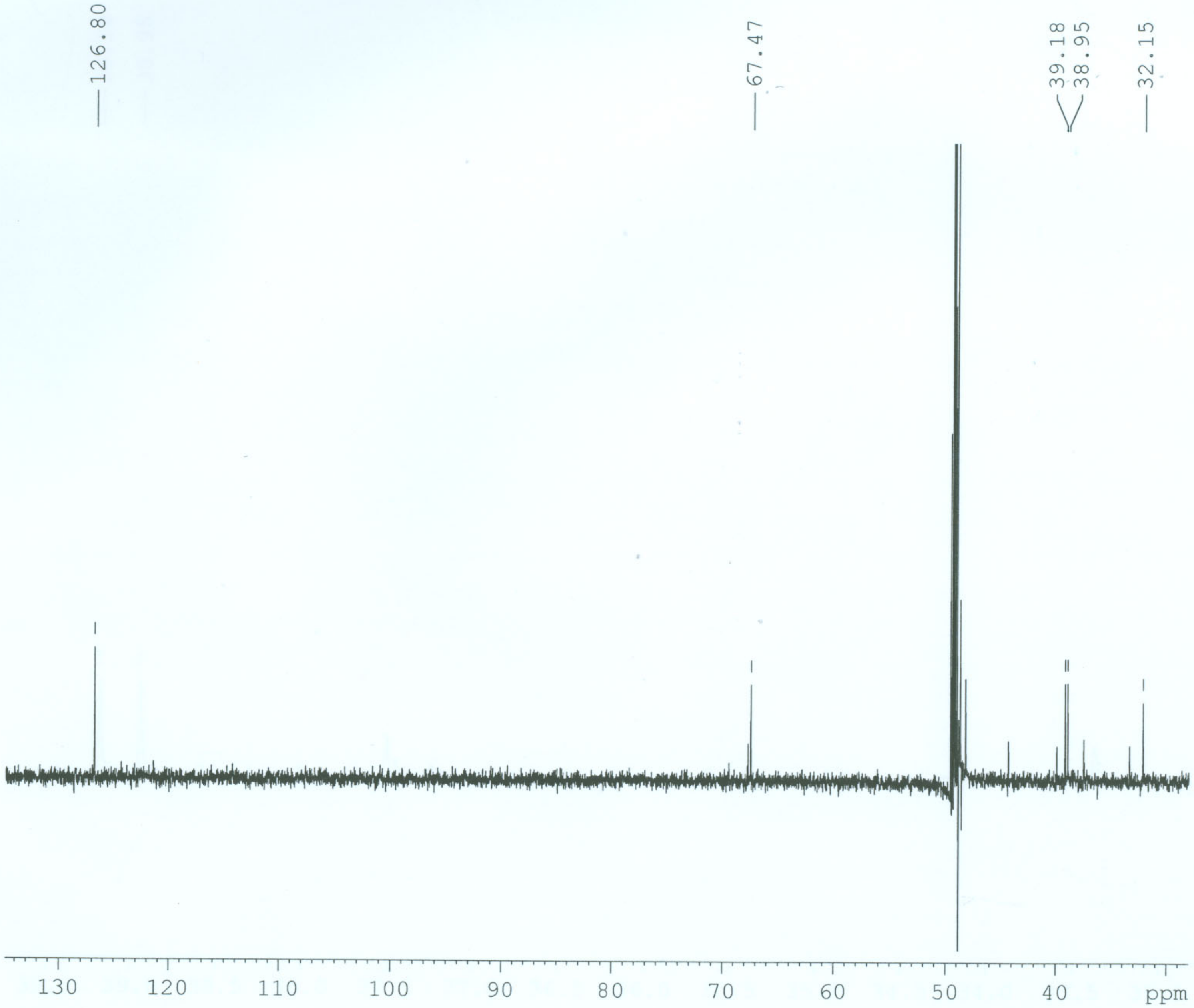

AVANCE AV-600
CRYO PROBE
LAB NO: 108

NAME	oct09-15
EXPNO	8
PROCNO	1
Date_	20151011
Time	4.23
INSTRUM	spect
PROBHD	5 mm CPTCI 1H-
PULPROG	deptsp90
TD	32768
SOLVENT	MeOD
NS	4096
DS	2
SWH	30303.031 Hz
FIDRES	0.924775 Hz
AQ	0.5407385 sec
RG	32768
DW	16.500 usec
DE	6.50 usec
TE	298.0 K
CNST2	145.0000000
D1	1.50000000 sec
D2	0.00344828 sec
D12	0.00002000 sec
TD0	4

======== CHANNEL f1 ========

NUC1	13C
P1	15.40 usec
P12	2000.00 usec
PL0	120.00 dB
PL1	1.00 dB
PL0W	0.00000000 W
PL1W	83.60149384 W
SFO1	150.9430468 MHz
SP2	5.40 dB
SPNAM2	Crp60comp.4
SPOAL2	0.500
SPOFFS2	0.00 Hz

======== CHANNEL f2 ========

CPDPRG2	waltz16
NUC2	1H
P3	7.50 usec
P4	15.00 usec
PCPD2	65.00 usec
PL2	3.30 dB
PL12	22.06 dB
PL2W	9.16420078 W
PL12W	0.12192553 W
SFO2	600.2324009 MHz
SI	16384
SF	150.9277401 MHz
WDW	EM
SSB	0
LB	1.00 Hz
GB	0
PC	1.00

MAHWISH / Dr. IQBAL / JM-25-2 / CD3OD
HSQC

comp-5

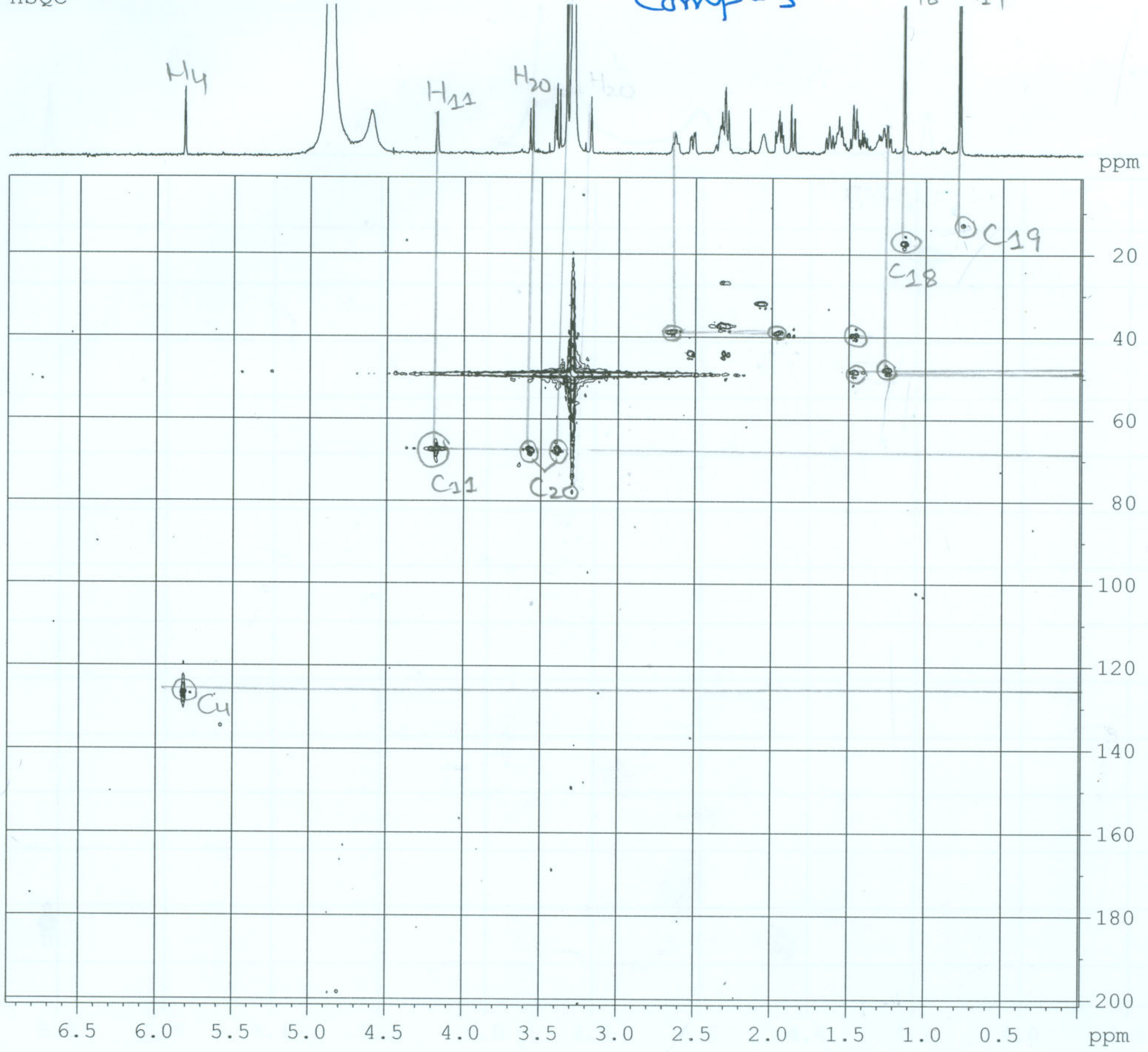

AVANCE AV-600
CRYO PROBE
LAB NO: 108

NAME	oct09-15
EXPNO	4
PROCNO	1
Date_	20151010
Time	0.47
INSTRUM	spect
PROBHD	5 mm CPTCI 1H-
PULPROG	hsqcetgpsi
TD	1024
SOLVENT	MeOD
NS	32
DS	8
SWH	4194.631 Hz
FIDRES	4.096319 Hz
AQ	0.1222300 sec
RG	29193
DW	119.200 usec
DE	6.50 usec
TE	298.0 K
CNST2	145.0000000
D0	0.00000300 sec
D1	2.00000000 sec
D4	0.00172414 sec
D11	0.03000000 sec
D13	0.00000400 sec
D16	0.00015000 sec
D24	0.00110000 sec
IN0	0.00001655 sec
ZGOPTNS	

======== CHANNEL f1 ========

NUC1	1H
P1	7.20 usec
P2	14.40 usec
P28	0.50 usec
PL1	3.30 dB
PL1W	9.16420078 W
SFO1	600.2321008 MHz

======== CHANNEL f2 ========

CPDPRG2	garp
NUC2	13C
P3	15.40 usec
P4	30.80 usec
PCPD2	61.00 usec
PL2	1.00 dB
PL12	13.00 dB
PL2W	83.60149384 W
PL12W	5.27489758 W
SFO2	150.9430468 MHz

====== GRADIENT CHANNEL =====

GPNAM1	SINE.100
GPNAM2	SINE.100
GPZ1	80.00 %
GPZ2	20.10 %
P16	2000.00 usec
ND0	2
TD	256
SFO1	150.943 MHz
FIDRES	117.924255 Hz
SW	200.000 ppm
FnMODE	Echo-Antiecho
SI	1024
SF	600.2300154 MHz
WDW	QSINE
SSB	2
LB	0.00 Hz
GB	0
PC	4.00
SI	1024
MC2	echo-antiecho
SF	150.9277401 MHz
WDW	QSINE
SSB	2
LB	0.00 Hz
GB	0

MAHWISH / Dr. IQBAL / JM-25-2 / CD3OD
HMBC

comp - 5

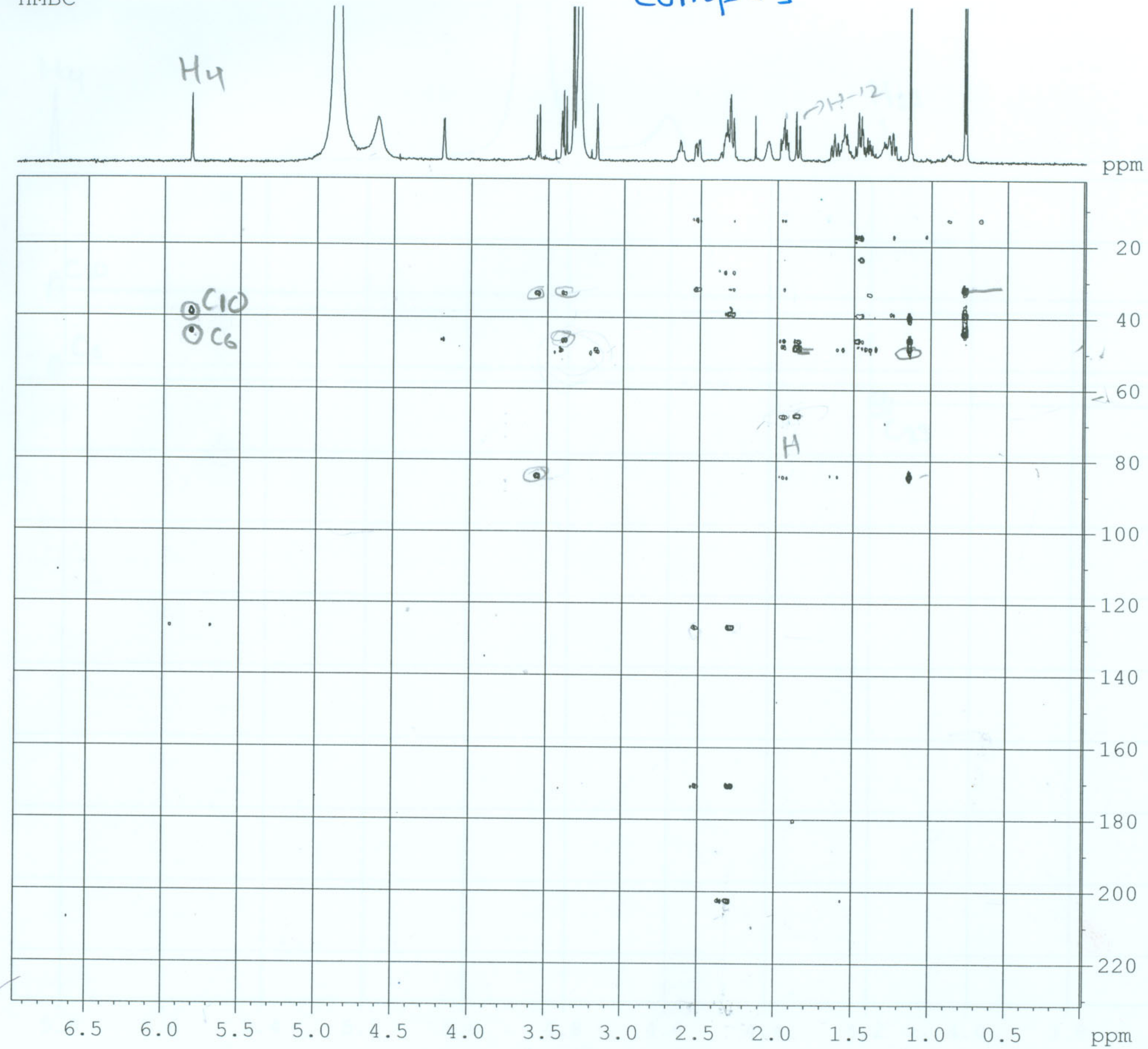

AVANCE AV-600
CRYO PROBE
LAB NO: 108

NAME	oct09-15	
EXPNO	5	
PROCNO	1	
Date_	20151010	
Time	5.41	
INSTRUM	spect	
PROBHD	5 mm CPTCI 1H-	
PULPROG	hmbcgplpndqf	
TD	2048	
SOLVENT	MeOD	
NS	64	
DS	16	
SWH	4194.631	Hz
FIDRES	2.048160	Hz
AQ	0.2442908	sec
RG	46341	
DW	119.200	usec
DE	6.50	usec
TE	298.0	K
CNST2	145.0000000	
CNST13	13.0000000	
D0	0.00000300	sec
D1	1.50000000	sec
D2	0.00344828	sec
D6	0.03846154	sec
D16	0.00015000	sec
IN0	0.00001440	sec

======== CHANNEL f1 ========

NUC1	1H	
P1	7.20	usec
P2	14.40	usec
PL1	3.30	dB
PL1W	9.16420078	W
SFO1	600.2321008	MHz

======== CHANNEL f2 ========

NUC2	13C	
P3	15.40	usec
PL2	1.00	dB
PL2W	83.60149384	W
SFO2	150.9453107	MHz

====== GRADIENT CHANNEL =====

GPNAM1	SINE.100	
GPNAM2	SINE.100	
GPNAM3	SINE.100	
GPZ1	50.00	%
GPZ2	30.00	%
GPZ3	40.10	%
P16	2000.00	usec
ND0	2	
TD	256	
SFO1	150.9453	MHz
FIDRES	135.614929	Hz
SW	230.000	ppm
FnMODE	QF	
SI	1024	
SF	600.2300154	MHz
WDW	SINE	
SSB	0	
LB	0.00	Hz
GB	0	
PC	1.40	
SI	1024	
MC2	QF	
SF	150.9277401	MHz
WDW	SINE	
SSB	0	
LB	0.00	Hz
GB	0	

MAHWISH / Dr. IQBAL / JM-25-2 // CD3OD
ICCBS/U.O.K
COSY

Comp 5

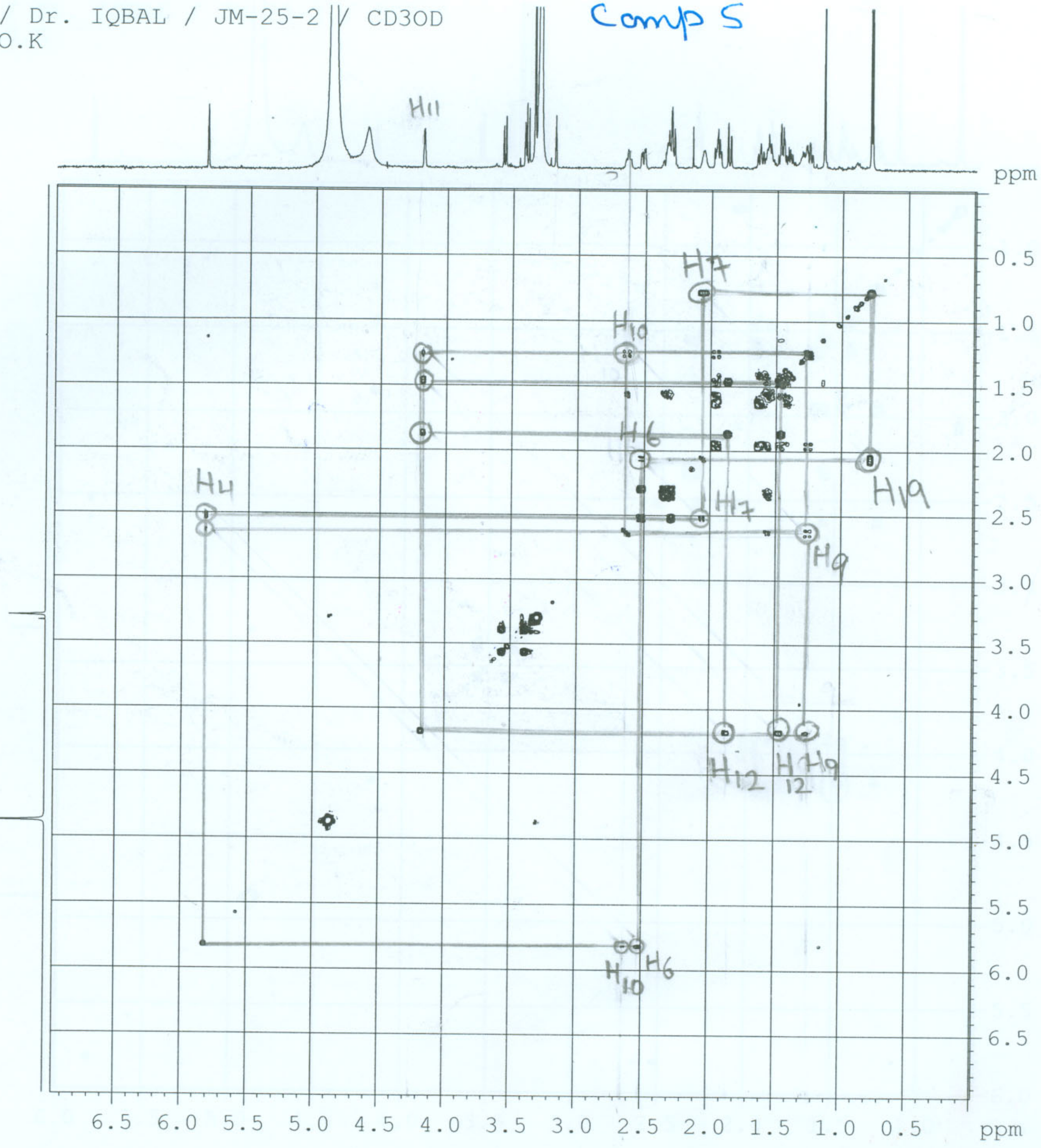

AVANCE AV-600
CRYO PROBE
LAB NO: 108

NAME	oct09-15	
EXPNO	2	
PROCNO	1	
Date_	20151009	
Time	16.02	
INSTRUM	spect	
PROBHD	5 mm CPTCI 1H-	
PULPROG	cosydfqf	
TD	2048	
SOLVENT	MeOD	
NS	16	
DS	4	
SWH	4194.631	Hz
FIDRES	2.048160	Hz
AQ	0.2442908	sec
RG	128	
DW	119.200	usec
DE	6.50	usec
TE	298.0	K
D0	0.00000300	sec
D1	1.50000000	sec
D13	0.00000400	sec
D20	0.00000200	sec
IN0	0.00023840	sec

======== CHANNEL f1 ========

NUC1	1H	
P1	7.20	usec
PL1	3.30	dB
PL1W	9.16420078	W
SFO1	600.2321008	MHz
ND0	1	
TD	256	
SFO1	600.2321	MHz
FIDRES	16.385277	Hz
SW	6.988	ppm
FnMODE	QF	
SI	1024	
SF	600.2300154	MHz
WDW	QSINE	
SSB	0	
LB	0.00	Hz
GB	0	
PC	1.40	
SI	1024	
MC2	QF	
SF	600.2300154	MHz
WDW	QSINE	
SSB	0	
LB	0.00	Hz
GB	0	

MAHWISH / Dr. IQBAL / JM-25-2 / CD3OD
ICCBS/U.O.K
NOESY

Comp - 5

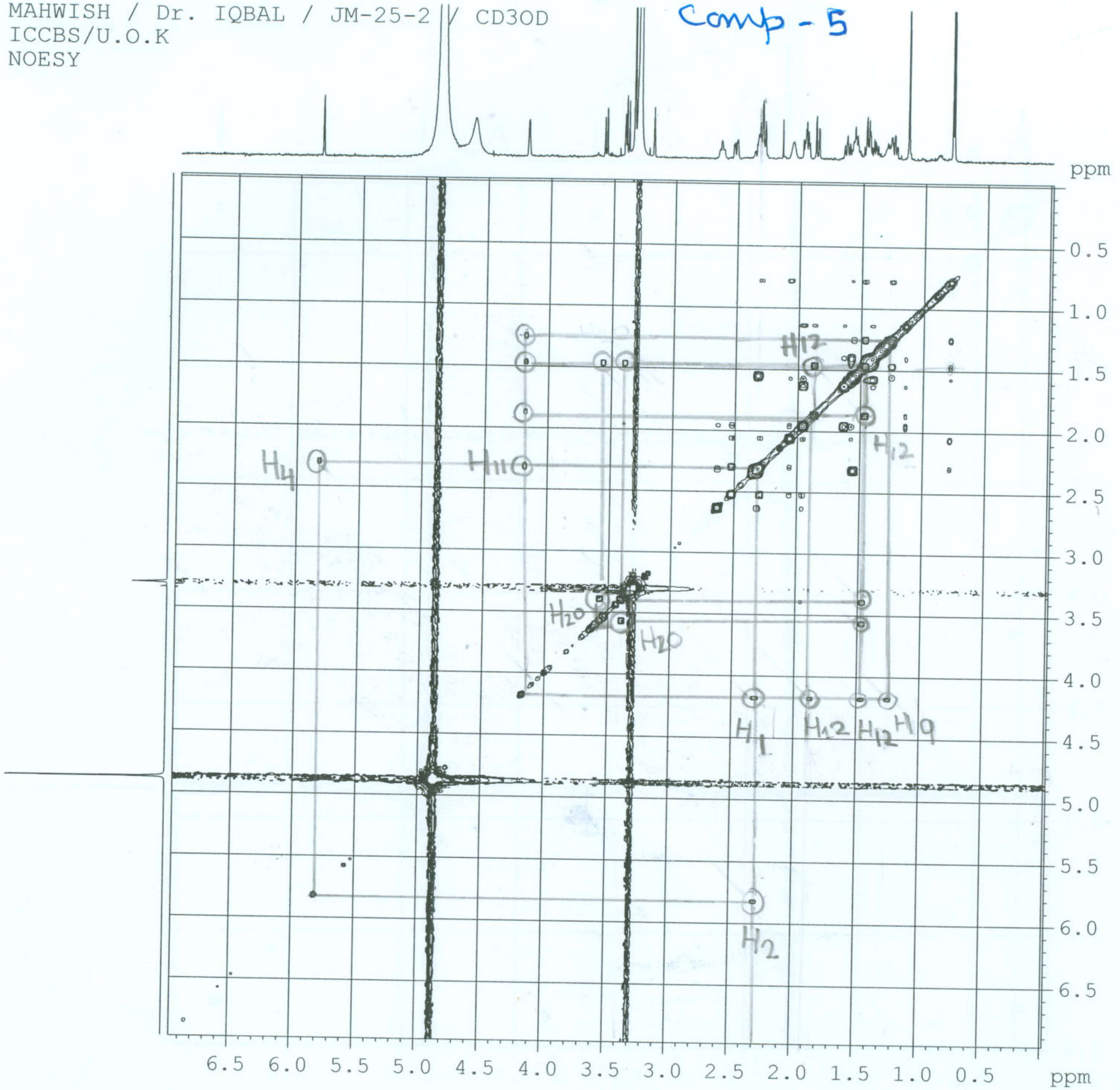

AVANCE AV-600
CRYO PROBE
LAB NO: 108

NAME	oct09-15
EXPNO	3
PROCNO	1
Date_	20151009
Time	18.05
INSTRUM	spect
PROBHD	5 mm CPTCI 1H-
PULPROG	noesygpph
TD	1024
SOLVENT	MeOD
NS	32
DS	4
SWH	4194.631 Hz
FIDRES	4.096319 Hz
AQ	0.1222300 sec
RG	71.8
DW	119.200 usec
DE	6.50 usec
TE	298.0 K
D0	0.00010978 sec
D1	2.00000000 sec
D8	0.80000001 sec
D16	0.00015000 sec
IN0	0.00023840 sec

======== CHANNEL f1 ========

NUC1	1H
P1	7.40 usec
P2	14.80 usec
PL1	3.30 dB
PL1W	9.16420078 W
SFO1	600.2321008 MHz

====== GRADIENT CHANNEL =====

GPNAM1	SINE.100
GPNAM2	SINE.100
GPZ1	40.00 %
GPZ2	-40.00 %
P16	2000.00 usec
ND0	1
TD	256
SFO1	600.2321 MHz
FIDRES	16.385277 Hz
SW	6.988 ppm
FnMODE	States-TPPI
SI	1024
SF	600.2300154 MHz
WDW	QSINE
SSB	2
LB	0.00 Hz
GB	0
PC	1.40
SI	1024
MC2	States-TPPI
SF	600.2300154 MHz
WDW	QSINE
SSB	2
LB	0.00 Hz
GB	0

Comp - 5

THERMO ELECTRON ~ VISIONpro SOFTWARE V4.10

Operator Name	Arshad Alam	Date of Report	10/8/2015
Department	Analytical laboratory#004 TWC	Time of Report	11:05:24AM
Organization	ICCBS.Karachi University.		
Information	Porf . Dr. M. Iqbal / Mahwish.		

Scan Graph

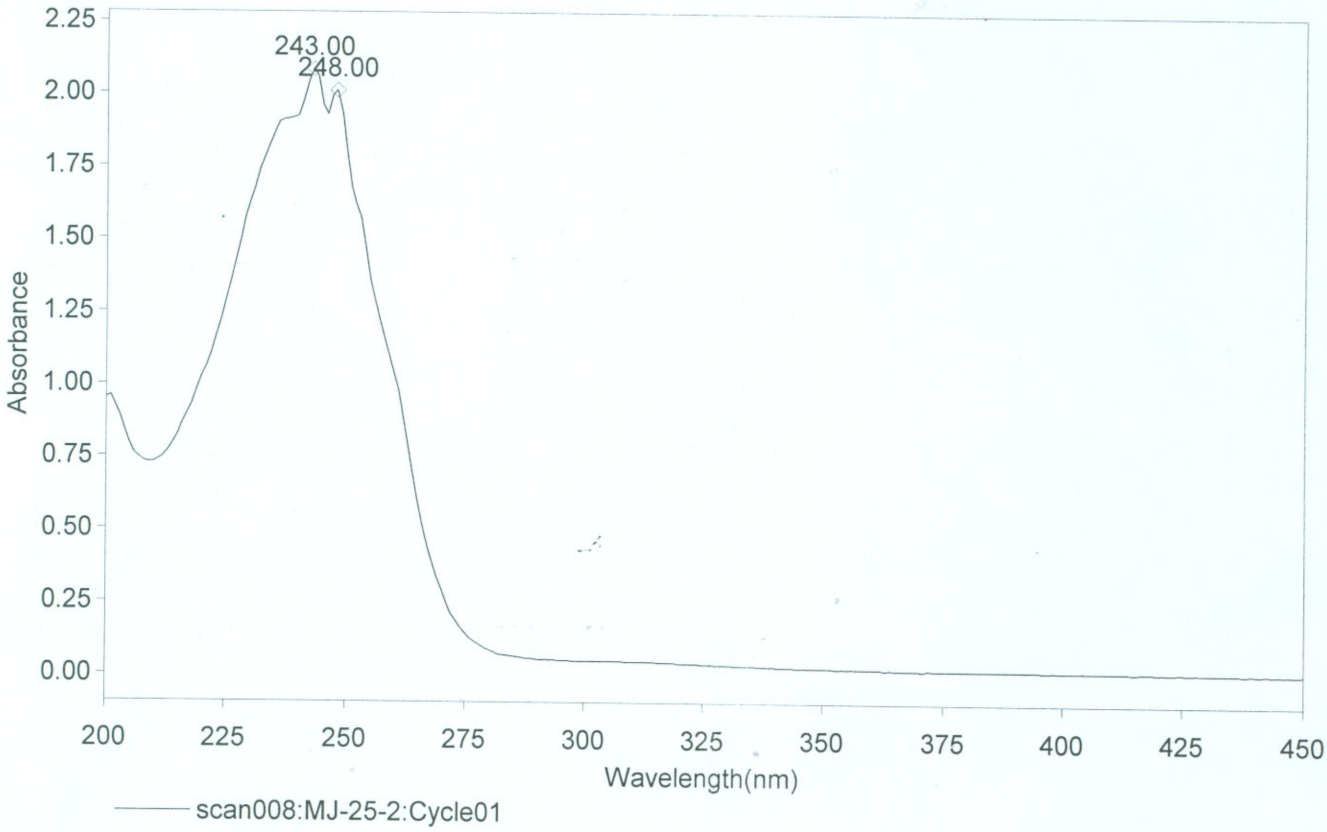

Results Table - JM- 25-2.sre,MJ-25-2,Cycle01

nm	A
243.00	2.083
248.00	2.010

Peak Pick Method
Find 8 Peaks Above -3.0000 A
Start Wavelength 200.00 nm
Stop Wavelength 450.00 nm
Sort By Wavelength

Sensitivity Medium

2ml → 0.3 ml + 2 ml

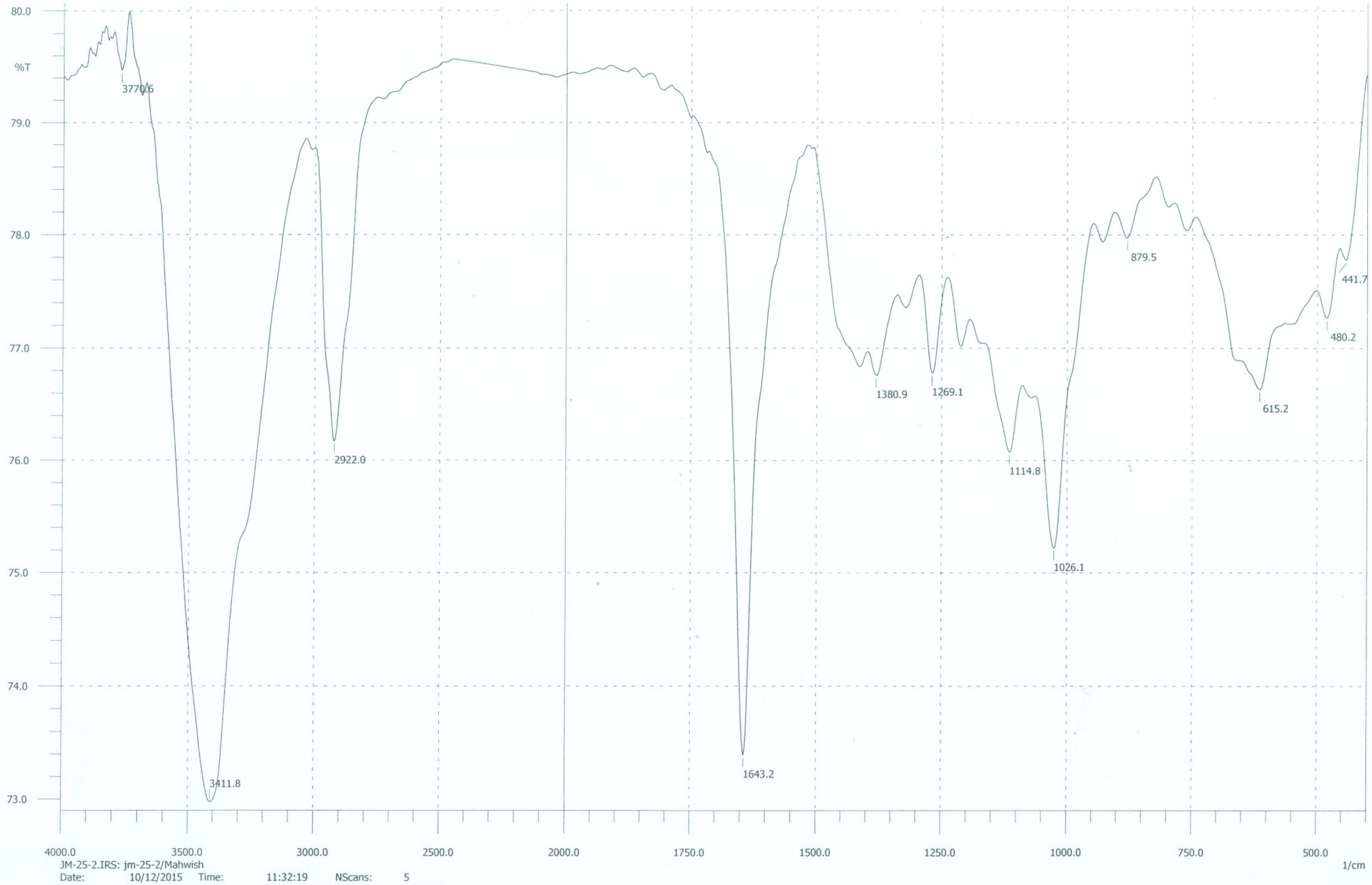

JM-25-2.IRS: jm-25-2/Mahwish

Date:	10/12/2015	Time:	11:32:19	NScans:	5
Type:	HYPER IR	User:	Zubair Ahmed	Detector:	standard
Abscissa:	1/cm	Ordinate:	%T	Apodization:	Happ
Min:	401.17	Max:	3998.16	Range:	1/cm
Ndp:	1866	Data Interval:	1.92868	Resolution:	4.0
Gain:	auto	Aperture:	auto	Mirror Speed:	2.8(low)

Comp - 5
