# Supplementary material for: Biotransformation of a potent anabolic steroid, mibolerone, with Cunninghamella blakesleeana, C. echinulata, and Macrophomina phaseolina, and biological activity evaluation of its metabolites
Source: PLoS One. 2017 Feb 24;12(2):e0171476. doi: 10.1371/journal.pone.0171476 (PMC5325191; doi:10.1371/journal.pone.0171476)
Supplement: S5 Data — (PDF) [file pone.0171476.s005.pdf]

HEJ MASS SECTION

11/4/2015 1:44:38 PM

File: IM-22-3
Sample: MAHWISH / DR. M. IQBAL
Instrument: JEOL MS 600H-1

Date Run: 11-04-2015 (Time Run: 13:39:43)

Ionization mode: EI+

comp 6

Scan: 15
Base: m/z 301; 8.8%FS TIC: 3071213

R.T.: 1.25

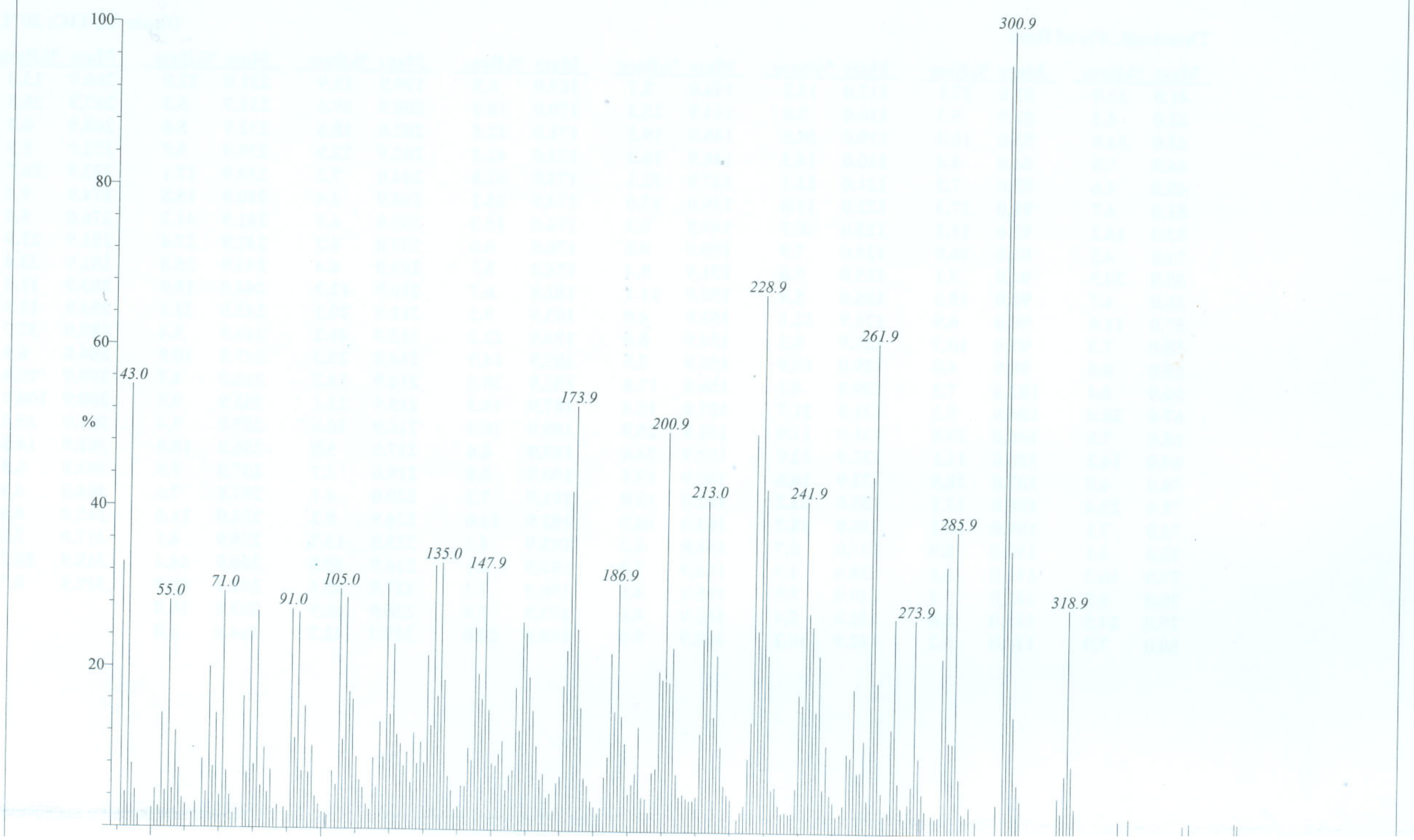

Comp. 6

Mass	Relative Intensity	Theoretical Mass	Delta [ppm]	Delta [mmu]	RDB	Composition
247.1630	4.0	247.1698	-27.6	-6.8	5.5	$C_{16} H_{23} O_2$
248.1748	2.9	248.1776	-11.4	-2.8	5.0	$C_{16} H_{24} O_2$
249.1737	2.1	249.1702	14.1	3.5	0.5	$C_{12} H_{25} O_5$
250.1837	1.0	250.1780	22.7	5.7	0.0	$C_{12} H_{26} O_5$
253.1500	2.8	253.1440	23.8	6.0	4.5	$C_{14} H_{21} O_4$
254.1642	3.7	254.1671	-11.2	-2.8	8.0	$C_{18} H_{22} O_1$
254.2016	3.2	254.2035	-7.4	-1.9	7.0	$C_{19} H_{26}$
255.1782	2.7	255.1749	12.8	3.3	7.5	$C_{18} H_{23} O_1$
255.2074	3.2	255.2113	-15.3	-3.9	6.5	$C_{19} H_{27}$
256.1823	8.7	256.1827	-1.6	-0.4	7.0	$C_{18} H_{24} O_1$
257.1874	3.5	257.1905	-12.1	-3.1	6.5	$C_{18} H_{25} O_1$
258.1580	1.6	258.1620	-15.4	-4.0	7.0	$C_{17} H_{22} O_2$
258.1971	1.6	258.1984	-4.8	-1.2	6.0	$C_{18} H_{26} O_1$
259.1670	2.8	259.1698	-10.7	-2.8	6.5	$C_{17} H_{23} O_2$
260.1783	1.5	260.1776	2.4	0.6	6.0	$C_{17} H_{24} O_2$
261.1850	19.1	261.1855	-1.9	-0.5	5.5	$C_{17} H_{25} O_2$
262.1901	22.3	262.1933	-12.1	-3.2	5.0	$C_{17} H_{26} O_2$
263.1942	6.9	263.2011	-26.2	-6.9	4.5	$C_{17} H_{27} O_2$
264.1912	1.3	264.1937	-9.5	-2.5	0.0	$C_{13} H_{28} O_5$
		264.1878	12.7	3.4	9.0	$C_{20} H_{24}$
267.1751	33.0	267.1749	0.9	0.2	8.5	$C_{19} H_{23} O_1$
268.1800	26.7	268.1827	-10.0	-2.7	8.0	$C_{19} H_{24} O_1$
269.1839	7.8	269.1905	-24.7	-6.6	7.5	$C_{19} H_{25} O_1$
270.1899	1.3	270.1831	25.1	6.8	3.0	$C_{15} H_{26} O_4$
274.1920	8.5	274.1933	-4.7	-1.3	6.0	$C_{18} H_{26} O_2$
275.1999	2.3	275.2011	-4.3	-1.2	5.5	$C_{18} H_{27} O_2$
276.2048	1.4	276.2089	-15.0	-4.2	5.0	$C_{18} H_{28} O_2$
281.1858	1.6	281.1905	-16.9	-4.8	8.5	$C_{20} H_{25} O_1$
282.1948	21.3	282.1984	-12.5	-3.5	8.0	$C_{20} H_{26} O_1$
283.1998	20.7	283.2062	-22.6	-6.4	7.5	$C_{20} H_{27} O_1$
284.2073	6.8	284.2140	-23.7	-6.7	7.0	$C_{20} H_{28} O_1$
		284.1988	30.0	8.5	3.0	$C_{16} H_{28} O_4$
285.1848	12.8	285.1855	-2.2	-0.6	7.5	$C_{19} H_{25} O_2$
286.1901	17.3	286.1933	-11.3	-3.2	7.0	$C_{19} H_{26} O_2$
287.1959	6.3	287.2011	-18.0	-5.2	6.5	$C_{19} H_{27} O_2$
298.1931	1.3	298.1933	-0.6	-0.2	8.0	$C_{20} H_{26} O_2$
299.1990	2.2	299.2011	-7.1	-2.1	7.5	$C_{20} H_{27} O_2$
300.2073	95.3	300.2089	-5.4	-1.6	7.0	$C_{20} H_{28} O_2$
301.2116	92.6	301.2168	-17.2	-5.2	6.5	$C_{20} H_{29} O_2$
302.2164	26.8	302.2093	23.3	7.0	2.0	$C_{16} H_{30} O_5$
		302.2246	-27.2	-8.2	6.0	$C_{20} H_{30} O_2$
303.2093	6.5	303.2113	-6.5	-2.0	10.5	$C_{23} H_{27}$
		303.2171	-25.9	-7.8	1.5	$C_{16} H_{31} O_5$
304.2002	2.5	304.2038	-11.8	-3.6	6.0	$C_{19} H_{28} O_3$
318.2193	3.6	318.2195	-0.7	-0.2	6.0	$C_{20} H_{30} O_3$
319.2311	4.6	319.2273	11.9	3.8	5.5	$C_{20} H_{31} O_3$
320.2256	1.4	320.2199	17.8	5.7	1.0	$C_{16} H_{32} O_6$
		320.2351	-29.8	-9.6	5.0	$C_{20} H_{32} O_3$

Repeat

Comp 6

MAHWISH/DR.IQBAL/JM.22.3'
1H

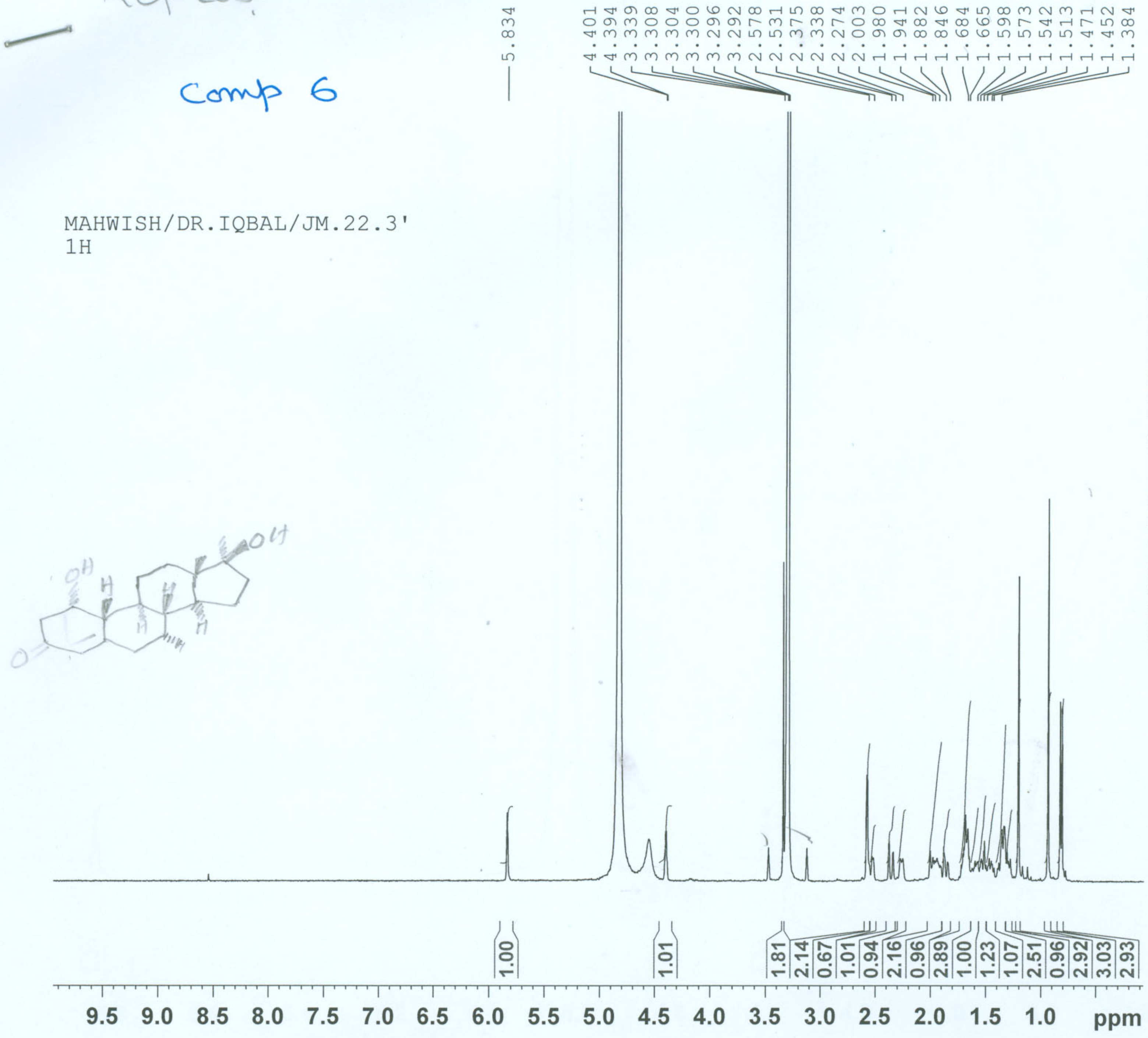

AVANCE AV-400 MHz
Lab # 115

NAME	sep15-15
EXPNO	18
PROCNO	1
Date_	20150915
Time	16.09
INSTRUM	spect
PROBHD	5 mm SEI 1H-13
PULPROG	zg30
TD	65536
SOLVENT	MeOD
NS	128
DS	0
SWH	8012.820 Hz
FIDRES	0.122266 Hz
AQ	4.0894966 sec
RG	362
DW	62.400 usec
DE	6.50 usec
TE	300.0 K
D1	2.00000000 sec
TD0	1

======== CHANNEL f1 ========

NUC1	1H
P1	10.80 usec
PL1	3.00 dB
SFO1	400.0332002 MHz
SI	32768
SF	400.0300087 MHz
WDW	EM
SSB	0
LB	0.30 Hz
GB	0
PC	1.00

Mehwish / Dr. Iqbal / JM-22-3 / MeOD
BB

Comp 6

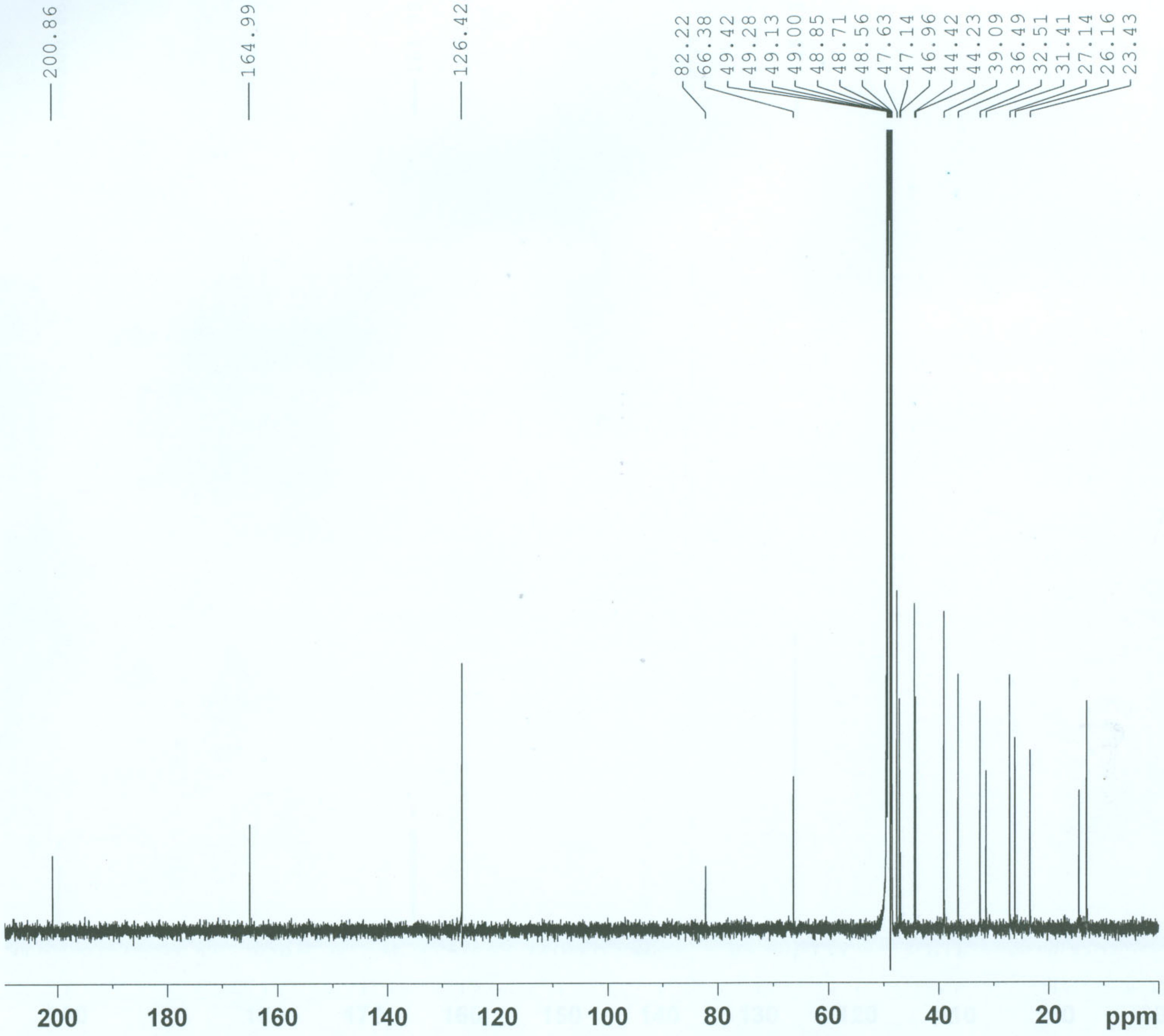

NAME	sep17-15
EXPNO	6
PROCNO	1
Date_	20150917
Time	17.16
INSTRUM	spect
PROBHD	5 mm CPTCI 1H-
PULPROG	zgpg
TD	32768
SOLVENT	MeOD
NS	12288
DS	4
SWH	35971.223 Hz
FIDRES	1.097755 Hz
AQ	0.4555391 sec
RG	32768
DW	13.900 usec
DE	6.50 usec
TE	298.1 K
D1	2.00000000 sec
D11	0.03000000 sec
TD0	12

======== CHANNEL f1 ========

NUC1	13C
P1	12.70 usec
PL1	-1.81 dB
PL1W	81.92915344 W
SFO1	150.8950149 MHz

======== CHANNEL f2 ========

CPDPRG2	waltz16
NUC2	1H
PCPD2	80.00 usec
PL2	3.31 dB
PL12	23.31 dB
PL13	22.50 dB
PL2W	6.79873323 W
PL12W	0.06798734 W
PL13W	0.08192718 W
SFO2	600.0336002 MHz
SI	16384
SF	150.8774513 MHz
WDW	EM
SSB	0
LB	1.00 Hz
GB	0
PC	1.00

MAHWISH / DR.IQBAL / JM-22-3' / CD3OD
DEPT135

comp 6

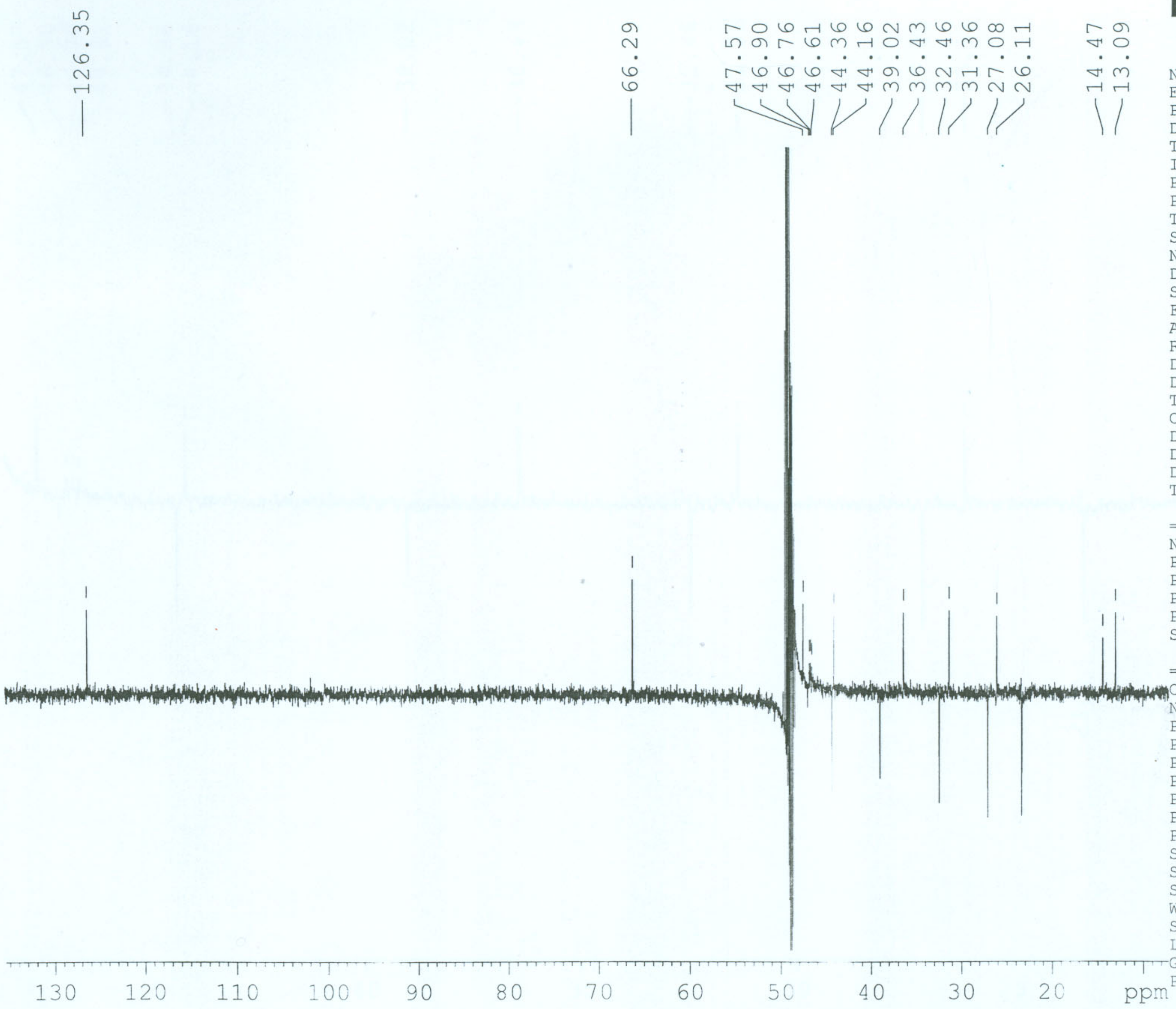

BRUKER

NAME	oct01-15	
EXPNO	4	
PROCNO	1	
Date_	20151002	
Time	6.47	
INSTRUM	spect	
PROBHD	5 mm CPDUL 13C	
PULPROG	dept135	
TD	32768	
SOLVENT	MeOD	
NS	5401	
DS	2	
SWH	25252.525	Hz
FIDRES	0.770646	Hz
AQ	0.6488762	sec
RG	32768	
DW	19.800	usec
DE	6.50	usec
TE	293.6	K
CNST2	145.0000000	
D1	1.50000000	sec
D2	0.00344828	sec
D12	0.00002000	sec
TD0	6	

======== CHANNEL f1 ========

NUC1	13C	
P1	10.30	usec
P2	20.60	usec
PL1	5.00	dB
PL1W	[illegible]1.04969788	W
SFO1	[illegible]25.8206598	MHz

======== CHANNEL f2 ========

CPDPRG2	waltz16	
NUC2	1H	
P3	16.70	usec
P4	33.40	usec
PCPD2	80.00	usec
PL2	-0.50	dB
PL12	13.11	dB
PL2W	[illegible]4.09191513	W
PL12W	[illegible].61371964	W
SFO2	[illegible]00.3330020	MHz
SI	16384	
SF	[illegible]25.8079087	MHz
WDW	EM	
SSB	0	
LB	1.00	Hz
GB	0	
PC	1.00	

Mehwish / Dr. Iqbal / JM-22-3 / MeOD
dept135
dept 90

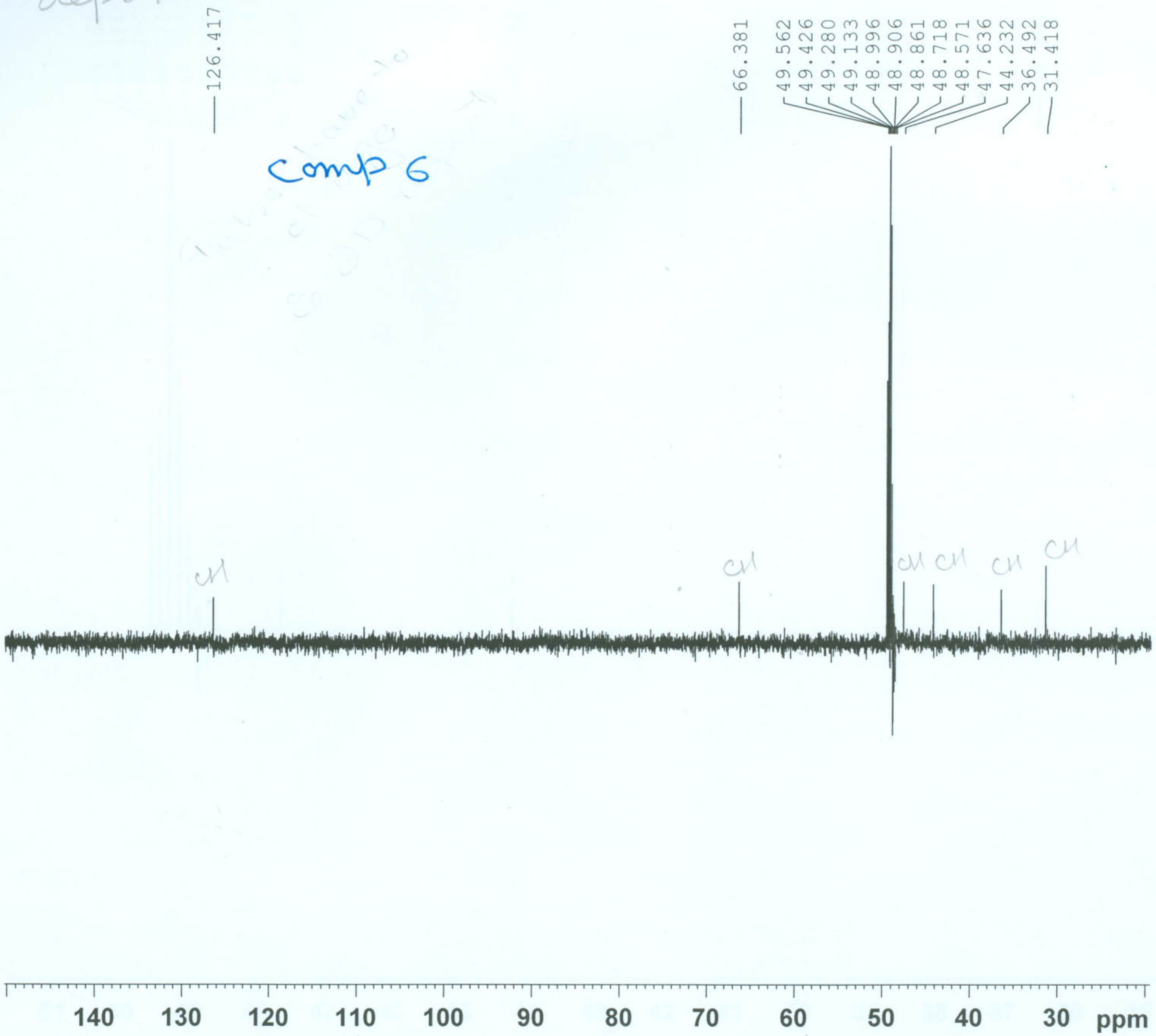

AVANCE AV-[illegible]-LC
CRYOPR[illegible]E
LAB NO:108

NAME	sep17-15	
EXPNO	9	
PROCNO	1	
Date_	20150918	
Time	9.15	
INSTRUM	spect	
PROBHD	5 mm CPTCI 1H-	
PULPROG	deptsp135	
TD	32768	
SOLVENT	MeOD	
NS	10240	
DS	2	
SWH	30303.031	Hz
FIDRES	0.924775	Hz
AQ	0.5407385	sec
RG	32768	
DW	16.500	usec
DE	6.50	usec
TE	298.1	K
CNST2	145.0000000	
D1	1.50000000	sec
D2	0.00344828	sec
D12	0.00002000	sec
TD0	10	

======== CHANNEL f1 ========

NUC1	13C	
P1	12.70	usec
P12	2000.00	usec
PL0	120.00	dB
PL1	-1.81	dB
PL0W	0.00000000	W
PL1W	81.92915344	W
SFO1	150.8927518	MHz
SP2	4.19	dB
SPNAM2	Crp60comp.4	
SPOAL2	0.500	
SPOFFS2	0.00	Hz

======== CHANNEL f2 ========

CPDPRG2	waltz16	
NUC2	1H	
P3	8.00	usec
P4	16.00	usec
PCPD2	80.00	usec
PL2	3.31	dB
PL12	23.31	dB
PL2W	6.79873323	W
PL12W	0.06798734	W
SFO2	600.0330002	MHz
SI	16384	
SF	150.8774513	MHz
WDW	EM	
SSB	0	
LB	1.00	Hz
GB	0	
PC	1.40	

Mehwish / Dr. Iqbal / Jm-22-3 / MeOD
HSQC

Comp 6

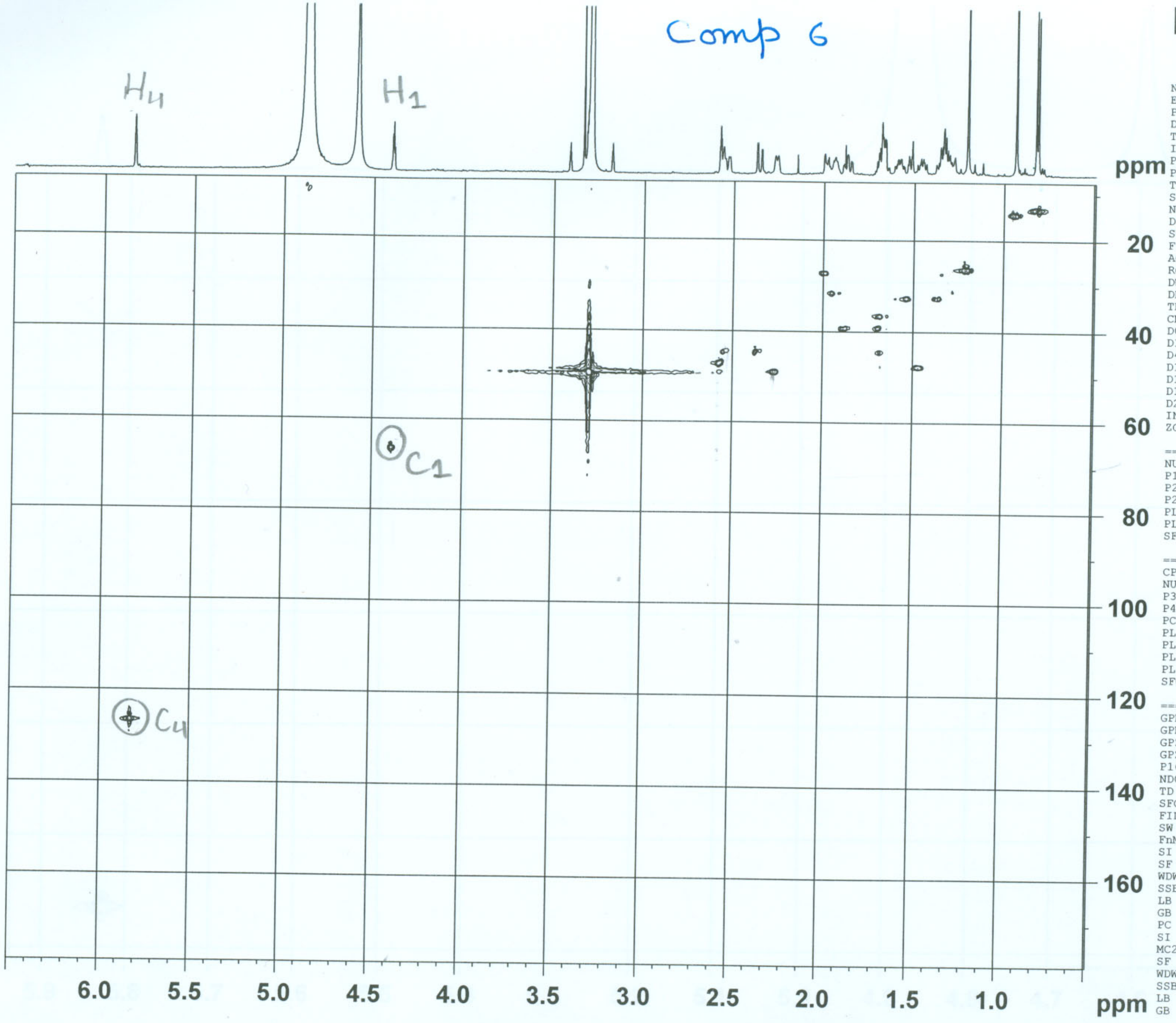

BRUKER

NAME	sep22-15	
EXPNO	8	
PROCNO	1	
Date_	20150922	
Time	20.26	
INSTRUM	spect	
PROBHD	5 mm CPTCI 1H-	
PULPROG	hsqcetgpsi	
TD	1024	
SOLVENT	MeOD	
NS	32	
DS	8	
SWH	4194.631	Hz
FIDRES	4.096319	Hz
AQ	0.1222300	sec
RG	29193	
DW	119.200	usec
DE	6.50	usec
TE	298.0	K
CNST2	145.0000000	
D0	0.00000300	sec
D1	2.00000000	sec
D4	0.00172414	sec
D11	0.03000000	sec
D13	0.00000400	sec
D16	0.00020000	sec
D24	0.00110000	sec
IN0	0.00001655	sec
ZGOPTNS		

======== CHANNEL f1 ========

NUC1	1H	
P1	8.00	usec
P2	16.00	usec
P28	1000.00	usec
PL1	3.31	dB
PL1W	6.79873323	W
SFO1	600.0321001	MHz

======== CHANNEL f2 ========

CPDPRG2	garp	
NUC2	13C	
P3	11.50	usec
P4	23.00	usec
PCPD2	55.00	usec
PL2	-1.81	dB
PL12	11.70	dB
PL2W	81.92915344	W
PL12W	3.65122390	W
SFO2	150.8927518	MHz

====== GRADIENT CHANNEL =====

GPNAM1	SINE.100	
GPNAM2	SINE.100	
GPZ1	80.00	%
GPZ2	20.10	%
P16	1000.00	usec
ND0	2	
TD	256	
SFO1	150.8928	MHz
FIDRES	117.884964	Hz
SW	200.000	ppm
FnMODE	Echo-Antiecho	
SI	1024	
SF	600.0300175	MHz
WDW	QSINE	
SSB	2	
LB	0.00	Hz
GB	0	
PC	4.00	
SI	1024	
MC2	echo-antiecho	
SF	150.8774513	MHz
WDW	QSINE	
SSB	2	
LB	0.00	Hz
GB	0	

MAHWISH / DR.IQBAL / JM-22-3' / CD3OD
HMBC

comp 6

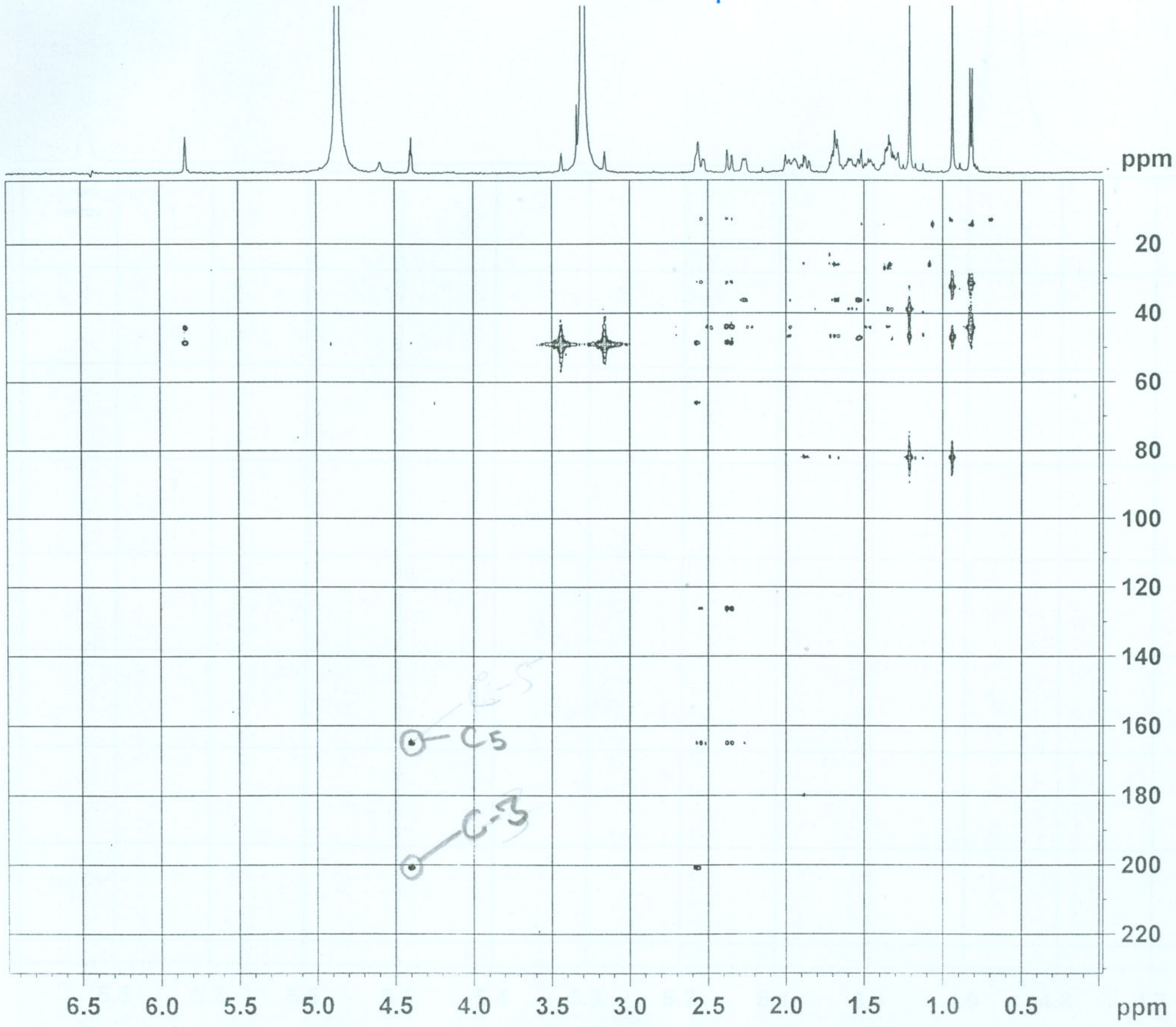

BRUKER

NAME	oct01-15	
EXPNO	3	
PROCNO	1	
Date_	20151001	
Time	20.27	
INSTRUM	spect	
PROBHD	5 mm CPDUL 13C	
PULPROG	hmbcgplpndqf	
TD	4096	
SOLVENT	MeOD	
NS	64	
DS	4	
SWH	3501.401	Hz
FIDRES	0.854834	Hz
AQ	0.5851016	sec
RG	13004	
DW	142.800	usec
DE	6.50	usec
TE	293.2	K
CNST2	145.0000000	
CNST13	13.0000000	
D0	0.00000300	sec
D1	1.50000000	sec
D2	0.00344828	sec
D6	0.03846154	sec
D16	0.00015000	sec
IN0	0.00001730	sec
======== CHANNEL f1 ========		
NUC1	1H	
P1	16.50	usec
P2	33.00	usec
PL1	-0.50	dB
PL1W	14.09191513	W
SFO1	500.3317512	MHz
======== CHANNEL f2 ========		
NUC2	13C	
P3	10.50	usec
PL2	5.00	dB
PL2W	21.04969788	W
SFO2	125.8225469	MHz
====== GRADIENT CHANNEL =====		
GPNAM1	SINE.100	
GPNAM2	SINE.100	
GPNAM3	SINE.100	
GPZ1	50.00	%
GPZ2	30.00	%
GPZ3	40.10	%
P16	2000.00	usec
ND0	2	
TD	256	
SFO1	125.8225	MHz
FIDRES	113.043694	Hz
SW	230.000	ppm
FnMODE	QF	
SI	2048	
SF	500.330014[illegible]	MHz
WDW	QSINE	
SSB	[illegible]	
LB	0.00	Hz
GB	0	
PC	4.00	
SI	1024	
MC2	QF	
SF	125.80790[illegible]	MHz
WDW	QSINE	
SSB	[illegible]	
LB	0.00	Hz
GB	[illegible]	

MAHWISH / DR.IQBAL / JM-22-3' / CD3OD
NOESY

comp 6

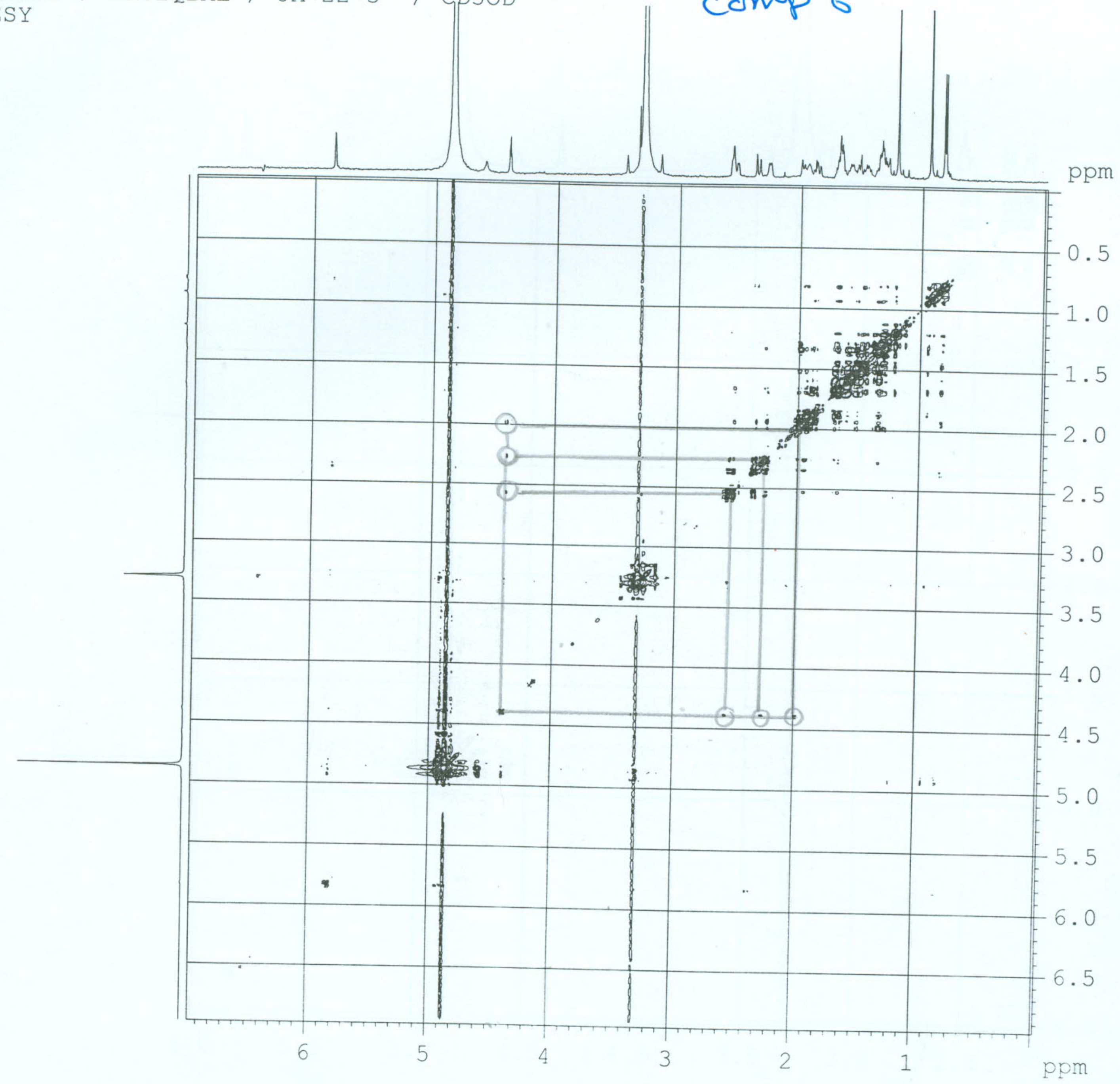

NAME	oct01-15
EXPNO	2
PROCNO	1
Date_	20151001
Time	14.29
INSTRUM	spect
PROBHD	5 mm CPDUL 13C
PULPROG	noesygpph
TD	2048
SOLVENT	MeOD
NS	32
DS	16
SWH	3501.401 Hz
FIDRES	1.709668 Hz
AQ	0.2926472 sec
RG	40.3
DW	142.800 usec
DE	6.50 usec
TE	298.1 K
D0	0.00012179 sec
D1	1.50000000 sec
D8	0.80000001 sec
D16	0.00020000 sec
IN0	0.00028560 sec

======== CHANNEL f1 ========

NUC1	1H
P1	16.50 usec
P2	33.00 usec
PL1	-0.50 dB
PL1W	14.09191513 W
SFO1	500.3317512 MHz

====== GRADIENT CHANNEL =====

GPNAM1	SINE.100
GPZ1	40.00 %
P16	1000.00 usec
ND0	1
TD	256
SFO1	500.3318 MHz
FIDRES	13.677347 Hz
SW	6.998 ppm
FnMODE	States-TPPI
SI	1024
SF	500.3300143 MHz
WDW	QSINE
SSB	2
LB	0.00 Hz
GB	0
PC	4.00
SI	1024
MC2	States-TPPI
SF	500.3300143 MHz
WDW	QSINE
SSB	2
LB	0.00 Hz
GB	0

Mehwish / Dr. Iqbal / Jm-22-3 / MeOD
cosy

comp 6

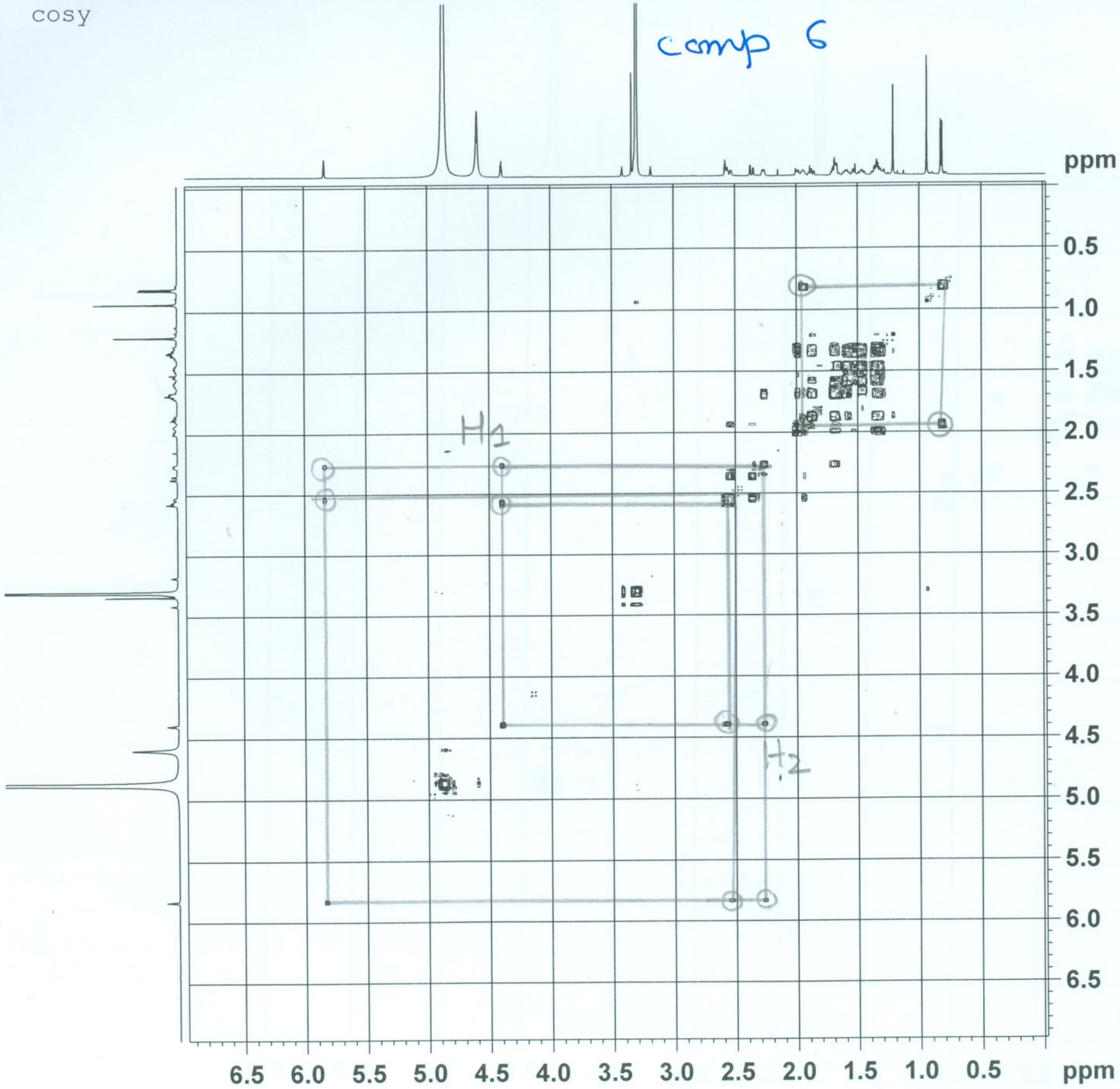

NAME	sep22-15
EXPNO	6
PROCNO	1
Date_	20150922
Time	16.16
INSTRUM	spect
PROBHD	5 mm CPTCI 1H-
PULPROG	cosydfqf
TD	2048
SOLVENT	MeOD
NS	8
DS	4
SWH	4194.631 Hz
FIDRES	2.048160 Hz
AQ	0.2442908 sec
RG	18
DW	119.200 usec
DE	6.50 usec
TE	298.0 K
D0	0.00000300 sec
D1	2.00000000 sec
D13	0.00000400 sec
D20	0.00000200 sec
IN0	0.00023840 sec

======== CHANNEL f1 ========

NUC1	1H
P1	8.00 usec
PL1	3.31 dB
PL1W	6.79873323 W
SFO1	600.0321001 MHz
ND0	1
TD	128
SFO1	600.0321 MHz
FIDRES	32.770554 Hz
SW	6.991 ppm
FnMODE	QF
SI	1024
SF	600.0300175 MHz
WDW	QSINE
SSB	0
LB	0.00 Hz
GB	0
PC	4.00
SI	1024
MC2	QF
SF	600.0300175 MHz
WDW	QSINE
SSB	0
LB	0.00 Hz
GB	0

Comp 6

THERMO ELECTRON ~ VISIONpro SOFTWARE V4.10

Operator Name	Arshad Alam	Date of Report	10/8/2015
Department	Analytical laboratory#004 TWC	Time of Report	10:46:58AM
Organization	ICCBS.Karachi University.		
Information	Porf . Dr. M. Iqbal / Mahwish.		

Scan Graph

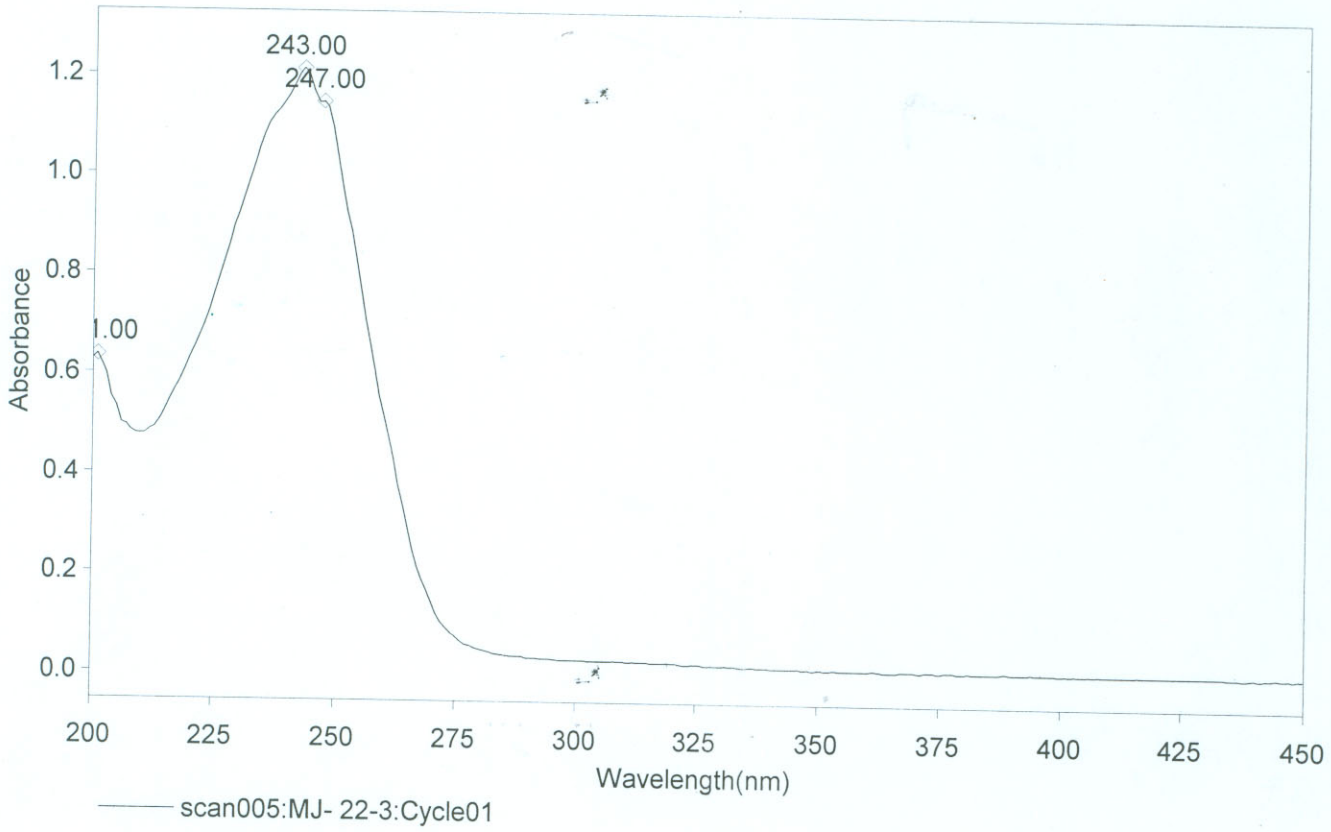

Results Table - MJ- 22-3.sre,MJ- 22-3,Cycle01

nm	A
201.00	0.636
243.00	1.212
247.00	1.146

Sensitivity Very High

Peak Pick Method
Find 8 Peaks Above -3.0000 A
Start Wavelength 200.00 nm
Stop Wavelength 250.00 nm
Sort By Wavelength

2ml ——→ 0·3ml + 2ml

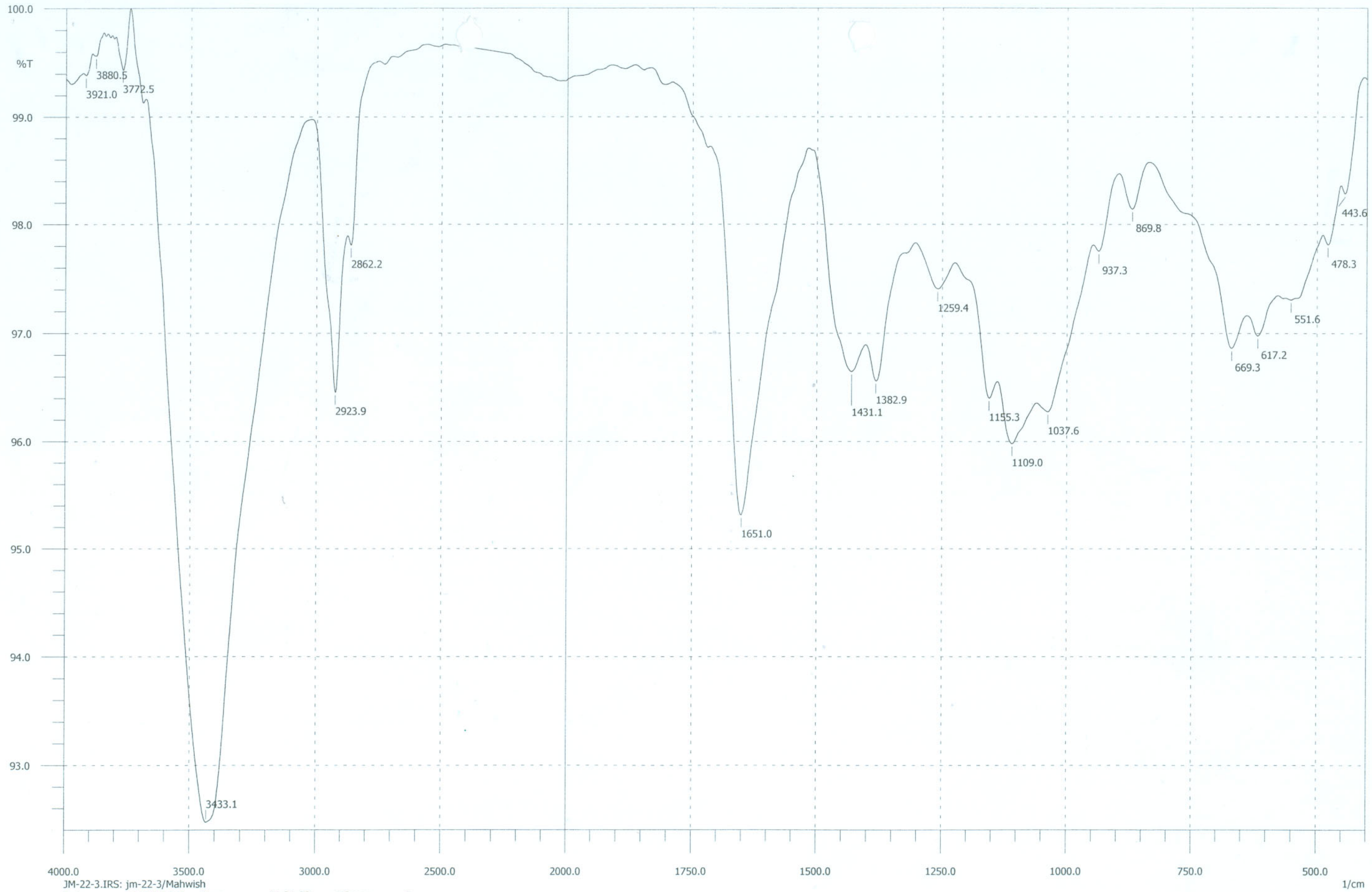

JM-22-3.IRS: jm-22-3/Mahwish

Date:	10/12/2015	Time:	11:31:08	NScans:	5
Type:	HYPER IR	User:	Zubair Ahmed	Detector:	standard
Abscissa:	1/cm	Ordinate:	%T	Apodization:	Happ
Min:	401.17	Max:	3998.16	Range:	1/cm
Ndp:	1866	Data Interval:	1.92868	Resolution:	4.0
Gain:	auto	Aperture:	auto	Mirror Speed:	2.8(low)

Compound 6
