# Supplementary material for: Biotransformation of a potent anabolic steroid, mibolerone, with Cunninghamella blakesleeana, C. echinulata, and Macrophomina phaseolina, and biological activity evaluation of its metabolites
Source: PLoS One. 2017 Feb 24;12(2):e0171476. doi: 10.1371/journal.pone.0171476 (PMC5325191; doi:10.1371/journal.pone.0171476)
Supplement: S6 Data — (PDF) [file pone.0171476.s006.pdf]

File: NU-7
Sample: MAHWISH / DR. M. IQBAL
Instrument: JEOL MS 600H-1

Date Run: 10-30-2015 (Time Run: 10:49:45)

Ionization mode: EI+

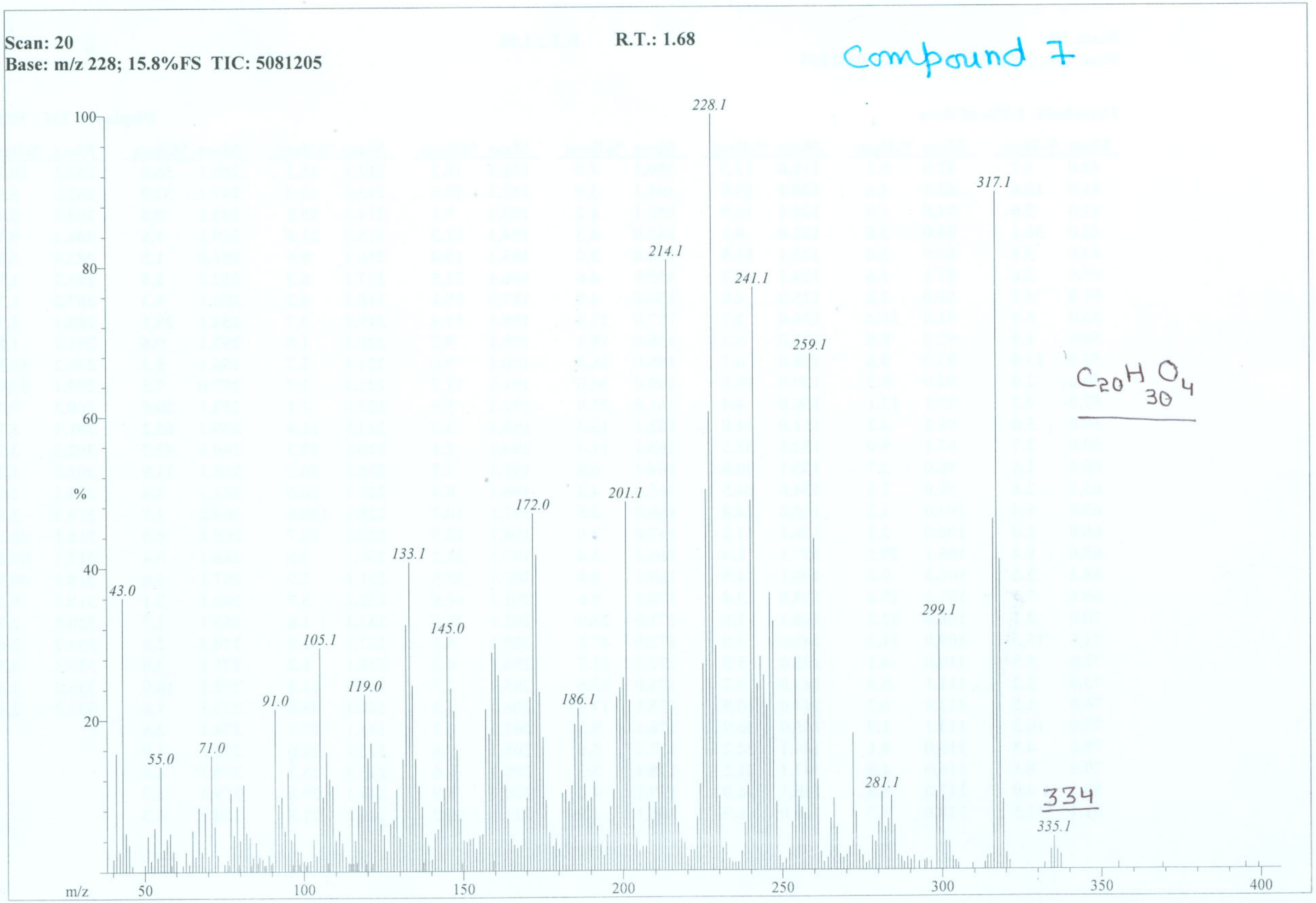

Compound 7

Mass	Relative Intensity	Theoretical Mass	Delta [ppm]	Delta [mmu]	RDB	Composition
276.1672	0.4	276.1725	-19.2	-5.3	6.0	$C_{17} H_{24} O_{3}$
276.2051	0.3	276.2089	-13.7	-3.8	5.0	$C_{18} H_{28} O_{2}$
277.1755	0.8	277.1804	-17.4	-4.8	5.5	$C_{17} H_{25} O_{3}$
278.1834	2.5	278.1882	-17.3	-4.8	5.0	$C_{17} H_{26} O_{3}$
279.1872	3.9	279.1808	23.0	6.4	0.5	$C_{13} H_{27} O_{6}$
280.1825	11.3	280.1827	-0.7	-0.2	9.0	$C_{20} H_{24} O_{1}$
		280.1886	-21.6	-6.1	0.0	$C_{13} H_{28} O_{6}$
281.1884	16.4	281.1905	-7.6	-2.1	8.5	$C_{20} H_{25} O_{1}$
282.1927	7.9	282.1984	-20.0	-5.6	8.0	$C_{20} H_{26} O_{1}$
283.1717	6.4	283.1698	6.7	1.9	8.5	$C_{19} H_{23} O_{2}$
284.1757	9.3	284.1776	-6.8	-1.9	8.0	$C_{19} H_{24} O_{2}$
285.1820	6.5	285.1855	-12.2	-3.5	7.5	$C_{19} H_{25} O_{2}$
286.1871	2.2	286.1933	-21.5	-6.1	7.0	$C_{19} H_{26} O_{2}$
287.1908	0.8	287.1858	17.4	5.0	2.5	$C_{15} H_{27} O_{5}$
288.1694	0.3	288.1725	-10.8	-3.1	7.0	$C_{18} H_{24} O_{3}$
288.2061	0.4	288.2089	-9.7	-2.8	6.0	$C_{19} H_{28} O_{2}$
289.1816	0.6	289.1804	4.3	1.2	6.5	$C_{18} H_{25} O_{3}$
289.2127	0.7	289.2168	-14.0	-4.0	5.5	$C_{19} H_{29} O_{2}$
290.1927	0.8	290.1882	15.4	4.5	6.0	$C_{18} H_{26} O_{3}$
290.2133	0.7	290.2093	13.8	4.0	1.0	$C_{15} H_{30} O_{5}$
291.1955	1.2	291.1960	-1.9	-0.5	5.5	$C_{18} H_{27} O_{3}$
292.2002	0.6	292.2038	-12.3	-3.6	5.0	$C_{18} H_{28} O_{3}$
296.1736	0.4	296.1776	-13.6	-4.0	9.0	$C_{20} H_{24} O_{2}$
297.1841	0.9	297.1855	-4.4	-1.3	8.5	$C_{20} H_{25} O_{2}$
298.1905	13.3	298.1933	-9.4	-2.8	8.0	$C_{20} H_{26} O_{2}$
299.1970	25.6	299.2011	-13.6	-4.1	7.5	$C_{20} H_{27} O_{2}$
300.2021	17.1	300.2089	-22.7	-6.8	7.0	$C_{20} H_{28} O_{2}$
		300.1937	28.1	8.4	3.0	$C_{16} H_{28} O_{5}$
301.2071	5.7	301.2015	18.6	5.6	2.5	$C_{16} H_{29} O_{5}$
302.1840	3.3	302.1882	-14.0	-4.2	7.0	$C_{19} H_{26} O_{3}$
303.1882	2.7	303.1808	24.5	7.4	2.5	$C_{15} H_{27} O_{6}$
		303.1960	-25.8	-7.8	6.5	$C_{19} H_{27} O_{3}$
304.1925	1.0	304.1886	12.8	3.9	2.0	$C_{15} H_{28} O_{6}$
314.1857	0.6	314.1882	-8.0	-2.5	8.0	$C_{20} H_{26} O_{3}$
315.1900	1.7	315.1960	-19.2	-6.0	7.5	$C_{20} H_{27} O_{3}$
		315.1808	29.2	9.2	3.5	$C_{16} H_{27} O_{6}$
316.1987	15.0	316.2038	-16.3	-5.2	7.0	$C_{20} H_{28} O_{3}$
317.2053	39.8	317.2117	-20.1	-6.4	6.5	$C_{20} H_{29} O_{3}$
		317.1964	28.0	8.9	2.5	$C_{16} H_{29} O_{6}$
318.2115	39.5	318.2042	22.8	7.3	2.0	$C_{16} H_{30} O_{6}$
		318.2195	-25.1	-8.0	6.0	$C_{20} H_{30} O_{3}$
319.2167	16.5	319.2121	14.6	4.7	1.5	$C_{16} H_{31} O_{6}$
320.2195	4.4	320.2199	-1.4	-0.4	1.0	$C_{16} H_{32} O_{6}$
		320.2140	17.0	5.4	10.0	$C_{23} H_{28} O_{1}$
321.2197	0.9	321.2218	-6.8	-2.2	9.5	$C_{23} H_{29} O_{1}$
		321.2277	-25.1	-8.1	0.5	$C_{16} H_{33} O_{6}$
322.2067	0.4	322.1992	23.4	7.5	1.0	$C_{15} H_{30} O_{7}$
		322.2144	-23.9	-7.7	5.0	$C_{19} H_{30} O_{4}$
334.2140	0.4	334.2144	-1.4	-0.5	6.0	$C_{20} H_{30} O_{4}$
335.2245	1.0	335.2222	6.9	2.3	5.5	$C_{20} H_{31} O_{4}$
336.2292	1.6	336.2301	-2.6	-0.9	5.0	$C_{20} H_{32} O_{4}$
337.2361	1.0	337.2379	-5.2	-1.7	4.5	$C_{20} H_{33} O_{4}$
338.2441	0.5	338.2457	-4.8	-1.6	4.0	$C_{20} H_{34} O_{4}$

AVANCE AV-600-LC
CRYOPROBE
LAB NO: 108

compound -7

MAHWISH / Dr. Iqbal / Nu-7 / MeOD
1H

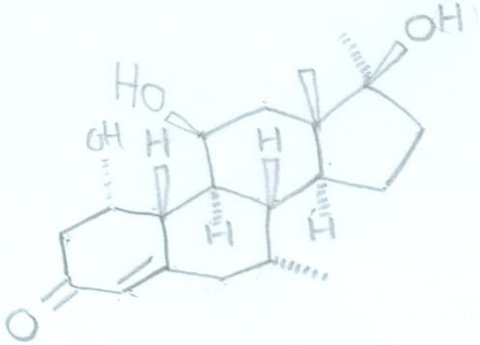

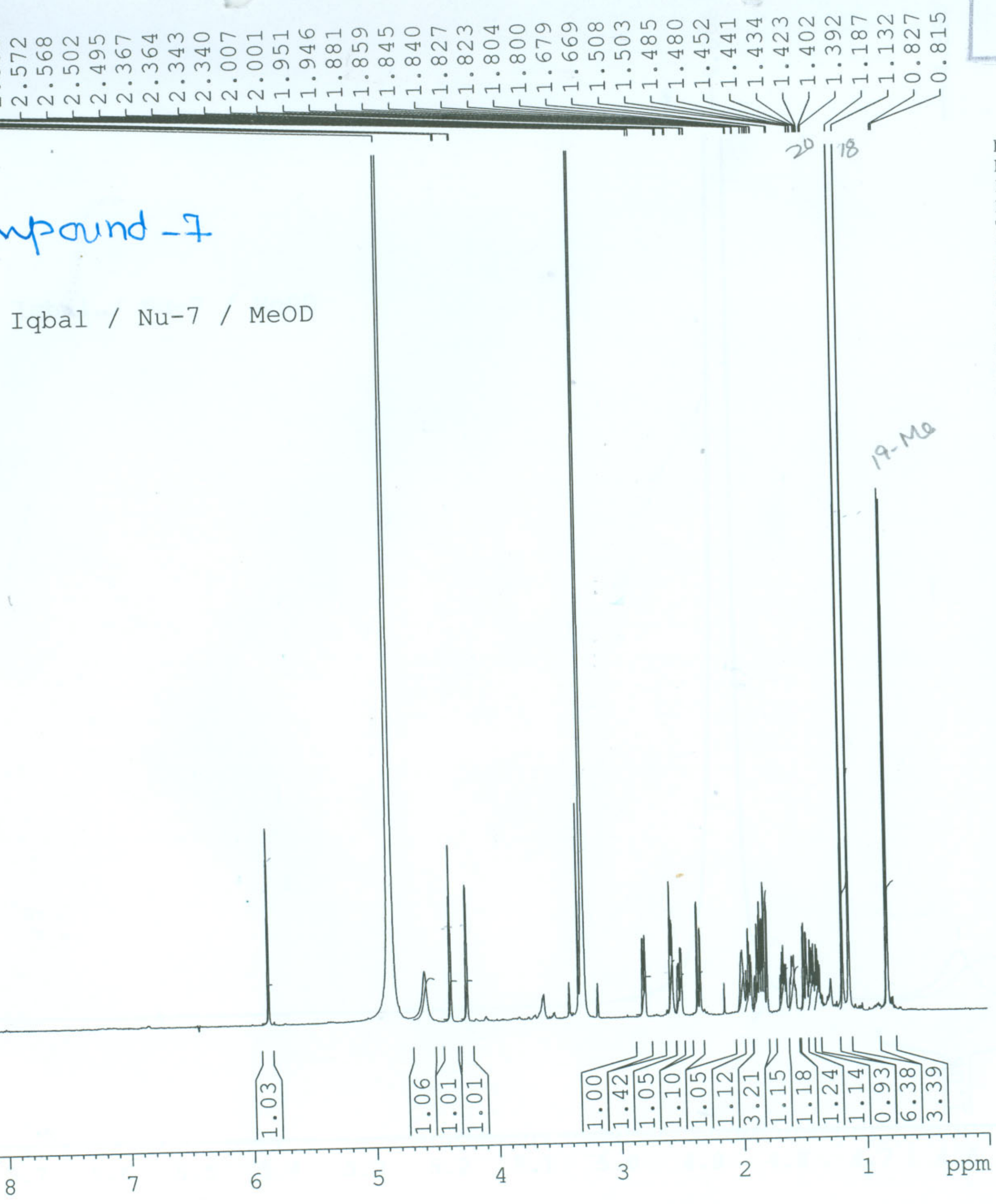

NAME	oct10-15
EXPNO	1
PROCNO	1
Date_	20151010
Time	11.03
INSTRUM	spect
PROBHD	5 mm CPTCI 1H-
PULPROG	zg30
TD	32768
SOLVENT	MeOD
NS	64
DS	0
SWH	12019.230 Hz
FIDRES	0.366798 Hz
AQ	1.3632404 sec
RG	9
DW	41.600 usec
DE	6.50 usec
TE	298.0 K
D1	2.00000000 sec
TD0	1

======== CHANNEL f1 ========

NUC1	1H
P1	8.00 usec
PL1	3.31 dB
PL1W	6.79873323 W
SFO1	600.0348002 MHz
SI	32768
SF	600.0300173 MHz
WDW	EM
SSB	0
LB	0.50 Hz
GB	0
PC	1.40

MAHWISH / Dr. Iqbal / Nu-7 / MeOD
BB

comp 7

AVANCE [illegible] LC
[illegible]
LAB [illegible]

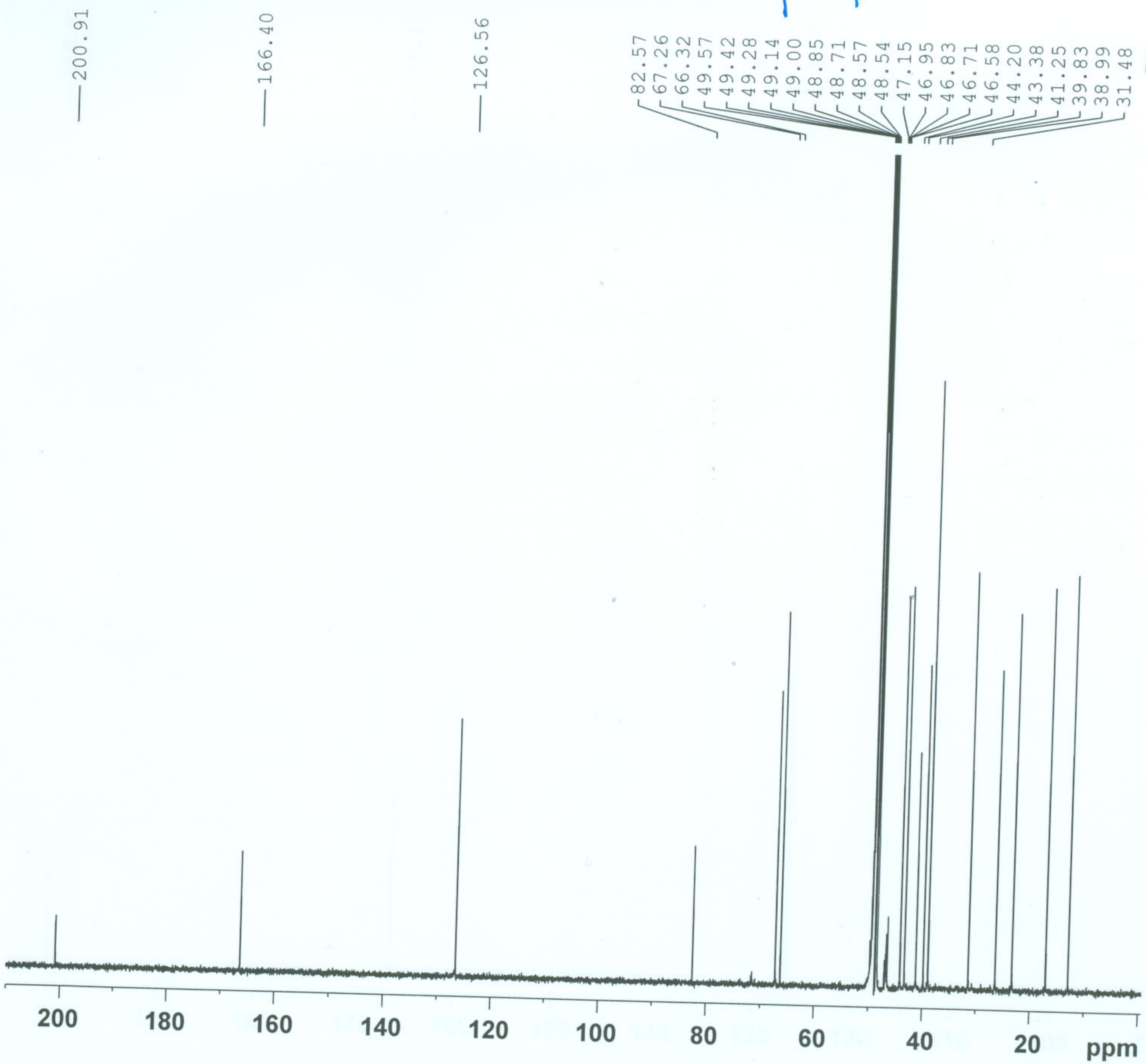

NAME	oct10-15	
EXPNO	6	
PROCNO	1	
Date_	20151011	
Time	7.55	
INSTRUM	spect	
PROBHD	5 mm CPTCI 1H-	
PULPROG	zgpg	
TD	32768	
SOLVENT	MeOD	
NS	16384	
DS	4	
SWH	35971.223	Hz
FIDRES	1.097755	Hz
AQ	0.4555391	sec
RG	32768	
DW	13.900	usec
DE	6.50	usec
TE	298.0	K
D1	2.00000000	sec
D11	0.03000000	sec
TD0	16	

======== CHANNEL f1 ========

NUC1	13C	
P1	12.70	usec
PL1	-1.81	dB
PL1W	81.92915344	W
SFO1	150.8950149	MHz

======== CHANNEL f2 ========

CPDPRG2	waltz16	
NUC2	1H	
PCPD2	80.00	usec
PL2	3.31	dB
PL12	23.31	dB
PL13	22.50	dB
PL2W	6.79873323	W
PL12W	0.06798734	W
PL13W	0.08192718	W
SFO2	600.0336002	MHz
SI	16384	
SF	150.8774513	MHz
WDW	EM	
SSB	0	
LB	1.00	Hz
GB	0	
PC	1.00	

MAHWISH / Dr. Iqbal / Nu-7 / MeOD
deptsp 90

Comp 7

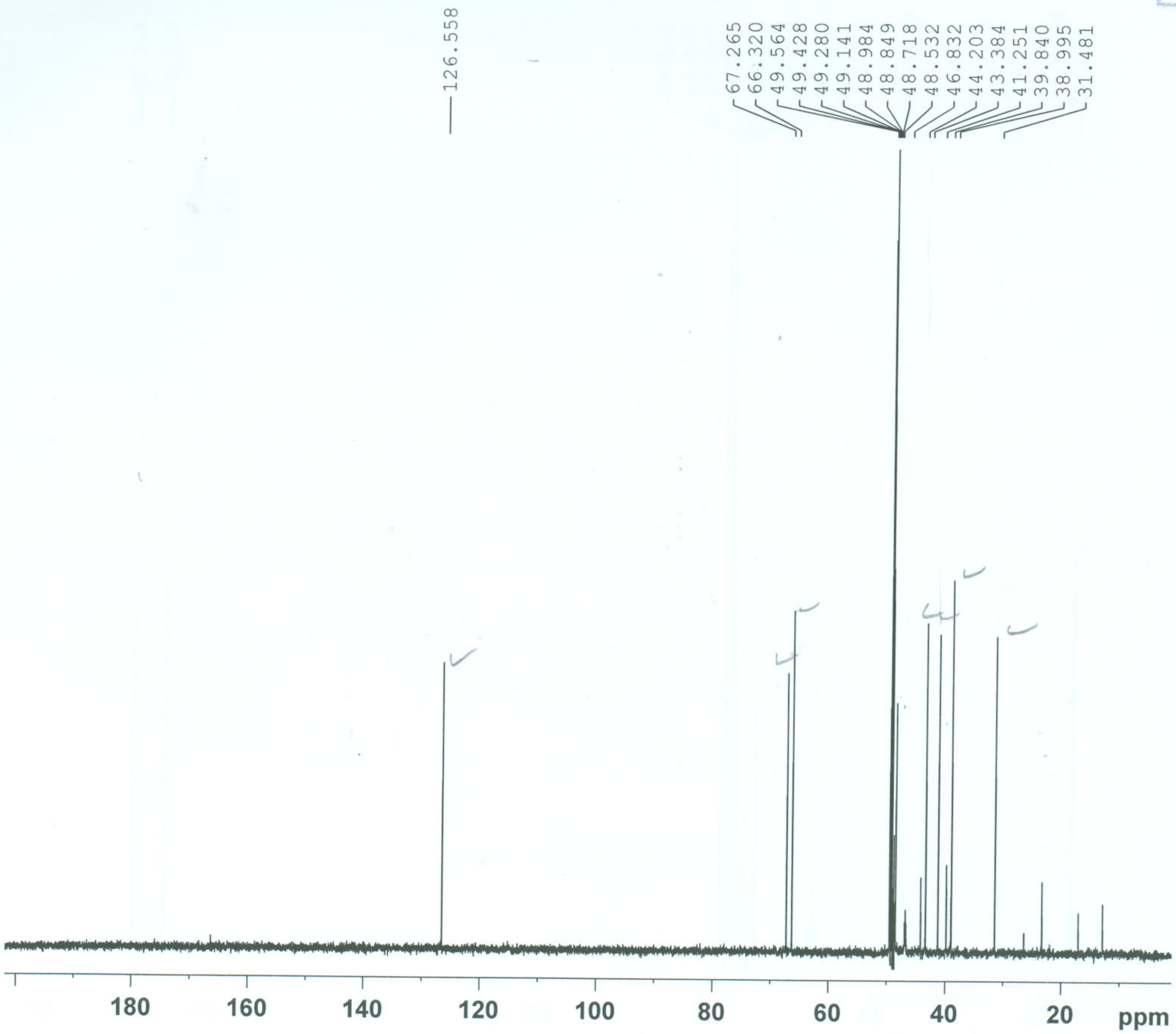

AVANCE [illegible]
CRYO[illegible]
LAB NO: 108

NAME	oct10-15
EXPNO	8
PROCNO	1
Date_	20151012
Time	0.01
INSTRUM	spect
PROBHD	5 mm CPTCI 1H-
PULPROG	deptsp90
TD	32768
SOLVENT	MeOD
NS	4096
DS	2
SWH	30303.031 Hz
FIDRES	0.924775 Hz
AQ	0.5407385 sec
RG	32768
DW	16.500 usec
DE	6.50 usec
TE	298.0 K
CNST2	145.0000000
D1	1.50000000 sec
D2	0.00344828 sec
D12	0.00002000 sec
TD0	4

======== CHANNEL f1 ========

NUC1	13C
P1	12.70 usec
P12	2000.00 usec
PL0	120.00 dB
PL1	-1.81 dB
PL0W	0.00000000 W
PL1W	81.92915344 W
SFO1	150.8927518 MHz
SP2	4.19 dB
SPNAM2	Crp60comp.4
SPOAL2	0.500
SPOFFS2	0.00 Hz

======== CHANNEL f2 ========

CPDPRG2	waltz16
NUC2	1H
P3	8.00 usec
P4	16.00 usec
PCPD2	80.00 usec
PL2	3.31 dB
PL12	23.31 dB
PL2W	6.79873323 W
PL12W	0.06798734 W
SFO2	600.0330002 MHz
SI	16384
SF	150.8774513 MHz
WDW	EM
SSB	0
LB	1.00 Hz
GB	0
PC	1.40

MAHWISH / Dr. Iqbal / Nu-7 / MeOD
dept135

Comp 7

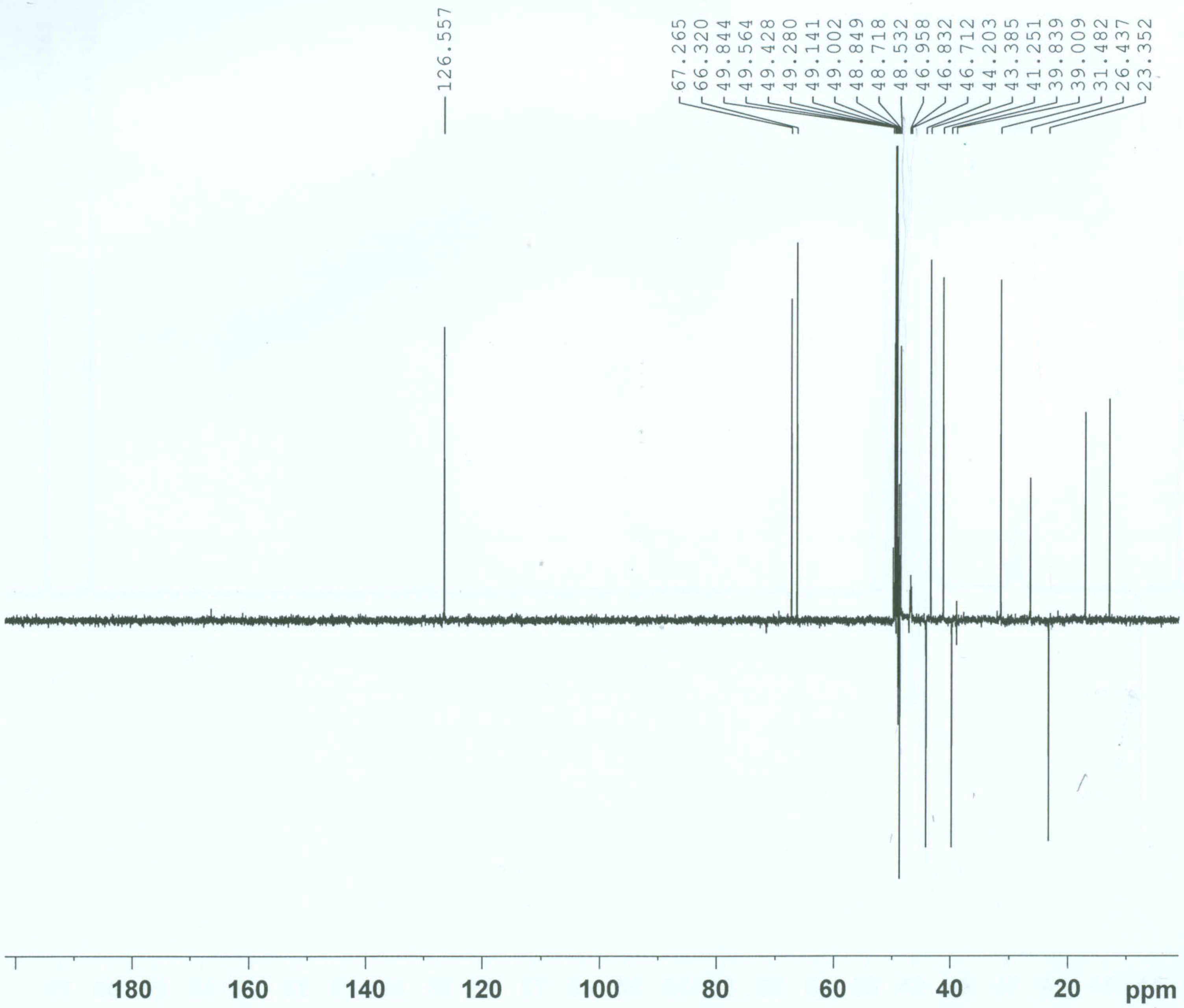

AVANCE AV-600-LC
CRYOPROBE
LAB No: 108

NAME	oct10-15	
EXPNO	7	
PROCNO	1	
Date_	20151011	
Time	19.16	
INSTRUM	spect	
PROBHD	5 mm CPTCI 1H-	
PULPROG	deptsp135	
TD	32768	
SOLVENT	MeOD	
NS	8192	
DS	2	
SWH	30303.031	Hz
FIDRES	0.924775	Hz
AQ	0.5407385	sec
RG	32768	
DW	16.500	usec
DE	6.50	usec
TE	298.0	K
CNST2	145.0000000	
D1	1.50000000	sec
D2	0.00344828	sec
D12	0.00002000	sec
TD0	8	

======== CHANNEL f1 ========

NUC1	13C	
P1	12.70	usec
P12	2000.00	usec
PL0	120.00	dB
PL1	-1.81	dB
PL0W	0.00000000	W
PL1W	81.92915344	W
SFO1	150.8927518	MHz
SP2	4.19	dB
SPNAM2	Crp60comp.4	
SPOAL2	0.500	
SPOFFS2	0.00	Hz

======== CHANNEL f2 ========

CPDPRG2	waltz16	
NUC2	1H	
P3	8.00	usec
P4	16.00	usec
PCPD2	80.00	usec
PL2	3.31	dB
PL12	23.31	dB
PL2W	6.79873323	W
PL12W	0.06798734	W
SFO2	600.0330002	MHz
SI	16384	
SF	150.8774513	MHz
WDW	EM	
SSB	0	
LB	1.00	Hz
GB	0	
PC	1.40	

MAHWISH / Dr. Iqbal / Nu-7 / MeOD
HSQC

comp 7

AVANCE AV-600-LC
CRY[illegible]ODE
LAB NO: 108

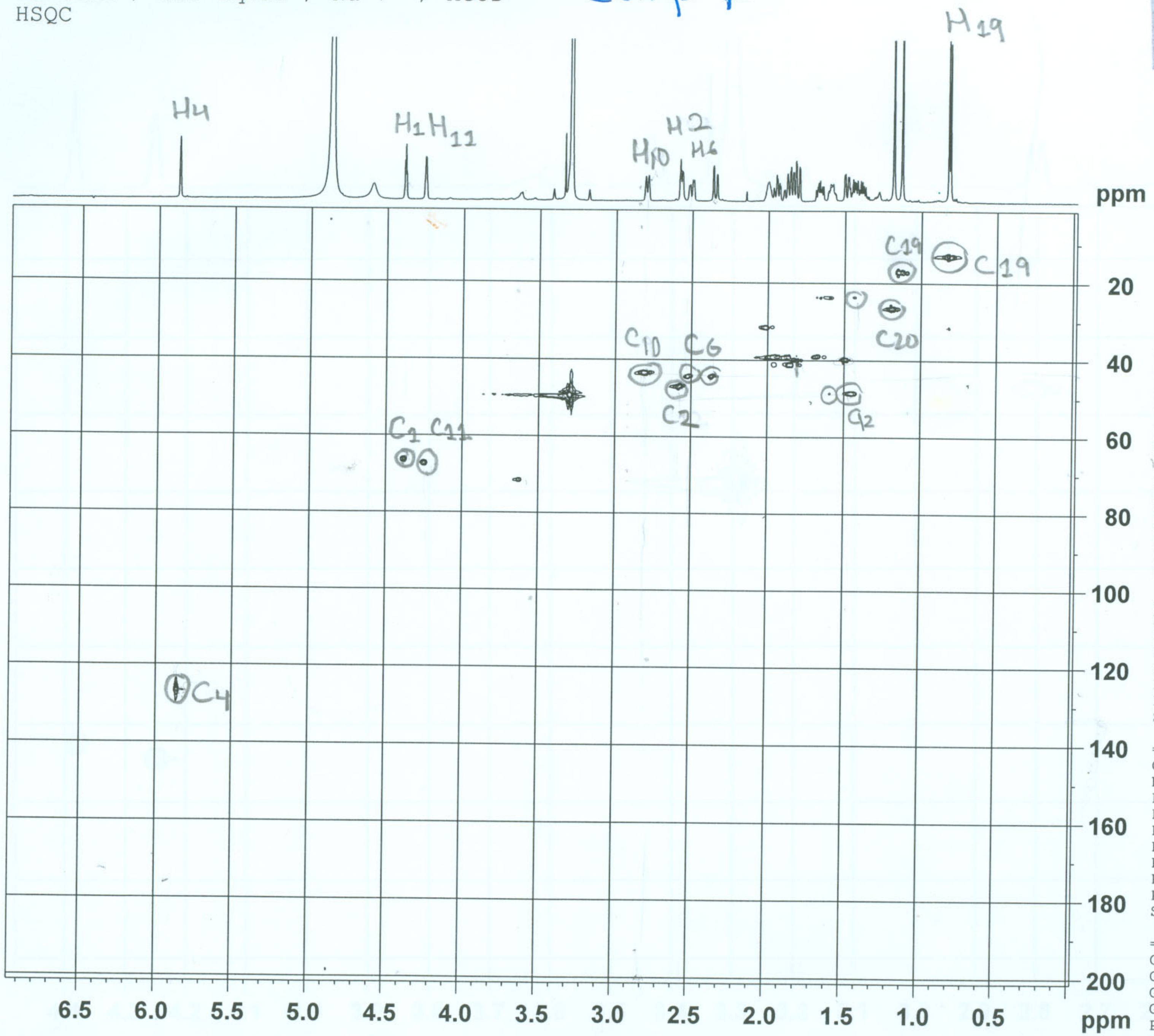

NAME	oct10-15
EXPNO	4
PROCNO	1
Date_	20151010
Time	15.51
INSTRUM	spect
PROBHD	5 mm CPTCI 1H-
PULPROG	hsqcetgpsi
TD	1024
SOLVENT	MeOD
NS	32
DS	8
SWH	4194.631 Hz
FIDRES	4.096319 Hz
AQ	0.1222300 sec
RG	46341
DW	119.200 usec
DE	6.50 usec
TE	298.0 K
CNST2	145.0000000
D0	0.00000300 sec
D1	2.00000000 sec
D4	0.00172414 sec
D11	0.03000000 sec
D13	0.00000400 sec
D16	0.00020000 sec
D24	0.00110000 sec
IN0	0.00001655 sec
ZGOPTNS	

======== CHANNEL f1 ========

NUC1	1H
P1	8.00 usec
P2	16.00 usec
P28	1000.00 usec
PL1	3.31 dB
PL1W	6.79873323 W
SFO1	600.0321001 MHz

======== CHANNEL f2 ========

CPDPRG2	garp
NUC2	13C
P3	11.50 usec
P4	23.00 usec
PCPD2	55.00 usec
PL2	-1.81 dB
PL12	11.70 dB
PL2W	81.92915344 W
PL12W	3.65122390 W
SFO2	150.8927518 MHz

====== GRADIENT CHANNEL =====

GPNAM1	SINE.100
GPNAM2	SINE.100
GPZ1	80.00 %
GPZ2	20.10 %
P16	1000.00 usec
ND0	2

Canada

MAHWISH / Dr. Iqbal / Nu-7 / MeOD
HMBC

compound 7

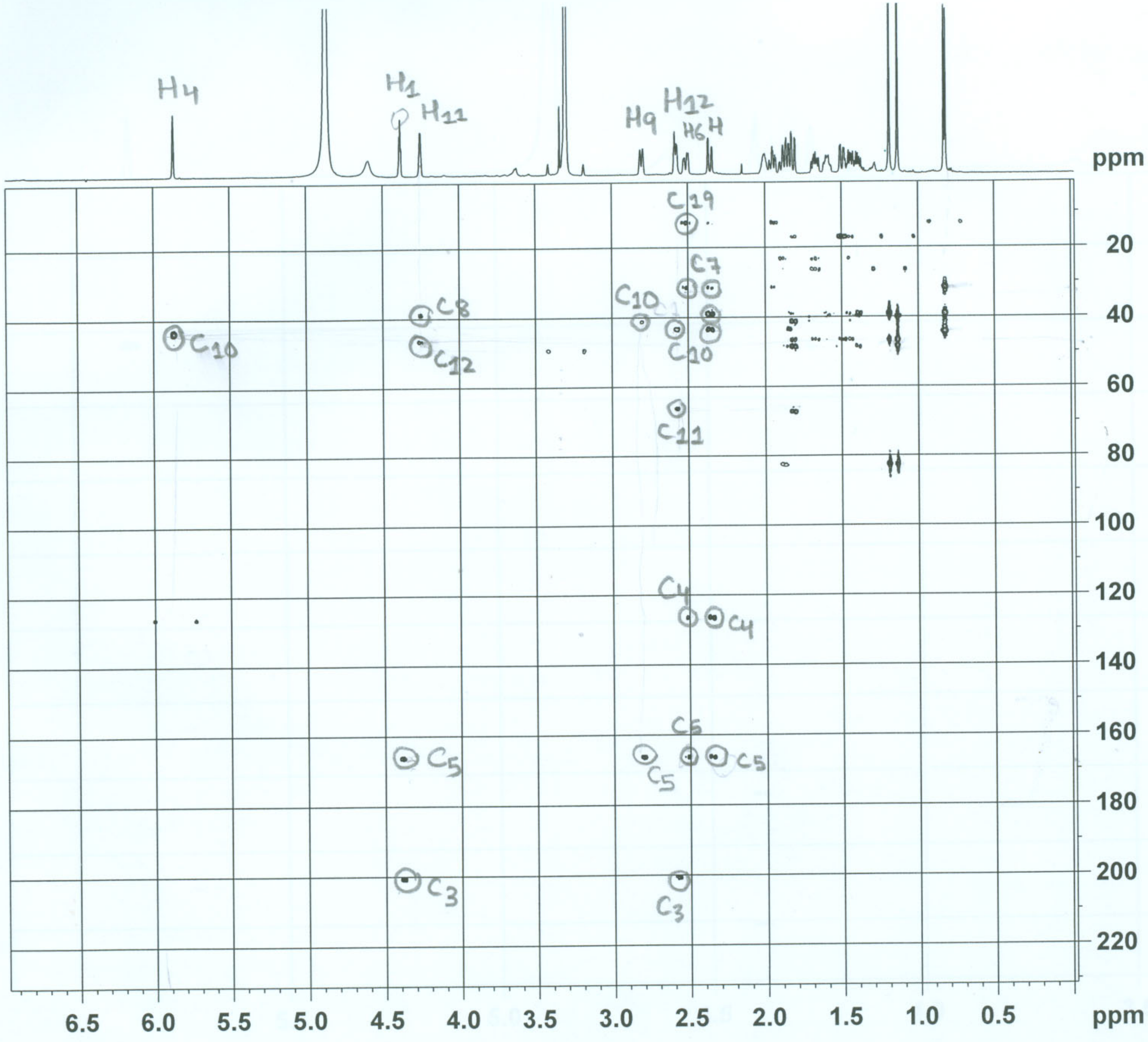

AVANCE AV-600-LC
CRYOPROBE
LAB NO: 108

NAME	oct10-15	
EXPNO	5	
PROCNO	1	
Date_	20151010	
Time	20.46	
INSTRUM	spect	
PROBHD	5 mm CPTCI 1H-	
PULPROG	hmbcgplpndqf	
TD	2048	
SOLVENT	MeOD	
NS	64	
DS	16	
SWH	4194.631	Hz
FIDRES	2.048160	Hz
AQ	0.2442908	sec
RG	41285.1	
DW	119.200	usec
DE	6.50	usec
TE	298.0	K
CNST2	145.0000000	
CNST13	13.0000000	
D0	0.00000300	sec
D1	2.00000000	sec
D2	0.00344828	sec
D6	0.03846154	sec
D16	0.00015000	sec
IN0	0.00001440	sec

======== CHANNEL f1 ========

NUC1	1H	
P1	8.00	usec
P2	16.00	usec
PL1	3.31	dB
PL1W	6.79873323	W
SFO1	600.0321001	MHz

======== CHANNEL f2 ========

NUC2	13C	
P3	11.50	usec
PL2	-1.81	dB
PL2W	81.92915344	W
SFO2	150.8950149	MHz

====== GRADIENT CHANNEL =====

GPNAM1	SINE.100	
GPNAM2	SINE.100	
GPNAM3	SINE.100	
GPZ1	50.00	%
GPZ2	30.00	%
GPZ3	40.10	%
P16	2000.00	usec
ND0	2	
TD	256	
SFO1	150.895	MHz
FIDRES	135.569733	Hz
SW	230.000	ppm
FnMODE	QF	
SI	2048	

MAHWISH / Dr. Iqbal / Nu-7 / MeOD
cosy

comp 7

LAB NO: 108

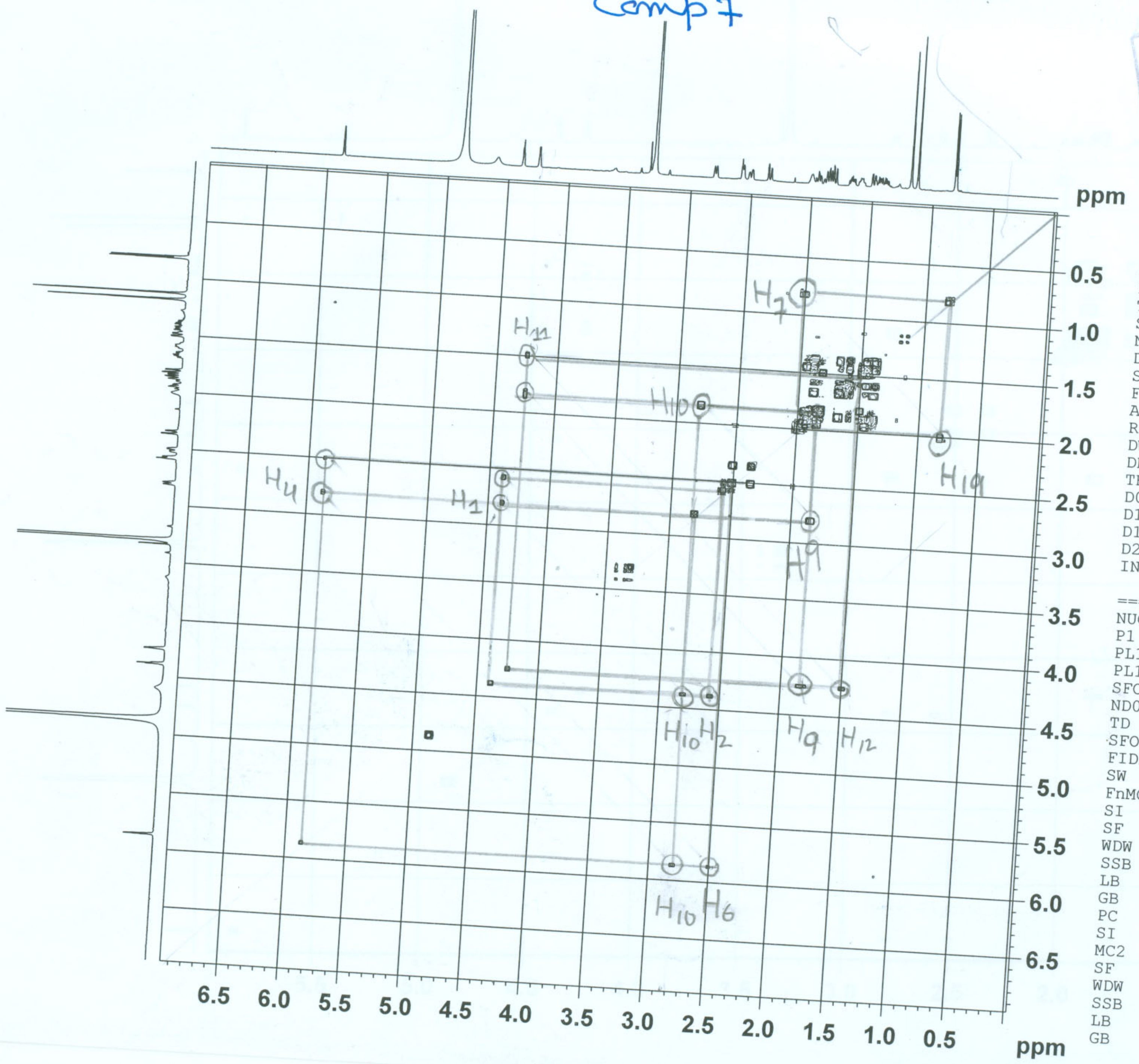

NAME	oct10-15
EXPNO	2
PROCNO	1
Date_	20151010
Time	11.04
INSTRUM	spect
PROBHD	5 mm CPTCI 1H-
PULPROG	cosydfqf
TD	2048
SOLVENT	MeOD
NS	16
DS	4
SWH	4194.631 Hz
FIDRES	2.048160 Hz
AQ	0.2442908 sec
RG	16
DW	119.200 usec
DE	6.50 usec
TE	298.0 K
D0	0.00000300 sec
D1	2.00000000 sec
D13	0.00000400 sec
D20	0.00000200 sec
IN0	0.00023840 sec

======== CHANNEL f1 ========

NUC1	1H
P1	8.00 usec
PL1	3.31 dB
PL1W	6.79873323 W
SFO1	600.0321001 MHz
ND0	1
TD	128
SFO1	600.0321 MHz
FIDRES	32.770554 Hz
SW	6.991 ppm
FnMODE	QF
SI	1024
SF	600.0300173 MHz
WDW	QSINE
SSB	0
LB	0.00 Hz
GB	0
PC	4.00
SI	1024
MC2	QF
SF	600.0300173 MHz
WDW	QSINE
SSB	0
LB	0.00 Hz
GB	0

Comp 7

MAHWISH / Dr. Iqbal / Nu-7 / MeOD
NOESY

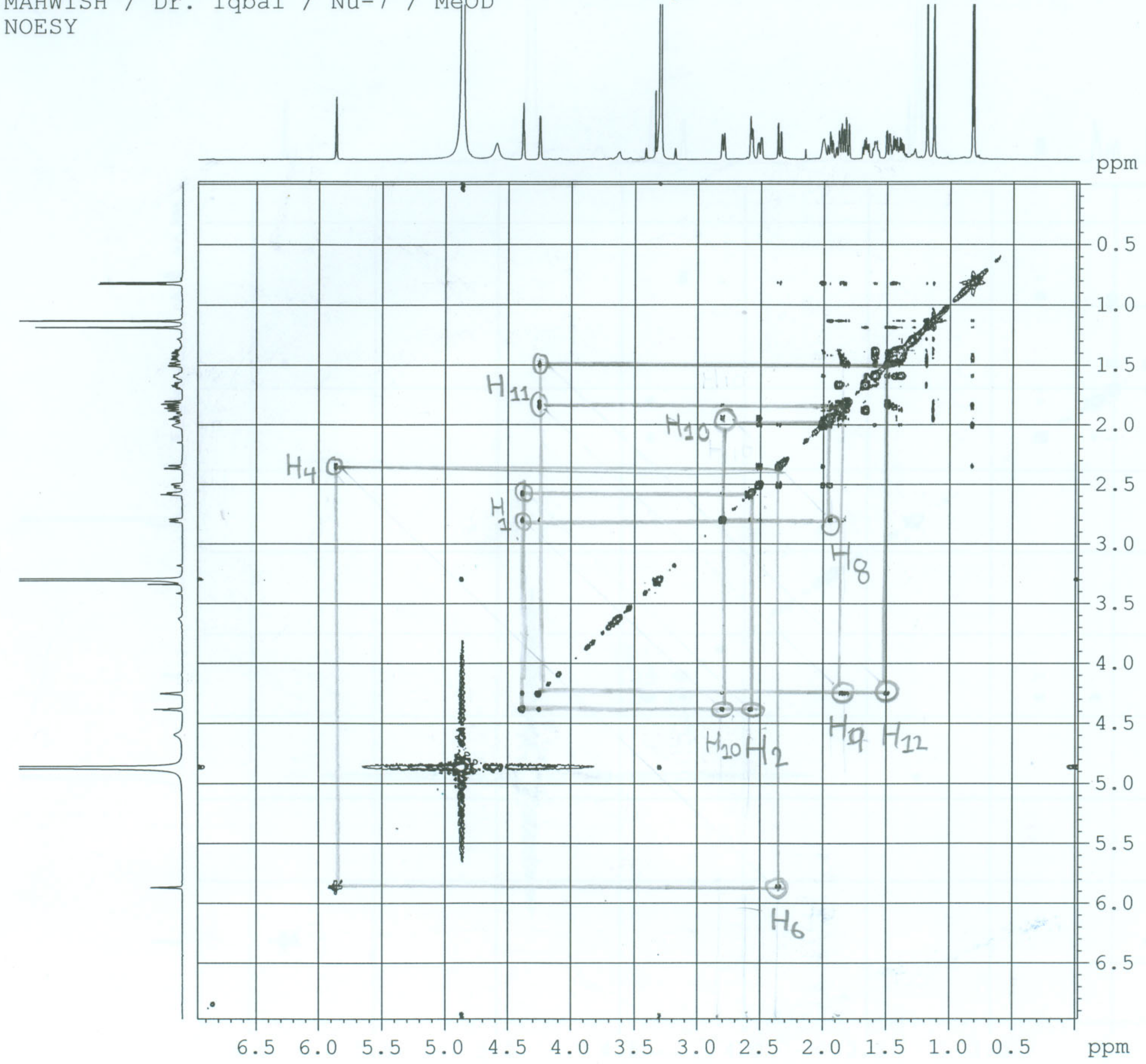

NAME	oct10-15	
EXPNO	3	
PROCNO	1	
Date_	20151010	
Time	12.21	
INSTRUM	spect	
PROBHD	5 mm CPTCI 1H-	
PULPROG	noesygpph	
TD	2048	
SOLVENT	MeOD	
NS	16	
DS	4	
SWH	4194.631	Hz
FIDRES	2.048160	Hz
AQ	0.2442908	sec
RG	25.4	
DW	119.200	usec
DE	6.50	usec
TE	298.0	K
D0	0.00010901	sec
D1	2.00000000	sec
D8	0.80000001	sec
D16	0.00020000	sec
IN0	0.00023840	sec
======== CHANNEL f1 ========		
NUC1	1H	
P1	8.00	usec
P2	16.00	usec
PL1	3.31	dB
PL1W	6.79873323	W
SFO1	600.0321001	MHz
====== GRADIENT CHANNEL =====		
GPNAM1	SINE.100	
GPNAM2	SINE.100	
GPZ1	40.00	%
GPZ2	-40.00	%
P16	1000.00	usec
ND0	1	
TD	256	
SFO1	600.0321	MHz
FIDRES	16.385277	Hz
SW	6.991	ppm
FnMODE	States-TPPI	
SI	1024	
SF	600.0300173	MHz
WDW	SINE	
SSB	2	
LB	0.00	Hz
GB	0	
PC	4.00	
SI	512	
MC2	States-TPPI	
SF	600.0300173	MHz
WDW	SINE	
SSB	2	
LB	0.00	Hz
GB	0	

Comp - 7

THERMO ELECTRON ~ VISIONpro SOFTWARE V4.10

Operator Name	Arshad Alam	Date of Report	11/6/2015
Department	Analytical laboratory#004 TWC	Time of Report	8:58:39AM
Organization	ICCBS.Karachi University.		
Information	Porf Dr. M.Iqbal /Mahwish.		

Scan Graph

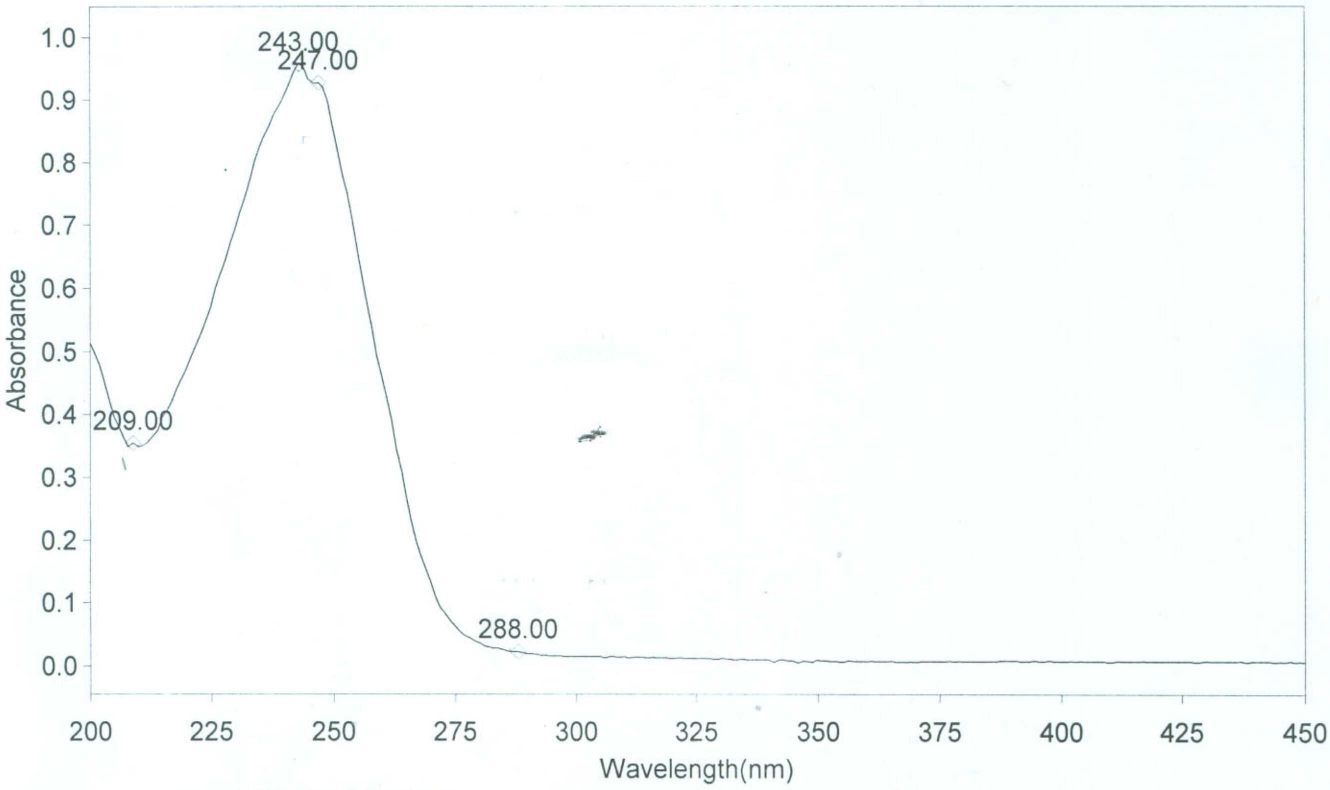

Results Table - NU-7.sre,NU-7,Cycle01

nm	A
209.00	0.355
243.00	0.957
247.00	0.927
288.00	0.022
Sensitivity	Manual
Rising Points	1
Falling Points	1
Min. Change	0.0000

Peak Pick Method
Find 8 Peaks Above -3.0000 A
Start Wavelength 200.00 nm
Stop Wavelength 290.00 nm
Sort By Wavelength

2 ml → 0.05 ml + 2 ml

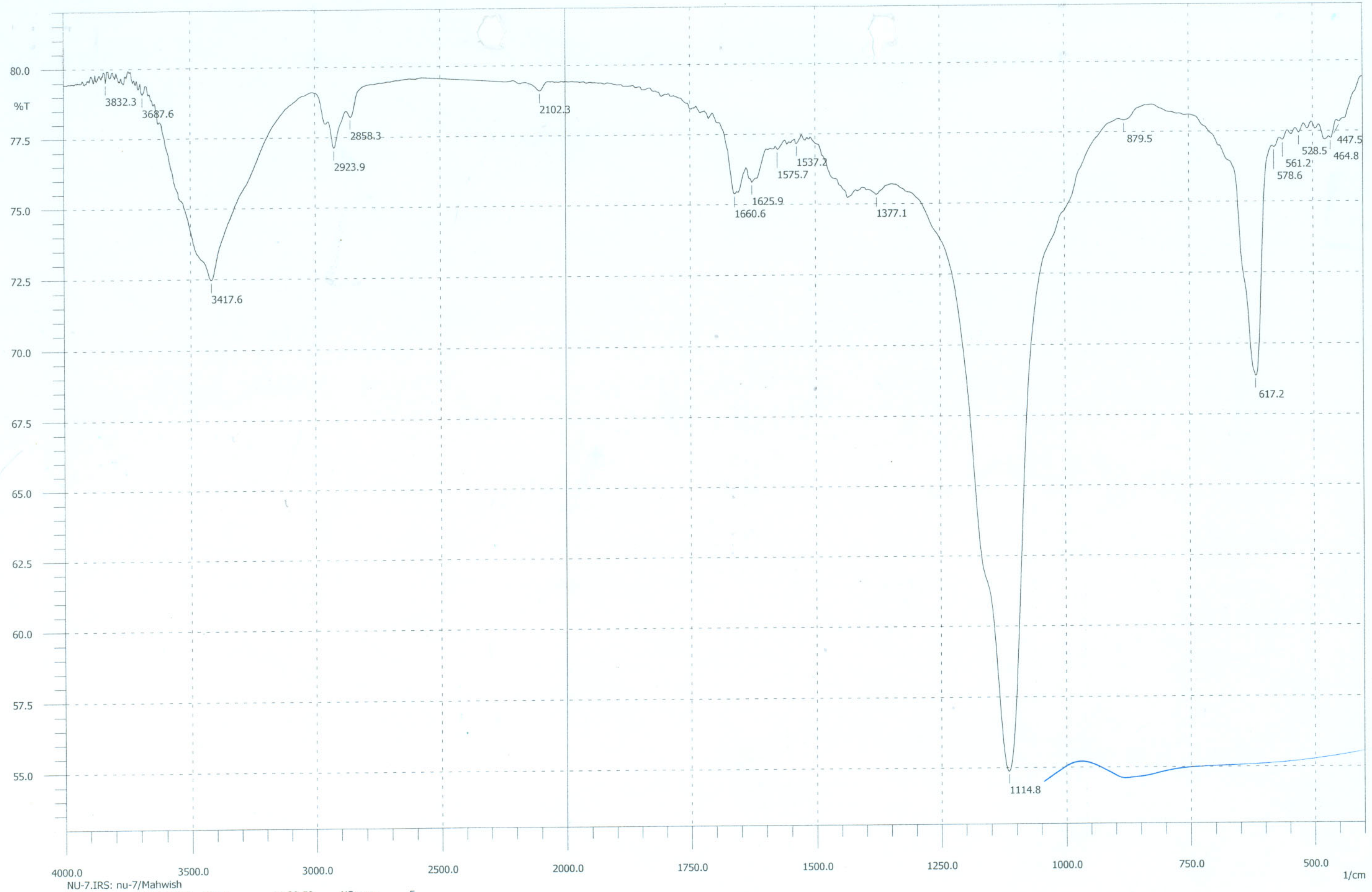

NU-7.IRS: nu-7/Mahwish

Date:	10/12/2015	Time:	11:29:58	NScans:	5
Type:	HYPER IR	User:	Zubair Ahmed	Detector:	standard
Abscissa:	1/cm	Ordinate:	%T	Apodization:	Happ
Min:	401.17	Max:	3998.16	Range:	1/cm
Ndp:	1866	Data Interval:	1.92868	Resolution:	4.0
Gain:	auto	Aperture:	auto	Mirror Speed:	2.8(low)

comp - 7
