# Supplementary material for: Biotransformation of a potent anabolic steroid, mibolerone, with Cunninghamella blakesleeana, C. echinulata, and Macrophomina phaseolina, and biological activity evaluation of its metabolites
Source: PLoS One. 2017 Feb 24;12(2):e0171476. doi: 10.1371/journal.pone.0171476 (PMC5325191; doi:10.1371/journal.pone.0171476)
Supplement: S7 Data — (PDF) [file pone.0171476.s007.pdf]

10/30/2015 10:33:20 A[illegible]

File: JM-8
Sample: MAHWISH / DR. M. IQBAL
Instrument: JEOL MS 600H-1

Date Run: 10-30-2015 (Time Run: 10:27:34)

Ionization mode: EI+

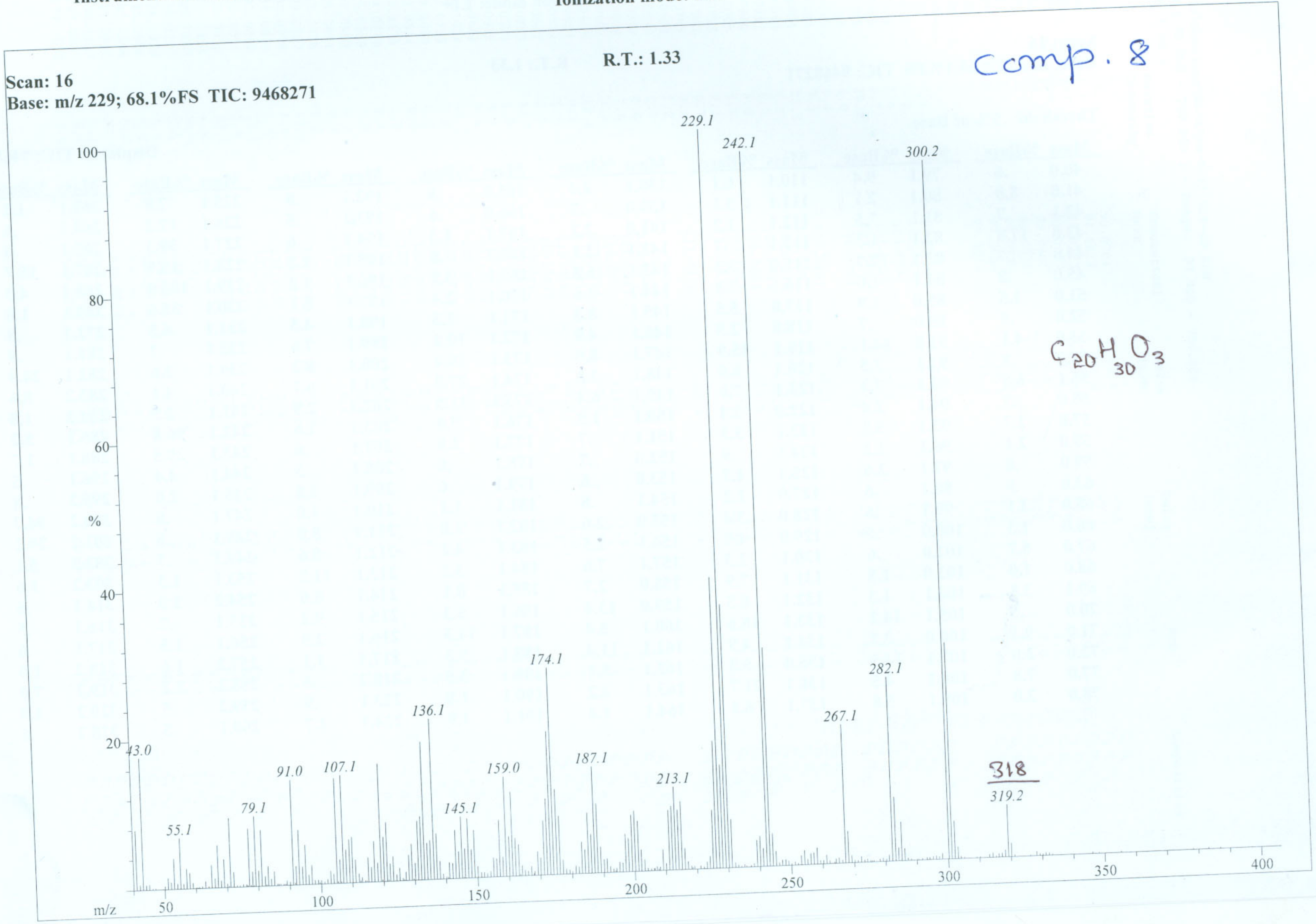

Comp. 8

| Mass | Relative Intensity | Theoretical Mass | Delta [ppm] | Delta [mmu] | RDB | Composition |
|---|---|---|---|---|---|---|
| 303.2009 | 0.8 | 303.1960 | 16.2 | 4.9 | 6.5 | $C_{19} H_{27} O_3$ |
| 316.2041 | 0.5 | 316.2038 | 0.9 | 0.3 | 7.0 | $C_{20} H_{28} O_3$ |
| 318.2211 | 0.6 | 318.2195 | 5.2 | 1.6 | 6.0 | $C_{20} H_{30} O_3$ |
| 319.2267 | 0.4 | 319.2273 | -1.9 | -0.6 | 5.5 | $C_{20} H_{31} O_3$ |

comp. 8

MAHWISH/DR.IQBAL/JM.8
1H

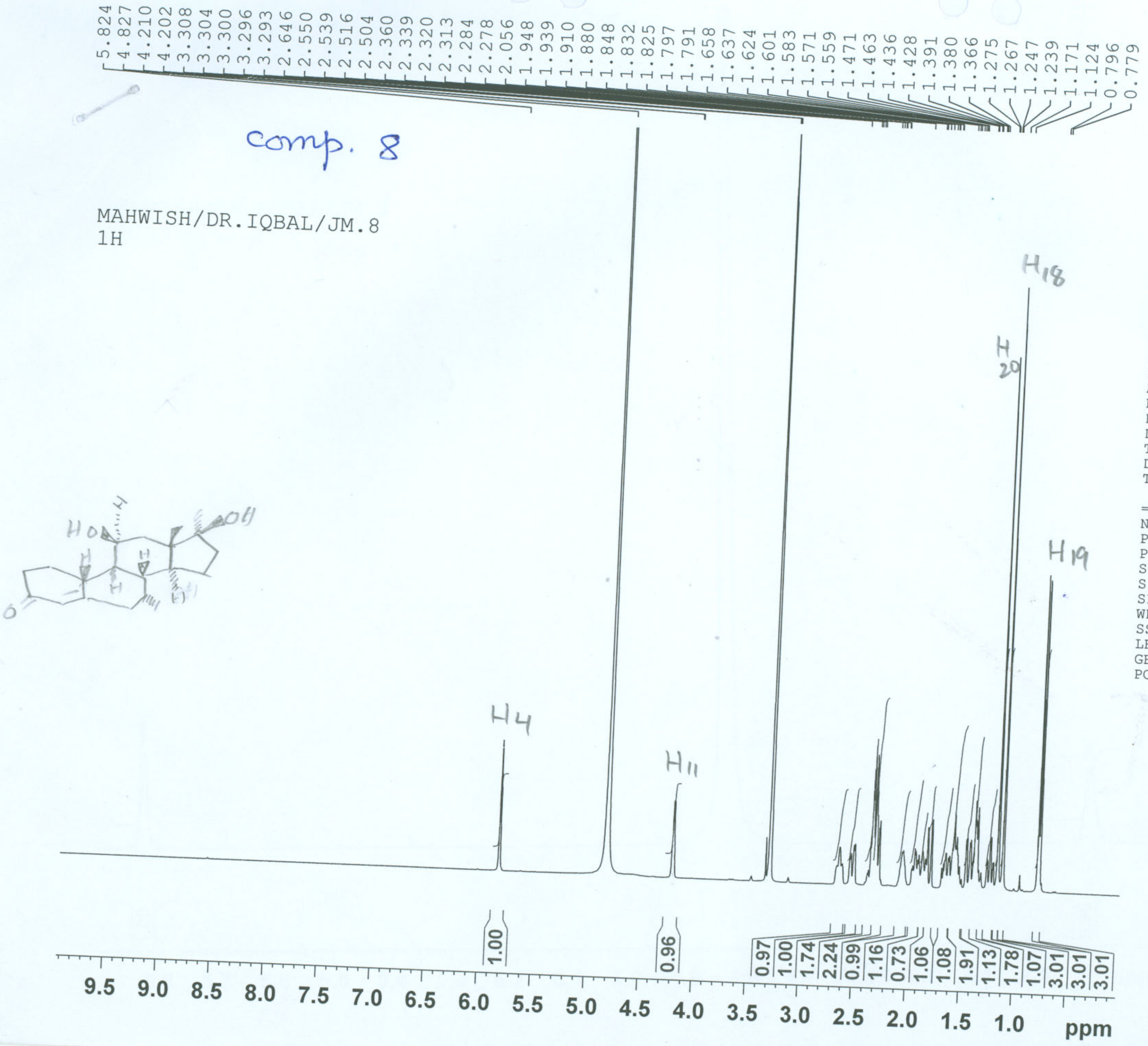


AVANCE AV-400 MHz
Lab # 115

| | |
|---|---|
| NAME | sep15-15 |
| EXPNO | 17 |
| PROCNO | 1 |
| Date_ | 20150915 |
| Time | 15.48 |
| INSTRUM | spect |
| PROBHD | 5 mm SEI 1H-13 |
| PULPROG | zg30 |
| TD | 65536 |
| SOLVENT | MeOD |
| NS | 128 |
| DS | 0 |
| SWH | 8012.820 Hz |
| FIDRES | 0.122266 Hz |
| AQ | 4.0894966 sec |
| RG | 256 |
| DW | 62.400 usec |
| DE | 6.50 usec |
| TE | 300.0 K |
| D1 | 2.00000000 sec |
| TD0 | 1 |

======== CHANNEL f1 ========

| | |
|---|---|
| NUC1 | 1H |
| P1 | 10.80 usec |
| PL1 | 3.00 dB |
| SFO1 | 400.0332002 MHz |
| SI | 32768 |
| SF | 400.0300087 MHz |
| WDW | EM |
| SSB | 0 |
| LB | 0.30 Hz |
| GB | 0 |
| PC | 1.00 |

MEHWISH/DR,IQBAL/JM-8/
ICCBS,U.O.K/BB

AVANCE 400
LAB NO 117

Comp. 8

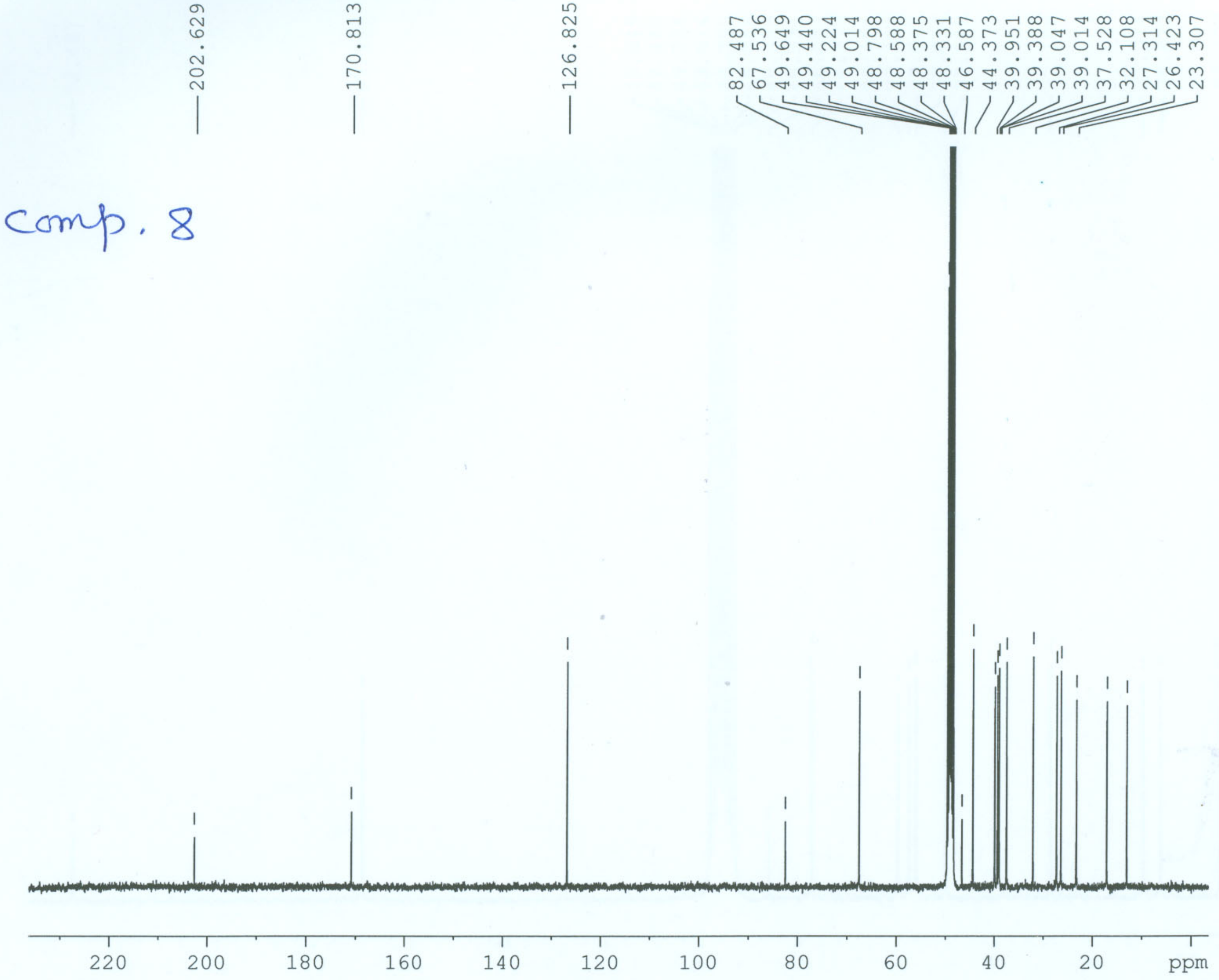


| | |
|---|---|
| NAME | sep17-15 |
| EXPNO | 9 |
| PROCNO | 1 |
| Date_ | 20150917 |
| Time | 11.40 |
| INSTRUM | spect |
| PROBHD | 5 mm DUL 13C-1 |
| PULPROG | zgpg |
| TD | 32768 |
| SOLVENT | MeOD |
| NS | 18432 |
| DS | 2 |
| SWH | 24154.590 Hz |
| FIDRES | 0.737140 Hz |
| AQ | 0.6783476 sec |
| RG | 32768 |
| DW | 20.700 usec |
| DE | 6.50 usec |
| TE | 300.0 K |
| D1 | 1.50000000 sec |
| D11 | 0.03000000 sec |
| TD0 | 18 |

======== CHANNEL f1 ========

| | |
|---|---|
| NUC1 | 13C |
| P1 | 8.10 usec |
| PL1 | 7.00 dB |
| SFO1 | 100.6243395 MHz |

======== CHANNEL f2 ========

| | |
|---|---|
| CPDPRG2 | waltz16 |
| NUC2 | 1H |
| PCPD2 | 80.00 usec |
| PL2 | 0.00 dB |
| PL12 | 20.00 dB |
| PL13 | 22.00 dB |
| SFO2 | 400.1324008 MHz |
| SI | 16384 |
| SF | 100.6126253 MHz |
| WDW | EM |
| SSB | 0 |
| LB | 1.50 Hz |
| GB | 0 |
| PC | 1.00 |

MEHWISH/DR,IQBAL/JM-8/
ICCBS,U.O.KDEPT-90/

Comp. 8

AVANCE 400
LAB NO 117

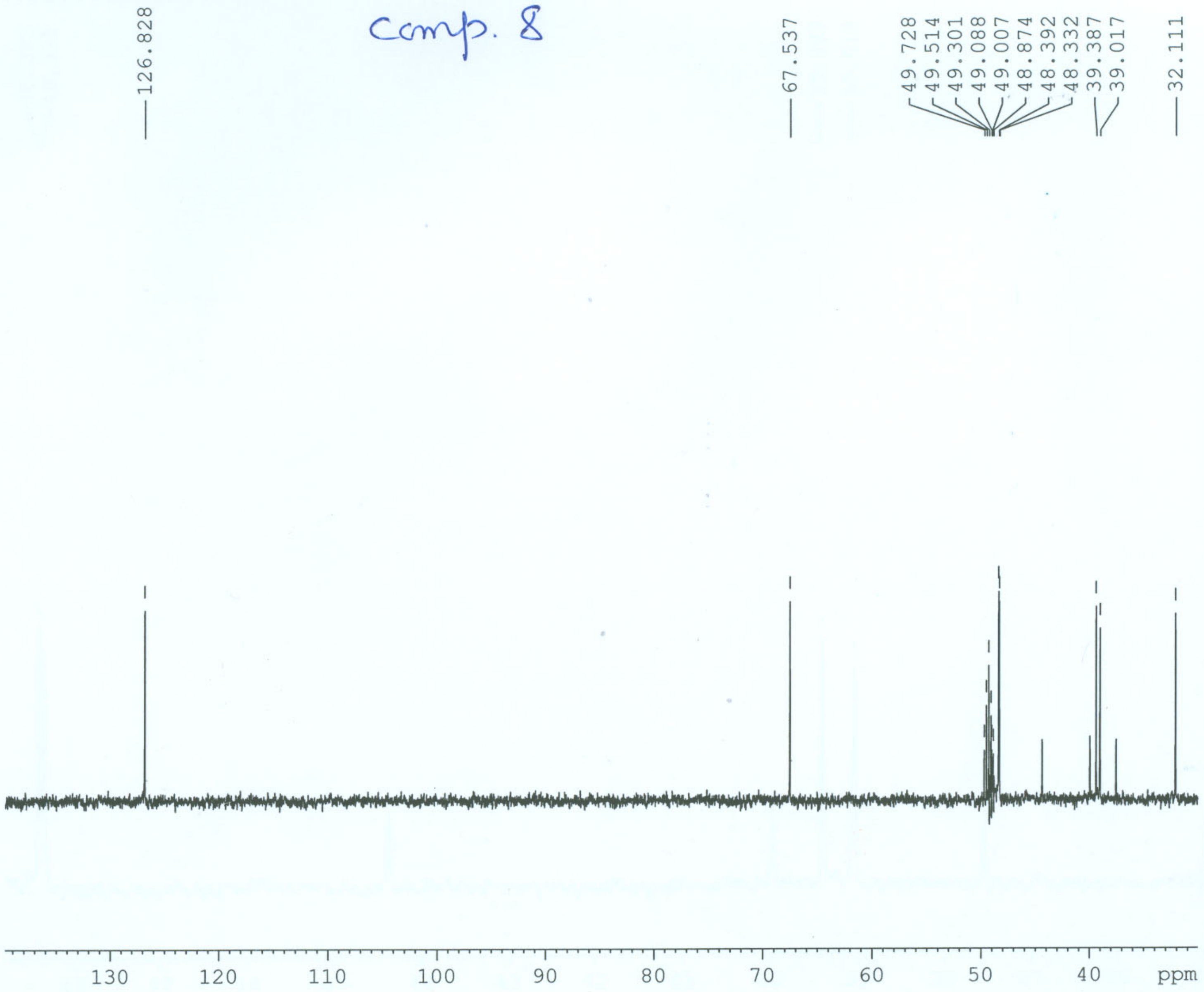


| | |
|---|---|
| NAME | sep17-15 |
| EXPNO | 11 |
| PROCNO | 1 |
| Date_ | 20150918 |
| Time | 5.20 |
| INSTRUM | spect |
| PROBHD | 5 mm DUL 13C-1 |
| PULPROG | dept90 |
| TD | 32768 |
| SOLVENT | MeOD |
| NS | 4096 |
| DS | 2 |
| SWH | 19157.088 Hz |
| FIDRES | 0.584628 Hz |
| AQ | 0.8552948 sec |
| RG | 14596.5 |
| DW | 26.100 usec |
| DE | 6.50 usec |
| TE | 300.0 K |
| CNST2 | 145.0000000 |
| D1 | 1.50000000 sec |
| D2 | 0.00344828 sec |
| D12 | 0.00002000 sec |
| TD0 | 4 |

======== CHANNEL f1 ========

| | |
|---|---|
| NUC1 | 13C |
| P1 | 8.10 usec |
| P2 | 16.20 usec |
| PL1 | 7.00 dB |
| SFO1 | 100.6220254 MHz |

======== CHANNEL f2 ========

| | |
|---|---|
| CPDPRG2 | waltz16 |
| NUC2 | 1H |
| P3 | 9.80 usec |
| P4 | 19.60 usec |
| PCPD2 | 80.00 usec |
| PL2 | 0.00 dB |
| PL12 | 20.00 dB |
| SFO2 | 400.1320007 MHz |
| SI | 32768 |
| SF | 100.6126253 MHz |
| WDW | EM |
| SSB | 0 |
| LB | 1.50 Hz |
| GB | 0 |
| PC | 1.40 |

MEHWISH/DR,IQBAL/JM-8/
ICCBS,U.O.K/DEPT-135

Comp. 8

AVANCE 400
LAB NO 117

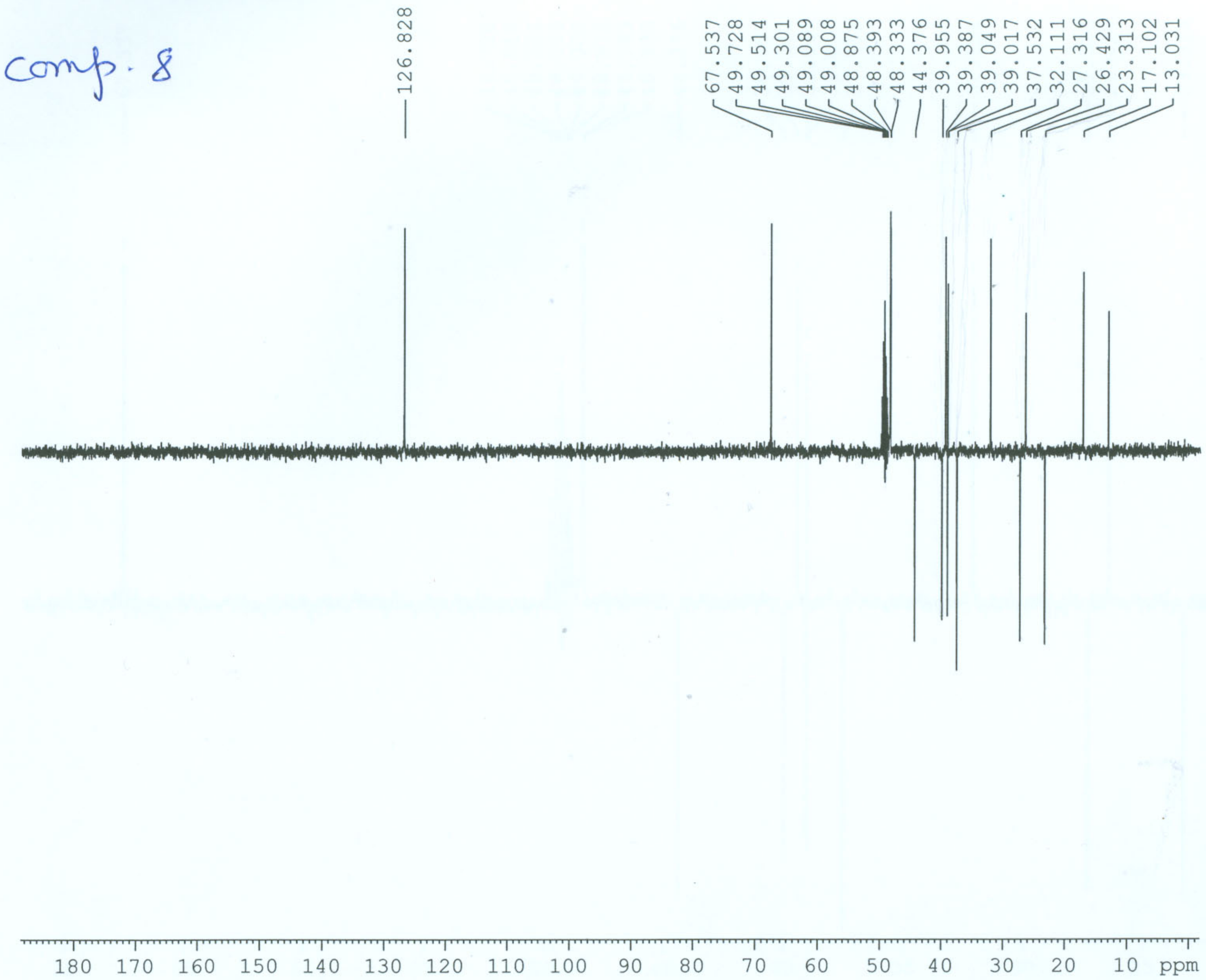


| | |
|---|---|
| NAME | sep17-15 |
| EXPNO | 10 |
| PROCNO | 1 |
| Date_ | 20150917 |
| Time | 23.11 |
| INSTRUM | spect |
| PROBHD | 5 mm DUL 13C-1 |
| PULPROG | dept135 |
| TD | 32768 |
| SOLVENT | MeOD |
| NS | 9216 |
| DS | 2 |
| SWH | 19157.088 Hz |
| FIDRES | 0.584628 Hz |
| AQ | 0.8552948 sec |
| RG | 32768 |
| DW | 26.100 usec |
| DE | 6.50 usec |
| TE | 300.0 K |
| CNST2 | 145.0000000 |
| D1 | 1.50000000 sec |
| D2 | 0.00344828 sec |
| D12 | 0.00002000 sec |
| TD0 | 9 |

======== CHANNEL f1 ========

| | |
|---|---|
| NUC1 | 13C |
| P1 | 8.10 usec |
| P2 | 16.20 usec |
| PL1 | 7.00 dB |
| SFO1 | 100.6220254 MHz |

======== CHANNEL f2 ========

| | |
|---|---|
| CPDPRG2 | waltz16 |
| NUC2 | 1H |
| P3 | 9.80 usec |
| P4 | 19.60 usec |
| PCPD2 | 80.00 usec |
| PL2 | 0.00 dB |
| PL12 | 20.00 dB |
| SFO2 | 400.1320007 MHz |
| SI | 32768 |
| SF | 100.6126253 MHz |
| WDW | EM |
| SSB | 0 |
| LB | 1.50 Hz |
| GB | 0 |
| PC | 1.40 |

Mehwish / Dr. Iqbal / Jm-8 / MeOD
HSQC

Comp. 8

AVANCE AV-600-LC
CRYOPROBE
LAB NO: 108

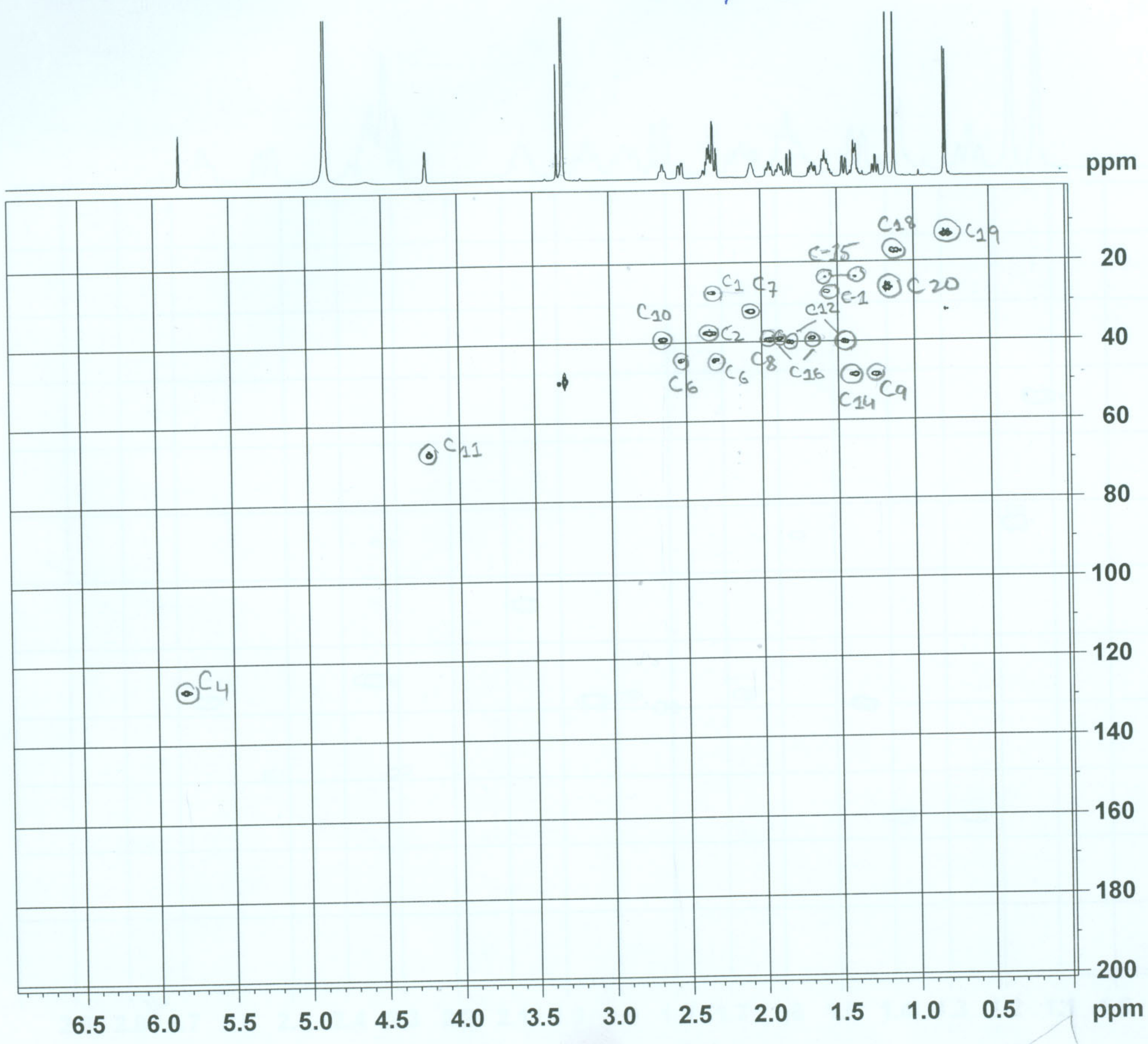


| | | |
|---|---|---|
| NAME | sep21-15 | |
| EXPNO | 13 | |
| PROCNO | 1 | |
| Date_ | 20150921 | |
| Time | 21.11 | |
| INSTRUM | spect | |
| PROBHD | 5 mm CPTCI 1H- | |
| PULPROG | hsqcetgpsi | |
| TD | 1024 | |
| SOLVENT | MeOD | |
| NS | 32 | |
| DS | 8 | |
| SWH | 4194.631 | Hz |
| FIDRES | 4.096319 | Hz |
| AQ | 0.1222300 | sec |
| RG | 29193 | |
| DW | 119.200 | usec |
| DE | 6.50 | usec |
| TE | 298.0 | K |
| CNST2 | 145.0000000 | |
| D0 | 0.00000300 | sec |
| D1 | 2.00000000 | sec |
| D4 | 0.00172414 | sec |
| D11 | 0.03000000 | sec |
| D13 | 0.00000400 | sec |
| D16 | 0.00020000 | sec |
| D24 | 0.00110000 | sec |
| IN0 | 0.00001655 | sec |
| ZGOPTNS | | |
| ======== CHANNEL f1 ======== | | |
| NUC1 | 1H | |
| P1 | 8.00 | usec |
| P2 | 16.00 | usec |
| P28 | 1000.00 | usec |
| PL1 | 3.31 | dB |
| PL1W | 6.79873323 | W |
| SFO1 | 600.0321001 | MHz |
| ======== CHANNEL f2 ======== | | |
| CPDPRG2 | garp | |
| NUC2 | 13C | |
| P3 | 11.50 | usec |
| P4 | 23.00 | usec |
| PCPD2 | 55.00 | usec |
| PL2 | -1.81 | dB |
| PL12 | 11.70 | dB |
| PL2W | 81.92915344 | W |
| PL12W | 3.65122390 | W |
| SFO2 | 150.8927518 | MHz |
| ====== GRADIENT CHANNEL ===== | | |
| GPNAM1 | SINE.100 | |
| GPNAM2 | SINE.100 | |
| GPZ1 | 80.00 | % |
| GPZ2 | 20.10 | % |
| P16 | 1000.00 | usec |
| ND0 | 2 | |

Mehwish / Dr. Iqbal / Jm-8 / MeOD
HMBC

Comp. 8

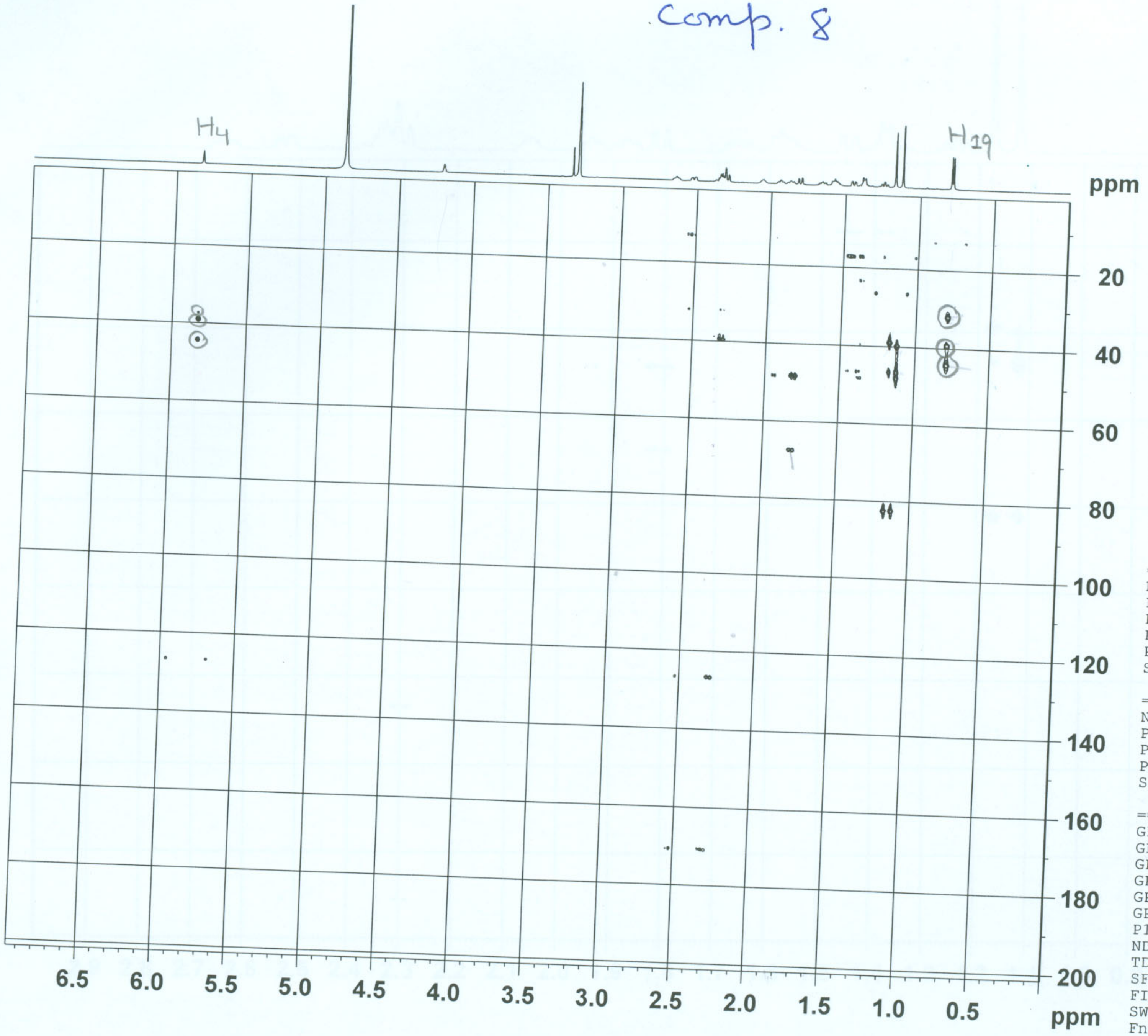


| | |
|---|---|
| NAME | sep21-15 |
| EXPNO | 14 |
| PROCNO | 1 |
| Date_ | 20150923 |
| Time | 16.49 |
| INSTRUM | spect |
| PROBHD | 5 mm CPTCI 1H- |
| PULPROG | hmbcgplpndqf |
| TD | 2048 |
| SOLVENT | MeOD |
| NS | 64 |
| DS | 16 |
| SWH | 4194.631 Hz |
| FIDRES | 2.048160 Hz |
| AQ | 0.2442908 sec |
| RG | 41285.1 |
| DW | 119.200 usec |
| DE | 6.50 usec |
| TE | 297.9 K |
| CNST2 | 145.0000000 |
| CNST13 | 13.0000000 |
| D0 | 0.00000300 sec |
| D1 | 2.00000000 sec |
| D2 | 0.00344828 sec |
| D6 | 0.03846154 sec |
| D16 | 0.00015000 sec |
| IN0 | 0.00001440 sec |

======== CHANNEL f1 ========

| | |
|---|---|
| NUC1 | 1H |
| P1 | 8.00 usec |
| P2 | 16.00 usec |
| PL1 | 3.31 dB |
| PL1W | 6.79873323 W |
| SFO1 | 600.0321001 MHz |

======== CHANNEL f2 ========

| | |
|---|---|
| NUC2 | 13C |
| P3 | 11.50 usec |
| PL2 | -1.81 dB |
| PL2W | 81.92915344 W |
| SFO2 | 150.8950149 MHz |

====== GRADIENT CHANNEL =====

| | |
|---|---|
| GPNAM1 | SINE.100 |
| GPNAM2 | SINE.100 |
| GPNAM3 | SINE.100 |
| GPZ1 | 50.00 % |
| GPZ2 | 30.00 % |
| GPZ3 | 40.10 % |
| P16 | 2000.00 usec |
| ND0 | 2 |
| TD | 256 |
| SFO1 | 150.895 MHz |
| FIDRES | 135.569733 Hz |
| SW | 230.000 ppm |
| FnMODE | QF |
| SI | 2048 |

Mehwish / Dr. Iqbal / Jm-8 / MeOD
cosy

Comp. 8

AVANCE AV-600-LC
CRYOPROBE
LAB NO: 108

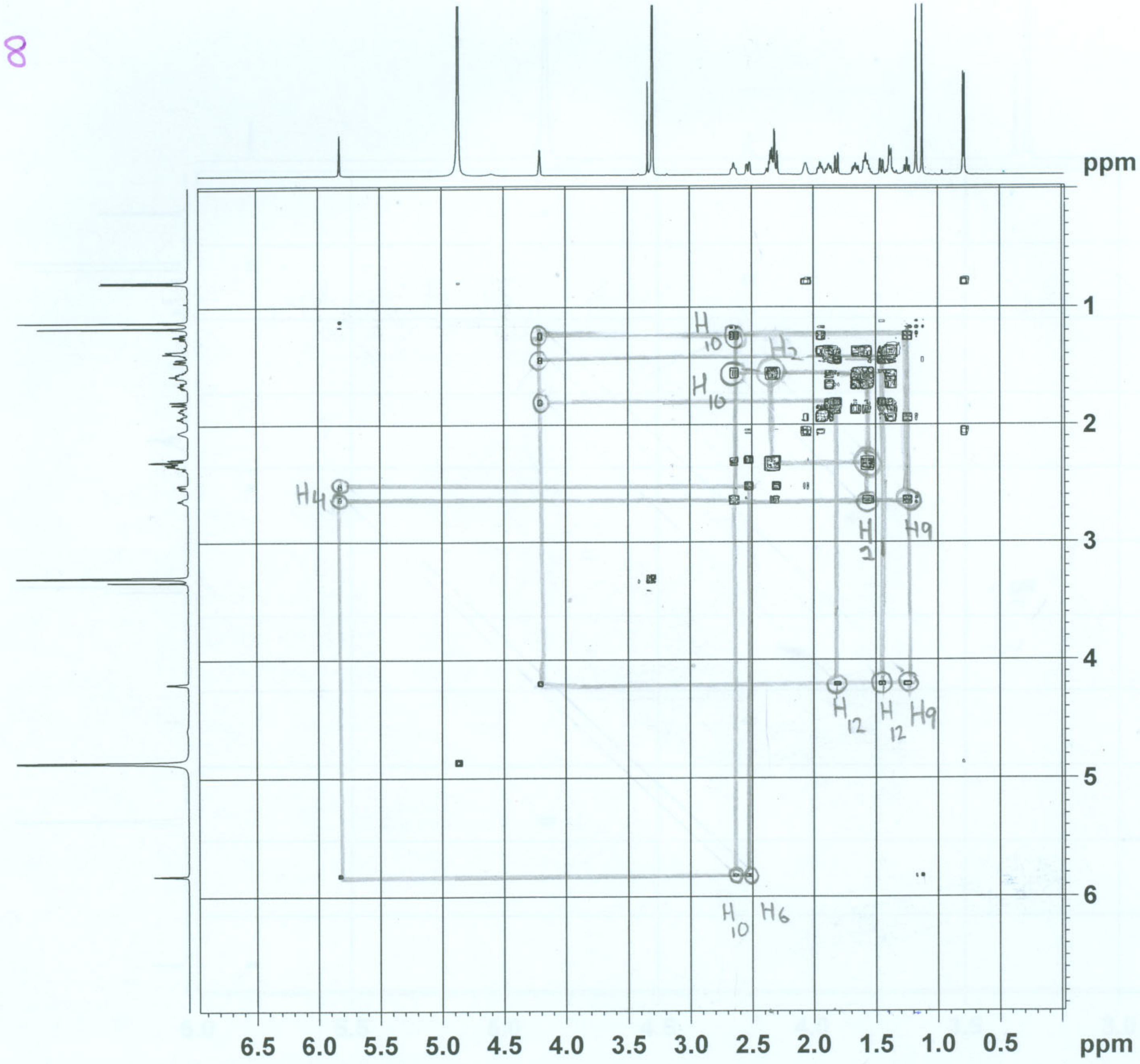


| | |
|---|---|
| NAME | sep21-15 |
| EXPNO | 10 |
| PROCNO | 1 |
| Date_ | 20150921 |
| Time | 16.22 |
| INSTRUM | spect |
| PROBHD | 5 mm CPTCI 1H- |
| PULPROG | cosydfqf |
| TD | 2048 |
| SOLVENT | MeOD |
| NS | 8 |
| DS | 4 |
| SWH | 4194.631 Hz |
| FIDRES | 2.048160 Hz |
| AQ | 0.2442908 sec |
| RG | 18 |
| DW | 119.200 usec |
| DE | 6.50 usec |
| TE | 298.0 K |
| D0 | 0.00000300 sec |
| D1 | 2.00000000 sec |
| D13 | 0.00000400 sec |
| D20 | 0.00000200 sec |
| IN0 | 0.00023840 sec |
| ======== CHANNEL f1 ======== | |
| NUC1 | 1H |
| P1 | 8.00 usec |
| PL1 | 3.31 dB |
| PL1W | 6.79873323 W |
| SFO1 | 600.0321001 MHz |
| ND0 | 1 |
| TD | 128 |
| SFO1 | 600.0321 MHz |
| FIDRES | 32.770554 Hz |
| SW | 6.991 ppm |
| FnMODE | QF |
| SI | 1024 |
| SF | 600.0300175 MHz |
| WDW | QSINE |
| SSB | 0 |
| LB | 0.00 Hz |
| GB | 0 |
| PC | 4.00 |
| SI | 1024 |
| MC2 | QF |
| SF | 600.0300175 MHz |
| WDW | QSINE |
| SSB | 0 |
| LB | 0.00 Hz |
| GB | 0 |

Mehwish / Dr. Iqbal / Jm-8 / MeOD
NOESY

Comp. 8

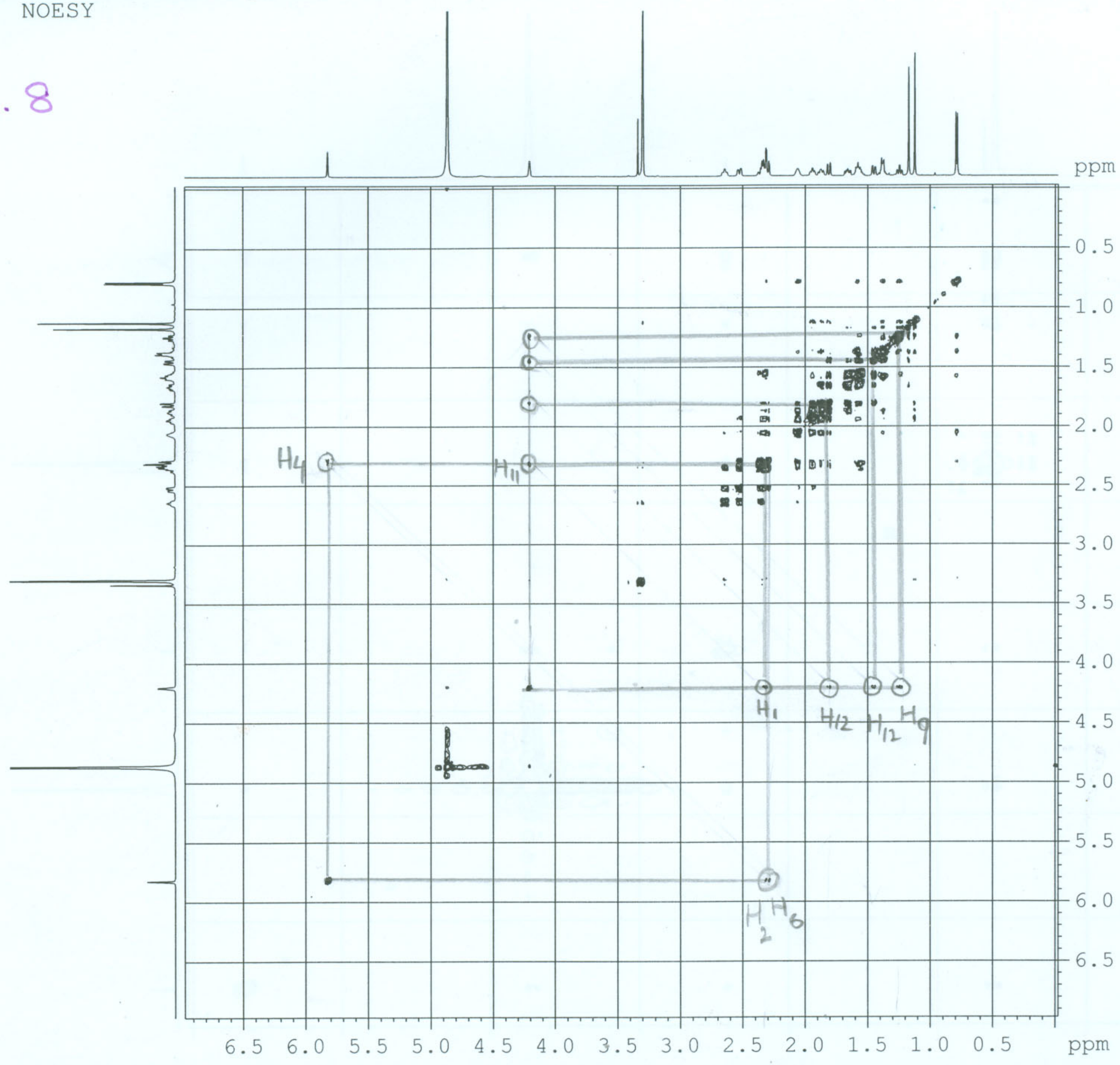


| | |
|---|---|
| NAME | sep21-15 |
| EXPNO | 12 |
| PROCNO | 1 |
| Date_ | 20150921 |
| Time | 17.41 |
| INSTRUM | spect |
| PROBHD | 5 mm CPTCI 1H- |
| PULPROG | noesygpph |
| TD | 2048 |
| SOLVENT | MeOD |
| NS | 16 |
| DS | 4 |
| SWH | 4194.631 Hz |
| FIDRES | 2.048160 Hz |
| AQ | 0.2442908 sec |
| RG | 28.5 |
| DW | 119.200 usec |
| DE | 6.50 usec |
| TE | 298.0 K |
| D0 | 0.00010901 sec |
| D1 | 2.00000000 sec |
| D8 | 0.80000001 sec |
| D16 | 0.00020000 sec |
| IN0 | 0.00023840 sec |

======== CHANNEL f1 ========

| | |
|---|---|
| NUC1 | 1H |
| P1 | 8.00 usec |
| P2 | 16.00 usec |
| PL1 | 3.31 dB |
| PL1W | 6.79873323 W |
| SFO1 | 600.0321001 MHz |

====== GRADIENT CHANNEL =====

| | |
|---|---|
| GPNAM1 | SINE.100 |
| GPNAM2 | SINE.100 |
| GPZ1 | 40.00 % |
| GPZ2 | -40.00 % |
| P16 | 1000.00 usec |
| ND0 | 1 |
| TD | 256 |
| SFO1 | 600.0321 MHz |
| FIDRES | 16.385277 Hz |
| SW | 6.991 ppm |
| FnMODE | States-TPPI |
| SI | 1024 |
| SF | 600.0300175 MHz |
| WDW | SINE |
| SSB | 2 |
| LB | 0.00 Hz |
| GB | 0 |
| PC | 4.00 |
| SI | 512 |
| MC2 | States-TPPI |
| SF | 600.0300175 MHz |
| WDW | SINE |
| SSB | 2 |
| LB | 0.00 Hz |
| GB | 0 |

comp. 8

## THERMO ELECTRON ~ VISIONpro SOFTWARE V4.10

| | | | |
|---|---|---|---|
| Operator Name | Arshad Alam | Date of Report | 10/8/2015 |
| Department | Analytical laboratory#004 TWC | Time of Report | 10:36:44AM |
| Organization | ICCBS.Karachi University. | | |
| Information | Porf . Dr. M. Iqbal / Mahwish. | | |

**Scan Graph**

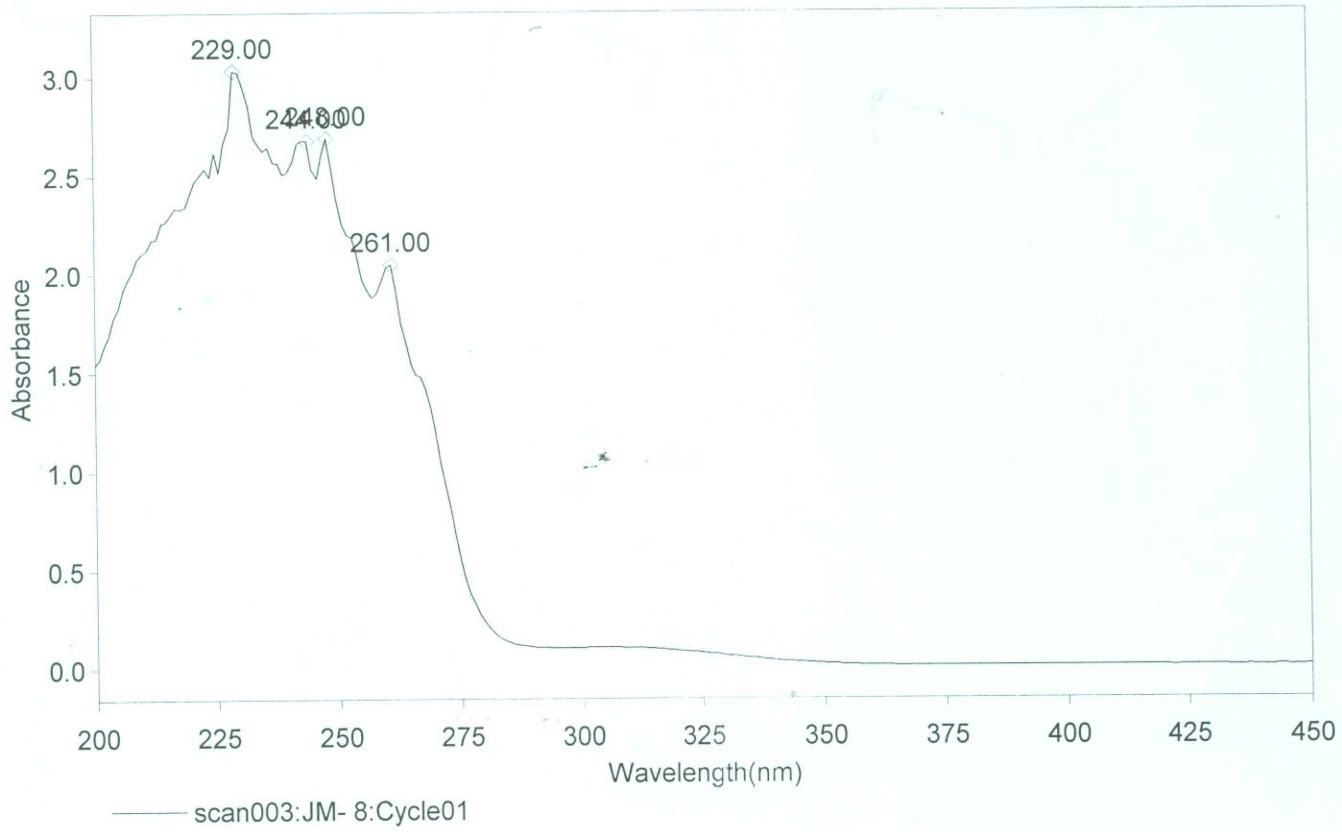


**Results Table - JM- 8.sre,JM- 8,Cycle01**

| nm | A | **Peak Pick Method** |
|---|---|---|
| 229.00 | 3.031 | Find 8 Peaks Above -3.0000 A |
| 244.00 | 2.675 | Start Wavelength 200.00 nm |
| 248.00 | 2.687 | Stop Wavelength 450.00 nm |
| 261.00 | 2.046 | Sort By Wavelength |
| Sensitivity | Medium | |

4ml → 0.5ml + 2ml

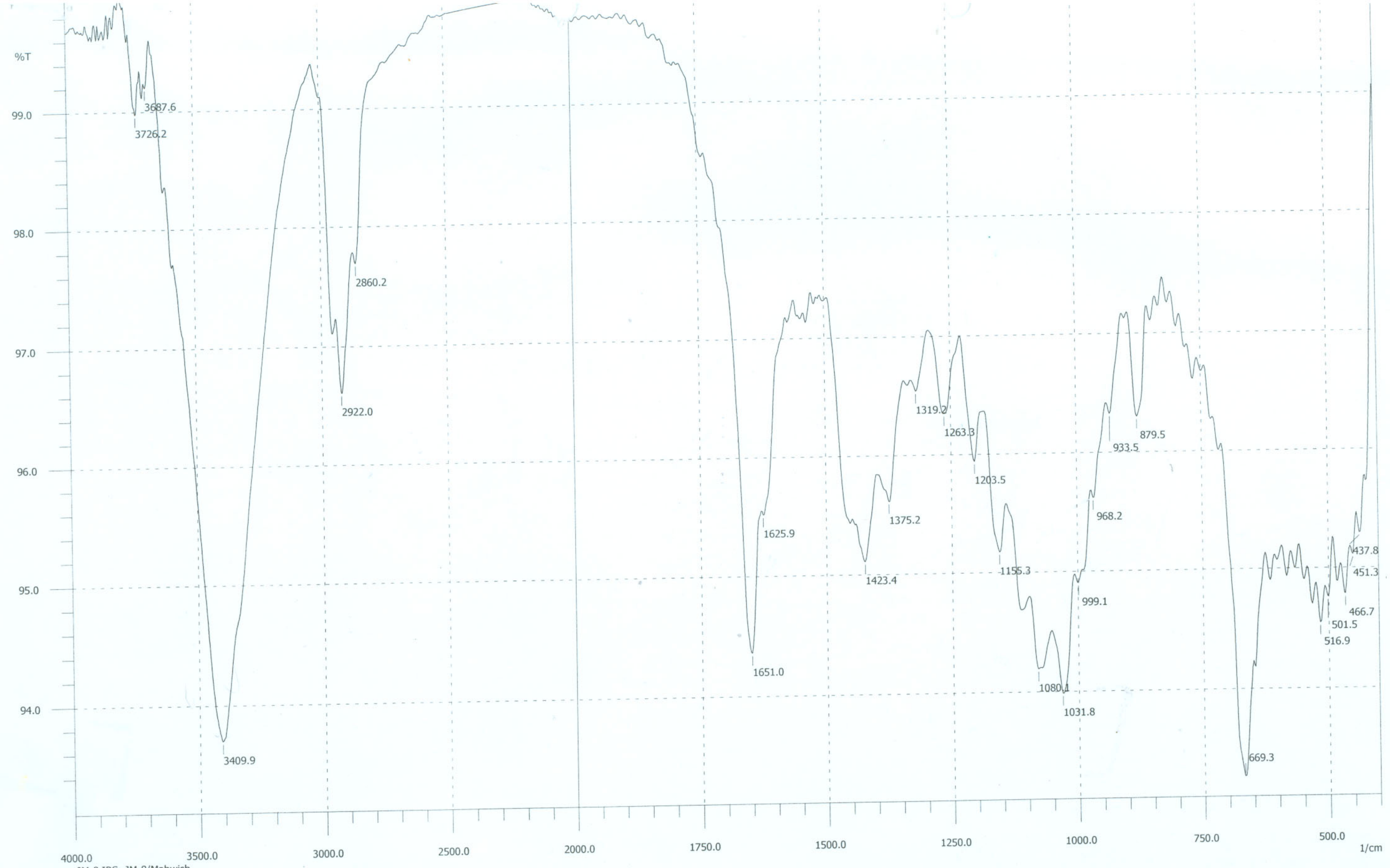


JM-8.IRS: JM-8/Mahwish

| | | | | | |
|---|---|---|---|---|---|
| Date: | 10/12/2015 | Time: | 11:28:49 | NScans: | 5 |
| Type: | HYPER IR | User: | Zubair Ahmed | Detector: | standard |
| Abscissa: | 1/cm | Ordinate: | %T | Apodization: | Happ |
| Min: | 401.17 | Max: | 3998.16 | Range: | 1/cm |
| Ndp: | 1866 | Data Interval: | 1.92868 | Resolution: | 4.0 |
| Gain: | auto | Aperture: | auto | Mirror Speed: | 2.8(low) |

Comp. 8
